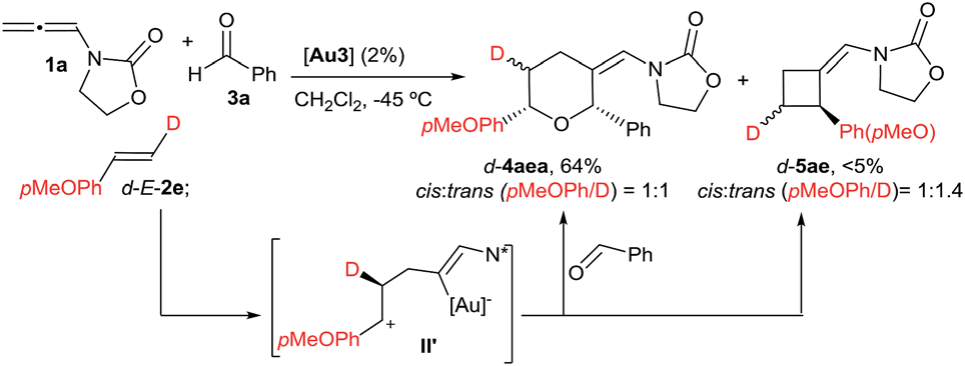# Gold(i)-catalyzed [2 + 2 + 2] cycloaddition of allenamides, alkenes and aldehydes: a straightforward approach to tetrahydropyrans†
†Electronic supplementary information (ESI) available: Characterization data and experimental procedures. CCDC 1038447–1038449. For ESI and crystallographic data in CIF or other electronic format see DOI: 10.1039/c5sc00295h



**DOI:** 10.1039/c5sc00295h

**Published:** 2015-03-02

**Authors:** Hélio Faustino, Iván Varela, José L. Mascareñas, Fernando López

**Affiliations:** a Centro Singular de Investigación en Química Biológica y Materiales Moleculares (CIQUS) and Departamento de Química Orgánica , Universidad de Santiago de Compostela , C/ Jenaro de la Fuente s/n , 15782 , Santiago de Compostela , Spain . Email: joseluis.mascarenas@usc.es ; Email: fernando.lopez@csic.es; b Instituto de Química Orgánica General (CSIC) , Juan de la Cierva 3 , 28006 , Madrid , Spain

## Abstract

Allenamides participate as two-carbon components in an intermolecular [2 + 2 + 2] cycloaddition with alkenes and aldehydes when treated with catalytic amounts of a phosphite gold complex. The reaction is highly regio- and chemoselective, and works with different types of alkenes, including styrenes, enol ethers or enamides, as well as with aromatic and aliphatic aldehydes. Accordingly, different types of 2,6-disubstituted tetrahydropyrans can be stereoselectively assembled in a single step from commercial or very accessible starting materials.

## Abstract 2

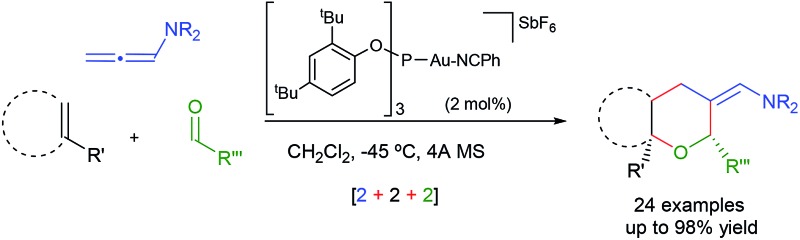
A novel fully intermolecular gold-catalyzed [2 + 2 + 2] cycloaddition involving an allenamide, an alkene and an aldehyde provides a straightforward entry to tetrahydropyrans.

## Introduction

Transition metal catalyzed [2 + 2 + 2] cycloadditions constitute one of the most attractive methodologies for the construction of six-membered cyclic systems.^1^ Despite the significant achievements reported in this field, intermolecular examples involving three different cycloaddition partners are extremely scarce, most probably because of the chemo- and regioselectivity issues associated with these multicomponent annulations.^2^ The few examples reported so far involve the use of Rh, Ru, Nb or Ni catalysts and at least one alkyne as cycloaddition component.^2^ Curiously, and despite the fact that gold catalysis has proven to be very efficient for unveiling novel types of cycloadditions,^3^ fully intermolecular [2 + 2 + 2] examples are almost unknown^4^ and, to the best of our knowledge, those of three different two-atom components are unprecedented.^5^

**Fig. 1:**
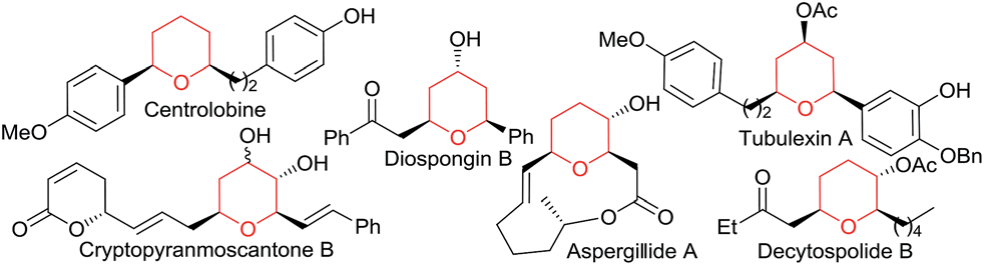
Tetrahydropyran frameworks in biologically active products.

Herein, we are pleased to report a fully intermolecular gold-catalyzed [2 + 2 + 2] cycloaddition involving three different π-unsaturated components, namely an allene, an alkene and an aldehyde. The reaction takes place with excellent chemo- and regioselectivity and provides a straightforward and atom-economical entry to tetrahydropyrans (THPs). THPs, and in particular their 2,6-disubstituted counterparts, are privileged scaffolds that are present in a myriad of biologically active molecules (Fig. 1).^6^ Although many elegant methods have been developed to construct these motifs,^6,7^ none of them encompass the coupling of three readily available components in a single catalytic annulation step.^8^

**Scheme 1:**
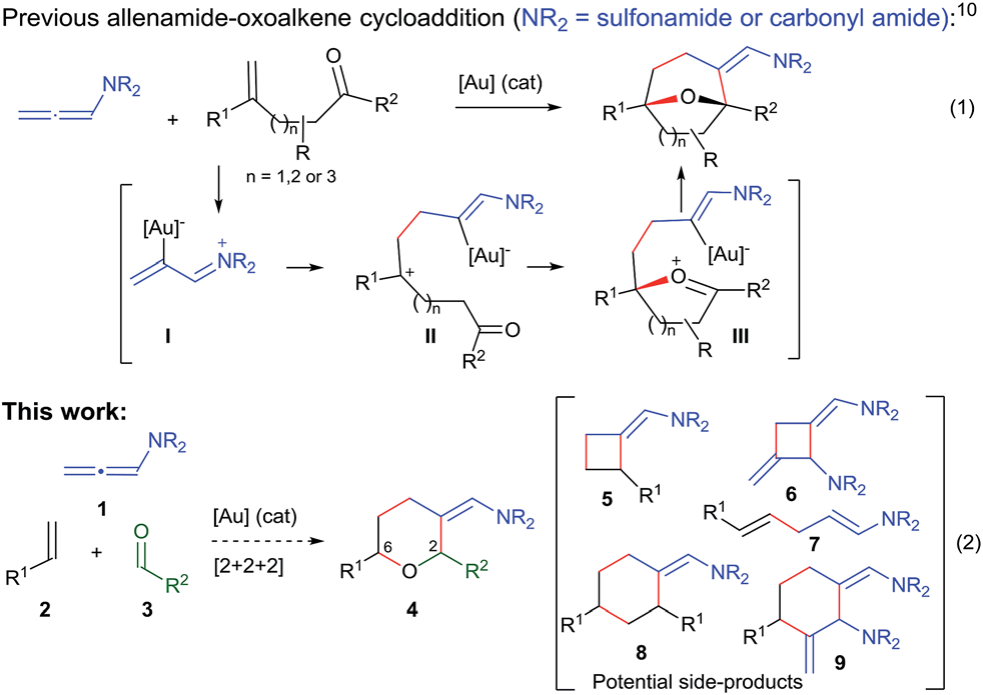
Previous gold-catalyzed cascade cycloadditions and current proposal.

Over the past few years, we have developed different types of Au-catalyzed annulations,^9^ including a cycloaddition between allenamides and oxoalkenes that affords oxabridged medium-sized carbocycles (Scheme 1, eqn (1)).^10,11^ This annulation was proposed to proceed through the intermediate **I**,^12^ which evolves to the product by the sequential formation of species **II** and **III**. On this basis, we then wondered whether it would be possible to achieve an annulation between the allenamide, alkene and carbonyl units in a fully intermolecular way, a process that would directly afford 2,6-disubstituted THPs like **4** (Scheme 1, eqn (2)). Despite the fact that the process could be viewed as an intermolecular version of the previous annulation, the timely assembly of three different components in a programmed manner is extremely challenging. Indeed, the feasibility of the reaction could be seriously compromised since more simple [2 + 2] adducts of type **5** and **6**,^9*c*^ acyclic products like **7**, or alternative [2 + 2 + 2] adducts (**8**/**9**) could be likewise expected.^13^

## Results and discussion

We began our studies by analyzing the reactivity of allenamide **1a** with (*E*)-β-methylstyrene (**2a**) and benzaldehyde (**3a**) (Table 1). Initial assays confirmed the expected difficulties for controlling the chemoselectivity of the process. Indeed, despite using an excess of the aldehyde (10 equiv.), and adding the allenamide over 2 hours, the gold complex **Au1** induced the formation of the [2 + 2] allenamide dimerization adduct **6a** in 44% yield, together with a minor amount of the cyclobutane **5aa**,^9*c*^ resulting from the [2 + 2] cycloaddition between **1a** and **2a** (entry 1). A [2 + 2 + 2] adduct, eventually identified as the 2,6-*cis* THP **4aaa**, was also detected, but only in trace amounts. Similarly, other frequently used gold catalysts such as Ph_3_PAuNTf_2_ or the NHC–gold complex **Au2** provided very low yields of the [2 + 2 + 2] adduct **4aaa** (entries 2 and 3), with poor mass recovery balances in all these cases. Interestingly, when using the phosphite-gold complex **Au3**, we observed a significant increase in the global yield of the reaction, which provided **5aa** in 60% yield along with the [2 + 2 + 2] adduct **4aaa** in 21% yield (entry 4). This last yield could be further improved up to 35% by carrying out the reaction at –45 °C (entry 5).^14^

**Table 1:** Preliminary evaluation of the [2 + 2 + 2] cycloaddition^*a*^ ^,^ ^*b*^

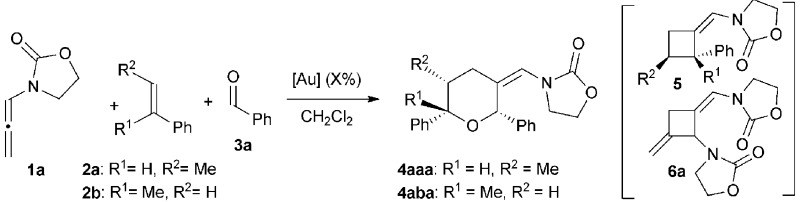								
Entry	[Au] (mol%)	**2**	R^1^	R^2^	Conv.	**4** (%)	**5** (%)	**6** (%)
1	**Au1** (5%)	**2a**	H	Me	99%	**4aaa**, 2	**5aa**, 4	**6a**, 44
2	Ph_3_PAuNTf_2_ (5%)	**2a**	H	Me	60%	**4aaa**, 2	**5aa**, 0	**6a**, 7
3	**Au2** (5%)	**2a**	H	Me	99%	**4aaa**, 15	**5aa**, 7	**6a**, 22
4	**Au3** (2%)	**2a**	H	Me	99%	**4aaa**, 21	**5aa**, 60	**6a**, 8
5^*c*^	**Au3** (2%)	**2a**	H	Me	99%	**4aaa**, 35	**5aa**, 37	—
6	**Au3** (2%)	**2b**	Me	H	99%	**4aba**, 98^*d*^	—	—
7^*e*^	**Au3** (2%)	**2b**	Me	H	99%	**4aba**, 99^*d*^	—	—
8	**Au1** (2%)	**2b**	Me	H	99%	**4aba**, 51^*d*^	**5ab**, 17	—
9	Ph_3_PAuNTf_2_ (2%)	**2b**	Me	H	99%	**4aba**, 77^*f*^	**5ab**, 14	—
10	**Au2** (2%)	**2b**	Me	H	99%	**4aba**, 80^*d*^	**5ab**, 6	—
11^*e*^ ^,^ ^*g*^	**Au3** (2%)	**2b**	Me	H	99%	**4aba**, 98^*h*^	—	—
12^*e*^ ^,^ ^*i*^	**Au3** (2%)	**2b**	Me	H	99%	**4aba**, 98^*j*^	—	—

^*a*^ **1a** (1 equiv.) added over 2 h to a solution of **2** (2 equiv.), **3a** (10 equiv.), [Au] (*X* mol%) and 4 Å MS, in CH_2_Cl_2_ at –15 °C, unless otherwise noted.

^*b*^Conversion of **1a** and yields of **4–6** determined by ^1^H-NMR of the crude mixture using 1,3,5-(MeO)_3_C_6_H_3_ as internal standard (IS).

^*c*^Carried out at –45 °C, (1 h).

^*d*^Overall yield for the mixture of 2,6-*cis* (**4aba**) and *trans* (**4aba′**); dr = 2 : 1. The major isomer is that drawn.

^*e*^ **1a** added in one portion.

^*f*^Overall yield. dr 1.5 : 1.

^*g*^Carried out at –78 °C, (1 h).

^*h*^90% overall isolated yield, dr 3.5 : 1 (**4aba** : **4aba′**).

^*i*^Carried out in F_3_C–Ph at –25 °C (4 h).

^*j*^86% overall isolated yield, dr 4.5 : 1. 
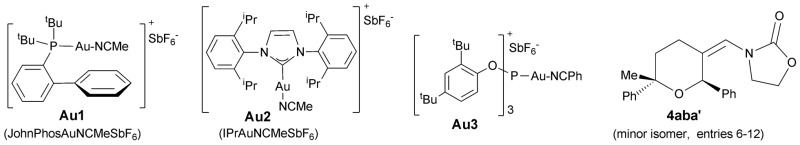

At this point, we envisioned that an additional stabilization of the putative carbocationic species of type **II**, resulting from the addition of the alkene to intermediate **I** (Scheme 1), could eventually facilitate its intermolecular capture by the aldehyde.

In consonance with this hypothesis, we were pleased to find that the use of α-methylstyrene (**2b**) instead of β-methylstyrene (**2a**) provided, under otherwise identical conditions, the desired THP in an excellent 98% yield, as a 2 : 1 mixture of 2,6-*cis* (**4aba**) and 2,6-*trans* (**4aba′**) diastereoisomers (entry 6).^15^ The same result was obtained when **1a** was added in one portion (entry 7). Gold catalysts such as JohnPhosAuNCMeSbF_6_ (**Au1**), Ph_3_PAuNTf_2_ or IPrAuNCMeSbF_6_ (**Au2**), also provided the desired [2 + 2 + 2] cycloadduct **4aba** as the major adduct; however, yields and chemoselectivities were significantly lower than those obtained with the phosphite–gold catalyst **Au3** (entry 7 *vs.* 8–10). Moreover, with this latter catalyst the diastereoselectivity could be improved by either performing the reaction at –78 °C (dr 3.5 : 1, 90% isolated yield, entry 11) or by using α,α,α-trifluorotoluene as solvent (dr 4.5 : 1, 86% yield, entry 12).

With these results in hand, we next analyzed the scope of the process (Table 2). In consonance with the performance of β-methylstyrene (**2a**, Table 1, entry 5), the cycloaddition of styrene (**2c**) with **1a** and benzaldehyde provided the desired 2,6-disubstituted THP (**4aca**) in a moderate 37% yield, but with complete 2,6-*cis* selectivity (**5ac** was also isolated in 45% yield). Gratifyingly, use of styrenes with electron-donating groups (*e.g. p*-MeO or *o*-MeO) allowed significant improvement of the chemoselectivity, so the corresponding THPs, **4ada** and **4aea**, were isolated in good yields (60–65% yield) and with complete 2,6-*cis* diastereoselectivity.

**Table 2:** Scope of the Au-catalyzed [2 + 2 + 2] intermolecular cycloaddition^*a*^

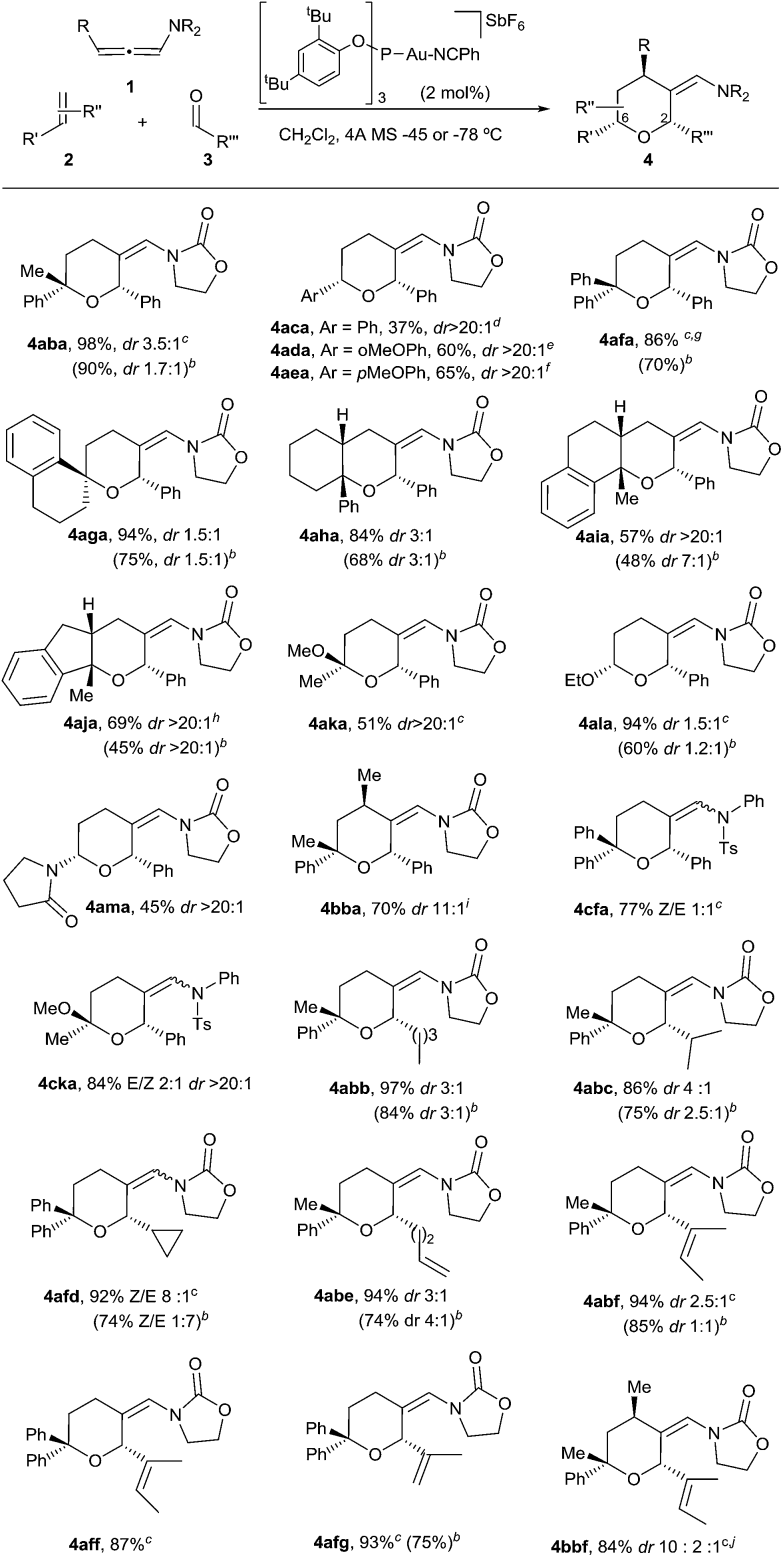

^*a*^ **1** (1 equiv.) added to a solution of **2** (2 equiv.), aldehyde (10 equiv.), [**Au3**] (2 mol%) and 4 Å MS, in CH_2_Cl_2_ at –45 °C, unless otherwise noted. Conversions >99% (^1^H-NMR). When a mixture of 2,6-isomers is formed, the major is that drawn.

^*b*^Carried out at –45 °C with a **1**/**2**/**3** molar ratio of 1/1.25/2.

^*c*^Carried out at –78 °C.

^*d*^45% of **5ac** was also isolated.

^*e*^21% of **5ad** was also isolated.

^*f*^Traces of **5ae** and **8ae** (5% yield) were also isolated.

^*g*^Traces of **7af** (5% yield) were also isolated.

^*h*^Traces of **7aj** (5% yield) were also isolated.

^*i*^17% yield of **5bb** was also isolated.

^*j*^For the structure of the minor isomers, see the ESI. 
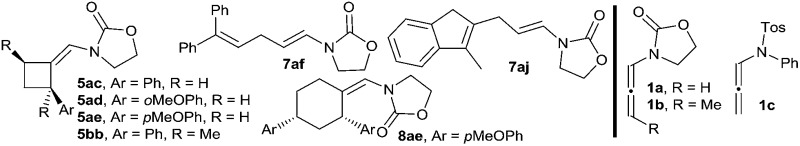

On the other hand, the cycloaddition with α-phenylstyrene provided the desired THP (**4afa**) in an excellent 86% yield, whereas the use of *exo*-methylenes such as 1-methylene-tetrahydronaphthalene allowed an efficient access to spirotetrahydropyran derivatives like **4aga**, which was isolated in an excellent 94% yield (dr 1.5 : 1).^16^

**Fig. 2:**
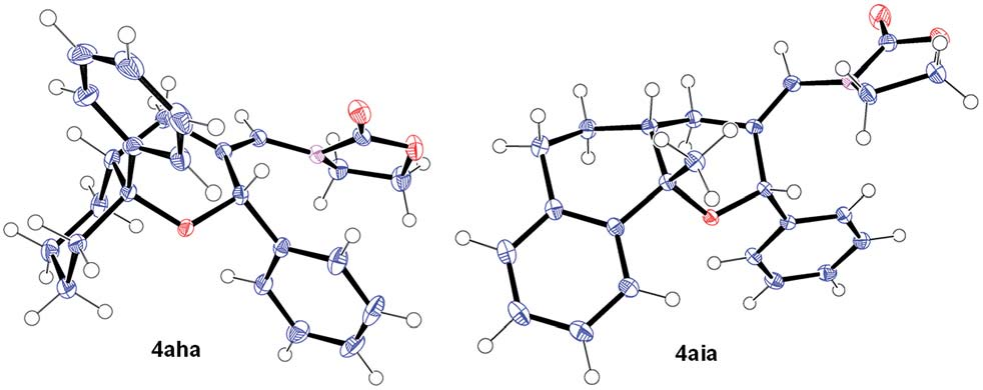
X-ray structures of **4aha** (left, major isomer) and **4aia** (right).^14^

Remarkably, cyclic alkene derivatives were also excellent partners for this process. Thus, the cycloadditions of allenamide **1a** and benzaldehyde with 1-phenylcyclohexene, 4-methyl-1,2-dihydronaphthalene or 3-methyl-1*H*-indene provided the corresponding THPs (**4aha–4aja**) in good yields (57–84% yield) and moderate (**4aha**) or complete (**4aia–aja**) stereoselectivity.^17^ X-ray analysis of crystals of **4aha** and **4aia** unambiguously confirmed their structures and relative stereochemistry (Fig. 2).^14^

We next explored the use of alternative electron-rich alkenes. Gratifyingly, the cycloaddition could also be performed with enol ethers such as 2-methoxyprop-1-ene or ethoxyethene, to obtain the corresponding cyclic acetals (**4aka–4ala**) with moderate to good yields. Similarly, the cycloaddition between **1a**, **3a** and 1-vinylpyrrolidin-2-one was also feasible, providing the cyclic hemiaminal ether **4ama** in 45% yield and with complete diastereoselectivity.

These annulations are also feasible with other allenamides. For instance, the reaction of γ-methyl-substituted allenamide **1b** (see Table 2, footnote) with α-methylstyrene and benzaldehyde provided the [2 + 2 + 2] adduct **4bba**, featuring three new stereogenic centers, in 70% yield and with excellent diastereoselectivity (dr 11 : 1).^18^ On the other hand, *N*-tosylphenyl allenamides such as **1c** were also suitable partners. Thus, the [2 + 2 + 2] adduct **4cfa**, resulting from the cycloaddition of **1c**, benzaldehyde and α-phenylstyrene was obtained in 77% yield, whereas the adduct **4cka**, from 2-methoxyprop-1-ene, was obtained in 84% yield and, importantly, with complete stereoselectivity.

Remarkably, the scope of the method is not limited to benzaldehyde. Indeed, the reaction of α-methylstyrene, allenamide **1a** and an aliphatic aldehyde such as pentanal led to the desired adduct, **4abb**, in 97% yield (dr 3 : 1). Other aldehydes such as isobutyraldehyde, cyclopropanecarbaldehyde or pent-4-enal also gave the THPs **4abc–4abe** in excellent yields. α,β-Unsaturated aldehydes such as 2-methylbut-2-enal or methacrolein also participated in the annulation yielding the desired THPs (**4abf**, **4aff**, **4afg**) in yields above 90%. Moreover, the cycloaddition of the γ-substituted allenamide **1b** with an aliphatic aldehyde such as 2-methylbut-2-enal was also feasible, providing **4bbf** in 84% yield (dr 10 : 2 : 1).^14^

Overall, it is important to highlight that the current method constitutes one of the very few catalytic approaches that affords THPs featuring fully substituted carbons at the oxygen-adjacent position (*e.g.* C6).^19^ On the other hand, while the above reactions were carried out using a relatively large excess of the aldehyde, gratifyingly, we found that in most of the cases the reaction can be efficiently performed using an allenamide (**1**)/alkene (**2**)/aldehyde (**3**) molar ratio of 1/1.2/2 (Table 2, footnote *b*, results in parentheses). Thus, using these conditions, THPs **4aba**, **4afa**, **4aga**, **4ala**, **4abb**, **4abc**, **4afd**, **4abe**, **4abf** or **4afg** were obtained in yields varying from 60% to 90% (Table 2).^20^ Additionally, more complex polycyclic systems like **4aha–4aja** could also be obtained in yields from 45% to 68%.^14^

**Scheme 2:**
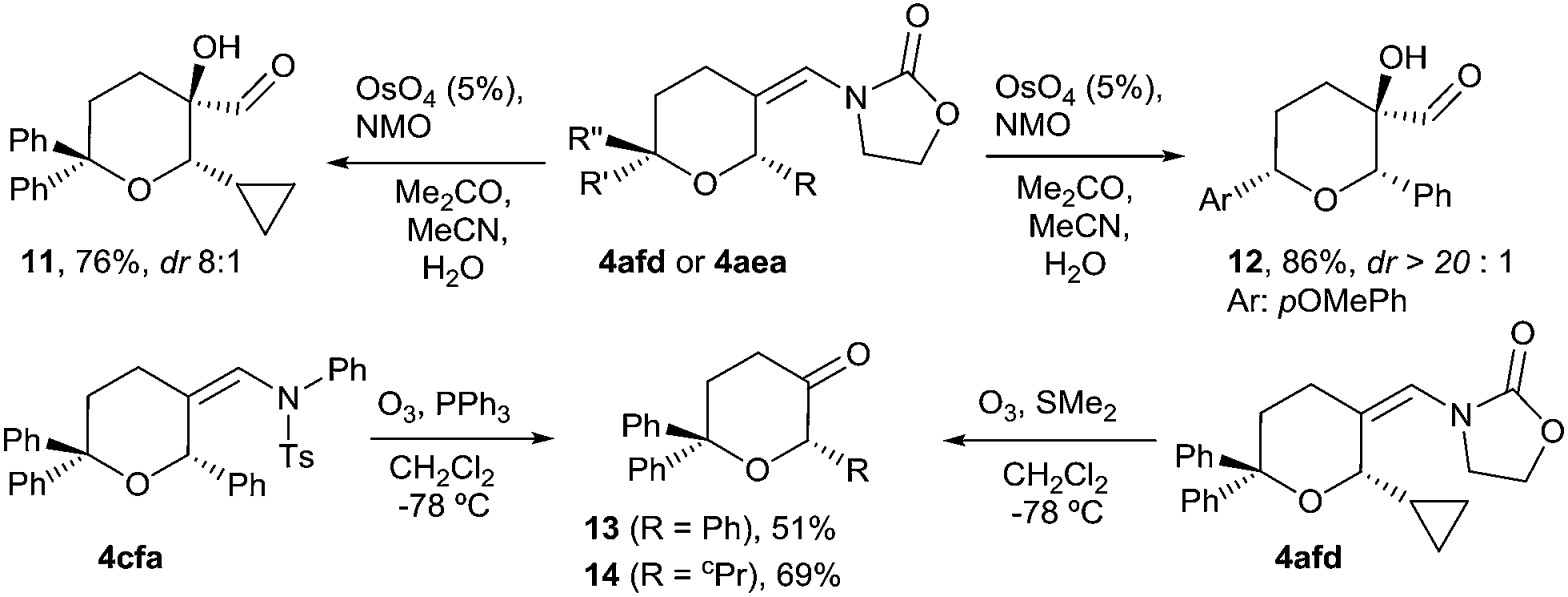
Functionalization of the *exo*-enamide moiety.

We next explored some manipulations of the *exo*-enamide moiety of the products (Scheme 2). Thus, THPs the like **4afd** or **4aea** can be dihydroxylated to afford the α-hydroxo aldehydes **11** and **12** in excellent yields and with very good or complete diastereoselectivity (Scheme 2, eqn (1)). Moreover, both types of enamides (*e.g.* **4afd** and **4cfa**) could be easily converted into their corresponding ketones upon ozonolysis (Scheme 2, eqn (2)).

**Scheme 3:**
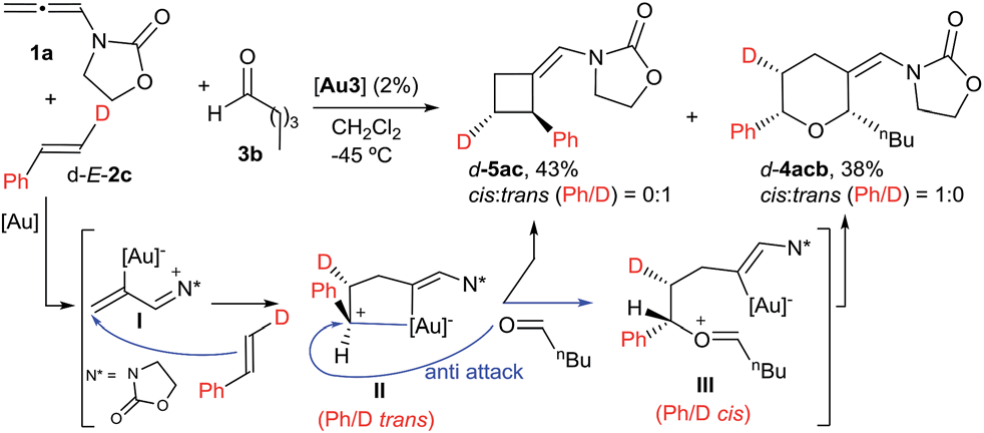
Cycloaddition of d-*E*-**2c** and the proposed key intermediate **II**.

With regard to the mechanism of the annulation, the general proposal indicated in Scheme 1 could also apply for this intermolecular process; however, we found some results that were indicative of a more complex scenario. In particular, it is curious that while the [2 + 2] product (**5aa**) obtained from **1a** and *E*-β-methylstyrene retains the *trans* configuration of the alkene, the [2 + 2 + 2] adduct **4aaa** displays these groups in a *cis* disposition (Table 1, entry 5). On the contrary, polycyclic [2 + 2 + 2] adducts like **4aha**, **4aia** or **4aja** retained the configuration of the parent alkene. To shed light on this divergence, we carried out the cycloaddition of **1a** and pentanal with the *trans*-deuterated styrene d-*E*-**2c** (Scheme 3). As expected, the reaction provided a mixture of the [2 + 2 + 2] and [2 + 2] adducts d-**4acb** (38% yield) and d-**5ac** (43% yield), respectively. Interestingly, d-**5ac** incorporates the Ph and the deuterium atom in a *trans* disposition, whereas the [2 + 2 + 2] adduct, d-**4acb**, holds these groups in a *cis* arrangement. These results strongly suggest the formation of an intermediate of type **II** that preserves the stereochemical information of the alkene due to an stabilizing electrostatic interaction between the gold atom and the benzylic carbocation (Scheme 3).^21^ A subsequent nucleophilic *anti* attack of the carbonyl moiety would lead to intermediate **III** and, eventually, to the product d-**4acb**. The preferential formation of this THP with the C2 and C6 substituents in *cis* is in agreement with a transition state that places these groups in equatorial disposition (Prins-like cyclization from **III** to **4**). On the other hand, if species **II** collapses to render a [2 + 2] adduct, the Ph and the D atom would retain the initial *trans* arrangement, as observed in d-**5ac**.

**Scheme 4:**
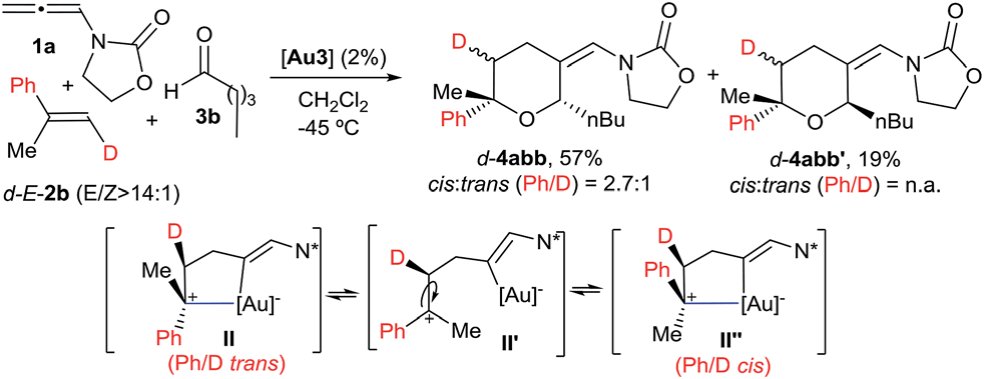
Cycloaddition of d-*E*-**2b** and the proposed key intermediate **II′**.

We also analysed the cycloaddition with deuterated α-methylstyrene (d-**2b**) as a model for α-substituted alkenes (Scheme 4). Curiously, the expected [2 + 2 + 2] isomeric adducts d-**4abb** and d-**4abb′** were obtained as mixtures of *cis*/*trans* (Ph/D) isomers. Accordingly, an acyclic carbocation species like **II′** or, alternatively, a fast equilibrium between the Ph/D-*trans* and *cis* intermediates **II** and **II′′**, could account for this result.^22,23^ Considering this proposal, the exclusive formation of the *cis*-fused polycyclic THPs **4aha–4aja** from cyclic alkene precursors can be also understood.

Finally, we carried out the above cycloadditions of Schemes 3 and 4 using the NHC–gold catalyst **Au2**, instead of **Au3**. Not unexpectedly, lower chemoselectivities and yields of the corresponding [2 + 2 + 2] adducts were obtained in both cases but, interestingly, the stereochemistry of each deuterated cycloadduct (d-**4acb**, d-**5ac** and d-**4abb**), turned out to be identical to that obtained with **Au3**.^14^ Thus, the σ-donor or π-acceptor characteristics of the ligand at the gold atom do not seem to significantly affect the nature of the intermediate of type **II**.

## Conclusions

In summary, we have developed a gold-catalyzed fully intermolecular [2 + 2 + 2] cycloaddition that constitutes one of the few transition metal catalyzed annulations involving three different π-unsaturated components. The process shows a broad scope with regard to the alkenes and aldehydes that can be used, and provides an efficient, atom-economical and stereoselective access to a variety of 2,6-disubstituted THPs from easily accessible or even commercially available materials.
