# Supplementary material for: Gold(i)-catalyzed [2 + 2 + 2] cycloaddition of allenamides, alkenes and aldehydes: a straightforward approach to tetrahydropyrans
Source: Chem Sci. 2015 Mar 2;6(5):2903–8. doi: 10.1039/c5sc00295h (PMC5655898; doi:10.1039/c5sc00295h)

Electronic Supplementary Material (ESI) for Chemical Science.
This journal is © The Royal Society of Chemistry 2015

ELECTRONIC SUPPLEMENTARY INFORMATION

Gold(I)-catalyzed [2+2+2] Cycloaddition of Allenamides, Alkenes and Aldehydes: A Straightforward Approach to Tetrahydropyrans

Hélio Faustino,[a,†] Iván Varela,[a,†] José L. Mascareñas*,[a] and Fernando López*,[a,b]

[a] Centro Singular de Investigación en Química Biológica y Materiales Moleculares (CIQUS) and Departamento de Química Orgánica. Universidad de Santiago de Compostela. C/ Jenaro de la Fuente, s/n, 15782, Santiago de Compostela, Spain.

[b] Instituto de Química Orgánica General (CSIC). Juan de la Cierva, 3, 28006, Madrid, Spain.

Contents:

General Procedures S2

Experimental data S3

Mechanistic experiments with deuterated alkenes S23

NMR Spectra S36

General Procedures

Dry solvents were freshly distilled under argon from an appropriate drying agent before use. Dry THF was obtained using Solvent Purification System (SPS). Toluene and CH_2Cl_2 was purchased from Aldrich. Gold complexes were prepared according to previously reported methods[1,2,3,4] or purchased from Aldrich. 3-(Propa-1,2-dien-1-yl)oxazolidin-2-one (**1a**),[5] 4-methyl-*N*-phenyl-*N*-(propa-1,2-dien-1-yl)benzenesulfonamide (**1b**),[6] 1-methylene-1,2,3,4-tetrahydronaphthalene (**2g**),[7] are known compounds and were synthesized following reported procedures. (*E*)-*ß* –Deuteriostyrene (*d*-*E*-**2c**) and *E*)-*ß* -Deuterio-1-methoxy-4-vinylbenzene (*d*-*E*-**2e**) were prepared from the corresponding alkyne, the Schwartz' reagent and D_2O, according to a reported procedure.[8] The spectral data of both compounds is in agreement with that previously reported.[9] (*E*)-(prop-1-en-2-yl-1-*d*)benzene (*d*-*E*-**2b**) was prepared according to a reported procedure,[10] using dichloromethane as solvent. 4-Methyl-1,2-dihydronaphthalene (**2i**),[11] and 3-methyl-1H-indene (**2j**)[,12] were synthesized following reported procedures,[11] in 97% and 65% yield from MeMgBr and 1-tetralone or 2,3-dihydro-1H-inden-1-one, respectively. All other alkenes and aldehydes used were bought from Aldrich, Alfa Aesar, TCI or Acros and used without further purification. Reactions were conducted in dry solvents under argon atmosphere unless otherwise stated. The abbreviation "*rt*" refers to reactions carried out approximately at 23ºC. Reaction mixtures were stirred using Teflon-coated magnetic stirring bars. Reaction temperatures were maintained using Thermowatch-controlled silicone oil baths. Thin-layer chromatography (TLC) was performed on silica gel plates and components were visualized by observation under UV light, and / or by treating the plates with *p*-anisaldehyde or cerium nitrate solutions, followed by heating. Flash chromatography was carried out on silica gel unless otherwise stated. Dryings were performed with anhydrous Na_2SO_4 or $MgSO_4$. Concentration refers to the removal of volatile solvents via distillation using a Büchi rotary evaporator followed by residual solvent removal under high vacuum. NMR spectra were recorded in $CDCl_3$, at 300 MHz (Varian) or 500 MHz (Varian). Carbon types and structure assignments were determined from DEPT-NMR and two-dimensional experiments (HMQC and HMBC, COSY and NOESY). NMR spectra were analyzed using MestreNova© NMR data processing software (www.mestrelab.com). The following abbreviations are used to indicate signal multiplicity: s, singlet; d, doublet; t, triplet; q, quartet; p, pentet, dd, double doublet; td, triple doublet; m, multiplet; br, broad. Mass spectra were acquired using chemical ionization (CI) electron impact (EI), or electrospray ionization (ESI) and were recorded at the CACTUS facility of the University of Santiago de Compostela. The reactions were monitored by TLC or GC-MS using the Agilent Technologies 6890N, Network GC System, equipped with the Agilent 190915-433 column and the Agilent 5973 Inert Mass Selective Detector in Electron Impact or Chemical Ionization Mode (with Methane). X-Ray diffraction experiments were carried out in a Brucker SMART 1000 difractometer.

---

[1] C. H. M. Amijs, V. López-Carrillo, M. Raducan, P. Pérez-Galán, C. Ferrer and A. M. Echavarren, *J. Org. Chem.,* 2008, **73**, 7721.
[2] P. de Frémont, N. Marion and S. P. Nolan, *J. Organomet. Chem.* 2009, **694**, 551.
[3] M. J. Johansson, D. J. Gorin, S. T. Staben and F. D. Toste, *J. Am. Chem. Soc.,* 2005, **127**, 18002.
[4] A. Z. González, D. Benitez, E. Tkatchouk, W. A Goddard and F. D. Toste, *J. Am. Chem. Soc.* 2011, **133**, 5500
[5] L. Wei, J. A. Mulder, C. A. Zificsak, C. J. Douglas and R. P. Hsung, *Tetrahedron*. 2001, **57**, 459.
[6] A. González-Gómez, G. Domínguez and J. Pérez-Castells, *Eur. J. Org. Chem.,* 2009, 5057
[7] D. H. T. Phan, K. G. M. Kou and V. M. Dong, *J. Am. Chem. Soc.,* 2010, **132**, 16354.
[8] L. T. Ball, G. C. Lloyd-Jones and C. A. Russell, *Chem.-Eur. J.*, 2012, **18**, 2931.
[9] F. Gao and A. H. Hoveyda, *J. Am. Chem. Soc.,* 2010, **132**, 10961.
[10] E. Negishi, D. E. Van Horn and T. Yoshida, *J. Am. Chem. Soc.*, 1985, **107**, 6639.
[11] L. F. Silva, F. A. Siqueira, E. C. Pedrozo, F. Y. M. Vieira and A. C. Doriguetto, *Org. Lett.,* 2007, **9**, 1433.
[12] H. Banks and H. Ziffer, *J. Org. Chem.*, 1982, **47**, 3743.

Representative procedure for the multicomponent [2 + 2 + 2] cycloaddition. (Exemplified for the reaction between allenamide **1a**, *α*-methylstyrene (**2b**) and benzaldehyde (**3a**)).

A solution of 3-(propa-1,2-dienyl)oxazolidin-2-one (**1a**, 20.0 mg, 0.160 mmol) in CH_2Cl_2 (0.5 mL) was added to solution of *α*-methylstyrene (41.6 µl, 0.320 mmol)), benzaldehyde (162 µl, 1.60 mmol) and **Au3** (3.9 mg, 3.2 µmol) in CH_2Cl_2 (1.5 mL) under Argon atmosphere, in a dried Schlenk tube with 200 mg of powder MS, at -78 °C. The mixture was stirred at that temperature for 1.2 h (the progress of the reaction was monitored by *tlc*) and filtered through a short pad of florisil, eluting with EtOAc. The solvent was removed and the crude residue was dissolved in 0.6 mL of a 1,3,5-trimethoxybenzene 0.0887 M solution in $CDCl_3$ for ^{1}H-NMR analysis, which showed a 98% yield and a **4aba** / **4aba'** ratio = 3.5 : 1. The crude mixture was then purified on column chromatography (hexanes/EtOAc, 10-40%) to afford 50.1 mg of **4aba** and **4aba'** (0.14 mmol, 90% yield).[13]

3-((Z)-((2*S,6*S**)-6-methyl-2,6-diphenyldihydro-2H-pyran-3(4*H*)-ylidene)methyl)oxazolidin-2-one (4aba).**[14]

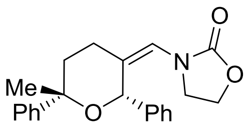

4aba

Major isomer. White solid. **^{1}H NMR** (500 MHz, $CDCl_3$) δ 7.51 – 7.46 (m, 4H), 7.37 – 7.32 (m, 3H), 7.31 – 7.27 (m, 2H), 7.19 (t, *J* = 7.7 Hz, 1H), 5.78 – 5.75 (m, 1H), 5.60 (s, 1H), 3.96 (td, *J* = 8.8, 5.3 Hz, 1H), 3.59 (q, *J* = 8.7 Hz, 1H), 3.31 (q, *J* = 8.6 Hz, 1H), 2.78 (td, *J* = 8.8, 5.3 Hz, 1H), 2.58 – 2.49 (m, 1H), 2.38 (dt, *J* = 14.3, 4.7 Hz, 1H), 2.25 (dt, *J* = 13.3, 5.0 Hz, 1H), 2.05 (ddd, *J* = 13.3, 11.5, 4.1 Hz, 1H), 1.67 (s, 3H). **^{13}C NMR** (75 MHz, $CDCl_3$) δ 155.7 (C), 148.7 (C), 141.2 (C), 139.0 (C), 128.2 (CH), 128.0 (CH), 127.9 (CH), 126.3 (CH), 124.7 (CH), 116.7 (CH), 75.7 (C), 73.0 (CH), 61.7 (CH_2), 45.3 (CH_2), 37.1 (CH_2), 27.1 (CH_3), 25.9 (CH_2). **LRMS** (*m/z, ESI*): 372.16 $(M+Na)^+$, 332.16, 263.14, 245.13, 143.11, 117.11. **HRMS** Calculated for $C_{22}H_{23}NNaO_3$: 372.1570, found 372.1560.

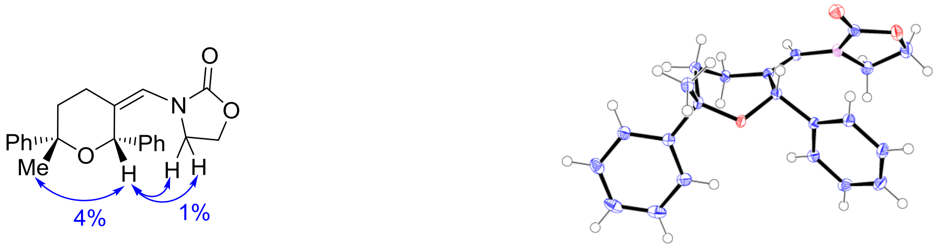

Figure S1. Significant nOe's and X-ray structure of **4aba**.[15]

3-((*Z*)-((2*R,6*S**)-6-Methyl-2,6-diphenyldihydro-2H-pyran-3(4*H*)-ylidene)methyl)oxazolidin-2-one (4aba')**.[16]

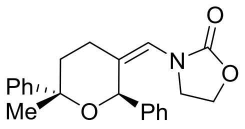

4aba'

Minor isomer. White solid. **^{1}H NMR** (300 MHz, $CDCl_3$) δ 7.52 – 7.40 (m, 4H), 7.38 – 7.27 (m, 5H), 7.22 – 7.15 (m, 1H), 5.81 (q, *J* = 1.8 Hz, 1H), 5.15 (t, *J* = 1.6 Hz, 1H), 3.79 (ddd, *J* = 9.2, 8.4, 5.9 Hz, 1H), 3.55 – 3.46 (m, 1H), 3.20 (ddd, *J* = 9.2, 8.3, 7.6 Hz, 1H), 2.86 – 2.72 (m, 1H), 2.72 – 2.60 (m, 1H), 2.47 – 2.41 (m, 1H), 2.31 – 2.27 (m, 2H), 1.43 (s, 3H). **^{13}C NMR** (75 MHz, $CDCl_3$) δ 155.8 (C), 146.7 (C), 140.7 (C), 137.0 (C), 128.4 (CH), 128.3 (CH), 128.0 (CH), 127.9 (CH), 126.7 (CH), 125.3 (CH), 116.9 (CH), 76.7 (C), 73.9 (CH), 61.7 (CH_2), 45.3 (CH_2), 34.6 (CH_2), 32.7 (CH_3), 26.3 (CH_2). **LRMS** (*m/z, ESI*): 372.16 $(M+Na)^+$, 332.16, 263.14, 245.13, 143.11, 117.11. **HRMS** Calculated for $C_{22}H_{23}NNaO_3$: 372.1570, found 372.1560.

[13] When the reaction was carried out with an allenamide (**1a**) / alkene (**2b**) / aldehyde (**3a**) molar ratio of 1.0 / 1.25 / 2.0, the products **4aba** and **4aba'** were isolated in 90% yield and a *dr* of 1.7:1 (Table 2, main manuscript, results under footnote *b*).

[14] We could easily separate by flash chromatography an almost pure fraction of **4aba** (**4aba**:**4aba'** ratio = 15:1) for full characterization.

[15] CCDC1038447 contains the crystallographic data for **4aba** that can be obtained free of charge from the CCDC via www.ccdc.cam.ac.uk/data_request/cif.

[16] NMR data of the minor isomer **4aba'** was deduced from a 1:1 mixture of **4aba** and **4aba'**.

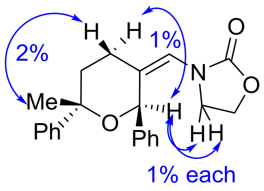

Figure S2. Significant nOe's observed for **4aba'**

3-((*Z*)-((2*S,5*R**,6*S**)-5-Methyl-2,6-diphenyldihydro-2H-pyran-3(4*H*)-ylidene)methyl)oxazolidin-2-one** (**4aaa**)

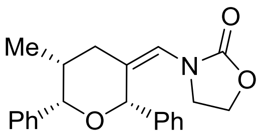

4aaa

35% yield, white solid. Reaction time: 1 h. ^{1}**H NMR** (500 MHz, $CDCl_3$) δ 7.54 – 7.45 (m, 2H), 7.38 – 7.27 (m, 3H), 7.30 – 7.21 (m, 4H), 7.22 – 7.14 (m, 1H), 5.87 (d, *J* = 1.3 Hz, 1H), 5.41 (s, 1H), 4.87 (d, *J* = 3.7 Hz, 1H), 3.96 – 3.87 (m, 1H), 3.58 (q, *J* = 8.3 Hz, 1H), 3.37 – 3.30 (m, 1H), 3.07 (td, *J* = 8.8, 5.2 Hz, 1H), 2.79 (ddd, *J* = 14.1, 6.0, 1.4 Hz, 1H), 2.33 (dd, *J* = 14.1, 5.0 Hz, 1H), 2.30 – 2.19 (m, 1H), 0.71 (d, *J* = 6.8 Hz, 3H). ^{13}**C NMR** (75 MHz, $CDCl_3$) δ 156.3 (C), 140.9 (C), 140.6 (C), 133.3 (C), 128.0 (CH), 127.9 (CH), 127.9 (CH), 127.4 (CH), 126.6 (CH), 125.6 (CH), 119.0 (CH), 79.5 (CH), 61.8 (CH_2), 45.8 (CH_2), 37.9 (CH_2), 34.9 (CH), 14.0 (CH_3). **LRMS** (*m/z, ESI*): 372.16 $(M+Na)^+$, 332.16, 282.28, 263.14, 245.13, 117.07, 91.06. **HRMS** Calculated for $C_{22}H_{23}NNaO_3$: 372.1570, found 372.1564.

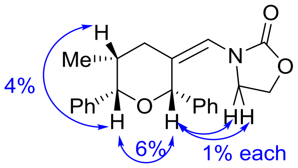

Figure S3. Significant nOe's observed for **4aaa**.

3-((*Z*)-(-3-Methyl-2-phenylcyclobutylidene)methyl)oxazolidin-2-one (**5aa**).[17]

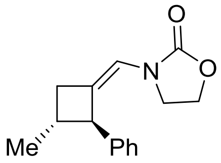

37% yield. ^{1}**H NMR** (300 MHz, $CDCl_3$) δ 7.38 – 7.17 (m, 5H), 6.40 (t, *J* = 2.0 Hz, 1H), 4.17 – 4.01 (m, 1H), 3.91 (qd, *J* = 8.5, 0.9 Hz, 1H), 3.78 – 3.67 (m, 1H), 3.56 – 3.38 (m, 1H), 3.15 (td, *J* = 9.0, 6.0 Hz, 1H), 3.05 – 2.88 (m, 1H), 2.41 – 2.24 (m, 2H), 1.26 (d, *J* = 6.2 Hz, 3H). ^{13}**C NMR** (75 MHz, $CDCl_3$) δ 156.2 (C), 143.6 (C), 128.7 (CH), 126.8 (CH), 126.5 (CH), 123.0 (C), 117.4 (CH), 62.0 (CH_2), 55.0 (CH), 44.3 (CH_2), 38.0 (CH), 34.0 (CH_2), 20.9 (CH_3). **LRMS** (*m/z, CI*): 244 [M^+ +1, 6], 195 (5), 157 (8), 135 (100), 126 (75). **HRMS** calculated for $C_{15}H_{18}NO_2$: 244.1338, found 244.1337.

3-((*Z*)-((2S*,6S*)-2,6-Diphenyldihydro-2H-pyran-3(4H)-ylidene)methyl)oxazolidin-2-one (**4aca**)

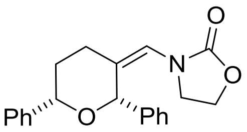

4aca

37% yield (*dr* = 1:0). White solid. Reaction time: 3 h.[18] ^{1}**H NMR** (500 MHz, $CDCl_3$) δ 7.55 – 7.50 (m, 2H), 7.43 – 7.31 (m, 7H), 7.29 – 7.24 (m, 1H), 6.00 (q, *J* = 1.5 Hz, 1H), 5.52 (s, 1H), 4.72 (dd, *J* = 10.3, 4.5 Hz, 1H), 4.00 (ddd, *J* = 9.2, 8.4, 5.4 Hz, 1H), 3.76 – 3.69 (m, 1H), 3.44 – 3.35 (m, 1H), 3.13 (ddd, *J* = 9.1, 8.4, 5.4 Hz, 1H), 2.75 – 2.67 (m, 1H), 2.58 (dtd, *J* = 14.2, 7.0, 1.4 Hz, 1H), 2.22 – 2.12 (m, 1H), 2.06 – 1.94 (m, 1H). ^{13}**C NMR** (75 MHz, $CDCl_3$) δ 156.27 (C), 142.91 (C), 140.53 (C), 133.78 (C), 128.27 (CH), 128.15 (CH), 127.93 (CH), 127.47 (CH), 127.28 (CH), 125.71 (CH), 118.58 (CH), 78.94 (CH), 76.96 (CH), 61.87 (CH_2), 45.79

---

[17] Previously described in H. Faustino, P. Bernal, L. Castedo, F. López and J. L. Mascareñas, *Adv. Synth. Catal.,* 2012, **354**, 165. (CCDC 863034).

[18] The [2+2] adduct **5ac** was also isolated in 45% yield. This product was previously described H. Faustino, P. Bernal, L. Castedo, F. López and J. L. Mascareñas, *Adv. Synth. Catal.,* 2012, **354**, 1658.

(CH_2), 33.41 (CH_2), 28.49 (CH_2). **LRMS** (*m/z, ESI*): 358.1424 $(M+Na)^+$, 249.1273, 145.0655, 117.0714. **HRMS** Calculated for $C_{21}H_{21}NNaO_3$: 358.15, found 358.1414.

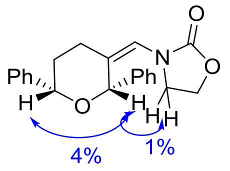

Figure S4. Significant nOe's observed for **4aca**.

3-((*Z*)-((2S*,6S*)-6-(2-Methoxyphenyl)-2-phenyldihydro-2H-pyran-3(4H)-ylidene)methyl)oxazolidin-2-one (4ada)

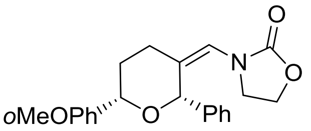

4ada

60% yield (*dr* = 1:0). Reaction time: 2 h.[19] **^{1}H NMR** 7.50 (dt, *J* = 7.7, 2.2 Hz, 3H), 7.41 – 7.29 (m, 3H), 7.24 – 7.17 (m, 1H), 6.94 (t, *J* = 7.5 Hz, 1H), 6.84 (d, *J* = 8.2 Hz, 1H), 6.02 (d, *J* = 1.5 Hz, 1H), 5.50 (s, 1H), 5.03 (dd, *J* = 9.9, 4.7 Hz, 1H), 3.97 (td, *J* = 8.8, 5.7 Hz, 1H), 3.83 (s, 3H), 3.74 (q, *J* = 8.9, 8.4 Hz, 1H), 3.38 (q, *J* = 8.7 Hz, 1H), 3.14 (td, *J* = 8.7, 5.7 Hz, 1H), 2.73 – 2.59 (m, 1H), 2.59 – 2.44 (m, 1H), 2.31 – 2.15 (m, 1H), 1.87 – 1.70 (m, 1H). **^{13}C NMR** (75 MHz, $CDCl_3$) δ 156.49 (C), 155.62 (C), 140.79 (C), 133.86 (C), 131.78 (C), 128.21 (CH), 127.94 (CH), 127.56 (CH), 126.05 (CH), 120.67 (CH), 118.33 (CH), 110.09 (CH), 78.83 (CH), 71.50 (CH), 61.82 (CH_2), 55.22 (CH_3), 45.65 (CH_2), 32.17 (CH_2), 28.32 (CH_2). **LRMS** (*m/z, ESI*): 388.15 $(M+Na)^+$, 298.08, 279.14, 261.13, 214.09, 129.07 . **HRMS** Calculated for $C_{22}H_{23}NNaO_4$: 388.1519, found 388.1534.

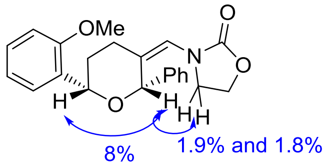

Figure S5. Significant nOe's observed for **4ada**

(*Z*)-3-((2-(2-Methoxyphenyl)cyclobutylidene)methyl)oxazolidin-2-one (5ad)

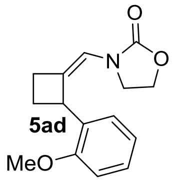

21% yield, white solid. **^{1}H NMR** (300 MHz, $CDCl_3$) δ 7.34 (dd, *J* = 7.4, 1.8 Hz, 1H), 7.22 (td, *J* = 7.8, 1.7 Hz, 1H), 6.95 (td, *J* = 7.4, 1.0 Hz, 1H), 6.85 (dd, *J* = 8.2, 1.0 Hz, 1H), 6.45 (q, *J* = 2.2 Hz, 1H), 4.65 – 4.54 (m, 1H), 4.21 – 4.12 (m, 1H), 4.10 – 3.95 (m, 1H), 3.82 (s, 3H), 3.59 (td, *J* = 9.2, 7.5 Hz, 1H), 3.34 (td, *J* = 9.1, 6.0 Hz, 1H), 2.94 – 2.77 (m, 1H), 2.76 – 2.50 (m, 2H), 1.87 – 1.72 (m, 1H). **^{13}C NMR** (75 MHz, $CDCl_3$) 156.32 (C), 156.25 (C), 132.75 (C), 127.70 (CH), 127.50 (CH), 124.39 (C), 120.86 (CH), 117.20 (CH), 110.35 (CH), 62.06 (CH_2), 55.28 (CH_3), 43.51 (CH_2), 40.70 (CH), 27.30 (CH_2), 26.34 (CH_2). **LRMS** (*m/z, ESI*): 282.11 $(M+Na)^+$, 220.05, 173.09, 158.07, 126.05, 105.04. **HRMS** Calculated for $C_{15}H_{17}NNaO_3$: 282.1101, found 282.1103.

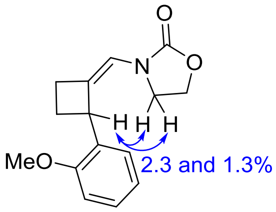

Figure S6. Significant nOe's observed for **5ad**

[19] The [2+2] adduct **5ad** was also isolated in 21% yield.

3-((*Z*)-((2S*,6S*)-6-(4-Methoxyphenyl)-2-phenyldihydro-2H-pyran-3(4H)-ylidene)methyl)oxazolidin-2-one (**4aea**)

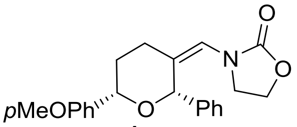

4aea

65% yield (*dr* = 1 :0). White solid. Reaction time: 0.5 h.[20] **^{1}H NMR** (500 MHz, $CDCl_3$) δ 7.51 – 7.44 (m, 2H), 7.36 – 7.27 (m, 5H), 6.88 – 6.80 (m, 2H), 5.96 (d, *J* = 1.3 Hz, 1H), 5.48 (s, 1H), 4.64 (dd, *J* = 10.3, 4.5 Hz, 1H), 3.98 – 3.91 (m, 1H), 3.77 (s, 3H), 3.70 (q, *J* = 8.5 Hz, 1H), 3.35 (q, *J* = 8.6 Hz, 1H), 3.09 (td, *J* = 8.8, 5.5 Hz, 1H), 2.72 – 2.64 (m, 1H), 2.58 – 2.50 (m, 1H), 2.15 – 2.06 (m, 1H), 2.02 – 1.94 (m, 1H). **^{13}C NMR** (75 MHz, $CDCl_3$) δ 158.8 (C), 156.2 (C), 140.5 (C), 135.1 (C), 133.8 (C), 128.1 (CH), 127.8 (CH), 127.4 (CH), 127.0 (CH), 118.5 (CH), 113.6 (CH), 78.9 (CH), 76.6 (CH), 61.8 (CH_2), 55.2 (CH_3), 45.7 (CH_2), 33.2 (CH_2), 28.5 (CH_2). **LRMS** (*m/z, ESI*): 388.15 $(M+Na)^+$, 348.16, 278.14, 261.13, 214.09, 145.07, 117.07. **HRMS** Calculated for $C_{22}H_{23}NNaO_4$: 388.1519, found 388.1521.

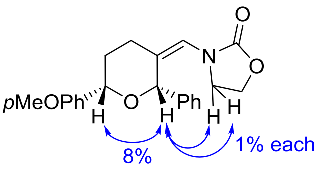

Figure S7. Significant nOe's observed for **4aea**

(*Z*)-3-((2-(4-Methoxyphenyl)cyclobutylidene)methyl)oxazolidin-2-one (5ae)

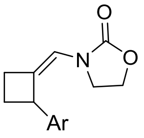

5ae
Ar = *p*MeOPh

<3% yield, white solid. **^{1}H NMR** (500 MHz, $CDCl_3$) δ 7.18 (d, *J* = 8.5 Hz, 2H), 6.85 (d, *J* = 8.5 Hz, 2H), 6.36 (q, *J* = 2.2 Hz, 1H), 4.24 (ddt, *J* = 9.1, 5.9, 2.9 Hz, 1H), 4.16 – 4.08 (m, 1H), 3.99 (q, *J* = 8.6 Hz, 1H), 3.80 (s, 3H), 3.51 (q, *J* = 9.0 Hz, 1H), 3.28 – 3.18 (m, 1H), 2.89 – 2.78 (m, 1H), 2.78 – 2.68 (m, 1H), 2.59 – 2.49 (m, 1H), 1.93 – 1.83 (m, 1H). **^{13}C NMR** (75 MHz, $CDCl_3$) δ 158.2 (C), 156.1 (C), 136.8 (C), 127.9 (CH), 126.0 (C), 116.9 (CH), 114.1 (CH), 62.0 (CH_2), 55.2 (CH_3), 46.1 (CH), 44.2 (CH_2), 28.7 (CH_2), 26.4 (CH_2). **LRMS** (*m/z, ESI*): 282.11$(M+Na)^+$, 220.05, 152.07, 126.06. **HRMS** Calculated for $C_{15}H_{17}NNaO_3$: 282.1101, found 282.1110.

3-((Z)-((2S*,4R*)-2,4-Bis(4-methoxyphenyl)cyclohexylidene)methyl)oxazolidin-2-one (**8ae**)

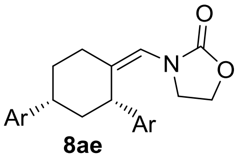

8ae
Ar = *p*MeOPh

5% yield. White solid. **^{1}H NMR** (500 MHz, $CDCl_3$) δ 7.21 (d, *J* = 8.5 Hz, 2H), 7.14 (d, *J* = 8.6 Hz, 2H), 6.86 – 6.79 (m, 4H), 5.82 (s, 1H), 3.78 (s, 6H), 3.74 – 3.63 (m, 2H), 3.66 – 3.59 (m, 1H), 3.14 (h, *J* = 8.3 Hz, 2H), 2.85 – 2.74 (m, 1H), 2.58 (dt, *J* = 14.3, 5.0 Hz, 1H), 2.45 – 2.31 (m, 1H), 2.14 – 2.01 (m, 2H), 1.90 (q, *J* = 12.5 Hz, 1H), 1.71 (ddt, *J* = 17.0, 12.2, 5.4 Hz, 1H). **^{13}C NMR** (75 MHz, $CDCl_3$) δ 158.1 (C), 157.9 (C), 156.6 (C), 140.4 (C), 138.4 (C), 136.2 (C), 128.3 (CH), 127.6 (CH), 117.6 (CH), 113.8 (CH), 113.3 (CH), 61.9 (CH_2), 55.3 (CH_3), 55.2 (CH_3), 46.4 CH_2), 45.5 (CH), 42.3 (CH_2), 42.1 (CH), 33.8 (CH_2), 33.4 (CH_2). **LRMS** (*m/z, ESI*): 394.20 $(M+H)^+$, 307.17, 286.15, 199.11, 178.09.**HRMS** Calculated for $C_{24}H_{28}NO_4$: 394.2013, found 394.2028.

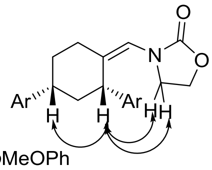

Ar = *p*MeOPh

Figure S8. Significant NOESY cross peaks observed for **8ae** (Ar = *p*MeOC_6H_4)**.**

[20] The reaction also provided a 5% yield of the [2C+2C+2C] cycloadduct **8ae** and traces of the [2+2] adduct **5ae**.

(*Z*)-3-((2,6,6-Triphenyldihydro-2H-pyran-3(4*H*)-ylidene)methyl)oxazolidin-2-one (**4afa**)

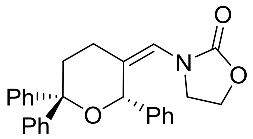

4afa

86% yield. White solid.[21] **^{1}H NMR** (300 MHz, $CDCl_3$) δ 7.61 – 7.45 (m, 4H), 7.47 – 7.29 (m, 7H), 7.33 – 7.16 (m, 3H), 7.20 – 7.07 (m, 1H), 5.78 (d, *J* = 1.5 Hz, 1H), 5.24 (s, 1H), 3.88 – 3.69 (m, 1H), 3.48 (q, *J* = 8.2 Hz, 1H), 3.19 (q, *J* = 8.0 Hz, 1H), 2.88 – 2.65 (m, 2H), 2.69 – 2.42 (m, 3H). **^{13}C NMR** (75 MHz, $CDCl_3$) δ 156.0 (C), 148.1 (C), 144.1 (C), 140.6 (C), 136.9 (C), 128.4 (CH), 128.1 (CH), 127.9 (CH), 127.8 (CH), 127.8 (CH), 127.1 (CH), 127.0 (CH), 126.3 (CH), 125.2 (CH), 117.2 (CH), 80.0 (C), 73.8 (CH), 61.7 (CH_2), 45.4 (CH_2), 35.5 (CH_2), 26.5 (CH_2). **LRMS** (*m/z, ESI*): 434.17 $(M+Na)^+$, 394.18, 325.26, 241.09, 193.10, 145.07, 117.07. **HRMS** Calculated for $C_{27}H_{25}NNaO_3$: 434.1727, found 434.1721.

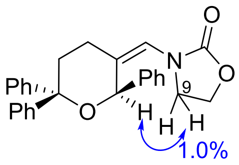

Figure S9. Significant nOe observed for **4afa**.

(*E*)-3-(5,5-Diphenylpenta-1,4-dien-1-yl)oxazolidin-2-one (**7af**)

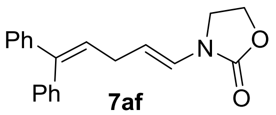

5% yield. **^{1}H NMR** (300 MHz, $CDCl_3$) δ 7.45 – 7.17 (m, 10H), 6.70 (dt, *J* = 14.3, 1.5 Hz, 1H), 6.08 (t, *J* = 7.6 Hz, 1H), 4.85 (dt, *J* = 14.3, 6.7 Hz, 1H), 4.46 – 4.37 (m, 2H), 3.72 – 3.65 (m, 2H), 2.94 – 2.86 (m, 2H). **^{13}C NMR** (75 MHz, $CDCl_3$) δ 155.3 (C), 142.6 (C), 142.2 (C), 139.5 (C), 129.6 (CH), 128.2 (CH), 128.0 (CH), 127.2 (CH), 127.1 (CH), 127.0 (CH), 126.6 (CH), 124.4 (CH), 109.2 (CH), 62.0 (CH_2), 42.5 (CH_2), 30.1 (CH_2). **LRMS** (*m/z, CI*): 306 [M^+ +1, 64], 235 (42), 209 (72), 183 (78), 116 (100). **HRMS** [M^+ +1], Calculated for $C_{20}H_{20}NO_2$: 306.1494, found 306.1482.

3-((Z)-((1R*,6'S*)-6'-Phenyl-3,3',4,4'-tetrahydro-2H-spiro[naphthalene-1,2'-pyran]-5'(6'H)-ylidene)methyl) oxazolidin-2-one (**4aga**).[22]

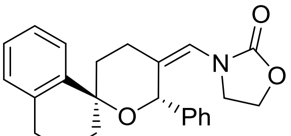

4aga

Major isomer, 55% yield. White solid. **^{1}H NMR** (500 MHz, $CDCl_3$) δ 7.69 (d, *J* = 7.9 Hz, 1H), 7.44 (d, *J* = 7.1 Hz, 2H), 7.34 (t, *J* = 7.3 Hz, 2H), 7.31 – 7.27 (m, 1H), 7.25 (t, *J* = 7.5 Hz, 1H), 7.19 (td, *J* = 7.4, 1.5 Hz, 1H), 7.08 (d, *J* = 7.6 Hz, 1H), 6.52 (s, 1H), 5.75 (s, 1H), 4.18 – 4.06 (m, 2H), 3.44 (h, *J* = 8.6 Hz, 2H), 2.84 – 2.65 (m, 3H), 2.45 (dt, *J* = 14.1, 4.0 Hz, 1H), 2.23 (td, *J* = 13.4, 4.6 Hz, 1H), 2.01 (dt, *J* = 13.6, 4.2 Hz, 1H), 1.87 – 1.69 (m, 2H), 1.66 – 1.60 (m, 1H), 1.58 – 1.48 (m, 1H). **^{13}C NMR** (75 MHz, $CDCl_3$) δ 157.01 (C), 141.68 (C), 141.13 (C), 137.43 (C), 128.99 (CH), 128.35 (CH), 127.77 (CH), 127.70 (CH), 127.47 (CH), 127.18 (CH), 125.81 (CH), 119.27 (CH), 74.67 (C), 72.45 (CH), 62.01 (CH_2), 45.23 (CH_2), 36.52 (CH_2), 35.40 (CH_2), 29.45 (CH_2), 26.09 (CH_2), 19.49 (CH_2). **LRMS** (*m/z, ESI*): 398.17 $(M+Na)^+$, 308.10, 271.15, 234.95, 193.10, 167.09, 141.07. **HRMS** Calculated for $C_{24}H_{25}NNaO_3$: 398.1727, found 398.1745.

---

[21] (a) Carried out at -78ºC. The reaction also provided a 5% yield of **7af**. (b) When the reaction was carried out with an allenamide (**1a**) / alkene (**2f**) / aldehyde (**3a**) molar ratio of 1.0 / 1.25 / 2.0, at -45ºC (Table 2, footnote *b*), **4afa** was isolated in 70% yield. In this case a 15% yield of **7af** was also obtained.

[22] (a) The cycloaddition of **1a**, **2g** and **3a** provided, after 10 min at -45 ºC, the adducts **4aga** and **4aga'** in a 1.5:1 diastereoisomeric ratio (measured by ^{1}H NMR in the crude mixture). The isomers could be easily separated by chromatography to yield **4aga** (55% yield) and **4aga'** (39% yield), a global 94% yield. (b) When the reaction was carried out with an allenamide (**1a**) / alkene (**2g**) / aldehyde (**3a**) molar ratio of 1.0 / 1.25 / 2.0, at -45 ºC (Table 2, footnote *b*), **4aga** and **4aga'** were isolated in 75% yield (*dr* 1.5: 1). A 8% yield of **5ag** was also obtained.

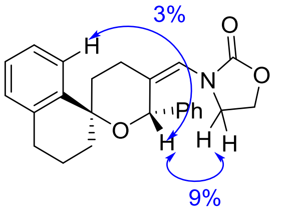

Figure S10. Significant nOe's observed for **4aga**.

3-((Z)-((1S*,6'S*)-6'-Phenyl-3,3',4,4'-tetrahydro-2H-spiro[naphthalene-1,2'-pyran]-5'(6'H)-ylidene)methyl) oxazolidin-2-one (**4aga'**).[22]

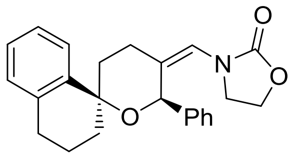

Minor isomer, 39% yield. White solid. **^{1}H NMR** (500 MHz, $CDCl_3$) δ 7.67 (d, *J* = 7.8 Hz, 1H), 7.48 (d, *J* = 6.8 Hz, 2H), 7.34 (t, *J* = 7.3 Hz, 2H), 7.32 – 7.25 (m, 1H), 7.19 (t, *J* = 7.5 Hz, 1H), 7.13 (td, *J* = 7.4, 1.4 Hz, 1H), 7.04 (d, *J* = 7.6 Hz, 1H), 5.83 (s, 1H), 5.62 (s, 1H), 4.00 (td, *J* = 9.0, 5.4 Hz, 1H), 3.70 (q, *J* = 8.4 Hz, 1H), 3.35 (q, *J* = 8.3 Hz, 1H), 2.93 – 2.74 (m, 4H), 2.51 (dt, *J* = 14.1, 5.0 Hz, 1H), 2.37 (ddd, *J* = 12.7, 6.6, 2.8 Hz, 1H), 2.30 (dt, *J* = 13.5, 5.3 Hz, 1H), 2.13 – 2.04 (m, 1H), 2.05 – 1.98 (m, 1H), 1.94 (ddd, *J* = 13.5, 11.0, 4.9 Hz, 1H), 1.86 – 1.75 (m, 1H). ^{13}C NMR (75 MHz, $CDCl_3$) δ 155.90 (C), 143.28 (C), 141.38 (C), 138.32 (C), 135.95 (C), 128.70 (CH), 128.32 (CH), 127.98 (CH), 127.92 (CH), 126.87 (CH), 126.23 (CH), 126.17 (CH), 116.94 (CH), 74.20 (C), 72.84 (CH), 61.73 (CH_2), 45.31 (CH_2), 37.31 (CH_2), 33.73 (CH_2), 29.18 (CH_2), 26.66 (CH_2), 19.29 (CH_2). **LRMS** (*m/z, ESI*): 398.17 $(M+Na)^+$, 308.10, 271.15, 214.09, 193.10, 141.07. **HRMS** Calculated for $C_{24}H_{25}NNaO_3$: 398.1727, found 398.1742.

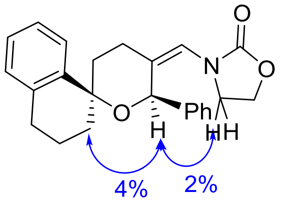

Figure S11. Significant nOe's observed for **4aga'**.

3-(((2S*,4aR*,8aR*,Z)-2,8a-Diphenylhexahydro-2H-chromen-3(4H)-ylidene)methyl)oxazolidin-2-one (**4aha**).[23]

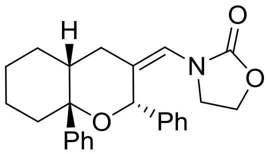

4aha

Major isomer. White solid. **^{1}H NMR** (500 MHz, $CDCl_3$) δ 7.58 – 7.51 (m, 2H), 7.50 – 7.44 (m, 2H), 7.44 – 7.34 (m, 5H), 7.32 – 7.25 (m, 1H), 5.45 (t, *J* = 2.0 Hz, 1H), 5.16 (s, 1H), 3.74 – 3.67 (m, 1H), 3.27 (q, *J* = 8.5 Hz, 1H), 3.15 (q, *J* = 8.5 Hz, 1H), 2.86 – 2.75 (m, 2H), 2.54 – 2.44 (m, 1H), 2.16 – 1.98 (m, 2H), 1.96 – 1.82 (m, 2H), 1.80 – 1.67 (m, 2H), 1.55 – 1.33 (m, 3H). **^{13}C NMR** (75 MHz, $CDCl_3$) δ 155.92 (C), 145.04 (C), 141.20 (C), 137.40 (C), 128.77 (CH), 127.84 (CH), 127.74 (CH), 126.74 (CH), 125.74 (CH), 117.77 (CH), 77.79 (C), 74.52 (CH), 61.56 (CH_2), 45.98 (CH_2), 43.19 (CH_2), 35.26 (CH), 34.60 (CH_2), 28.24 (CH_2), 25.67 (CH_2), 22.02 (CH_2). **LRMS** (*m/z, ESI*): 412.1886 $(M+Na)^+$, 303.1753, 214.0865, 145.0664, 117.0716. **HRMS** Calculated for $C_{25}H_{27}NNaO_3$: 412.19, found 412.1883.

---

[23] (a) The cycloaddition of **1a**, **2h** and **3a** provided after 3 h at -45 ºC the adducts **4aha** and **4aha'** in a 3:1 diastereoisomeric ratio (measured by ^{1}H NMR in the crude mixture) and a global 84% yield. The data of **4aha** and **4aha'** was obtained from pure samples of each isomer, obtained by column chromatography. However, a complete separation of both isomers was not possible. (b) When the reaction was carried out with an allenamide (**1a**) / alkene (**2h**) / benzaldehyde (**3a**) molar ratio of 1.0 / 1.25 / 2.0, at -45 ºC (Table 2, footnote *b*), the product **4aha** was isolated in a global 68% yield (*dr* = 3:1). In this case, a 20% yield of **5ah** was also obtained.

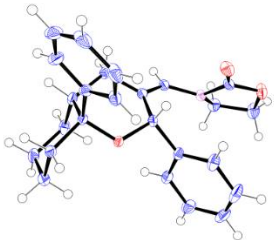

Figure S12. X Ray structure of **4aha.**[24]

3-(((2R*,4aR*,8aR*,Z)-2,8a-Diphenylhexahydro-2H-chromen-3(4H)-ylidene)methyl)oxazolidin-2-one (4aha').[23]

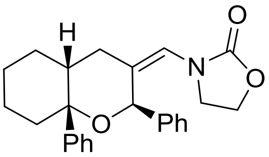

4aha'

Minor isomer. White solid. **^{1}H NMR** (500 MHz, $CDCl_3$) δ 7.15 – 7.02 (m, 7H), 6.93 – 6.85 (m, 3H), 6.25 (s, 1H), 5.88 (s, 1H), 4.14 – 4.00 (m, 2H), 3.49 – 3.40 (m, 1H), 3.16 (q, *J* = 8.5 Hz, 1H), 3.03 (d, *J* = 14.4 Hz, 1H), 2.52 (dq, *J* = 11.9, 4.1 Hz, 1H), 2.26 (dd, *J* = 14.5, 3.6 Hz, 1H), 2.01 – 1.57 (m, 5H), 1.54 – 1.43 (m, 1H), 1.43 – 1.10 (m, 2H). **^{13}C NMR** (75 MHz, $CDCl_3$) δ 156.87 (C), 145.49 (C), 141.95 (C), 128.18 (CH), 127.67(CH), 127.24 (CH), 126.86 (CH), 126.21 (CH), 125.57 (CH), 122.86 (C), 119.81 (CH), 76.38 (C), 73.72 (CH), 61.98 (CH_2), 45.78 (CH_2), 42.77 (CH_2), 35.02 (CH), 33.25 (CH_2), 27.84 (CH_2), 25.03 (CH_2), 22.09 (CH_2). **LRMS** (*m/z, ESI*): 412.1885 $(M+Na)^+$, 303.1744, 214.0858, 145.0661, 117.0712. **HRMS** Calculated for $C_{25}H_{27}NNaO_3$: 412.19, found 412.1885.

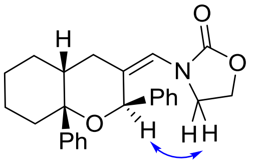

Figure S13. Significant NOESY cross peaks observed for **4aha'**.

3-((*Z*)-((2*S,4a*R**,10b*S**)-10b-Methyl-2-phenyl-4,4a,5,6-tetrahydro-2*H*-benzo[*h*]chromen-3(10b*H*)-ylidene) methyl)oxazolidin-2-one** (**4aia**)

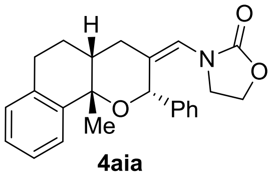

4aia

57% yield. *dr* = 1:0. Reaction time: 2h.[25] **^{1}H NMR** (500 MHz, $CDCl_3$) δ 7.55 (d, *J* = 7.4 Hz, 1H), 7.40 (d, *J* = 7.4 Hz, 2H), 7.34 – 7.24 (m, 3H), 7.17 – 7.07 (m, 2H), 7.04 (d, *J* = 7.1 Hz, 1H), 6.03 (s, 1H), 5.73 (s, 1H), 4.05 (q, *J* = 8.2, 7.8 Hz, 1H), 3.86 (q, *J* = 8.4 Hz, 1H), 3.38 (q, *J* = 8.5 Hz, 1H), 3.07 – 2.98 (m, 1H), 2.82 – 2.71 (m, 1H), 2.65 – 2.55 (m, 1H), 2.50 (t, *J* = 12.4 Hz, 1H), 2.42 (dd, *J* = 13.3, 4.9 Hz, 1H), 2.18 – 2.08 (m, 1H), 2.08 – 1.94 (m, 1H), 1.63 (s, 3H), 1.56 (dtd, *J* = 13.4, 9.4, 4.1 Hz, 1H). **^{13}C NMR** (75 MHz, $CDCl_3$) δ 156.1 (C), 141.9 (C), 140.8 (C), 136.2 (C), 135.3 (C), 128.3 (CH), 127.9 (CH), 127.8 (CH), 127.6 (CH), 126.4 (CH), 126.3 (CH), 116.7 (CH), 74.6 (C), 72.9 (CH), 61.9 (CH_2), 45.3 (CH_2), 42.6 (CH), 33.9 (CH_2), 30.3 (CH_3), 28.3 (CH_2), 27.8 (CH_2). **LRMS** (*m/z, ESI*): 398.17 $(M+Na)^+$, 358.18, 289.16, 214.09, 117.07. **HRMS** Calculated for $C_{24}H_{25}NNaO_3$: 398.1727, found 398.1719.

---

[24] CCDC 1038448 contains the crystallographic data for **4aha** that can be obtained from the CCDC via www.ccdc.cam.ac.uk/data_request/cif.

[25] (a) When the reaction was carried out with an allenamide (**1a**) / alkene (**2i**) / aldehyde (**3a**) molar ratio of 1.0 / 1.25 / 2.0, at -45 ºC (Table 2, footnote *b*), the product **4aia** was isolated in 48% yield (*dr* = 7:1). In this case, a 30% yield of **5ai** was also obtained. (b) CCDC 1038449 contains the crystallographic data for **4aia** that can be obtained from the CCDC via www.ccdc.cam.ac.uk/data_request/cif.

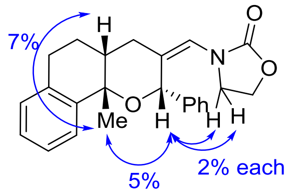

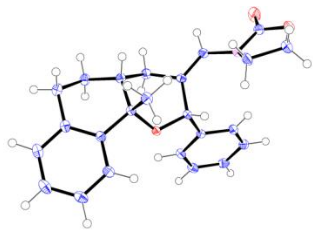

Figure S14. Significant nOe's observed for **4aia** and X-ray diffraction structure.[25b]

3-((Z)-((2S*,4aR*,9bS*)-9b-Methyl-2-phenyl-4,4a,5,9b-tetrahydroindeno[1,2-b]pyran-3(2H)-ylidene)methyl)oxazolidin-2-one (**4aja**)

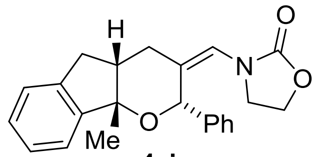

4aja

69% yield, *dr* = 1:0. White solid. Reaction time: 5h.[26] **^{1}H NMR** (500 MHz, $CDCl_3$) δ 7.43 – 7.19 (m, 9H), 5.96 (t, *J* = 1.9 Hz, 1H), 5.69 (s, 1H), 4.08 – 4.00 (m, 1H), 3.85 (q, *J* = 9.2, 8.4 Hz, 1H), 3.41 – 3.31 (m, 2H), 2.92 – 2.85 (m, 1H), 2.79 (dd, *J* = 17.2, 2.1 Hz, 1H), 2.59 – 2.44 (m, 3H), 1.70 (s, 3H). **^{13}C NMR** (75 MHz, $CDCl_3$) δ 155.85 (C), 146.75 (C), 140.86 (C), 140.56 (C), 136.69 (C), 128.34 (CH), 128.33 (CH), 128.02 (CH), 127.96 (CH), 126.96 (CH), 124.75 (CH), 124.38 (CH), 117.01 (CH), 85.04 (C), 73.21 (CH), 61.87 (CH_2), 45.23 (CH_2), 45.11 (CH), 37.92 (CH_2), 34.29 (CH_2), 25.24 (CH_3).). **LRMS** (*m/z, ESI*): 384.16 $(M+Na)^+$, 294.08, 257.13, 143.09, 129.07. **HRMS** Calculated for $C_{23}H_{23}NNaO_3$: 384.1570, found 384.1568

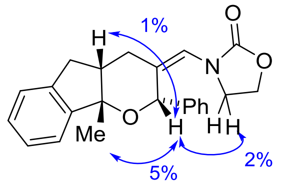

Figure S15. Significant nOe's observed for **4aja**.

3-((Z)-((2S*,6R*)-6-Methoxy-6-methyl-2-phenyldihydro-2H-pyran-3(4H)-ylidene)methyl)oxazolidin-2-one (**4aka**)

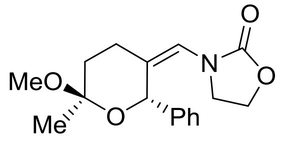

4aka

51% yield, *dr* = 1:0. Colorless oil. Reaction time 4 h at -78 ºC. **^{1}H NMR** (300 MHz, $CDCl_3$) δ 7.38 – 7.18 (m, 5H), 5.78 (q, *J* = 1.5 Hz, 1H), 5.25 (s, 1H), 3.81 – 3.72 (m, 1H), 3.60 – 3.45 (m, 1H), 3.26 – 3.15 (m, 4H), 2.92 – 2.77 (m, 1H), 2.44 – 2.31 (m, 2H), 2.03 – 1.91 (m, 1H), 1.84 – 1.67 (m, 1H), 1.30 (s, 3H). **^{13}C NMR** (75 MHz, $CDCl_3$) δ 155.81 (C), 140.23 (C), 136.21 (C), 128.28 (CH), 128.06 (CH), 128.03 (CH), 116.90 (CH), 99.38 (C), 72.14 (CH), 61.69 (CH_2), 48.71 (CH_3), 45.36 (CH_2), 36.09 (CH_2), 26.44 (CH_2), 23.59 (CH_3). **LRMS** (*m/z, CI*): 272.1 (M^+-OMe), 217.2, 203.2, 117.1.

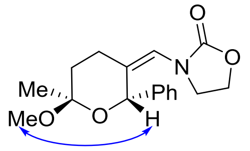

Figure S16. Significant NOESY cross peaks observed for **4aka**.

[26] (a) Carried out at -78ºC. The reaction also provided a 5% yield of **7aj**. (b) When the reaction was carried out with an allenamide (**1a**) / alkene (**2j**) / aldehyde (**3a**) molar ratio of 1.0 / 1.25 / 2.0, at -45 ºC (Table 2, footnote *b*) and 5% of catalyst (**Au3**), the product **4aja** was isolated in 45% yield. Additionally, a 15% yield of **7aj** was also observed.

(*Z*)-3-((6-Ethoxy-2-phenyldihydro-2*H*-pyran-3(4*H*)-ylidene)methyl)oxazolidin-2-one (**4ala** + **4ala'**; ratio 1.5 :1).[27]

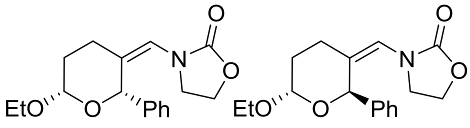

4ala 1.5 : 1 **4ala'**

94% global yield. Colorless oil. Reaction time 4h at -78ºC with 4% of **Au3**. **^{1}H NMR** (500 MHz, $CDCl_3$) δ 7.47 – 7.26 (m, 5.0H), 6.32 (s, 0.4H), 6.30 (s, 0.6H), 5.80 (s, 0.4H), 5.60 (s, 0.6H), 4.83 (t, *J* = 4.0 Hz, 0.6H), 4.70 (dd, *J* = 8.0, 3.8 Hz, 0.4H), 4.17 – 4.01 (m, 2H), 3.95 (dq, *J* = 9.5, 7.0 Hz, 0.4H), 3.59 – 3.45 (m, 1.2H), 3.45 – 3.41 (m, 0.6H), 3.41 – 3.34 (m, 1.2H), 3.29 (dq, *J* = 9.3, 7.0 Hz, 0.6H), 2.84 – 2.75 (m, 0.6H), 2.41 (dd, *J* = 6.3, 4.1 Hz, 0.8H), 2.33 (dt, *J* = 14.1, 4.9 Hz, 0.6H), 2.04 – 1.92 (m, 1.0H), 1.92 – 1.82 (m, 0.6H), 1.77 – 1.65 (m, 0.4H), 1.24 (t, *J* = 7.1 Hz, 1.20H), 0.75 (t, *J* = 7.1 Hz, 1.80H). **^{13}C NMR** (75 MHz, $CDCl_3$) δ 156.76 (C), 156.58 (C), 141.26 (C), 139.03 (C), 128.60 (CH), 128.07 (CH), 127.88 (CH), 127.52 (CH), 127.37 (CH), 127.16 (CH), 126.32 (C), 126.03 (C), 119.45 (CH), 119.39 (CH), 97.91 (CH), 96.49 (CH), 73.11 (CH), 72.27 (CH), 63.71 (CH_2), 63.23 (CH_2), 62.03 (CH_2), 61.94 (CH_2), 45.72 (CH_2), 45.40 (CH_2), 31.79 (CH_2), 31.14 (CH_2), 26.91 (CH_2), 24.76 (CH_2), 15.16 (CH_3), 14.36 (CH_3).

^{1}H NMR (500 MHz, **C_6D_6**) δ 7.44 – 7.38 (m, 2.0H), 7.22 (t, *J* = 7.6 Hz, 0.8H), 7.19 – 7.10 (m, 1.6H), 7.07 (t, *J* = 7.3 Hz, 0.60H), 6.41 (s, 1.0H), 5.76 (s, 0.4H), 5.58 (s, 0.6H), 4.77 (t, *J* = 3.5 Hz, 0.6H), 4.52 (dd, *J* = 8.3, 3.6 Hz, 0.4H), 4.06 – 3.96 (m, 0.4H), 3.49 – 3.38 (m, 0.6H), 3.41 – 3.29 (m, 0.4H), 3.18 – 3.08 (m, 0.6H), 3.08 – 2.96 (m, 1.4H), 2.95 – 2.86 (m, 0.6H), 2.76 – 2.66 (m, 0.6H), 2.54 – 2.42 (m, 1.4H), 2.45 – 2.37 (m, 0.6H), 2.14 – 2.03 (m, 0.4H), 1.98 – 1.88 (m, 1.0H), 1.82 – 1.71 (m, 1.6H), 1.74 – 1.64 (m, 0.4H), 1.17 (t, *J* = 7.0 Hz, 1.2H), 0.66 (t, *J* = 7.0 Hz, 1.8H).

LRMS (*m/z, ESI*): 326.14 $(M+Na)^+$, 258.11, 214.08, 171.08, 129.07, 88.04. **HRMS** Calculated for $C_{17}H_{21}NNaO_4$: 326.1363, found 326.1370.

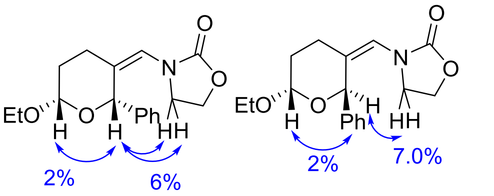

4ala **4ala'** (in C_6D_6)

Figure S17. Significant nOe's observed for **4ala** and **4ala'**

3-((*Z*)-((2S*,6S*)-6-(2-oxopyrrolidin-1-yl)-2-phenyldihydro-2H-pyran-3(4*H*)-ylidene)methyl)oxazolidin-2-one (**4ama**)

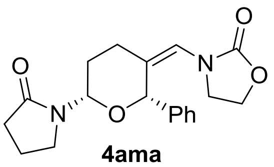

4ama

45% yield, *dr* = 1:0. Colorless oil. Reaction time: 6 h.[28] **^{1}H NMR** (500 MHz, $CDCl_3$) δ 7.42 – 7.37 (m, 2H), 7.36 – 7.26 (m, 3H), 6.07 (s, 1H), 5.57 (dd, *J* = 9.4, 4.8 Hz, 1H), 5.50 (s, 1H), 3.95 – 3.86 (m, 1H), 3.78 (q, *J* = 8.5 Hz, 1H), 3.47 (q, *J* = 8.1 Hz, 1H), 3.40 (q, *J* = 7.8 Hz, 1H), 3.32 (q, *J* = 8.2 Hz, 1H), 3.26 – 3.13 (m, 1H), 2.68 – 2.57 (m, 1H), 2.53 – 2.44 (m, 1H), 2.42 – 2.32 (m, 2H), 2.05 – 1.92 (m, 3H), 1.92 – 1.83 (m, 1H). **^{13}C NMR** (75 MHz, $CDCl_3$) δ 175.3 (C), 156.2 (C), 139.7 (C), 129.7 (C), 128.3 (CH), 128.2 (CH), 127.5 (CH), 119.1 (CH), 77.7 (CH), 77.2 (CH), 61.9 (CH_2), 45.4 (CH_2), 42.3 (CH_2), 31.4 (CH_2), 28.1 (CH_2),

[27] The isomers could not be separated by standard column chromatography. The NMR data was deduced from a 1.5 : 1 mixture of **4ala** : **4ala'**. (b) When the reaction was carried out with an allenamide (**1a**) / alkene (**2l**) / aldehyde (**3a**) molar ratio of 1.0 / 1.25 / 2.0, at -45 ºC (Table 2, footnote *b*), the product **4ala** was isolated in 60% yield (*dr* = 1.2 : 1).

[28] Reaction carried out from -45 ºC to -15 ºC, over 6 hours.

27.8 (CH_2), 17.9 (CH_2). **LRMS** (*m/z*, ESI): 365.15 $(M+Na)^+$, 256.13, 238.12, 171.08, 117.07, 98.06. **HRMS** Calculated for $C_{19}H_{22}N_2NaO_4$: 365.1472, found 365.1474.

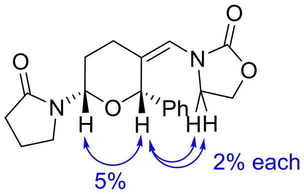

Figure S18. Significant nOe's observed for **4ama**.

3-((Z)-((2S*,4R*,6S*)-4,6-Dimethyl-2,6-diphenyldihydro-2H-pyran-3(4H)-ylidene)methyl)oxazolidin-2-one (4bba) [29]

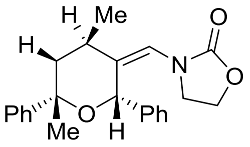

Major isomer. White solid. **^{1}H NMR** (500 MHz, $CDCl_3$) δ 7.54 – 7.44 (m, 4H), 7.40 – 7.32 (m, 2H), 7.35 – 7.27 (m, 3H), 7.24 – 7.16 (m, 1H), 5.67 (t, *J* = 1.9 Hz, 1H), 5.64 (t, *J* = 2.1 Hz, 1H), 4.02 (ddd, *J* = 9.5, 8.4, 5.3 Hz, 1H), 3.69 (q, *J* = 8.2 Hz, 1H), 3.37 (q, *J* = 8.2 Hz, 1H), 2.79 – 2.72 (m, 1H), 2.72 – 2.67 (m, 1H), 2.25 (dd, *J* = 13.2, 4.3 Hz, 1H), 1.80 (t, *J* = 13.0 Hz, 1H), 1.66 (s, 3H), 1.11 (d, *J* = 6.5 Hz, 3H). **^{13}C NMR** (126 MHz, $CDCl_3$) δ 155.47 (C), 149.17 (C), 145.09 (C), 141.4 (C)7, 128.29 (CH), 127.90 (CH), 126.25 (CH), 124.76 (CH), 115.12 (CH), 75.84 (C), 73.01 (CH), 61.71 (CH_2), 46.67 (CH_2), 45.42 (CH_2), 29.02 (CH), 28.11 (CH_3), 17.39 (CH_3). **LRMS** (*m/z, ESI*): 386.17 $(M+Na)^+$, 344.15, 277.16, 228.10, 159.08, 141.07, 131.08. **HRMS** Calculated for $C_{23}H_{25}NNaO_3$: 386.1727, found 386.1718.

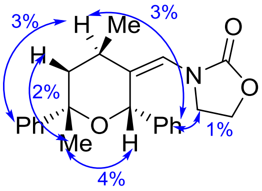

Figure S19. Significant nOe's observed for **4bba**

3-((Z)-((2R*,4R)-2,4-dimethyl-2-phenylcyclobutylidene)methyl)oxazolidin-2-one (5bb)

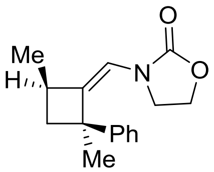

^{1}H NMR (500 MHz, $CDCl_3$) δ 7.39 – 7.36 (m, 2H), 7.32 (t, *J* = 7.7 Hz, 2H), 7.20 (t, *J* = 7.2 Hz, 1H), 6.34 (d, *J* = 2.4 Hz, 1H), 4.06 (td, *J* = 9.1, 5.5 Hz, 1H), 3.83 (q, *J* = 8.6 Hz, 1H), 3.25 (q, *J* = 8.0 Hz, 1H), 3.21 – 3.11 (m, 1H), 2.82 (td, *J* = 9.0, 5.5 Hz, 1H), 2.28 (dd, *J* = 10.8, 9.2 Hz, 1H), 1.81 (dd, *J* = 10.8, 8.3 Hz, 1H), 1.75 (s, 3H), 1.26 (d, *J* = 6.8 Hz, 3H). **^{13}C NMR** (75 MHz, $CDCl_3$) δ 156.54 (C), 147.74 (C), 135.81 (C), 128.62 (CH), 126.35 (CH), 125.99 (CH), 115.15 (CH), 61.81 (CH_2), 47.19 (C), 45.65 (CH_2), 45.08 (CH_2), 31.34 (CH), 26.12 (CH_3), 18.75 (CH_3).

[29] (a) The cycloaddition of **1b**, **2b** and **3a** provided after 2 h at -45 ºC the adducts **4bba** and **4bba'** in a 11:1 diastereoisomeric ratio (measured by ^{1}H NMR in the crude mixture) and a global 70% yield. The data of **4bba** was obtained from a pure sample, obtained by column chromatography. However, a complete separation of both isomers was not possible.The reaction also provided a 17% yield of the corresponding [2+2] adduct **5bb**.

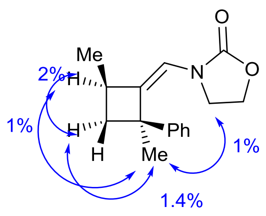

Figure S20. Significant nOe's observed for **5bb**

(*S)-4-Methyl-N-phenyl-N-((2,6,6-triphenyldihydro-2H-pyran-3(4H)-ylidene)methyl)benzenesulfonamide (4cfa).**[30]

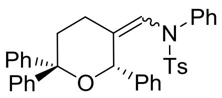

4cfa

77% yield, *E*:Z = 1:1. Colorless oil. Reaction time: 1 h at -78 ºC. **^{1}H NMR** (300 MHz, $CDCl_3$) δ 7.57 – 6.92 (m, 23H), 6.50 – 6.39 (m, 1H), 6.13 (s, 0.5H), 5.37 (t, *J* = 1.7 Hz, 0.5H), 5.08 (s, 1H), 2.88 – 2.53 (m, 4H), 2.39 (s, 1.5H), 2.36 (s, 1.5H). **^{13}C NMR** (126 MHz, $CDCl_3$) δ 148.19 (C), 148.16 (C), 145.13 (C), 143.70 (C), 143.65 (C), 142.08 (C), 141.35 (C), 140.08 (C), 139.68 (C), 139.43 (C), 139.37 (C), 139.01 (C), 134.28 (C), 133.95 (C), 129.30 (CH), 129.26 (CH), 128.94 (CH), 128.92 (CH), 128.82 (CH), 128.58 (CH), 128.28 (CH), 128.14 (CH), 127.98 (CH), 127.88 (CH), 127.80 (CH), 127.77 (CH), 127.56 (CH), 127.40 (CH), 127.21 (CH), 127.00 (CH), 126.93 (CH), 126.82 (CH), 126.76 (CH), 126.72 (CH), 126.67 (CH), 126.47 (CH), 126.32 (CH), 125.47 (CH), 125.03 (CH), 124.10 (CH), 120.72 (CH), 80.78 (C), 79.67 (C), 75.46 (CH), 73.82 (CH), 35.40 (CH_2), 35.18 (CH_2), 26.26 (CH_2), 23.86 (CH_2), 21.59 (CH_3), 21.55 (CH_3). **LRMS** (*m/z, CI*): 572.0 [M^+ +1], 417.0, 392.0, 325.0, 193.0, 135.0. **HRMS** [M^+ +1], Calculated for $C_{37}H_{34}NO_3S$ 572.2259, found 572.2266.

***N*-((*E*)-((2*S**,6*R**)-6-Methoxy-6-methyl-2-phenyldihydro-2H-pyran-3(4H)-ylidene)methyl)-4-methyl-N-phenylbenzenesulfonamide (*E*-4cka).**[31]

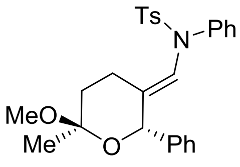

E-**4cka**

Major isomer. 55% yield. Colorless oil. **^{1}H NMR** (500 MHz, $CDCl_3$) δ 7.43 – 7.33 (m, 5H), 7.25 – 7.19 (m, 3H), 7.16 (d, *J* = 8.3 Hz, 2H), 7.11 – 7.07 (m, 2H), 6.97 – 6.93 (m, 2H), 5.30 (t, *J* = 1.8 Hz, 1H), 5.10 (s, 1H), 3.22 (d, *J* = 0.6 Hz, 3H), 2.51 (dt, *J* = 14.1, 4.4 Hz, 1H), 2.37 (s, 3H), 2.27 – 2.18 (m, 1H), 1.79 – 1.71 (m, 1H), 1.60 – 1.51 (m, 1H), 1.31 (s, 3H). **^{13}C NMR** (75 MHz, $CDCl_3$) δ 143.6 (C), 141.4 (C), 138.9 (C), 138.7 (C), 133.9 (C), 129.2 (CH), 128.8 (CH), 128.2 (CH), 128.1 (CH), 128.0 (CH), 127.7 (CH), 126.9 (CH), 126.8 (CH), 124.2 (CH), 98.7 (C), 73.9 (CH), 48.3 (CH_3), 35.4 (CH_2), 23.2 (CH_3), 22.0 (CH_2), 21.5 (CH_3).**LRMS** (*m/z, ESI*): 486.17 $(M+Na)^+$, 432.16, 326.12, 276.14, 218.10, 185.10, 119.06. **HRMS** Calculated for $C_{27}H_{29}NNaO_4S$: 486.1710, found 486.1706.

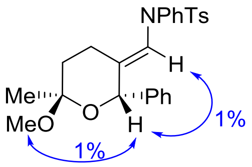

Figure S21. Significant nOe's observed for *E*-**4cka**.

---

[30] These *Z/E* isomers could not be separated by standard silica gel chromatography.

[31] The cycloaddition of **1c**, **2k** and **3a** provided after 10 min at -45 ºC the adducts *E*-**4cka** and *Z*-**4cka** in a 2:1 ratio. These isomers could be separated by column chromatography to yield *Z*-**4cka** (29% yield) and *E*-**4cka** (55% yield), a global 84% yield.

N-((Z)-((2S*,6R*)-6-Methoxy-6-methyl-2-phenyldihydro-2H-pyran-3(4H)-ylidene)methyl)-4-methyl-N-phenylbenzenesulfonamide (*Z*-**4cka**)

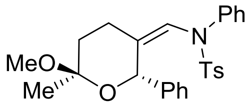

Z-**4cka**

Minor isomer. 29% yield. Colorless oil. **^{1}H NMR** (500 MHz, $CDCl_3$) δ 8.13 (d, *J* = 7.7 Hz, 2H), 7.63 (q, *J* = 7.8, 7.4 Hz, 1H), 7.49 (t, *J* = 7.7 Hz, 2H), 7.36 (d, *J* = 8.0 Hz, 2H), 7.25 – 7.03 (m, 5H), 6.69 (d, *J* = 7.3 Hz, 2H), 6.17 (s, 1H), 4.96 (s, 1H), 2.98 (s, 3H), 2.49 – 2.41 (m, 1H), 2.40 (s, 3H), 2.29 (dt, *J* = 13.7, 4.8 Hz, 1H), 2.05 (td, *J* = 10.6, 9.5, 4.0 Hz, 1H), 1.70 (td, *J* = 12.6, 5.7 Hz, 1H), 1.30 (s, 3H). **^{13}C NMR** (75 MHz, $CDCl_3$) δ 143.6 (C), 139.9 (C), 139.4 (C), 138.3 (C), 134.4 (C), 133.7 (CH), 130.2 (CH), 129.3 (CH), 128.4 (CH), 128.3 (CH), 128.1 (CH), 127.8 (CH), 127.2 (CH), 120.5 (CH), 99.1 (C), 71.8 (CH), 48.5 (CH_3), 36.4 (CH_2), 25.8 (CH_2), 23.8 (CH_3), 21.5 (CH_3). **LRMS** (*m/z, ESI*): 486.17 (M+Na)$^+$, 472.15, 381.30, 353.27, 185.10. **HRMS** Calculated for $C_{27}H_{29}NNaO_4S$, 486.1710 found 486.1706.

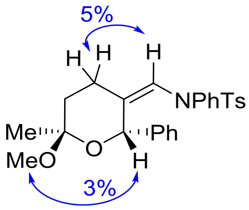

Figure S22. Significant nOe's observed for *Z*-**4cka**.

3-((Z)-((2S*,6S*)-2-Butyl-6-methyl-6-phenyldihydro-2H-pyran-3(4H)-ylidene)methyl)oxazolidin-2-one (4abb).[32]

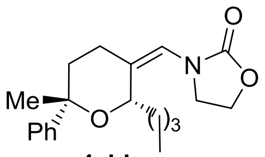

4abb

Major isomer. 73% yield. White solid. **^{1}H NMR** (500 MHz, $CDCl_3$) δ 7.48 (d, *J* = 7.7 Hz, 2H), 7.32 (t, *J* = 7.7 Hz, 2H), 7.21 (t, *J* = 7.3 Hz, 1H), 6.01 (s, 1H), 4.72 (d, *J* = 7.6 Hz, 1H), 4.39 (t, *J* = 8.0 Hz, 2H), 3.87 (q, *J* = 8.2 Hz, 1H), 3.70 (q, *J* = 8.3 Hz, 1H), 2.47 (td, *J* = 12.7, 5.9 Hz, 1H), 2.40 – 2.32 (m, 1H), 2.14 (dt, *J* = 13.4, 4.3 Hz, 1H), 1.90 (td, *J* = 12.5, 5.4 Hz, 1H), 1.70 – 1.58 (m, 1H), 1.52 (s, 3H), 1.57 – 1.46 (m, 2H), 1.47 – 1.36 (m, 1H), 1.38 – 1.18 (m, 2H), 0.88 (t, *J* = 7.3 Hz, 3H). **^{13}C NMR** (75 MHz, $CDCl_3$) δ 156.8 (C), 149.6 (C), 133.7 (C), 128.0 (CH), 126.3 (CH), 124.7 (CH), 115.8 (CH), 74.3 (C), 70.7 (CH), 62.1 (CH_2), 45.9 (CH_2), 37.9 (CH_2), 34.3 (CH_2), 30.2 (CH_3), 28.0 (CH_2), 25.9 (CH_2), 22.6 (CH_2), 14.0 (CH_3). **LRMS** (*m/z, ESI*): 352.19 (M+Na)$^+$, 312.20, 243.17, 225.16, 194.11, 169.10, 155.09, 123.12. **HRMS** Calculated for $C_{20}H_{27}NNaO_3$: 352.1883, found 352.1891.

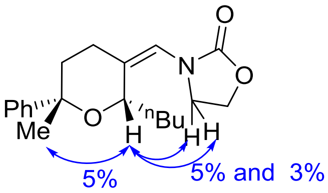

Figure S23. Significant nOe's observed for **4abb**.

[32] (a) The cycloaddition of **1a**, **2b** and **3b** provided after 2 h at -45 ºC the adducts **4abb** and **4abb'** in a 3:1 diastereoisomeric ratio (measured by ^{1}H NMR in the crude mixture). The isomers could be easily separated by column chromatography to yield **4abb** (73% yield) and **4abb'** (24% yield), a global 97% yield. (b) When the reaction was carried out with an allenamide (**1a**) / alkene (**2b**) / aldehyde (**3b**) molar ratio of 1.0 / 1.25 / 2.0, at -45 ºC (Table 2, footnote *b*), the products were isolated in a global 84% yield (*dr* = 3 : 1).

3-((*Z*)-((2*R,6*S**)-2-Butyl-6-methyl-6-phenyldihydro-2H-pyran-3(4H)-ylidene)methyl)oxazolidin-2-one (4abb').**[32]

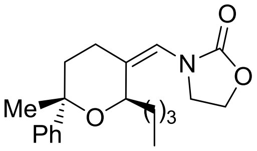

4abb'

Minor isomer. 24% yield. **^{1}H NMR** (500 MHz, $CDCl_3$) δ 7.39 – 7.35 (m, 2H), 7.33 – 7.29 (m, 2H), 7.23 – 7.18 (m, 1H), 5.89 (t, *J* = 1.7 Hz, 1H), 4.26 – 4.17 (m, 3H), 3.50 – 3.41 (m, 1H), 3.05 – 2.96 (m, 1H), 2.59 – 2.48 (m, 1H), 2.28 – 2.21 (m, 2H), 2.19 – 2.11 (m, 1H), 1.75 – 1.66 (m, 1H), 1.67 – 1.51 (m, 2H), 1.43 (s, 3H), 1.44 – 1.22 (m, 3H), 0.93 (t, *J* = 7.2 Hz, 3H). **^{13}C NMR** (75 MHz, $CDCl_3$) δ 157.1 (C), 147.1 (C), 133.6 (C), 128.1 (CH), 126.5 (CH), 125.7 (CH), 116.3 (CH), 75.4 (C), 70.8 (CH), 62.1 (CH_2), 45.3 (CH_2), 35.1 (CH_2), 34.0 (CH_2), 33.8 (CH_3), 27.6 (CH_2), 25.6 (CH_2), 22.8 (CH_2), 14.1 (CH_3). **^{1}H NMR** (500 MHz, C_6D_6) δ 7.39 – 7.33 (m, 2H), 7.24 – 7.16 (m, 2H), 7.08 (tt, *J* = 6.9, 1.2 Hz, 1H), 6.04 (t, *J* = 1.8 Hz, 1H), 4.27 – 4.20 (m, 1H), 3.24 – 3.08 (m, 2H), 2.64 (td, *J* = 8.8, 5.4 Hz, 1H), 2.45 – 2.34 (m, 1H), 2.09 (q, *J* = 8.6 Hz, 1H), 1.96 (dd, *J* = 8.6, 4.5 Hz, 2H), 1.82 (dt, *J* = 13.1, 4.4 Hz, 1H), 1.79 – 1.67 (m, 2H), 1.66 – 1.57 (m, 1H), 1.60 – 1.50 (m, 1H), 1.41 (s, 3H), 1.41 – 1.25 (m, 2H), 0.93 (t, *J* = 7.3 Hz, 3H). **^{13}C NMR** (126 MHz, C_6D_6) δ 156.7 (C), 148.2 (C), 130.8 (C), 128.5 (CH), 126.8 (CH), 126.0 (CH), 117.5 (CH), 75.4 (C), 70.9 (CH), 61.3 (CH_2), 44.8 (CH_2), 36.3 (CH_2), 34.7 (CH_2), 34.2 (CH_3), 28.1 (CH_2), 26.1 (CH_2), 23.3 (CH_2), 14.4 (CH_3). **LRMS** (*m/z, ESI*): 352.19 $(M+Na)^+$, 298.05, 243.17, 225.16. **HRMS** Calculated for $C_{20}H_{27}NNaO_3$: 352.1883, found 352.1892.

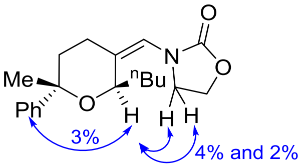

Figure S24. Significant nOe's observed for **4abb'** (in C_6D_6).

3-((*Z*)-((2*S,6*S**)-2-Isopropyl-6-methyl-6-phenyldihydro-2H-pyran-3(4*H*)-ylidene)methyl)oxazolidin-2-one (4abc).**[33]

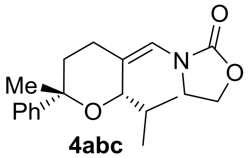

4abc

Major isomer. 71% yield. White solid. **^{1}H NMR** (300 MHz, $CDCl_3$) δ 7.57 – 7.39 (m, 2H), 7.43 – 7.16 (m, 3H), 6.09 (t, *J* = 1.7 Hz, 1H), 4.59 – 4.51 (m, 1H), 4.41 (t, *J* = 8.0 Hz, 2H), 3.92 (q, *J* = 8.1 Hz, 1H), 3.65 (q, *J* = 8.5 Hz, 1H), 2.49 – 2.21 (m, 2H), 2.16 – 1.71 (m, 3H), 1.54 (s, 3H), 1.06 (dd, *J* = 6.8, 2.6 Hz, 6H). **^{13}C NMR** (75 MHz, $CDCl_3$) δ 156.7 (C), 150.5 (C), 133.6 (C), 127.9 (CH), 126.2 (CH), 124.5 (CH), 116.3 (CH), 74.0 (C), 74.0 (CH), 62.1 (CH_2), 45.6 (CH_2), 39.1 (CH_2), 32.3 (CH), 29.1 (CH_3), 27.1 (CH_2), 19.9 (CH_3), 16.7 (CH_3). **LRMS** (*m/z, ESI*): 338.17 $(M+Na)^+$, 298.05, 229.16, 211.15, 155.09, 107.09. **HRMS** Calculated for $C_{19}H_{25}NNaO_3$: 338.1727, found 338.1733.

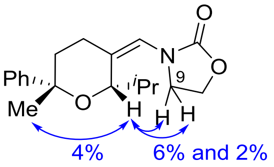

Figure S25. Significant nOe's observed for **4abc**.

---

[33] (a) The cycloaddition of **1a**, **2b** and **3c** provided after 10 min at -45 ºC the adducts **4abc** and **4abc'** in a 4:1 diastereoisomeric ratio (measured by ^{1}H NMR in the crude mixture). These isomers could be separated by column chromatography to yield **4abc** (71% yield) and **4abc'** (15% yield), a global 86% yield. (b) When the reaction was carried out with an allenamide (**1a**) / alkene (**2b**) / aldehyde (**3c**) molar ratio of 1.0 / 1.25 / 2.0, at -45 ºC (Table 2, footnote *b*), the products were isolated in a global 75% yield (*dr* = 2.5 : 1).

3-((*Z*)-((2*R,6*S**)-2-Isopropyl-6-methyl-6-phenyldihydro-2H-pyran-3(4H)-ylidene)methyl)oxazolidin-2-one (4abc').**[33]

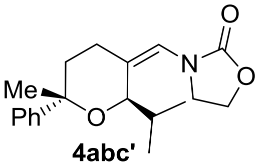

4abc'

Minor isomer. 15% yield. **^{1}H NMR** (500 MHz, $CDCl_3$) δ 7.40 – 7.33 (m, 2H), 7.34 – 7.27 (m, 2H), 7.20 (ddt, *J* = 7.9, 6.7, 1.3 Hz, 1H), 6.03 (t, *J* = 1.8 Hz, 1H), 4.27 – 4.15 (m, 2H), 4.04 (dt, *J* = 4.5, 1.7 Hz, 1H), 3.56 – 3.47 (m, 1H), 2.92 – 2.83 (m, 1H), 2.49 (dddt, *J* = 12.7, 10.5, 6.8, 1.7 Hz, 1H), 2.30 – 2.19 (m, 2H), 2.19 – 2.11 (m, 1H), 1.98 – 1.87 (m, 1H), 1.41 (s, 3H), 1.02 (dd, *J* = 12.4, 6.8 Hz, 6H) **^{13}C NMR** (75 MHz, $CDCl_3$) δ 157.2 (C), 147.4 (C), 131.5 (C), 128.1 (CH), 126.4 (CH), 125.7 (CH), 117.0 (CH), 75.0 (C), 74.7 (CH), 62.1 (CH_2), 44.8 (CH_2), 35.4 (CH_2), 34.0 (CH_3), 32.2 (CH), 26.4 (CH_2), 19.6 (CH_3), 16.5 (CH_3). **LRMS** (*m/z, ESI*): 338.17 $(M+Na)^+$, 298.05, 229.16,. **HRMS** Calculated for $C_{19}H_{25}NNaO_3$: 338.1727, found 338.1731.

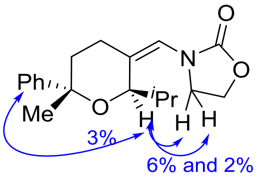

Figure S26. Significant nOe's observed for **4abc'**.

(*Z*)-3-((2-Cyclopropyl-6,6-diphenyldihydro-2H-pyran-3(4H)-ylidene)methyl)oxazolidin-2-one (*Z*-**4afd**).[34]

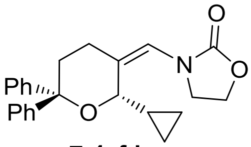

Z-**4afd**

Major isomer. 81% yield. White solid. **^{1}H NMR** (500 MHz, $CDCl_3$) δ 7.39 – 7.33 (m, 4H), 7.29 – 7.21 (m, 4H), 7.20 – 7.11 (m, 2H), 5.87 (s, 1H), 4.30 – 4.14 (m, 2H), 3.99 (d, *J* = 6.7 Hz, 1H), 3.46 (td, *J* = 8.7, 6.5 Hz, 1H), 3.09 (td, *J* = 8.8, 7.7 Hz, 1H), 2.71 – 2.58 (m, 2H), 2.46 – 2.37 (m, 1H), 2.38 – 2.28 (m, 1H), 1.13 – 0.99 (m, 1H), 0.61 – 0.52 (m, 1H), 0.52 – 0.41 (m, 2H), 0.44 – 0.34 (m, 1H). **^{13}C NMR** (75 MHz, $CDCl_3$) δ 157.25 (C), 148.43 (C), 145.08 (C), 133.28 (C), 128.02 (CH), 127.84 (CH), 127.13 (CH), 126.71 (CH), 126.31 (CH), 125.47 (CH), 116.93 (CH), 78.82 (C), 73.62 (CH), 62.06 (CH_2), 45.75 (CH_2), 35.66 (CH_2), 26.26 (CH_2), 15.03 (CH), 2.90 (CH_2), 2.75 (CH_2). **LRMS** (*m/z, ESI*): 398.17 $(M+Na)^+$, 358.18, 289.16, 193.10, 167.09, 117.07, 91.05. **HRMS** Calculated for $C_{24}H_{25}NNaO_3$: 398.1727, found 398.1731.

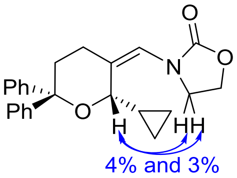

Figure S27. Significant nOe's observed for *Z*-**4afd**.

(*E*)-3-((2-Cyclopropyl-6,6-diphenyldihydro-2H-pyran-3(4H)-ylidene)methyl)oxazolidin-2-one (*E*-**4afd**)

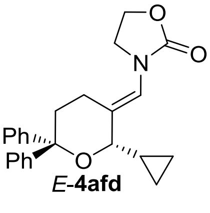

E-**4afd**

Minor isomer. 11% yield. White solid. **^{1}H NMR** (500 MHz, $CDCl_3$) δ 7.51 (dd, *J* = 8.4, 1.3 Hz, 2H), 7.43 – 7.38 (m, 2H), 7.32 – 7.20 (m, 5H), 7.18 – 7.14 (m, 1H), 5.77 (s, 1H), 4.67 (d, *J* = 9.6 Hz, 1H), 4.46 (td, *J* = 8.9, 4.4 Hz, 1H), 4.41 – 4.32 (m, 1H), 3.96 (q, *J* = 9.2 Hz, 1H), 3.67 (td, *J* = 8.8, 4.4 Hz, 1H), 2.82 (dt, *J* = 14.9, 4.2 Hz, 1H), 2.76 (dt, *J* = 13.9, 4.1 Hz, 1H), 2.46 – 2.37 (m, 1H), 2.10 (ddd, *J* = 13.8, 12.8, 4.2 Hz, 1H), 1.52 – 1.40 (m, 1H), 0.81 – 0.67 (m, 2H), 0.38 – 0.24 (m, 2H). **^{13}C NMR** (75 MHz, $CDCl_3$) δ 158.47 (C), 147.77

[34] (a) The cycloaddition of **1a**, **2f** and **3d** provided after 3 h at -78 ºC the adducts *Z*-**4afd** and *E*-**4afd** in a 8 :1 ratio (measured by ^{1}H NMR in the crude mixture) and. These Z/E isomers could be separated by column chromatography to yield *Z*-**4afd** (81% yield) and *E*-**4afd** (11% yield), a global 92% yield. (b) When the reaction was carried out with an allenamide (**1a**) / alkene (**2f**) / aldehyde (**3d**) molar ratio of 1.0 / 1.25 / 2.0, at -45 ºC (Table 2, footnote *b*), the products were isolated in a global 74% yield (*Z/E* = 1:7).

(C), 141.49 (C), 128.97 (C), 128.87 (CH), 128.46 (CH), 128.01 (CH), 127.42 (CH), 127.12 (CH), 126.66 (CH), 80.85 (C), 79.08 (CH), 62.64 (CH_2), 40.22 (CH_2), 35.02 (CH_2), 23.03 (CH_2), 9.29 (CH), 7.13 (CH_2), 6.82 (CH_2).). **LRMS** (m/z, ESI): 398.17 $(M+ Na)^+$, 357.13, 289.16, 271.15, 193.10, 167.09, 117.07. **HRMS** Calculated for $C_{24}H_{25}NNaO_3$: 398.1727, found 398.1732.

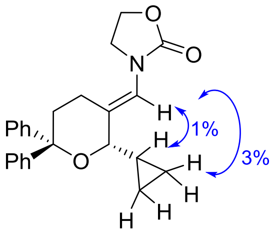

Figure S28. Significant nOe's observed for *E*-**4afd**.

3-((Z)-((2S*,6S*)-2-(But-3-en-1-yl)-6-methyl-6-phenyldihydro-2H-pyran-3(4H)-ylidene)methyl)oxazolidin-2-one (**4abe**).[35]

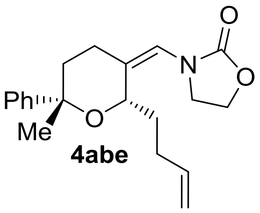

4abe

Major isomer. White solid. **^{1}H NMR** (500 MHz, $CDCl_3$) δ 7.52 – 7.45 (m, 2H), 7.33 (t, *J* = 7.7 Hz, 2H), 7.22 (t, *J* = 7.3 Hz, 1H), 6.02 (t, *J* = 1.7 Hz, 1H), 5.87 – 5.74 (m, 1H), 5.04 (dq, *J* = 17.1, 1.7 Hz, 1H), 4.99 – 4.95 (m, 1H), 4.77 – 4.72 (m, 1H), 4.44 – 4.33 (m, 2H), 3.91 – 3.83 (m, 1H), 3.73 – 3.66 (m, 1H), 2.53 – 2.44 (m, 1H), 2.37 (ddd, *J* = 13.1, 6.0, 3.2 Hz, 1H), 2.30 – 2.23 (m, 2H), 2.19 – 2.12 (m, 1H), 1.91 (td, *J* = 12.5, 12.1, 5.3 Hz, 1H), 1.81 – 1.71 (m, 1H), 1.61 – 1.54 (m, 1H), 1.53 (s, 3H). **^{13}C NMR** (75 MHz, $CDCl_3$) δ 157.00 (C), 149.71 (C), 138.30 (CH), 133.62 (C), 128.07 (CH), 126.46 (CH), 124.70 (CH), 116.11 (CH), 115.06 (CH_2), 74.39 (C), 69.63 (CH), 62.04 (CH_2), 45.88 (CH_2), 37.81 (CH_2), 33.47 (CH_2), 30.04 (CH_3), 29.72 (CH_2), 25.75 (CH_2). **LRMS** (m/z, ESI): 350.17 $(M+Na)^+$, 266.11, 241.16, 223.15, 182.11, 125.99. **HRMS** calculated for $C_{20}H_{25}NNaO_3$: 350.1727, found 350.1741.

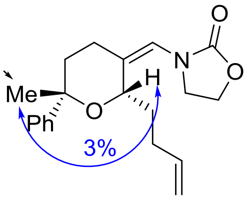

Figure S29. Significant nOe's observed for **4abe**.

3-((Z)-((2R*,6S*)-2-(But-3-en-1-yl)-6-methyl-6-phenyldihydro-2H-pyran-3(4H)-ylidene)methyl)oxazolidin-2-one (**4abe'**).[35]

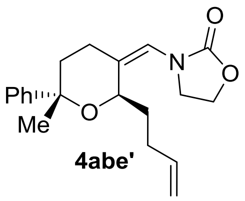

4abe'

Minor isomer. White solid. **^{1}H NMR** (500 MHz, $CDCl_3$) δ 7.41 – 7.35 (m, 2H), 7.31 (t, *J* = 7.7 Hz, 2H), 7.25 – 7.18 (m, 1H), 5.89 (t, *J* = 1.6 Hz, 1H), 5.90 – 5.79 (m, 1H), 5.09 (dq, *J* = 17.1, 1.6 Hz, 1H), 5.02 – 4.95 (m, 1H), 4.28 – 4.21 (m, 1H), 4.25 – 4.18 (m, 2H), 3.48 – 3.42 (m, 1H), 2.99 (q, *J* = 8.7 Hz, 1H), 2.58 – 2.50 (m, 1H), 2.38 – 2.29 (m, 1H), 2.29 – 2.21 (m, 3H), 2.20 – 2.14 (m, 1H), 1.87 – 1.78 (m, 1H), 1.74 – 1.66 (m, 1H), 1.45 (s, 3H). **^{13}C NMR** (126 MHz, $CDCl_3$) δ 157.15 (C), 146.87 (C), 138.43 (CH),

[35] (a) The cycloaddition of **1a**, **2b** and **3e** provided after 10 min at -45 ºC the adducts **4abe** and **4abe'** in a 3 : 1 diastereoisomeric ratio (measured by ^{1}H NMR in the crude mixture) and a global 92% yield. The data of **4abe** and **4abe'** was obtained from pure samples of each isomer, obtained by column chromatography. However, a complete separation of both isomers was not possible. (b) When the reaction was carried out with an allenamide (**1a**) / alkene (**2b**) / aldehyde (**3e**) molar ratio of 1.0 / 1.25 / 2.0, at -45 ºC (Table 2, footnote *b*), the [2+2+2] products were isolated in a global 74% yield (*dr* = 4 : 1). A 10% yield of the [2+2] adduct **5ab** was also isolated.

133.32 (C), 128.10 (CH), 126.54 (CH), 125.72 (CH), 116.52 (CH), 114.95 (CH_2), 75.47 (C), 69.97 (CH), 62.08 (CH_2), 45.29 (CH_2), 34.98 (CH_2), 33.80 (CH_3), 33.49 (CH_2), 29.51 (CH_2), 25.48 (CH_2). **LRMS** (*m/z, ESI*): 350.17 $(M+Na)^+$, 282.08, 241.16, 223.15, 155.09, 125.99. **HRMS** Calculated for $C_{20}H_{25}NNaO_3$: 350.1727, found 350.1739.

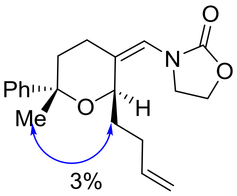

Figure S30. Significant nOe's observed for **4abe'**.

3-((Z)-((2S*,6S*)-2-((E)-But-2-en-2-yl)-6-methyl-6-phenyldihydro-2H-pyran-3(4H)-ylidene)methyl)oxazolidin-2-one (**4abf**).[36]

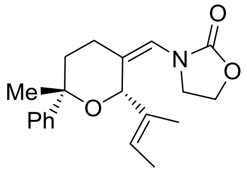

4abf

Major isomer. White solid. ^{1}**H NMR** (500 MHz, $CDCl_3$) δ 7.52 – 7.47 (m, 2H), 7.36 – 7.31 (m, 2H), 7.25 – 7.20 (m, 1H), 5.95 (t, *J* = 1.6 Hz, 1H), 5.67 – 5.61 (m, 1H), 5.02 (s, 1H), 4.34 – 4.24 (m, 2H), 3.76 (td, *J* = 8.8, 7.2 Hz, 1H), 3.56 (td, *J* = 8.8, 6.7 Hz, 1H), 2.47 – 2.34 (m, 1H), 2.27 – 2.19 (m, 2H), 1.96 – 1.87 (m, 1H), 1.76 (t, *J* = 1.1 Hz, 3H), 1.64 (dd, *J* = 6.8, 1.2 Hz, 3H), 1.61 (s, 3H). ^{13}**C NMR** (75 MHz, $CDCl_3$) δ 156.16 (C), 149.31 (C), 134.88 (C), 134.23 (C), 127.91 (CH), 126.29 (CH), 124.64 (CH), 123.45 (CH), 116.60 (CH), 76.10 (CH), 74.90 (C), 62.03 (CH_2), 45.81 (CH_2), 37.55 (CH_2), 27.90 (CH_3), 26.21 (CH_2), 13.42 (CH_3), 12.75 (CH_3). **LRMS** (*m/z, ESI*): 350.1729 $(M+Na)^+$, 223.1472, 162.0913, 131.0862, 105.0691. **HRMS** Calculated for $C_{20}H_{25}NNaO_3$ 350.1727, found 350.1729.

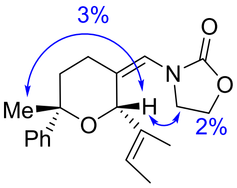

Figure S31. Significant nOe's observed for **4abf**.

3-((Z)-((2R*,6S*)-2-((E)-But-2-en-2-yl)-6-methyl-6-phenyldihydro-2H-pyran-3(4H)-ylidene)methyl)oxazolidin-2-one (**4abf'**).[36]

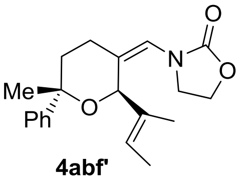

4abf'

Minor isomer. White solid. ^{1}**H NMR** (500 MHz, $CDCl_3$) δ 7.43 – 7.39 (m, 2H), 7.36 (dd, *J* = 8.6, 6.9 Hz, 2H), 7.27 – 7.23 (m, 1H), 6.00 (t, *J* = 1.9 Hz, 1H), 5.63 (qt, *J* = 6.7, 1.2 Hz, 1H), 4.60 (s, 1H), 4.25 – 4.13 (m, 2H), 3.61 (td, *J* = 8.6, 6.5 Hz, 1H), 3.30 (td, *J* = 8.7, 7.6 Hz, 1H), 2.58 – 2.48 (m, 1H), 2.31 – 2.20 (m, 2H), 2.19 – 2.09 (m, 1H), 1.76 (t, *J* = 1.2 Hz, 3H), 1.70 (d, *J* = 6.8 Hz, 3H), 1.47 (s, 3H). ^{13}**C NMR** (126 MHz, $CDCl_3$) δ 156.69 (C), 147.25 (C), 134.68 (C), 132.66 (C), 128.29 (CH), 126.56 (CH), 125.34 (CH), 123.24 (CH), 117.08 (CH), 76.74 (CH), 75.90 (C), 62.11 (CH_2), 45.73 (CH_2), 35.17 (CH_2), 32.57 (CH_3), 26.30 (CH_2), 13.57 (CH_3), 12.69 (CH_3). **LRMS** (*m/z, ESI*): 350.1735 $(M+Na)^+$, 282.0801, 223.1478, 193.1015, 131.0841. **HRMS** Calculated for $C_{20}H_{25}NNaO_3$ 350.1727, found 350.1735.

---

[36] (a) The cycloaddition of **1a**, **2b** and **3f** provided after 1 h at -78 ºC the adducts **4abf** and **4abf'** in a 2.5 : 1 diastereoisomeric ratio (measured by ^{1}H NMR in the crude mixture) and a global 94% yield. The data of **4abf** and **4abf'** was obtained from pure samples of each isomer, obtained by column chromatography. However, a complete separation of both isomers was not possible. (b) When the reaction was carried out with an allenamide (**1a**) / alkene (**2b**) / aldehyde (**3f**) molar ratio of 1.0 / 1.25 / 2.0, at -45 ºC (Table 2, footnote *b*), the products were isolated in a global 85% yield (*dr* = 1 : 1).

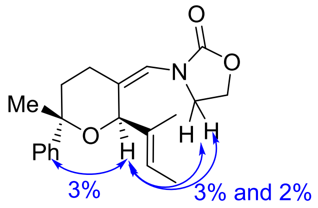

Figure S32. Significant nOe's observed for **4abf'**.

3-((Z)-((2S*,6S*)-2-((*E*)-But-2-en-2-yl)-6-methyl-6-phenyldihydro-2H-pyran-3(4H)-ylidene)methyl)oxazolidin-2-one (**4aff**)

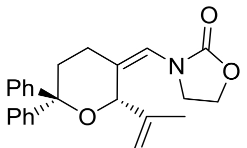

4aff

87% yield. Reaction time 2 h.[37] **^{1}H NMR** (500 MHz, $CDCl_3$) δ 7.52 – 7.46 (m, 2H), 7.42 – 7.33 (m, 4H), 7.30 – 7.23 (m, 3H), 7.20 – 7.15 (m, 1H), 5.98 (q, *J* = 1.5 Hz, 1H), 5.65 – 5.58 (qt, *J* = 6.7, 1.2 Hz, 1H), 4.64 (s, 1H), 4.18 (t, *J* = 7.9 Hz, 2H), 3.57 (q, *J* = 7.8 Hz, 1H), 3.18 (q, *J* = 8.4 Hz, 1H), 2.72 – 2.63 (m, 1H), 2.60 – 2.49 (m, 1H), 2.51 – 2.42 (m, 1H), 2.40 – 2.31 (m, 1H), 1.83 – 1.79 (t, *J* = 1.1 Hz 3H), 1.69 (d, *J* = 6.8 Hz, 3H). **^{13}C NMR** (75 MHz, $CDCl_3$) δ 156.72 (C), 148.79 (C), 144.69 (C), 134.34 (C), 132.25 (C), 128.21 (CH), 127.89 (CH), 127.26 (CH), 126.92 (CH), 126.30 (CH), 125.26 (CH), 122.70 (CH), 117.37 (CH), 79.27 (C), 76.73 (CH), 62.07 (CH_2), 45.80 (CH_2), 35.45 (CH_2), 26.30 (CH_2), 13.38 (CH_3), 12.97 (CH_3). **LRMS** (*m/z, ESI*): 412.1869 $(M+Na)^+$, 285.1642, 193.1009. **HRMS** Calculated for $C_{25}H_{27}NNaO_3$: 412.1883, found 412.1869.

(S,Z)-3-((6,6-Diphenyl-2-(prop-1-en-2-yl)dihydro-2H-pyran-3(4H)-ylidene)methyl)oxazolidin-2-one (**4afg**)

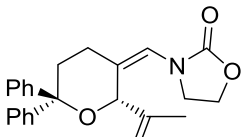

4afg

93% yield. Reaction time 15 h.[38] **^{1}H NMR** (500 MHz, $CDCl_3$) δ 7.51 – 7.46 (m, 2H), 7.40 – 7.32 (m, 4H), 7.30 – 7.24 (m, 3H), 7.20 – 7.15 (m, 1H), 6.07 (q, *J* = 1.4 Hz, 1H), 5.14 – 5.10 (m, 1H), 5.01 (p, *J* = 1.5 Hz, 1H), 4.71 (s, 1H), 4.20 (t, *J* = 8.0 Hz, 2H), 3.60 – 3.55 (m, 1H), 3.08 (q, *J* = 8.3 Hz, 1H), 2.80 – 2.70 (m, 1H), 2.62 – 2.52 (m, 1H), 2.51 – 2.44 (m, 1H), 2.38 – 2.29 (m, 1H), 1.91 (s, 3H). **^{13}C NMR** (75 MHz, $CDCl_3$) δ 156.82 (C), 148.91 (C), 144.58 (C), 143.59 (C), 129.85 (C), 128.20 (CH), 127.95 (CH), 127.37 (CH), 126.97 (CH), 126.32 (CH), 125.15 (CH), 117.98 (CH), 113.35 (CH_2), 79.43 (C), 74.88 (CH), 62.02 (CH_2), 45.40 (CH_2), 35.28 (CH_2), 25.83 (CH_2), 19.08 (CH_3). **LRMS** (m/z, ESI): 398.1725 $(M+Na)^+$, 271.1478, 193.1007, 167.0856. **HRMS** Calculated for $C_{24}H_{25}NNaO_3$: 398.1727, found 398.1725.

3-((Z)-((2S*,4R*,6S*)-2-((E)-But-2-en-2-yl)-4,6-dimethyl-6-phenyldihydro-2H-pyran-3(4H)-ylidene)methyl) oxazolidin-2-one (4bbf)[39]

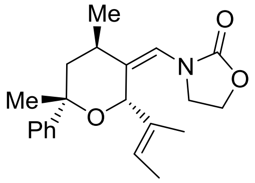

4bbf

^{1}H NMR (300 MHz, $CDCl_3$) δ 7.49 (dd, *J* = 7.6, 1.7 Hz, 2H), 7.38 – 7.14 (m, 3H), 5.78 (t, *J* = 2.1 Hz, 1H), 5.61 (q, *J* = 6.7 Hz, 1H), 5.09 (s, 1H), 4.38 – 4.16 (m, 2H), 3.76 (q, *J* = 8.6 Hz, 1H), 3.50 (q, 1H), 2.65 – 2.47 (m, 1H), 2.21 (dd, *J* = 13.0, 4.7 Hz, 1H), 1.75 (s, 3H), 1.65 (d, *J* = 6.6 Hz, 3H), 1.62 – 1.59 (m, 1H), 1.58 (s, 3H), 1.05 (d, *J* = 6.5 Hz, 3H). **^{13}C NMR** (75 MHz, $CDCl_3$) δ 155.89 (C), 149.86 (C), 141.17 (C), 134.23 (C), 127.96 (CH), 126.26 (CH), 124.56 (CH), 123.27 (CH), 115.11 (CH), 76.16 (CH), 74.98 (C), 61.98 (CH_2), 47.42 (CH_2), 45.90 (CH_2), 29.32 (CH), 28.32 (CH_3), 17.22 (CH_3), 13.46 (CH_3), 12.47 (CH_3).

---

37 Reaction carried out at -78 ºC.

38 (a) Reaction carried out at -78 ºC. (b) When the reaction was carried out with an allenamide (**1a**) / alkene (**2f**) / aldehyde (**3g**) molar ratio of 1.0 / 1.25 / 2.0, at -45 ºC (Table 2, footnote *b*), the product was isolated in 75% yield.

39 Reaction carried out at -78ºC for 1.5 h. The cycloadducts **4bbf**, **4bbf'** and **4bbf''** were obtained in a global 84% yield and a diastereomeric ratio of 10 : 2: 1. Pure samples of the two major isomers (**4bbf**, **4bbf'**) were isolated by flash chromatography.

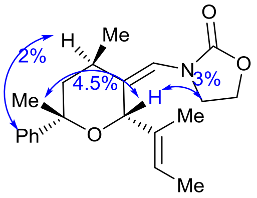

Figure S33. Significant nOe's observed for **4bbf**.

3-((Z)-((2R*,4R*,6S*)-2-((E)-but-2-en-2-yl)-4,6-dimethyl-6-phenyldihydro-2H-pyran-3(4H)-ylidene)methyl) oxazolidin-2-one (4bbf')[39]

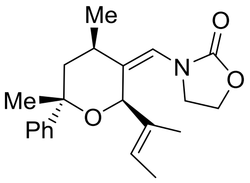

4bbf'

^{1}H NMR (300 MHz, $CDCl_3$) δ 7.45 – 7.27 (m, 4H), 7.29 – 7.16 (m, 1H), 5.89 (s, 1H), 5.64 (q, *J* = 6.4 Hz, 1H), 4.64 (s, 1H), 4.20 (t, *J* = 7.8 Hz, 2H), 3.61 (q, *J* = 8.0 Hz, 1H), 3.39 (q, *J* = 8.1 Hz, 1H), 2.49 – 2.39 (m, 1H), 2.34 (dd, *J* = 13.5, 5.1 Hz, 1H), 1.89 (dd, *J* = 13.4, 9.3 Hz, 1H), 1.72 (s, 3H), 1.68 (d, *J* = 6.7 Hz, 3H), 1.44 (s, 3H), 1.23 (d, *J* = 6.7 Hz, 3H). **^{13}C NMR** (75 MHz, $CDCl_3$) δ 156.98 (C), 146.54 (C), 136.07 (C), 135.38 (C), 128.32 (CH), 126.51 (CH), 125.43 (CH), 122.02 (CH), 117.97 (CH), 76.84 (CH), 76.44 (C), 62.14 (CH_2), 46.61 (CH_2), 41.75 (CH_2), 34.10 (CH_3), 31.38 (CH), 21.05 (CH_3), 13.51 (CH_3), 13.21 (CH_3).

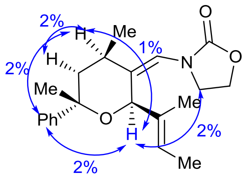

Figure S34. Significant nOe's observed for **4bbf'**.

3-((Z)-((2S,4S,6S)-2-((E)-but-2-en-2-yl)-4,6-dimethyl-6-phenyldihydro-2H-pyran-3(4H)-ylidene)methyl) oxazolidin-2-one (4bff'')[40]

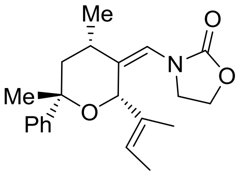

4bbf''

^{1}H NMR (300 MHz, $CDCl_3$) δ 7.47 – 7.40 (m, 2H), 7.34 – 7.26 (m, 2H), 7.24 – 7.15 (m, 1H), 6.04 (t, *J* = 1.7 Hz, 1H), 5.57 (q, *J* = 6.8 Hz, 1H), 5.00 (s, 1H), 4.39 – 4.20 (m, 2H), 3.73 – 3.63 (m, 1H), 3.63 – 3.52 (m, 1H), 2.74 (q, *J* = 6.7, 6.2 Hz, 1H), 2.05 – 1.96 (m, 2H), 1.59 (s, 3H), 1.56 (d, *J* = 6.0 Hz, 3H), 1.53 (s, 3H), 1.18 (d, *J* = 6.9 Hz, 3H).

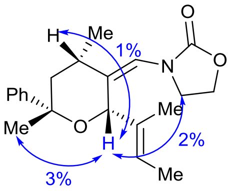

Figure S35. Significant nOe's observed for **4bbf''**.

---

[40] Data deduced from a 1:1 mixture of the major isomer (**4bbf**) and **4bbf''**

Procedure for the Os-catalyzed dihydroxilation of the tetrahydropyranic products (Exemplified for the reaction of **4afd**).

To a stirred solution of (Z)-3-((2-cyclopropyl-6,6-diphenyl-2H-pyran-3(4H,5H,6H)-ylidene)methyl)oxazolidin-2-one (**4afd**, 62 mg, 0.165 mmol) in an Acetone:MeCN 1:1 mixture (0.5 ml), NMO (39.0 mg, 0.330 mmol), H_2O (0.25 ml), and OsO_4 (0.052 ml, 8.26 µmol, 4 wt. % solution in H_2O) were successively added. After being stirred at *rt* for 1.5 h, the reaction was quenched by addition of NaS_2O_3 (sat) (3 ml) and the resulting mixture was further stirred for 30 min. The product was extracted with CH_2Cl_2 (3 x 5ml), and the organic phases were dried, filtered and evaporated to afford a crude which contains a 8:1 mixture (^{1}H-NMR) of **11** and its epimer at C-3 (epi-**11**). This crude residue was chromatographed in hexanes/Et_2O (10-20%) to give **11** (35 mg, 66% yield) and **11'** (5.4 mg, 10% yield).

(2S*,3R*)-2-Cyclopropyl-3-hydroxy-6,6-diphenyltetrahydro-2H-pyran-3-carbaldehyde (**11**)

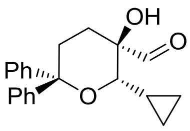

Major isomer. 66% yield. Colorless oil. ^{1}**H NMR** (500 MHz, $CDCl_3$) δ 10.36 (d, *J* = 1.0 Hz, 1H), 7.47 – 7.42 (m, 2H), 7.43 – 7.37 (m, 2H), 7.36 – 7.33 (m, 2H), 7.31 – 7.25 (m, 3H), 7.22 – 7.17 (m, 1H), 3.63 (d, *J* = 1.0 Hz, 1H), 3.40 (d, *J* = 5.5 Hz, 1H), 2.93 (ddd, *J* = 14.9, 4.2, 3.3 Hz, 1H), 2.37 (td, *J* = 14.6, 4.4 Hz, 1H), 2.14 (td, *J* = 14.0, 4.3 Hz, 1H), 1.99 (ddd, *J* = 13.7, 4.3, 3.3 Hz, 1H), 1.03 – 0.94 (m, 1H), 0.68 – 0.60 (m, 1H), 0.58 – 0.51 (m, 1H), 0.52 – 0.45 (m, 2H). ^{13}**C NMR** (126 MHz, $CDCl_3$) δ 204.81 (CH), 147.94 (C), 141.34 (C), 128.83 (CH), 128.15 (CH), 127.36 (CH), 127.16 (CH), 126.62 (CH), 124.67 (CH), 80.14 (C), 77.08 (C), 76.87 (CH), 32.90 (CH_2), 30.98 (CH_2), 10.37 (CH), 1.66 (CH_2), 1.58 (CH_2). **LRMS** (*m/z, ESI*): 345.15 $(M+Na)^+$, , 291.07, 245.08, 193.10, 178.08, 115.05. **HRMS** Calculated for $C_{21}H_{22}NaO_3$: 345.1461, found 345.1464

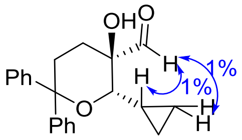

Figure S36. Significant nOe's observed for **11.**[41]

(**2S*,3S*)-2-Cyclopropyl-3-hydroxy-6,6-diphenyltetrahydro-2H-pyran-3-carbaldehyde** (*epi*-**11**)

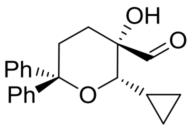

Minor isomer. 10% yield. Colorless oil.^{1}**H NMR** (500 MHz, $CDCl_3$) δ 9.52 (d, *J* = 0.6 Hz, 1H), 7.49 – 7.42 (m, 2H), 7.41 – 7.32 (m, 4H), 7.32 – 7.22 (m, 3H), 7.20 – 7.12 (m, 1H), 3.36 (d, *J* = 0.7 Hz, 1H), 2.92 (d, *J* = 8.7 Hz, 1H), 2.69 (ddd, *J* = 14.4, 3.9, 2.9 Hz, 1H), 2.31 – 2.21 (m, 1H), 2.11 (td, *J* = 13.6, 3.9 Hz, 1H), 1.65 (ddd, *J* = 13.4, 3.9, 2.9 Hz, 1H), 1.26 – 1.18 (m, 1H), 0.75 – 0.64 (m, 1H), 0.48 – 0.35 (m, 2H), 0.01 – -0.06 (m, 1H). ^{13}**C NMR** (75 MHz, $CDCl_3$) δ 203.13 (CH), 148.21 (C), 141.53 (C), 128.89 (CH), 128.16 (CH), 127.51 (CH), 127.31 (CH), 126.70 (CH), 124.99 (CH), 80.15 (C), 78.44 (C), 77.12 (CH), 28.39 (CH_2), 28.38 (CH_2), 11.13 (CH), 3.23 (CH_2), 2.56 (CH_2). **LRMS** (*m/z, ESI*): 345.15, $(M+Na)^+$, 315.03, 291.07, 193.10, 115.06. **HRMS** Calculated for $C_{21}H_{22}NaO_3$: 345.1461, found 345.1452.

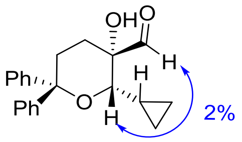

Figure S37. Significant nOe's observed for *epi*-**11.**[41]

[41] nOe's observed in **11** were not observed in its epimer (epi-**11**) and viceversa.

(2S*,3R*,6S*)-3-Hydroxy-6-(4-methoxyphenyl)-2-phenyltetrahydro-2H-pyran-3-carbaldehyde (**12**)

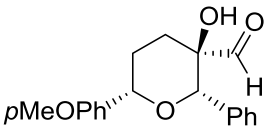

12

86% yield. Colorless oil. ^{1}**H NMR** (500 MHz, $CDCl_3$) δ 10.25 (d, *J* = 0.7 Hz, 1H), 7.45 – 7.40 (m, 2H), 7.42 – 7.36 (m, 2H), 7.35 – 7.25 (m, 3H), 6.94 (d, *J* = 8.7 Hz, 2H), 4.85 – 4.79 (m, 1H), 4.73 (s, 1H), 3.83 (s, 3H), 3.45 (d, *J* = 1.1 Hz, 1H), 2.31 – 2.14 (m, 4H). ^{13}**C NMR** (75 MHz, $CDCl_3$) δ 203.82 (CH), 159.15 (C), 136.64 (C), 133.67 (C), 128.09 (CH), 127.98 (CH), 126.93 (CH), 126.78 (CH), 113.8 (CH)1, 85.24 (CH), 80.00 (CH), 76.09 (C), 55.26 (CH3), 34.34 (CH2), 32.16 (CH2). **LRMS** (*m/z, ESI*): 335.13 $(M+Na)^+$, 229.08, 189.09, 147.08, 121.07. **HRMS** Calculated for $C_{19}H_{20}NaO_4$: 335.1254, found 335.1257.

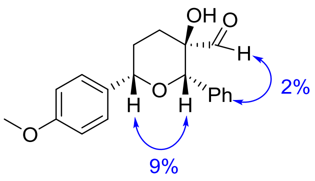

Figure S38. Significant nOe's observed for **12**

Procedure for the ozonolysis of the cycloadducts (Exemplified for the reaction of **4afd**)

(Z)-3-((2-cyclopropyl-6,6-diphenyl-2H-pyran-3(4H,5H,6H)-ylidene)methyl)oxazolidin-2-one (**4afd**, 81 mg, 0,216 mmol) was dissolved in CH_2Cl_2 (12 ml) and cooled to -78°C. Ozone was bubbled through the solution until the solution gets a deep blue. Then, Nitrogen is bubbled through the solution, Me_2S (1,25 ml, 17,0 mmol) was added and the resulting solution was allowed to reach *rt*. After 3h, the solvent was evaporated and the resulting crude residue was chromatographed to obtain (S)-2-cyclopropyl-6,6-diphenyldihydro-2H-pyran-3(4H)-one (**14**, 44 mg) in 69% yield.

(S)-2-Cyclopropyl-6,6-diphenyldihydro-2H-pyran-3(4H)-one (**14**)

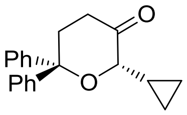

69% yield. Colorless oil. ^{1}**H NMR** (300 MHz, $CDCl_3$) δ 7.58 – 7.47 (m, 2H), 7.46 – 7.35 (m, 4H), 7.34 – 7.23 (m, 3H), 7.23 – 7.14 (m, 1H), 3.45 (d, *J* = 6.9 Hz, 1H), 3.00 – 2.86 (m, 1H), 2.70 – 2.46 (m, 3H), 1.36 – 1.18 (m, 1H), 0.74 – 0.54 (m, 2H), 0.54 – 0.36 (m, 2H). ^{13}**C NMR** (75 MHz, $CDCl_3$) δ (ppm): 209.29(C), 146.86(C), 142.67(C), 128.79(CH), 128.13(CH), 127.58(CH), 126.91(CH), 126.78(CH), 125.17(CH), 79.66(CH), 79.47(C), 35.55(CH2), 35.07(CH2), 10.82(CH), 2.31(CH_2), 1.49(CH_2). **LRMS** (*m/z, ESI*): 315.13 $(M+Na)^+$, 193.10, 178.08, 115.06. **HRMS** Calculated for $C_{20}H_{20}NaO_2$: 315.1356, found 315.1339.

(S)-2,6,6-Triphenyldihydro-2H-pyran-3(4H)-one (**13**)

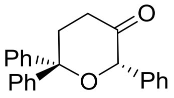

51% yield. Colorless oil. ^{1}**H NMR** (300 MHz, $CDCl_3$) δ 7.70 – 7.21 (m, 15H), 5.02 (s, 1H), 3.17 – 3.02 (m, 1H), 2.94 – 2.63 (m, 3H). ^{13}**C NMR** (75 MHz, $CDCl_3$) δ 207.78 (C), 146.69 (C), 142.39 (C), 135.85 (C), 128.98 (CH), 128.26 (CH), 127.79 (CH), 127.65 (CH), 126.94 (CH), 125.20 (CH), 80.36 (CH), 79.84 (C), 35.46 (CH_2), 34.97 (CH_2). **LRMS** (*m/z, ESI*): 351.1353 $(M+Na)^+$, 193.10, 178.08, 115.06. **HRMS** Calculated for $C_{23}H_{20}NaO_2$: 351.1356, found 351.1353.

Mechanistic experiments with deuterated alkenes

Scheme 4 (main manuscript). [2 + 2 + 2] Cycloaddition between allenamide 1a, pentanal and deuterated-*E*-2c.[42]

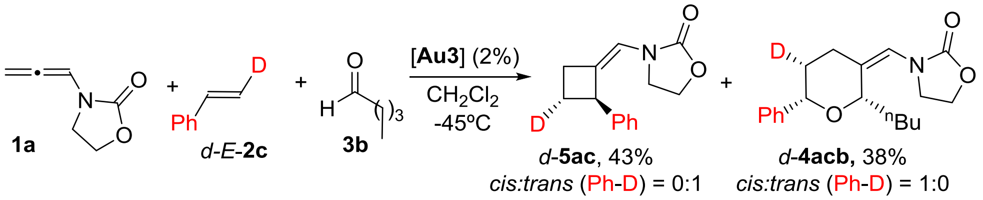

NMR spectra of **2c** vs *d-E-***2c**

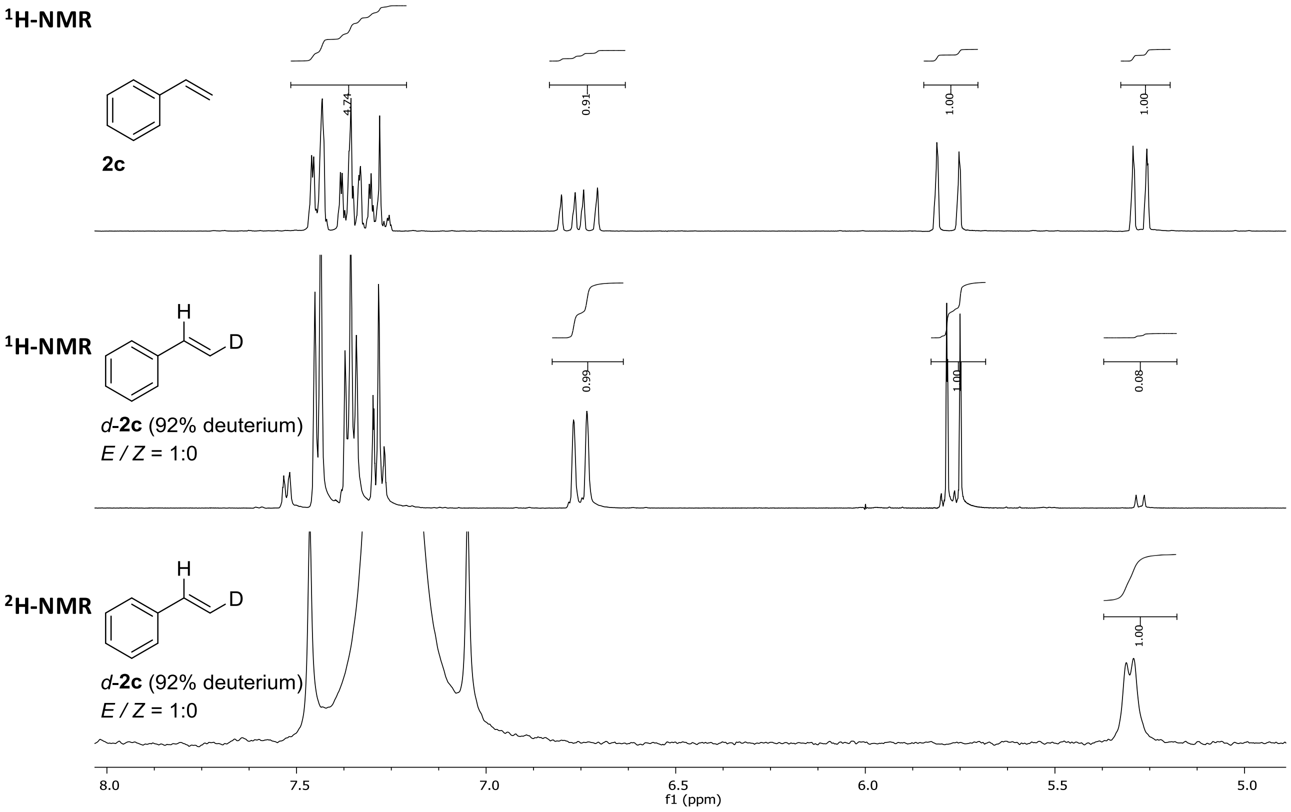

[42] (a) Cycloaddition carried out following the above-mentioned general procedure. (b) *d*-**2c** was prepared following the procedure described in: L. T. Ball, G. C. Lloyd-Jones and C. A. Russell, *Chem.-Eur. J.* 2012, **18**, 2931. See also: F. Gao and A. H. Hoveyda, *J. Am. Chem. Soc.* 2010, **132**, 10961. As can be deduce from the spectra, *d*-**2c** has a 92% content of deuterium (*E/Z* = 1:0). (c) The analog cycloaddition using styrene instead of *d-E*-**2c**, under otherwise identical conditions, provided the same yield of **5ac** and **4acb**. Cyclobutane **5ac** is a known compound (H. Faustino, P. Bernal, L. Castedo, F. López and J. L. Mascareñas, *Adv. Synth. Catal.* 2012, **354**, 1658).

3-((Z)-((2S,6S)-2-butyl-6-phenyldihydro-2H-pyran-3(4H)-ylidene)methyl)oxazolidin-2-one (**4acb**). **^{1}H NMR** (500 MHz, $CDCl_3$) δ 7.29 – 7.22 (m, 4H), 7.20 – 7.14 (m, 1H), 6.05 (t, *J* = 1.7 Hz, 1H), 4.63 – 4.56 (m, 1H), 4.38 (dd, *J* = 10.4, 6.0 Hz, 1H), 4.33 (t, *J* = 8.0 Hz, 2H), 3.82 (q, *J* = 8.1 Hz, 1H), 3.67 (q, *J* = 8.1 Hz, 1H), 2.59 – 2.43 (m, 1H), 2.22 – 2.11 (m, 1H), 2.08 – 1.97 (m, 1H), 1.95 – 1.84 (m, 1H), 1.78 – 1.69 (m, 1H), 1.60 – 1.46 (m, 3H), 1.42 – 1.34 (m, 1H), 1.34 – 1.23 (m, 1H), 0.84 (t, *J* = 7.3 Hz, 3H). **^{13}C NMR** (75 MHz, $CDCl_3$) δ 156.91 (C), 143.49 (C), 131.92 (C), 128.24 (CH), 127.03 (CH), 125.40 (CH), 117.43 (CH), 76.11 (CH), 74.75 (CH), 62.06 (CH_2), 46.11 (CH_2), 34.77 (CH_2), 33.51 (CH_2), 27.71 (CH_2), 25.96 (CH_2), 22.78 (CH_2), 14.04 (CH_3). **LRMS** (*m/z,* CI): 316.1 [M$^+$ +1], 258.1, 229.1, 185.1, 126.0. **HRMS** [M$^+$ +1], Calculated for $C_{19}H_{26}NO_3$: 316.1913, found 316.1922.

NMR spectra of **5ac** vs *d*-**5ac**:

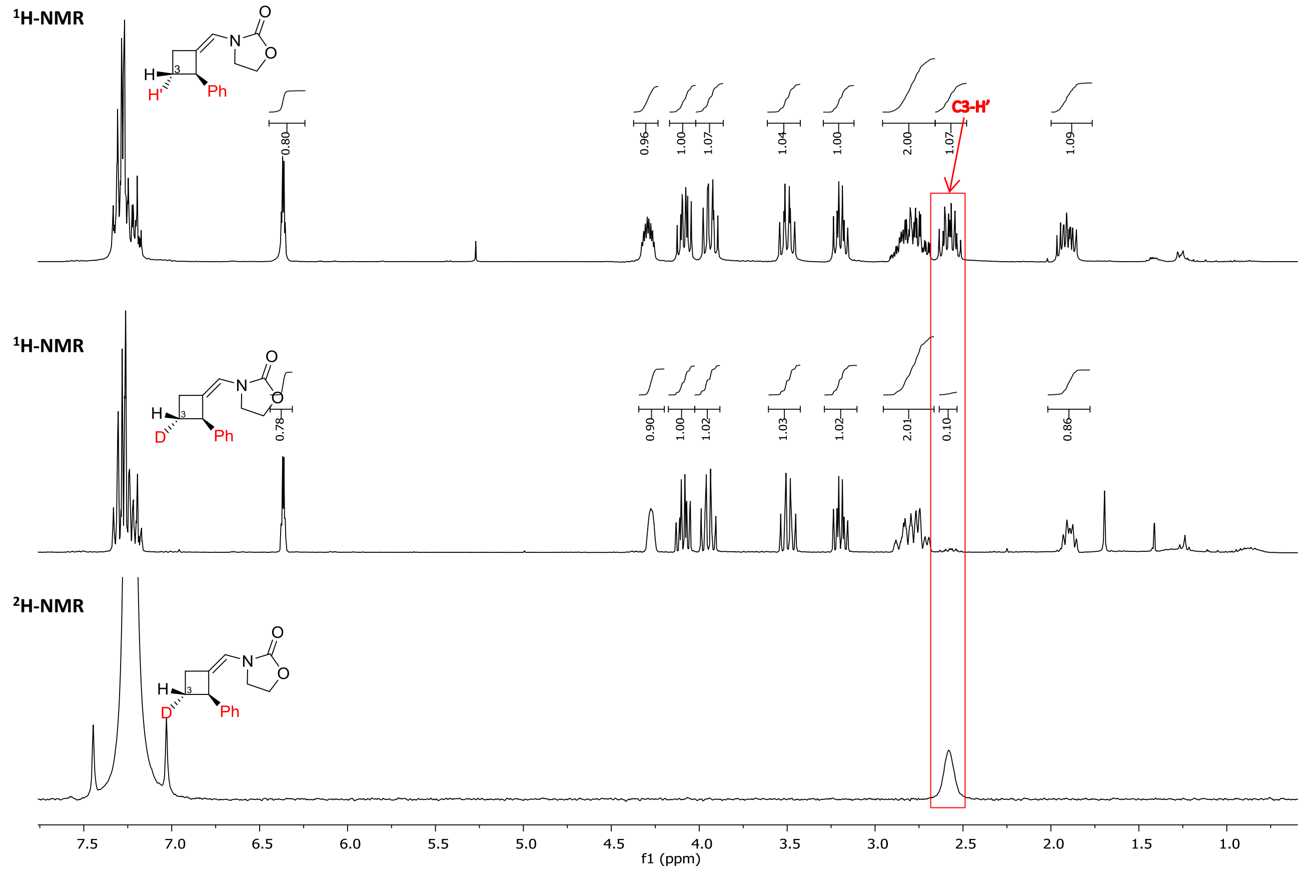

HSQC and nOe spectra for **5ac**:

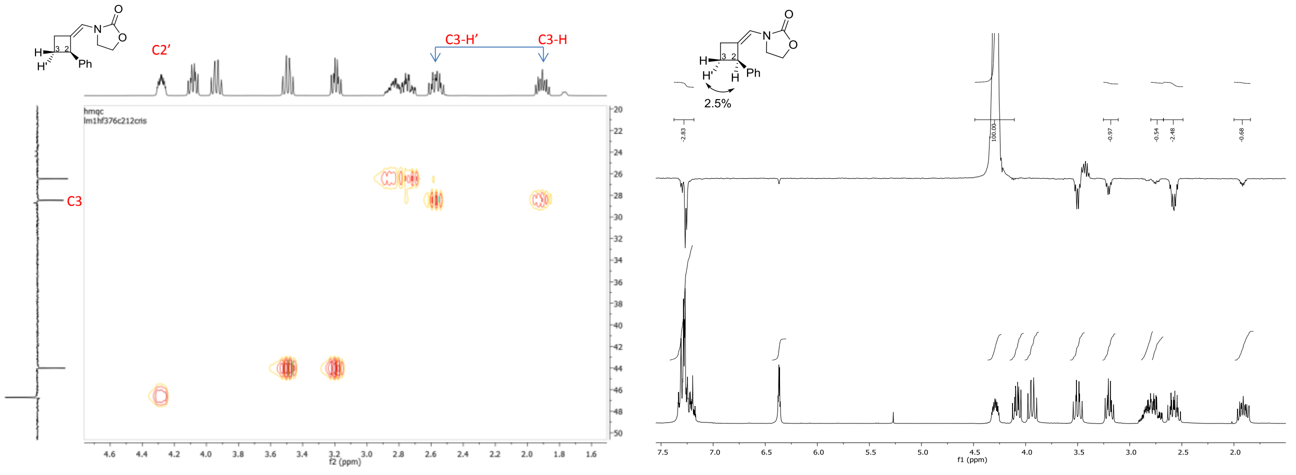

NMR spectra of **4acb** vs *d*-**4acb**:

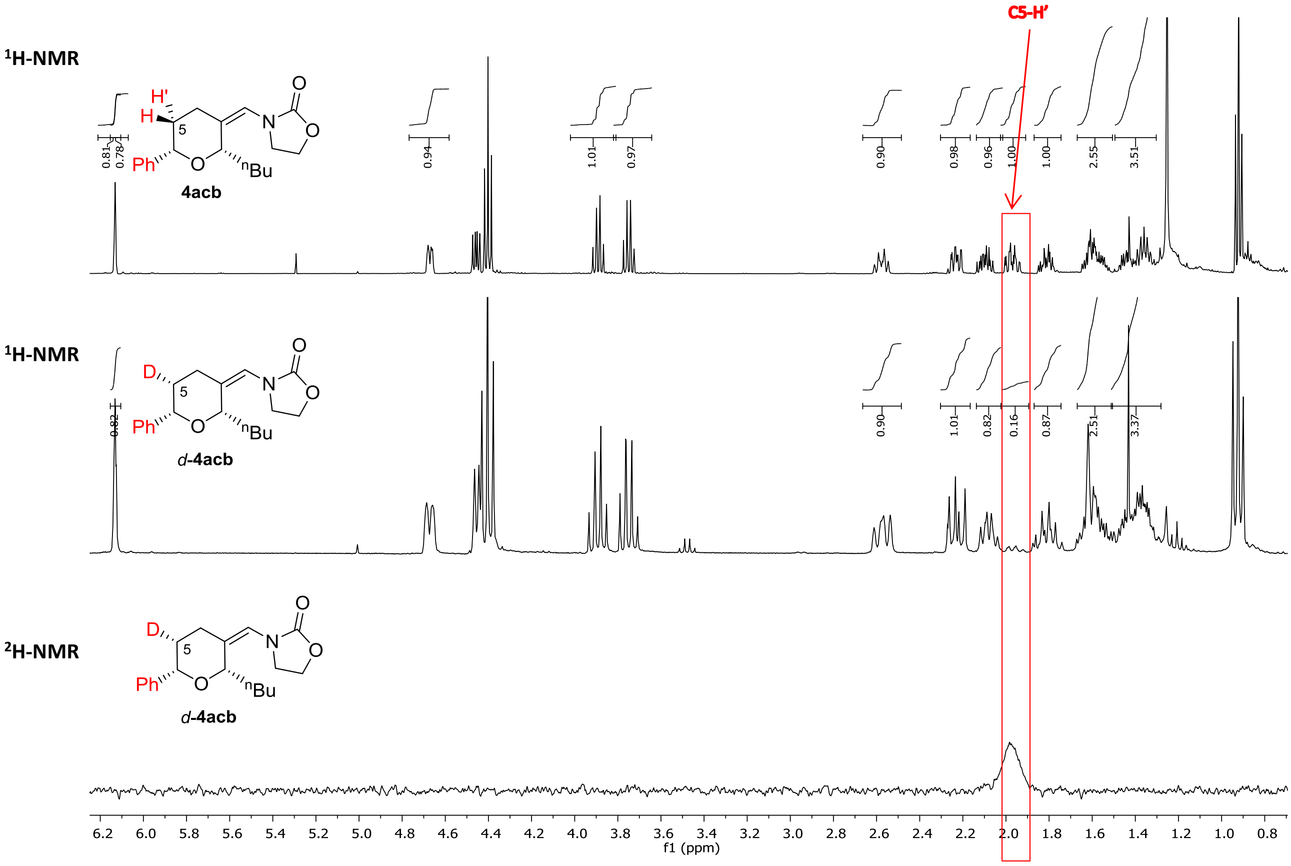

HSQC and nOe spectra for **4acb**:

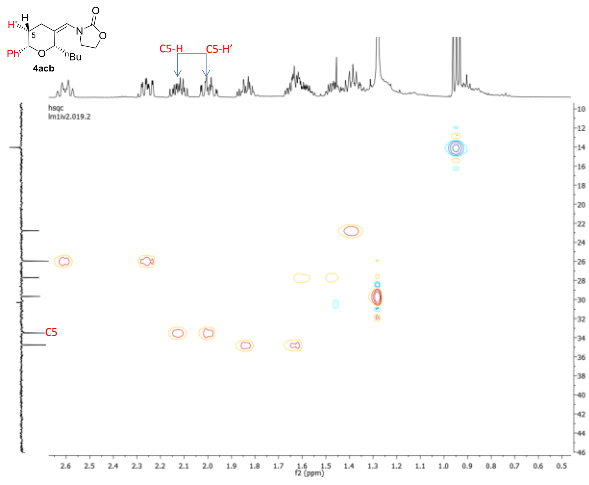

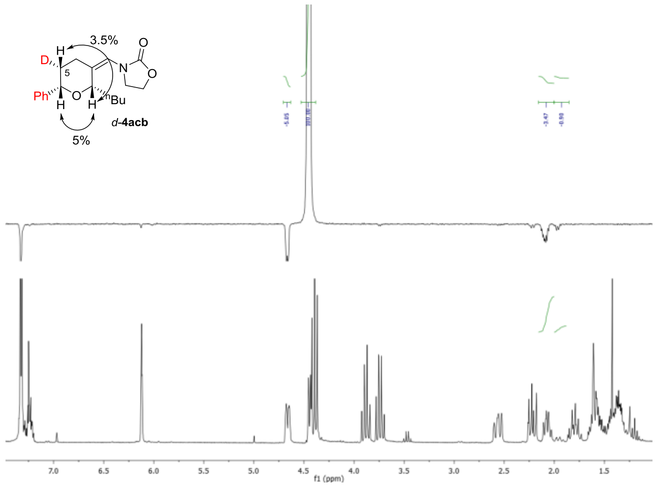

Scheme 5 (main manuscript). [2 + 2 + 2] Cycloaddition of allenamide 1a, pentanal (3b) and deuterated-*E*-2b.[43,44]

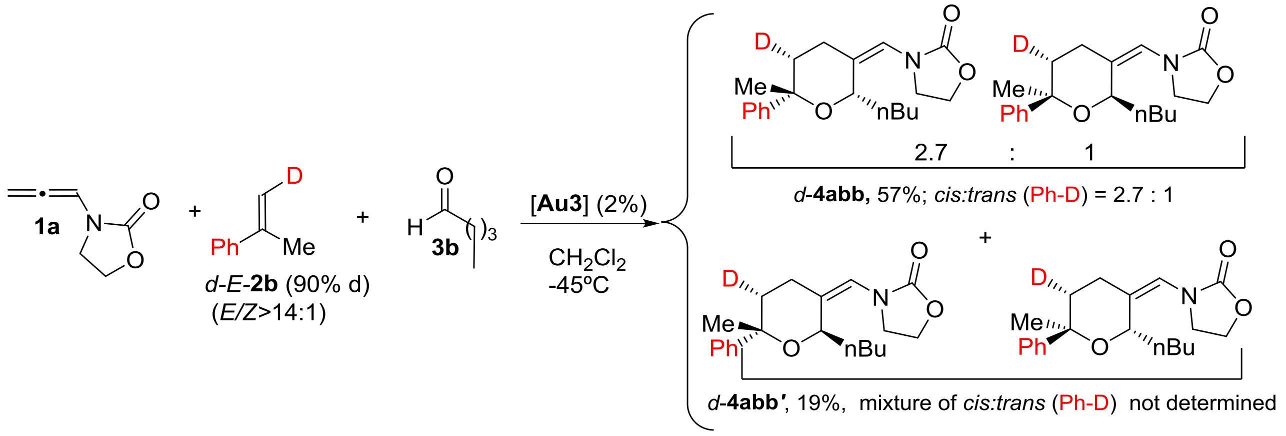

NMR spectra of **2b** vs *d*-E-**2b**

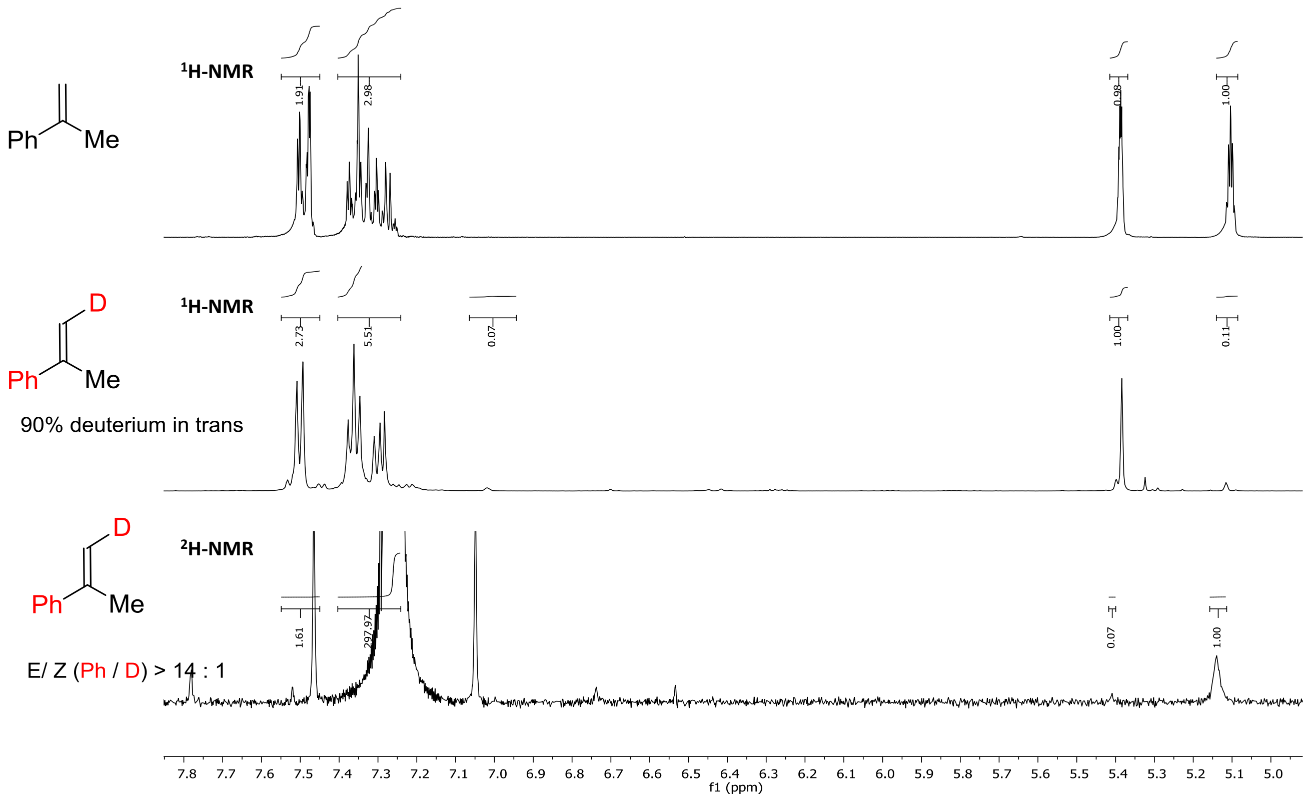

[43] (a) Cycloaddition carried out following the above mentioned general procedure. (b) *d*-**2b** was prepared following a known procedure, using dichloromethane instead of 1,2-dichloroethane, see: E. Negishi, D. E. Van Horn, T. Yoshida, *J. Am. Chem. Soc.* **1985**, *107*, 6639. As can be deduce from the spectra, *d*-**2b** has a 90% content on deuterium (*trans* to the phenyl group) and a *E/Z* ratio >14:1). c) The analog cycloaddition using **2b** instead of *d*-*E*-**2b**, under otherwise identical conditions, is described in the manuscript (Table 2) and provided an equivalent result in terms of yield and diastereoselectivity.

[44] The relative position of deuterium with respect to the phenyl group in **4abb'** could not be determined because of overlapping of C5-H and C5-H' ^{1}H-NMR signals.

NMR spectra of **4abb** vs *d*-**4abb**

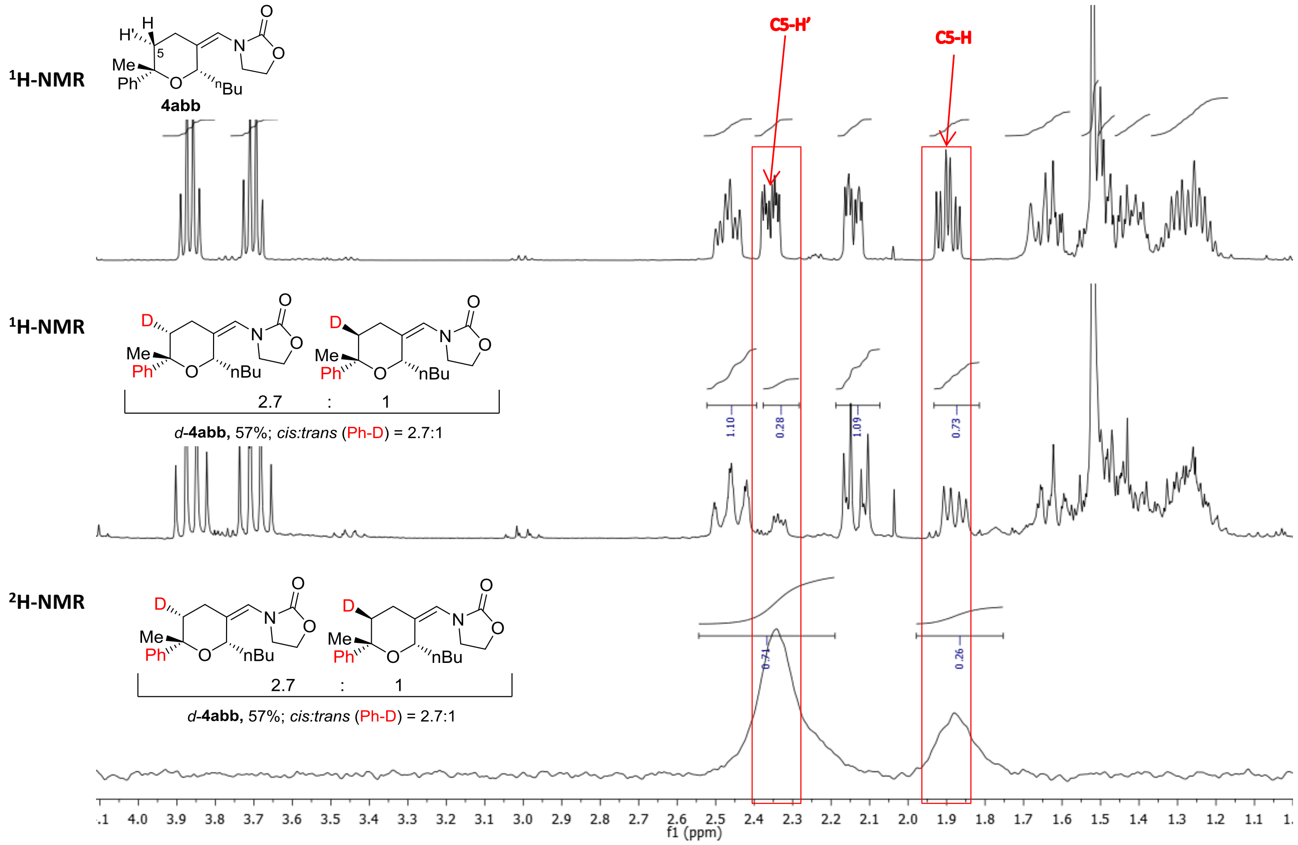

HSQC and nOe spectra for **4abb**

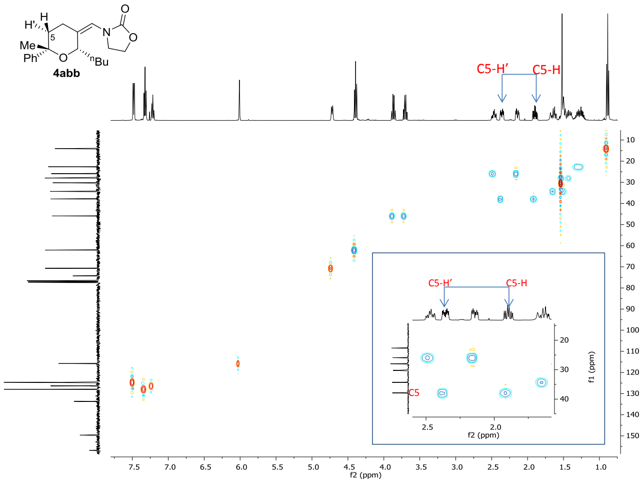

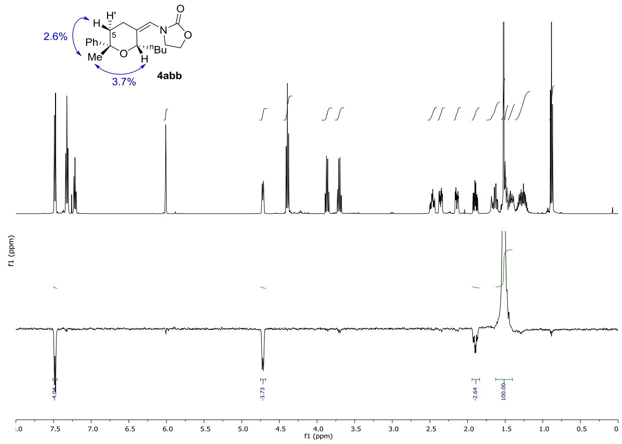

Scheme of reference 19 (main manuscript). [2 + 2 + 2] Cycloaddition of allenamide 1a, benzaldehyde (3a) and deuterated-*E*-2e.[45]

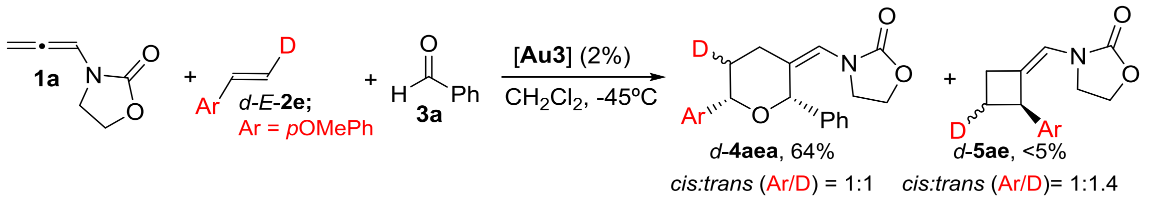

NMR spectra of **2e** vs *d*-*E*-**2e**

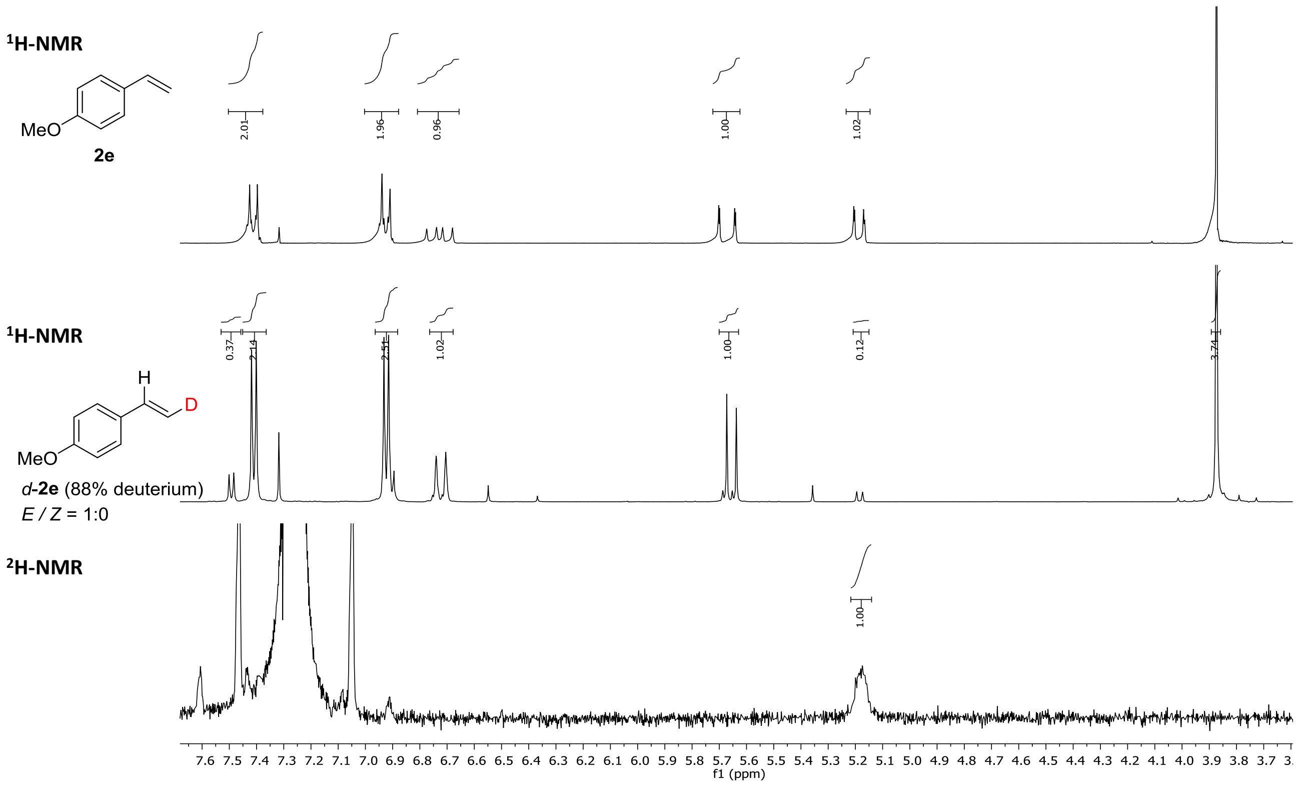

---

[45] (a) Cycloaddition carried out following the abovementioned general procedure. (b) *d*-**2e** was prepared following the procedure described in: L. T. Ball, G. C. Lloyd-Jones, C. A. Russell, *Chem. Eur. J.* **2012**, *18*, 2931. See also: Gao, F.; Hoveyda, A. H. *J. Am. Chem. Soc*. **2010**, *132* 10961. (b) As can be deduce from the spectra, *d*-**2e** has a 88% content on deuterium (*trans* to the aryl group) and a *E*/*Z* ratio 1:0). c) The analog cycloaddition using **2e** instead of *d*-*E*-**2e**, under otherwise identical conditions, is described in the manuscript (Table 2) and provided an equivalent result in terms of yields, chemoselectivity and diastereoselectivity.

NMR spectra of **5ae** vs *d*-**5ae**

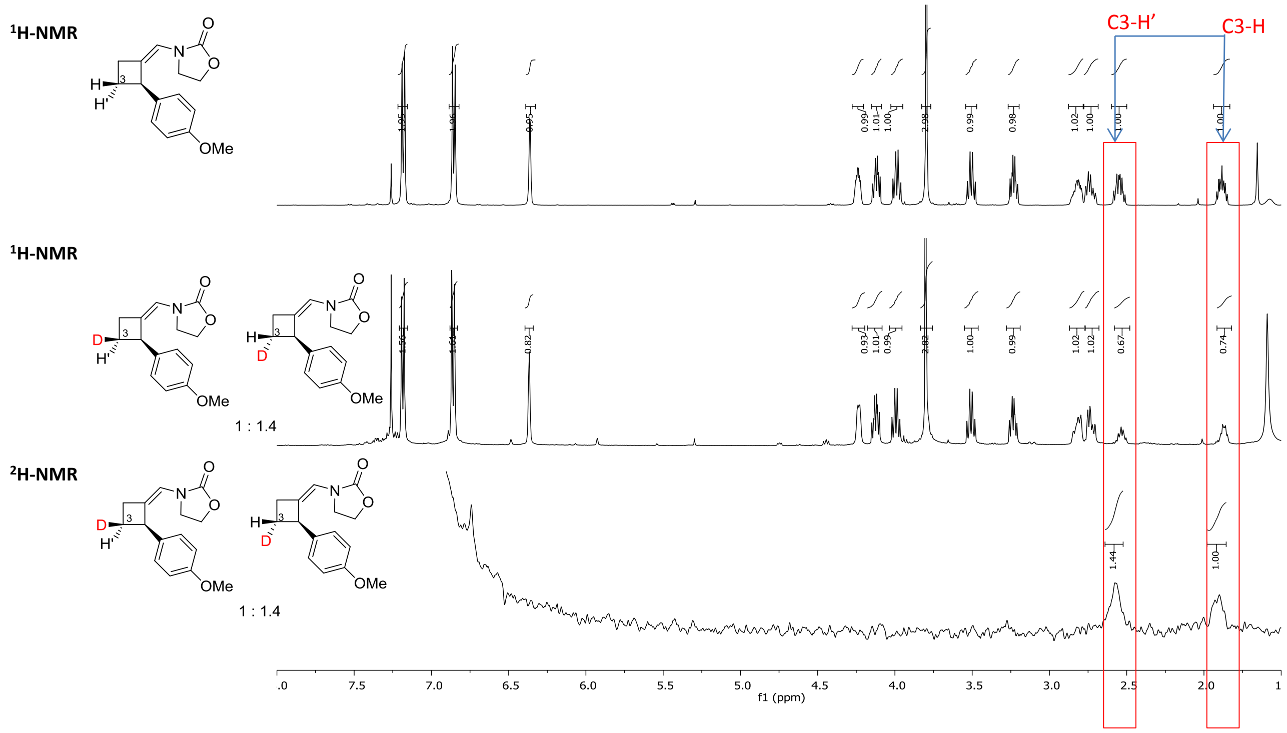

HSQC and nOe spectra for **5ae**

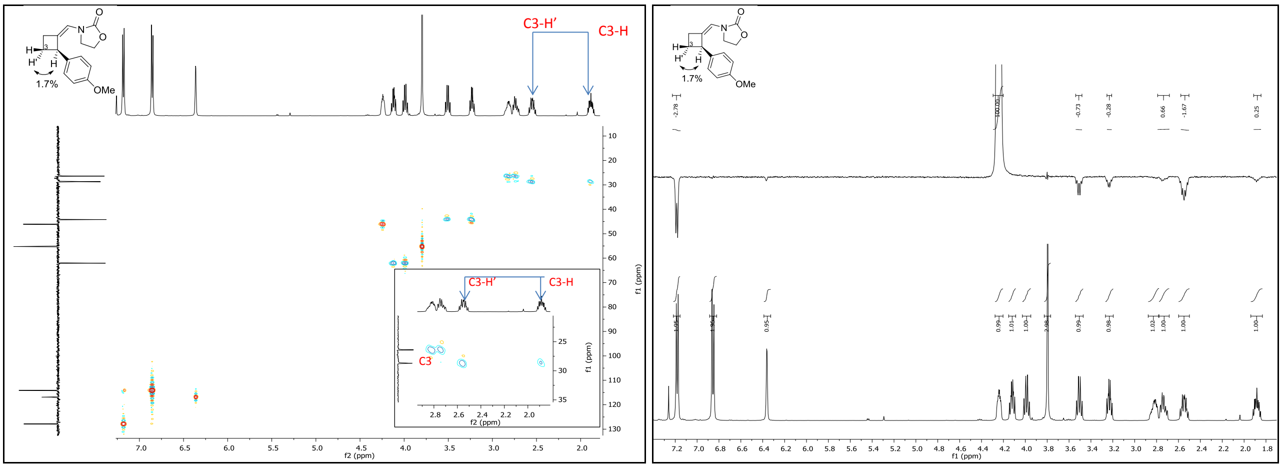

NMR spectra of **4aea** vs *d*-**4aea**

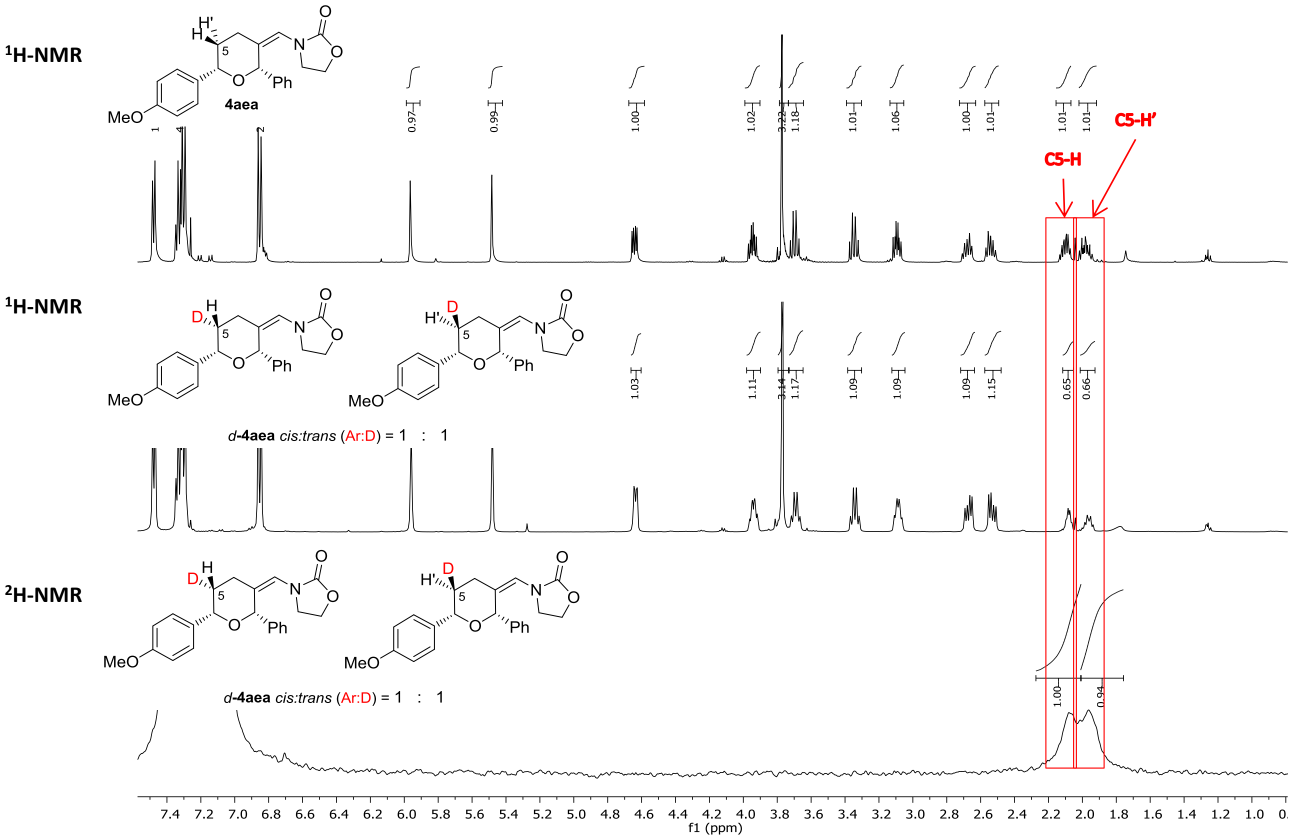

HSQC and nOe spectra for **4aea**

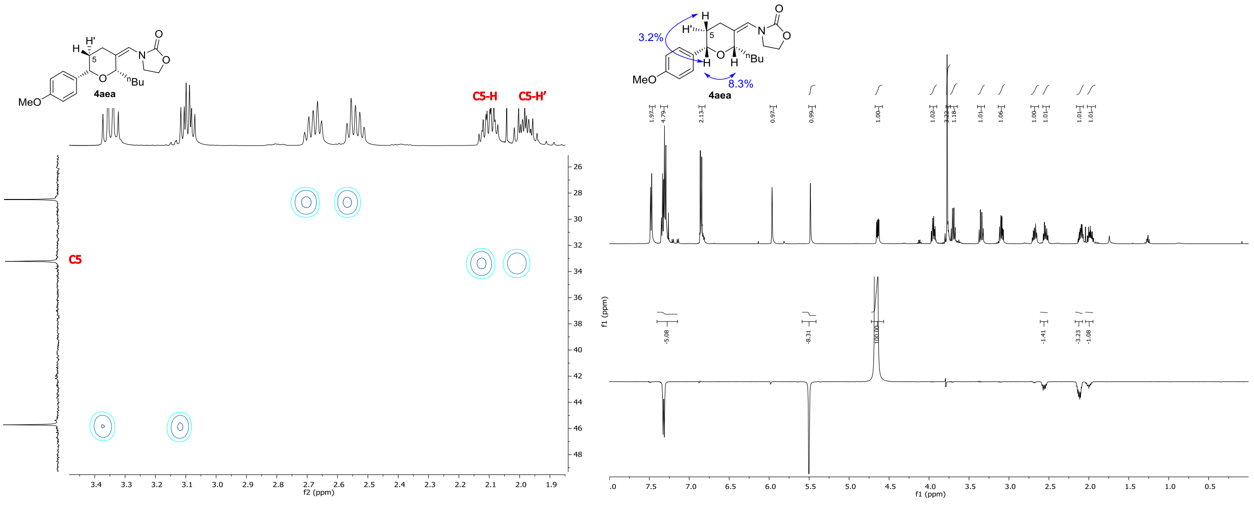

Cycloaddition of *d*-*E*-2c with the NHC-gold catalyst Au2.

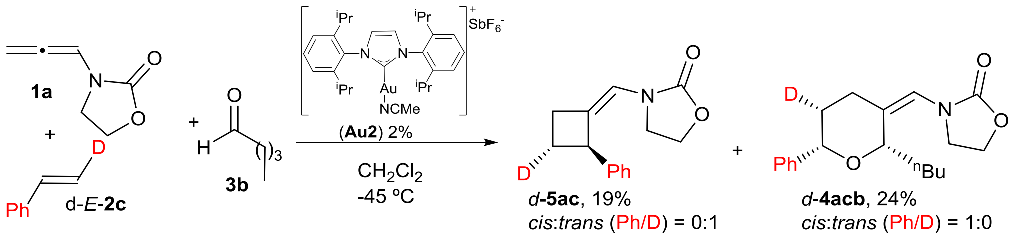

(1)

NMR spectra of **5ac** vs *d*-**5ac** (obtained with **Au2**):

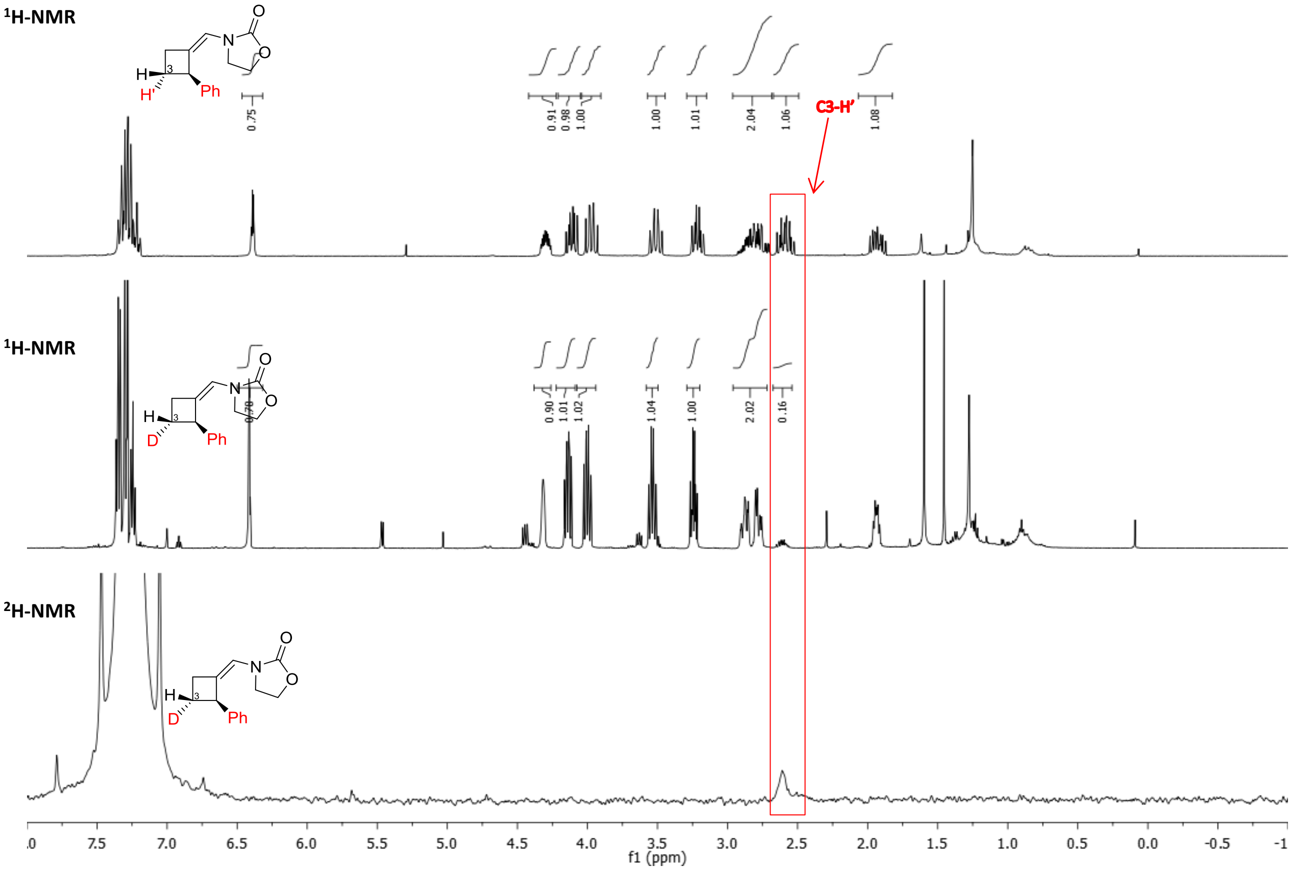

NMR spectra of **4acb** vs *d*-**4acb** (obtained with **Au2**):

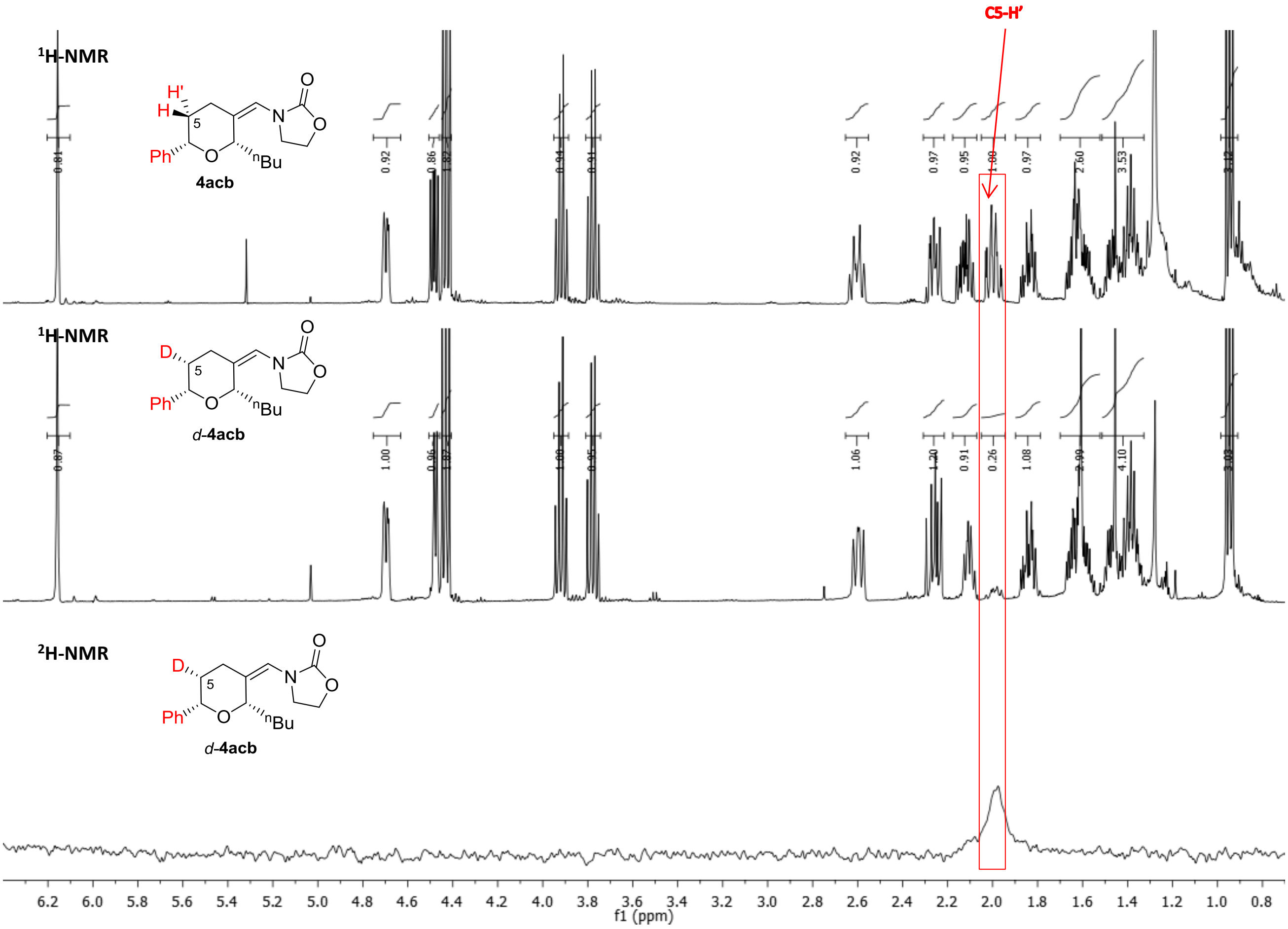

Cycloaddition of *d*-*E*-2b with the NHC-gold catalyst Au2.

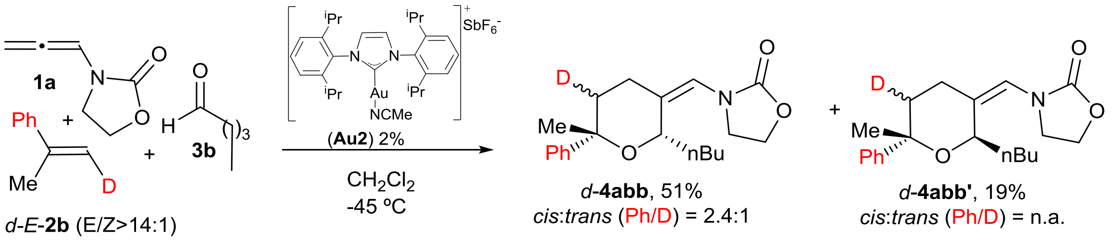

NMR spectra of **4abb** vs *d*-**4abb** (obtained with **Au2**):

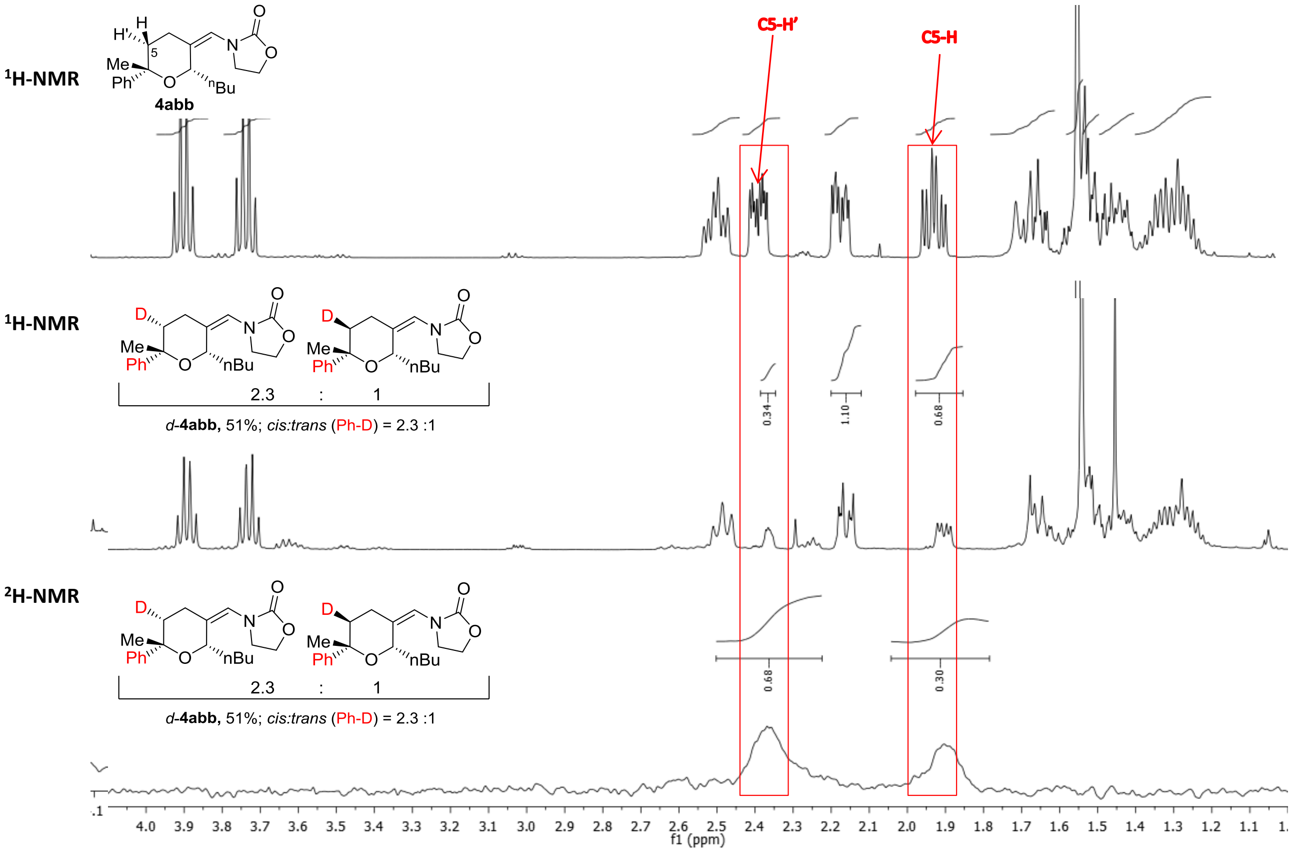

Cycloaddition of *d*-*E*-2e with the NHC-gold catalyst Au2.[46]

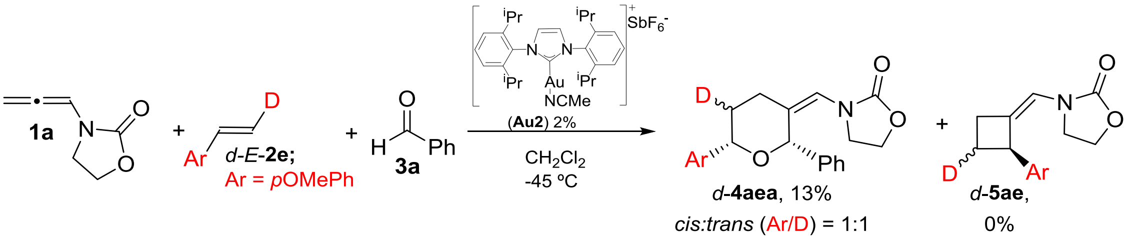

NMR spectra of **4aea** vs *d*-**4aea** (obtained with **Au2**)

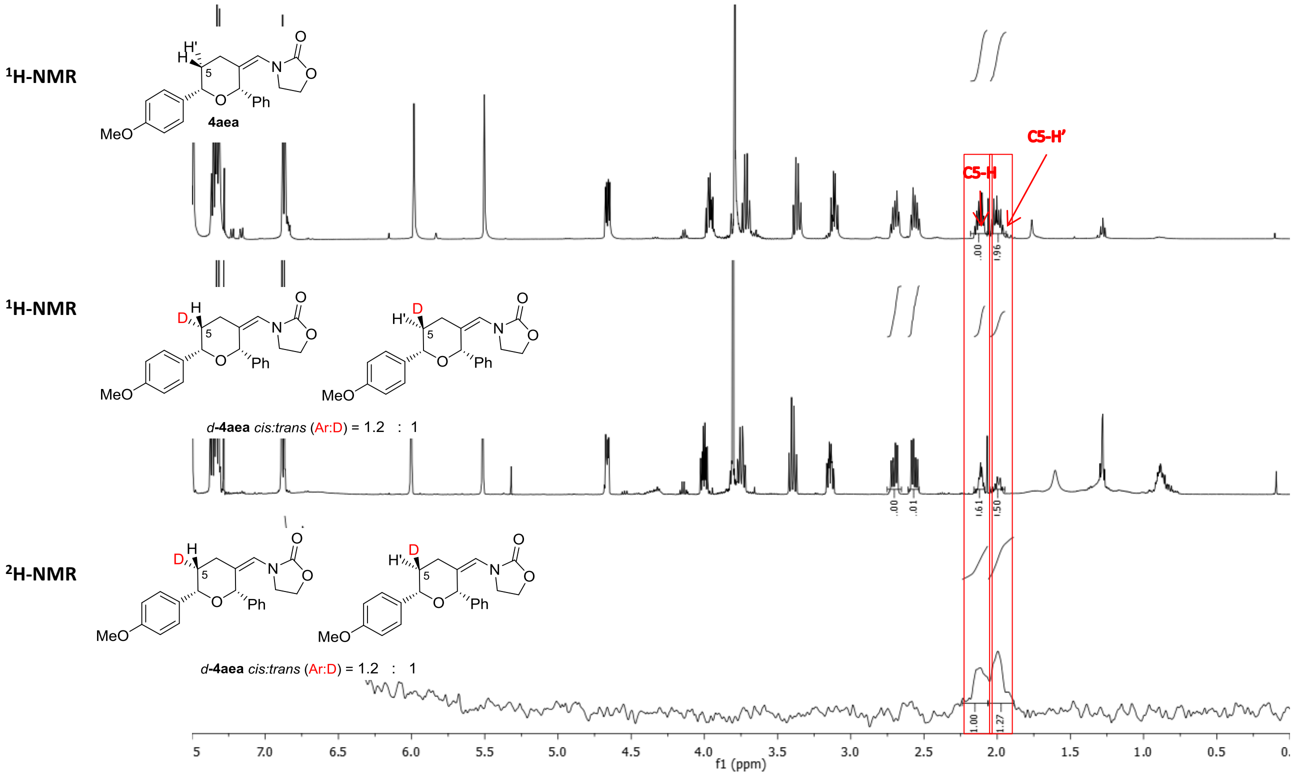

[46] The reaction was not efficient. We could only observed in the crude mixture the [2+2+2] cycloadduct *d*-**4aea**. , which was eventually isolated in a 13% yield. The [2+2] cycloadduct *d*-**5ae** was not observed. However, this adduct can be prepared independently by the **Au2**-catalyzed reaction between allenamide **1a** and *d*-*E*-**2e**. As with **Au3**, the obtained adduct showed a Ar / D *cis* : *trans* ratio of 1: 1.6.

Brief summary of other attemps to optimize the reaction between allenamide (1a), β-methyl styrene (2a) and benzaldehyde (3a)

Table S1. Preliminary evaluation of the [2 + 2 + 2] cycloaddition.[a,b]

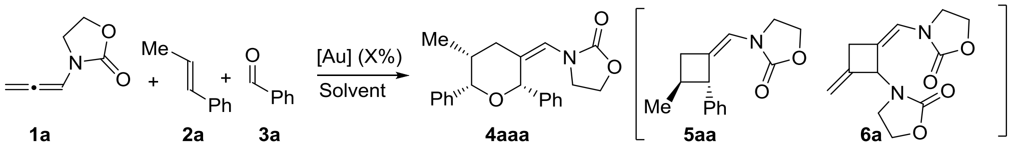

entry	[Au] (mol %)	Solvent	*t* (ºC)	*t* (h)	Conv.	**4** (%)	**5** (%)	**6** (%)
1	**Au1** (5%)	CH_2Cl_2	-15	5	99%	**4aaa**, 2	**5aa**, 4	**6a**, 44
2	$Ph_3PAuNTf_2$ (5%)	CH_2Cl_2	-15	24	60%	**4aaa**, 2	**5aa**, 0	**6a**, 7
3	**Au2** (5%)	CH_2Cl_2	-15	0.2	99%	**4aaa**, 15	**5aa**, 7	**6a**, 22
4	**Au3** (2%)	CH_2Cl_2	-15	3	99%	**4aaa**, 21	**5aa**, 60	**6a**, 8
5	**Au3'** (2 %)	CH_2Cl_2	-15	3	99%	**4aaa**, 15	**5aa**, 30	**6a**, 4
6	**Au3** (2%)	CH_2Cl_2	-45	1	99%	**4aaa**, 35	**5aa**, 37	**6a**, 0
7	**Au3** (2%)	CH_2Cl_2	-78	20	99%	**4aaa**, 28	**5aa**, 3o	**6a**, 0
8	**Au3** (5%)	$MeNO_2$	-15	0.5	99%	**4aaa**, 16	**5aa**, 30	**6a**, 8
9	**Au3** (5%)	Toluene	-15	0.5	99%	**4aaa**, 0	**5aa**, 0	**6a**, 20
10	**Au3** (5%)	TFT[c]	-15	0.5	94%	**4aaa**, 19	**5aa**, 20	**6a**, 14

[a] **1a** (1 equiv) added over 2 h to a solution of **2** (2 equiv), benzaldehyde (**3a**,10 equiv), [Au] (mol%) and 4Å MS, in CH_2Cl_2 at the indicated temperature, unless otherwise noted. [b] Conversion of **1a** and yields of **4-6** determined by ^{1}H-NMR of the crude mixture using 1,3,5-$(MeO)_3C_6H_3$ as internal standard (IS) [c] TFT stands for F_3C-C_6H_5.

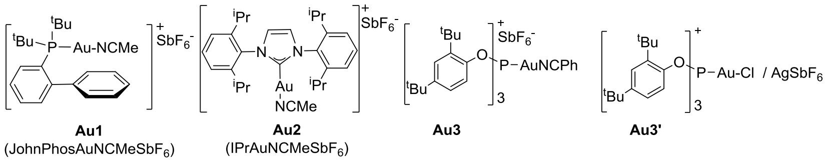

NMR SPECTRA

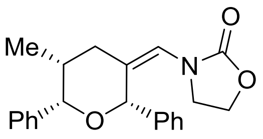

4aaa

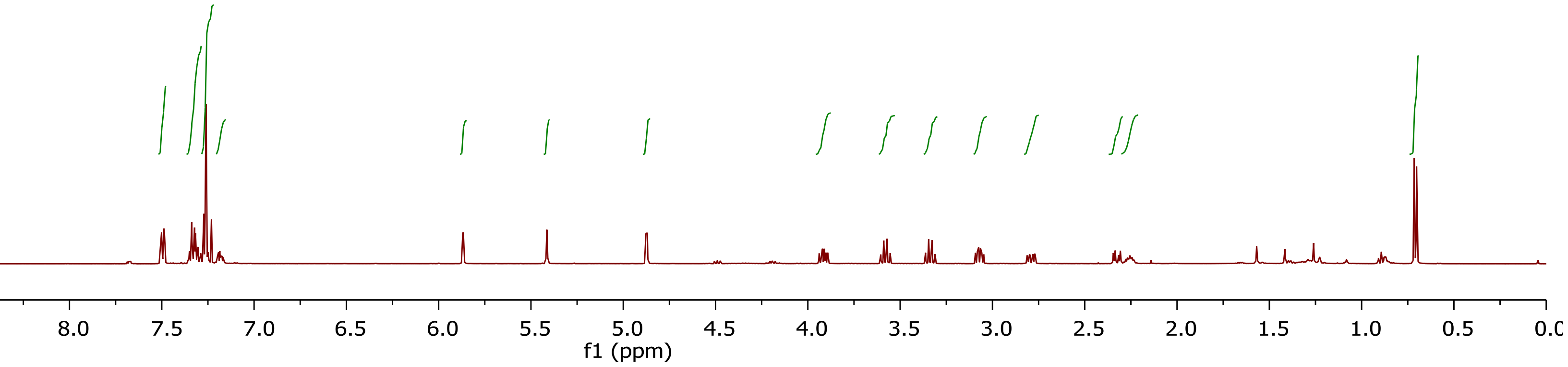

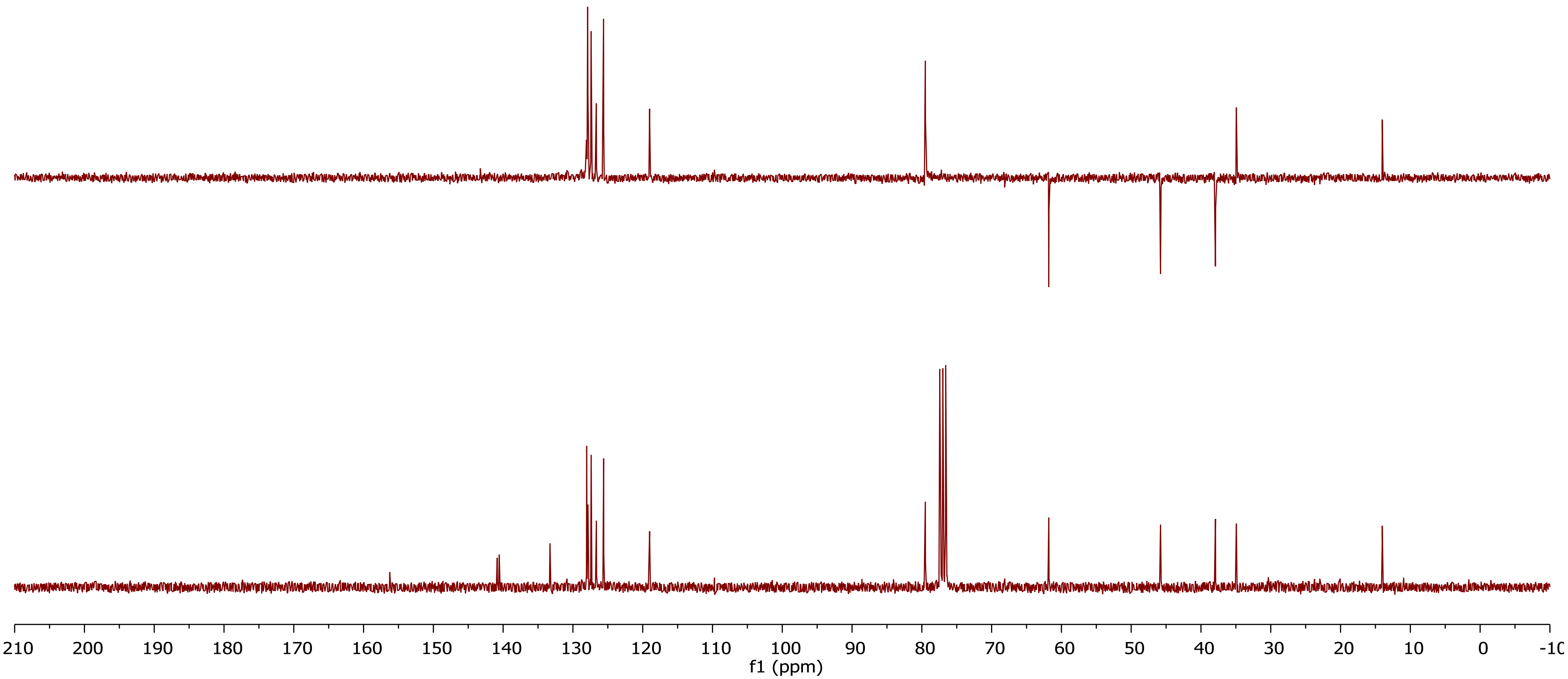

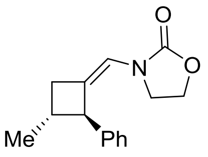
O
N
O
Me
Ph

5aa

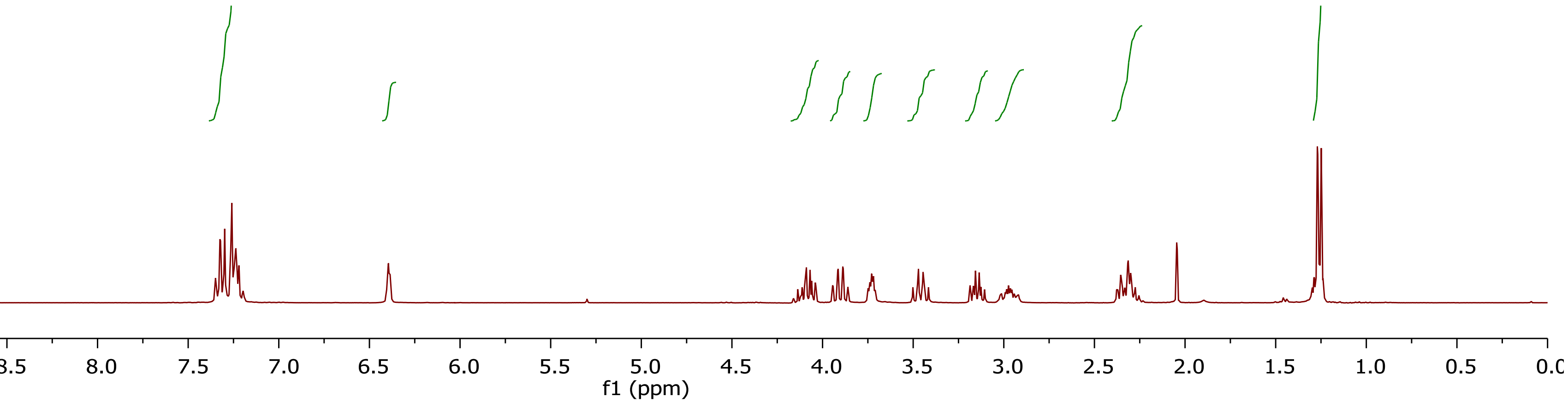
10.0 9.5 9.0 8.5 8.0 7.5 7.0 6.5 6.0 5.5 5.0 4.5 4.0 3.5 3.0 2.5 2.0 1.5 1.0 0.5
f1 (ppm)

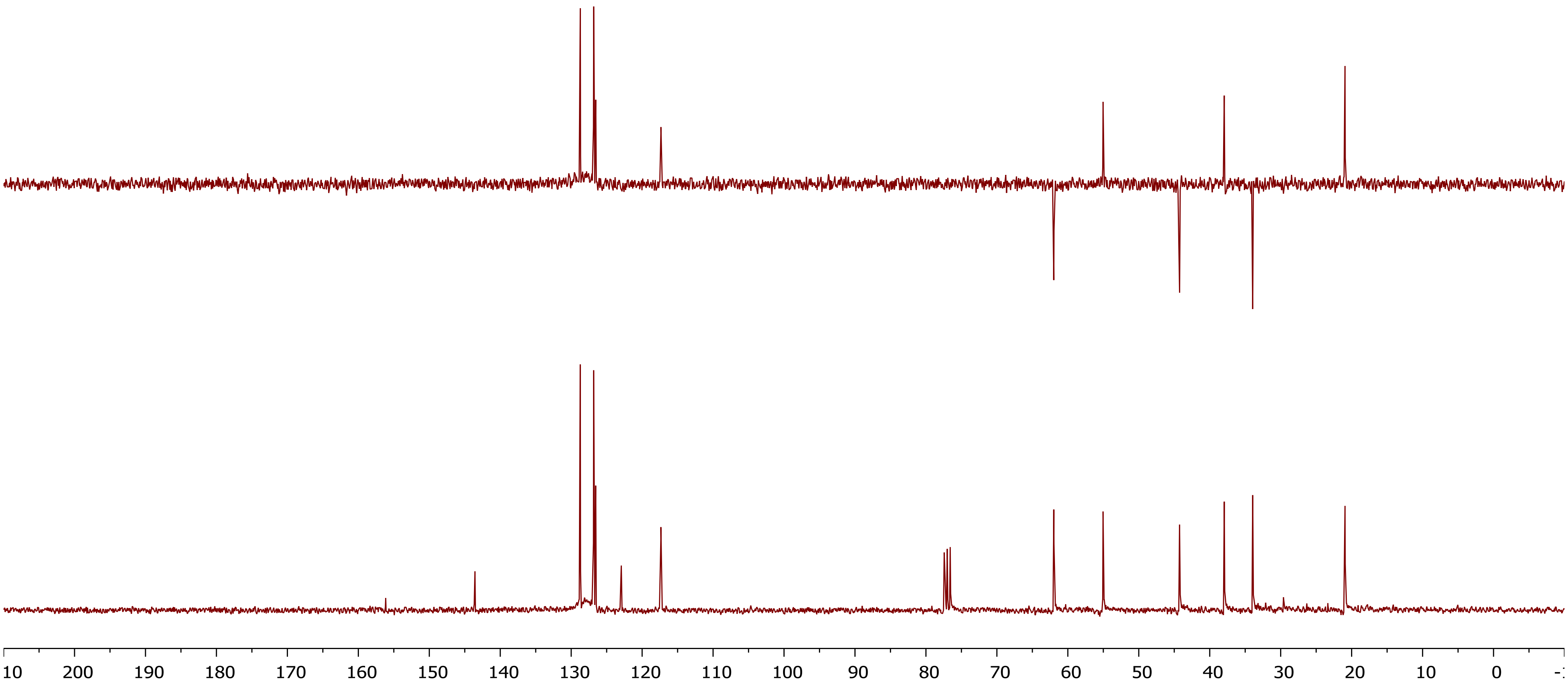
200 190 180 170 160 150 140 130 120 110 100 90 80 70 60 50 40 30 20 10 0
f1 (ppm)

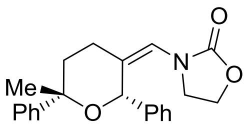
Me
Ph
O
Ph
N
O
O

4aba

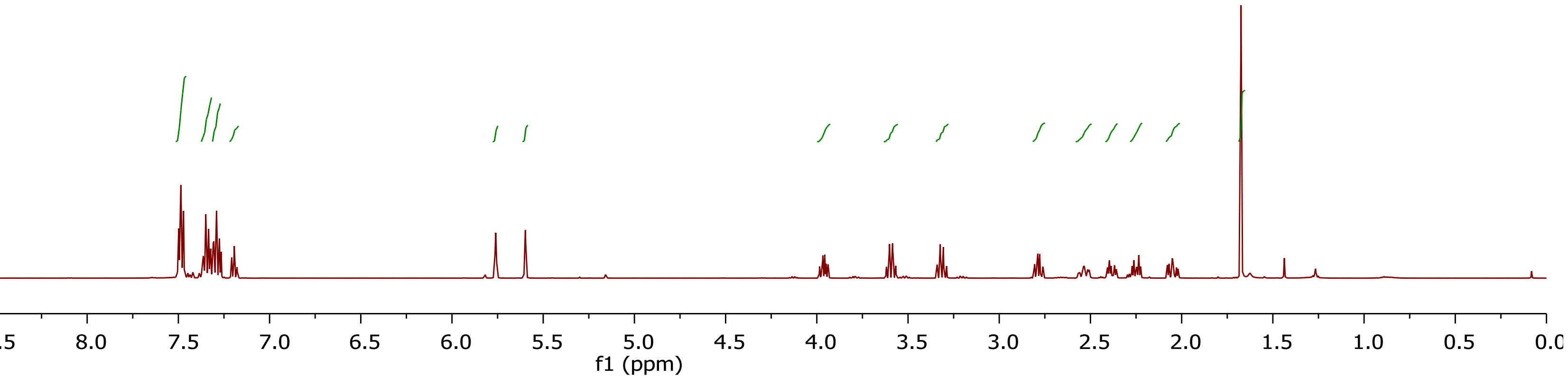
9.5 9.0 8.5 8.0 7.5 7.0 6.5 6.0 5.5 5.0 4.5 4.0 3.5 3.0 2.5 2.0 1.5 1.0 0.5 0.0
f1 (ppm)

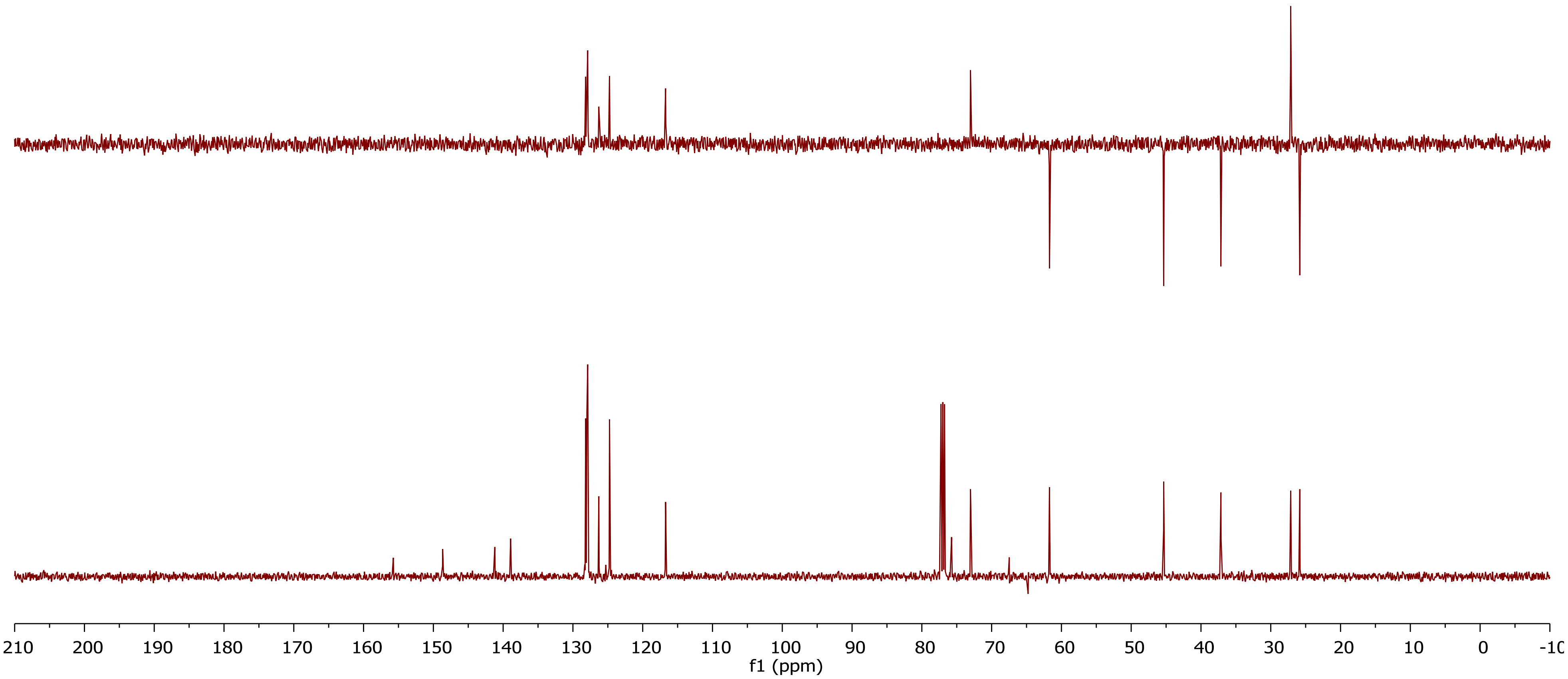
210 200 190 180 170 160 150 140 130 120 110 100 90 80 70 60 50 40 30 20 10 0 -10
f1 (ppm)

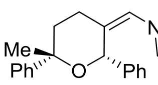

4aba

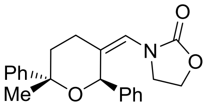

4aba'

1:1 mixture of **4aba** and **4aba'**

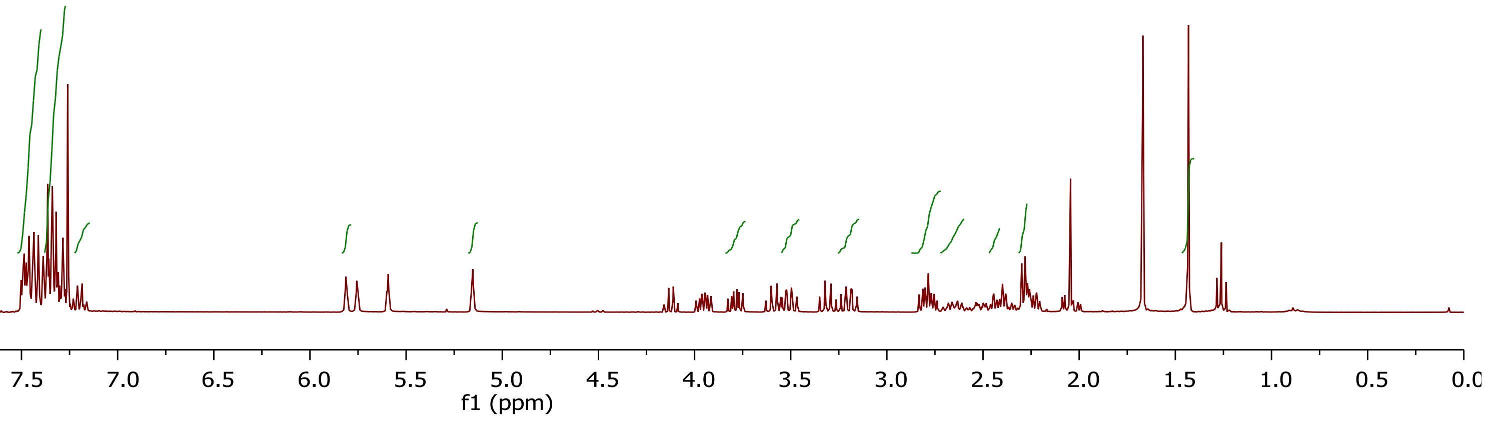

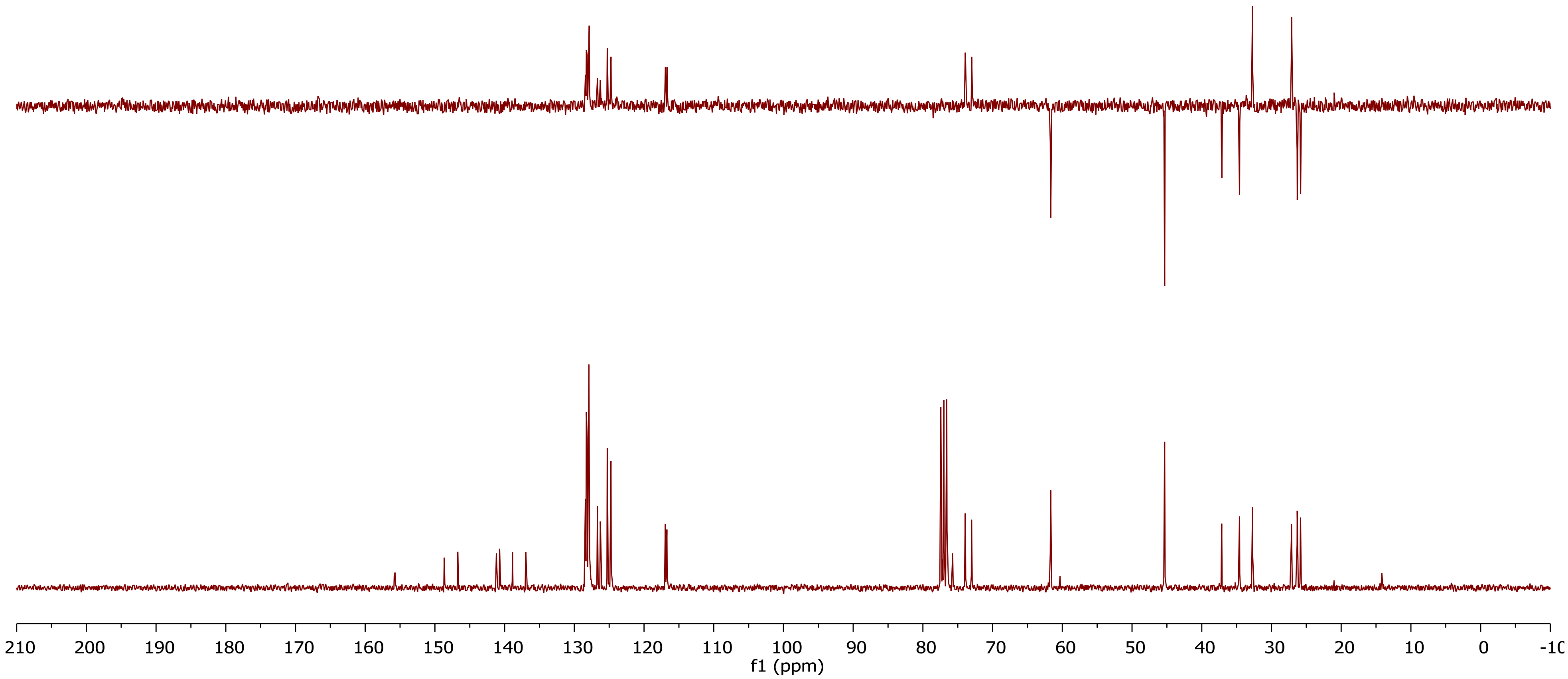

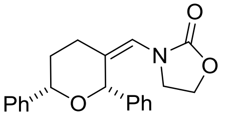

4aca

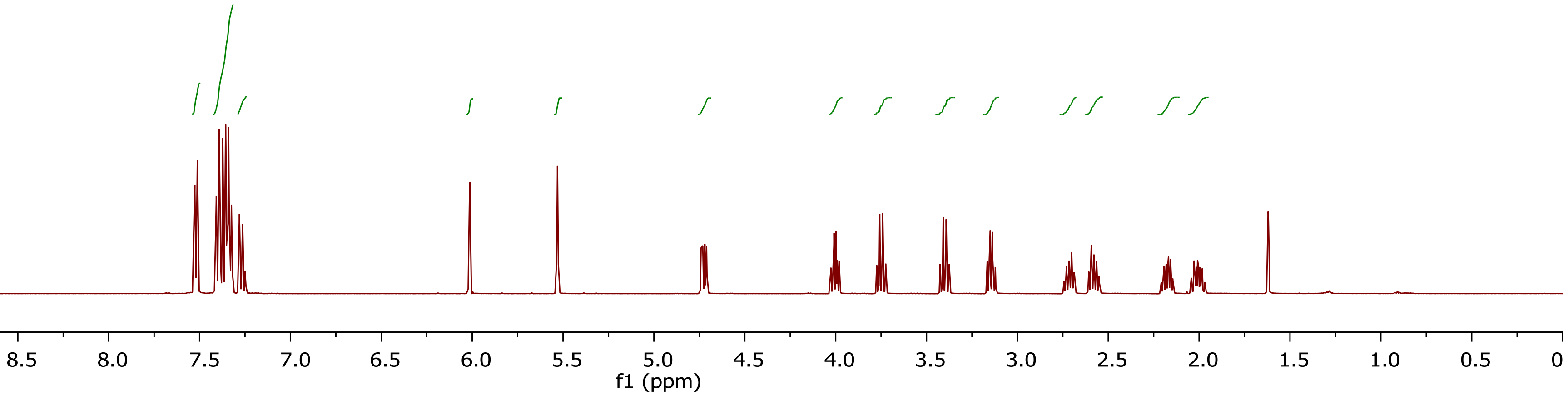

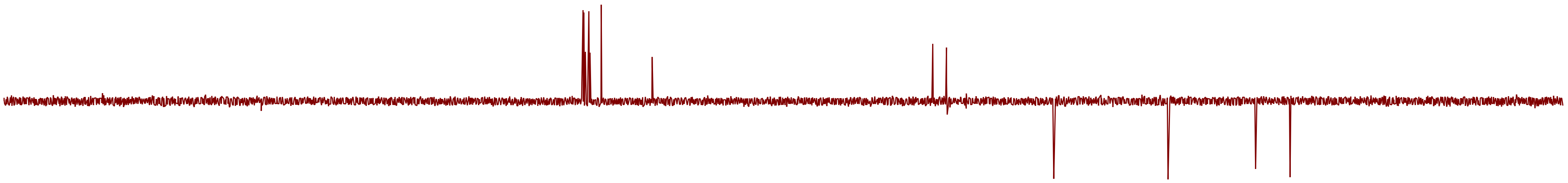

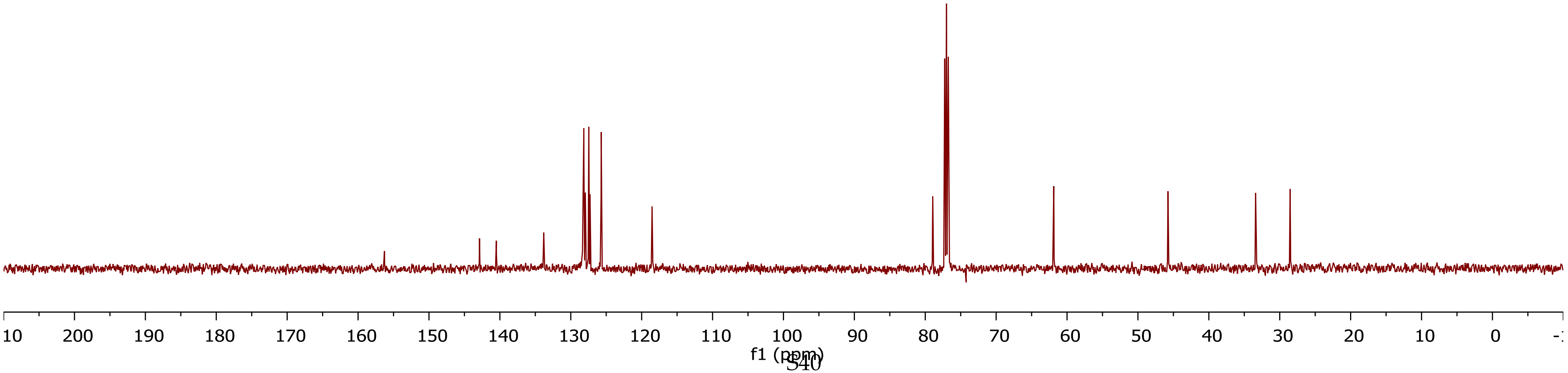

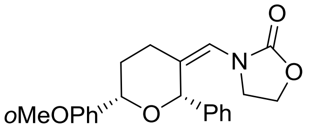

4ada

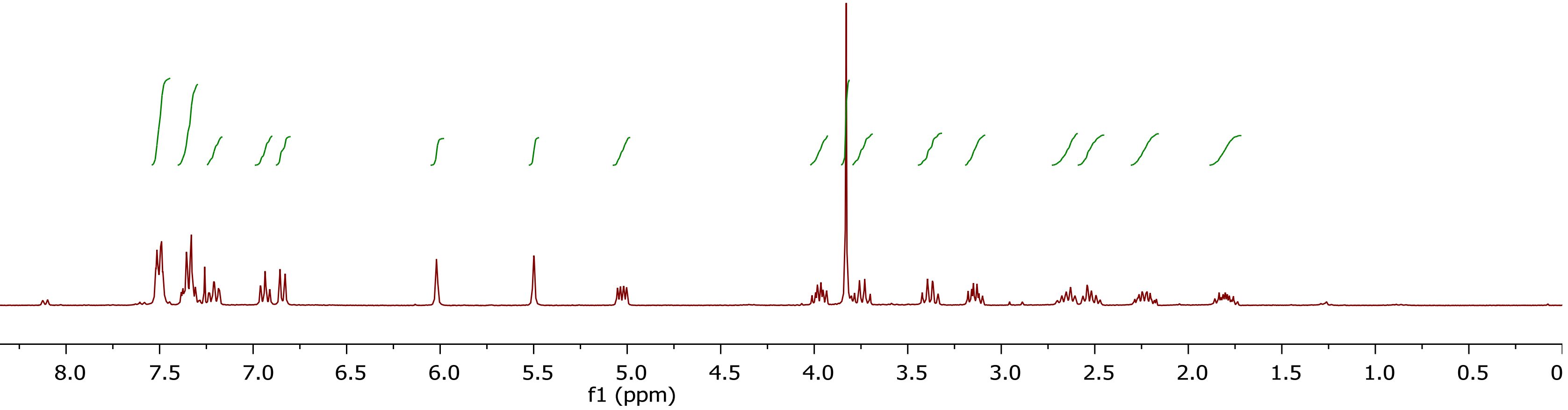

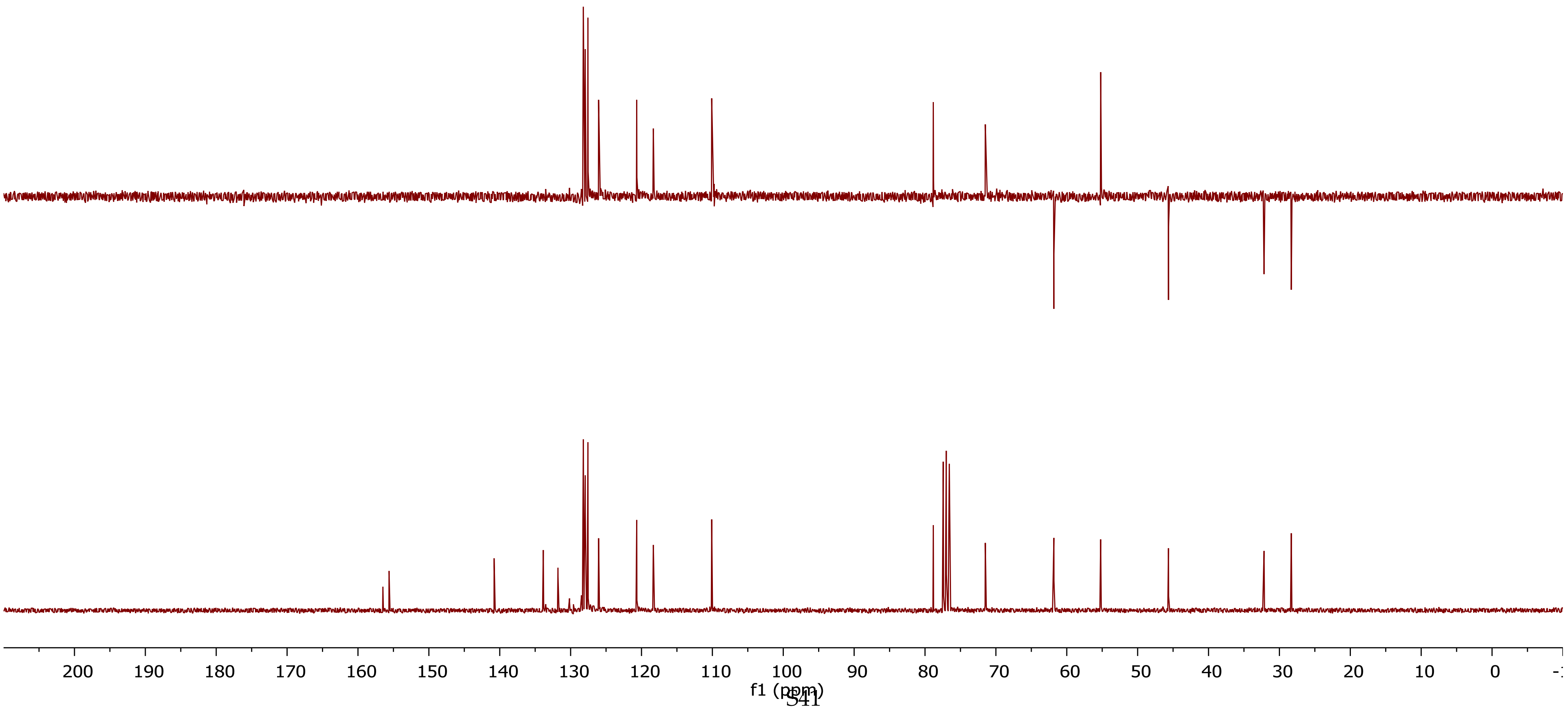

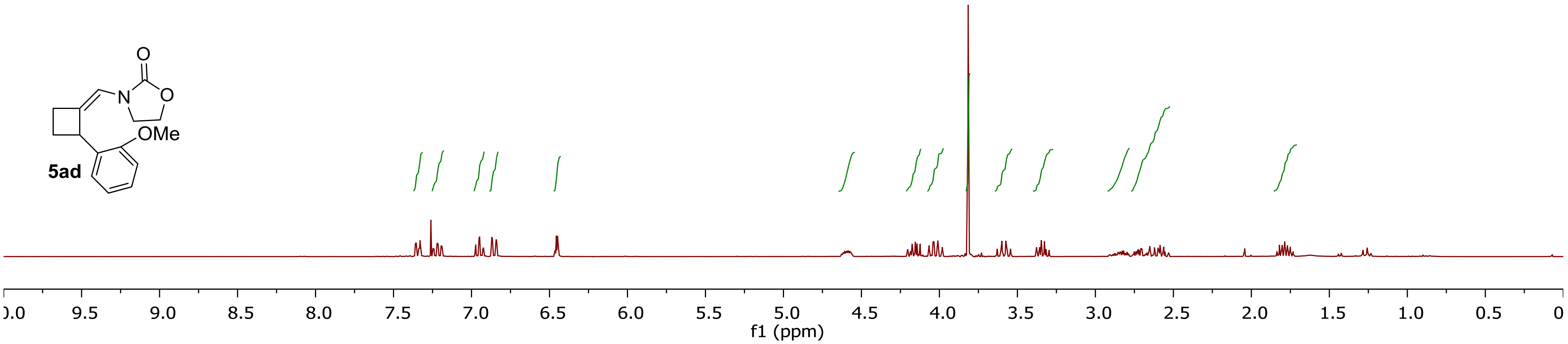
O
N
O
OMe
5ad
f1 (ppm)

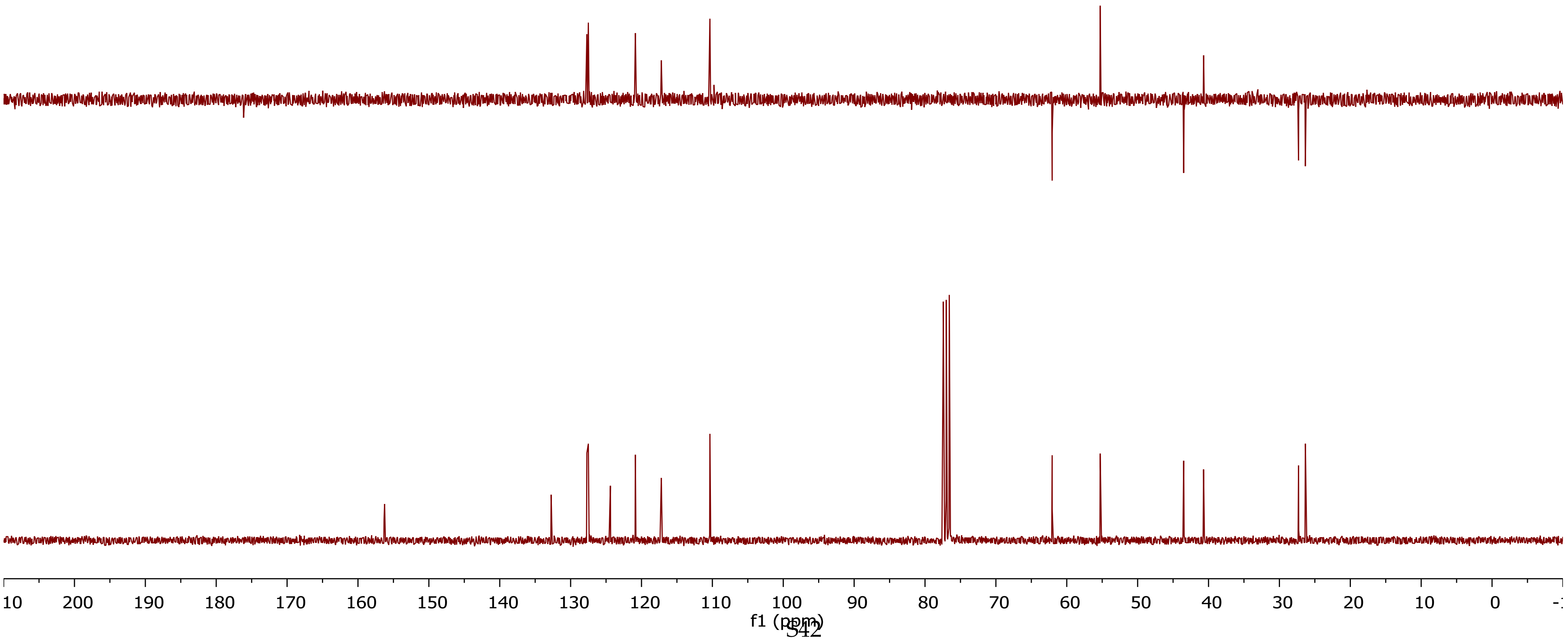
f1 (ppm)

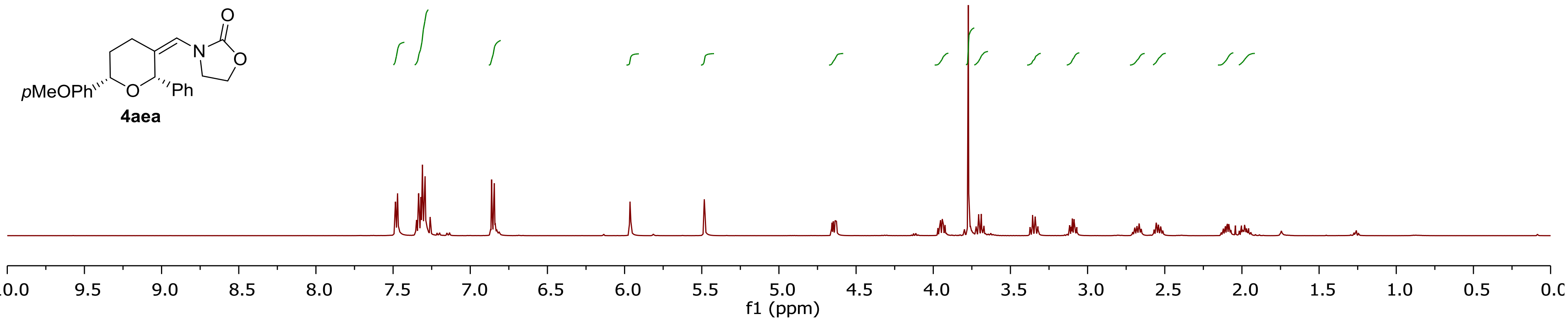
O
N
O
pMeOPh
O
Ph
4aea
10.0 9.5 9.0 8.5 8.0 7.5 7.0 6.5 6.0 5.5 5.0 4.5 4.0 3.5 3.0 2.5 2.0 1.5 1.0 0.5 0.0
f1 (ppm)

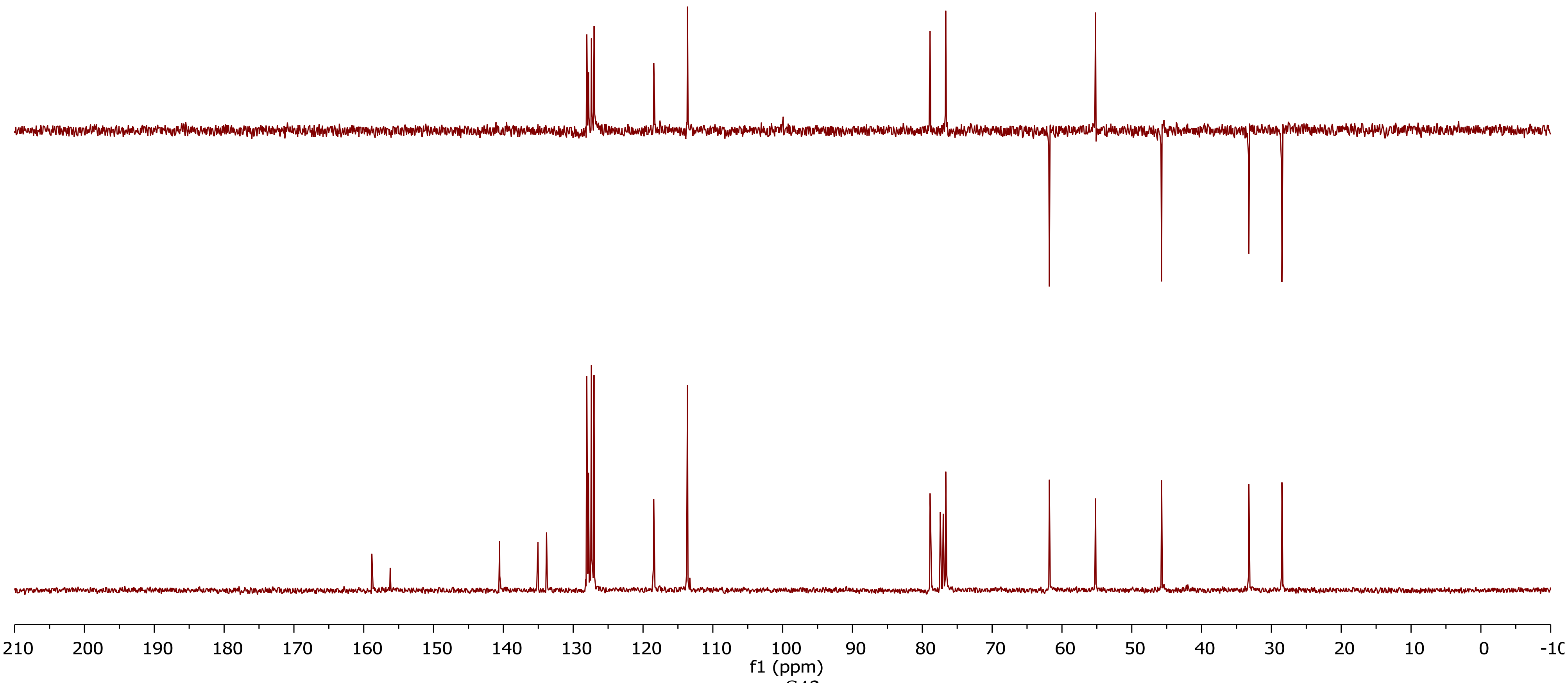
210 200 190 180 170 160 150 140 130 120 110 100 90 80 70 60 50 40 30 20 10 0 -10
f1 (ppm)

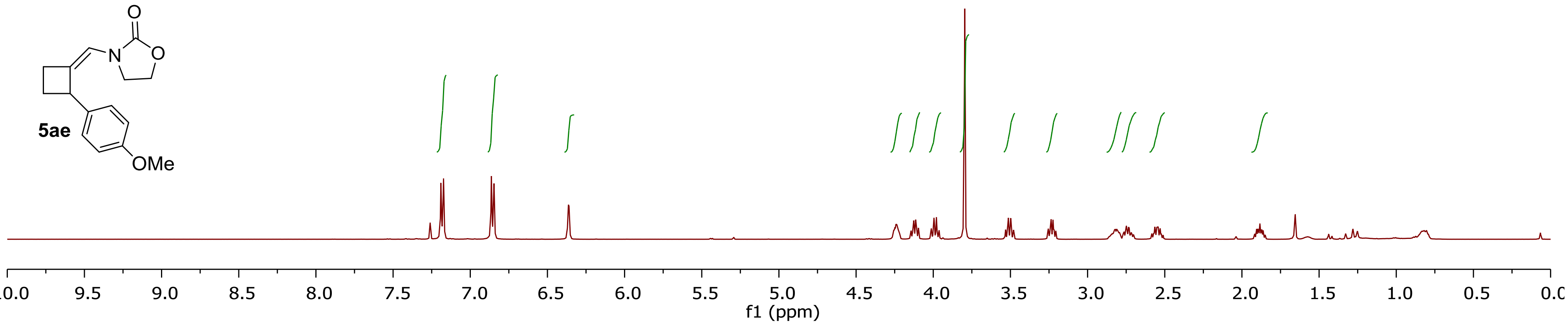
O
N
O
5ae
OMe
10.0 9.5 9.0 8.5 8.0 7.5 7.0 6.5 6.0 5.5 5.0 4.5 4.0 3.5 3.0 2.5 2.0 1.5 1.0 0.5 0.0
f1 (ppm)

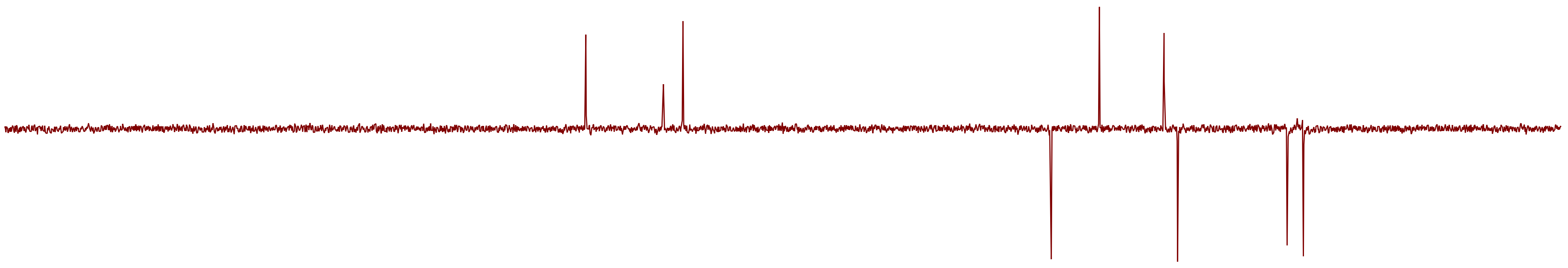

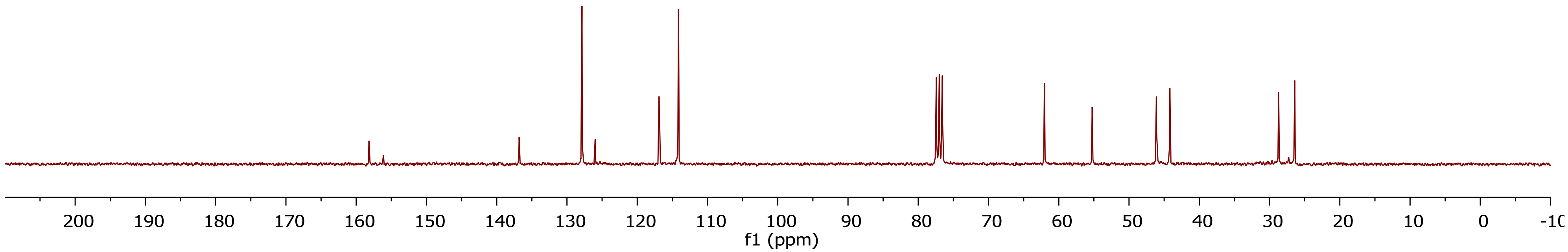
200 190 180 170 160 150 140 130 120 110 100 90 80 70 60 50 40 30 20 10 0 -10
f1 (ppm)

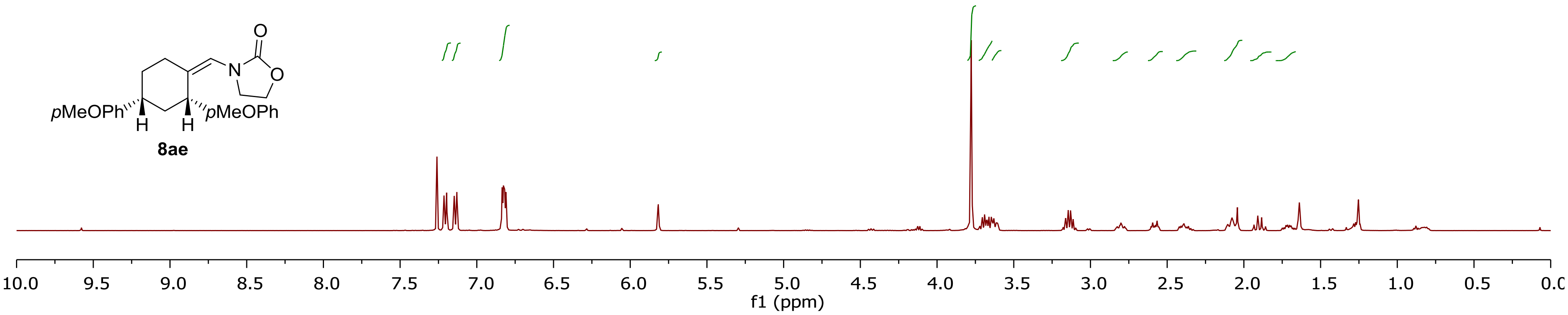
O
N
O
pMeOPh
H
H
pMeOPh
8ae
10.0 9.5 9.0 8.5 8.0 7.5 7.0 6.5 6.0 5.5 5.0 4.5 4.0 3.5 3.0 2.5 2.0 1.5 1.0 0.5
f1 (ppm)

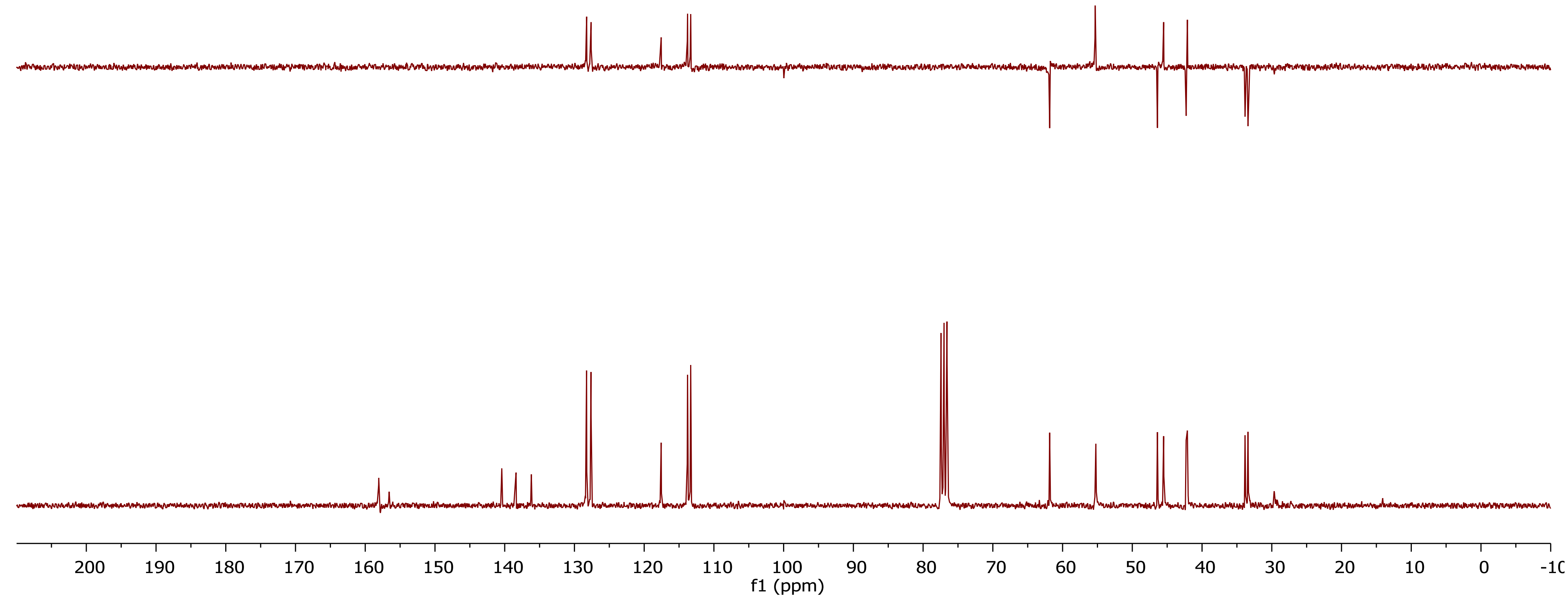
200 190 180 170 160 150 140 130 120 110 100 90 80 70 60 50 40 30 20 10 0
f1 (ppm)

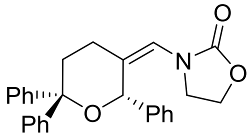

4afa

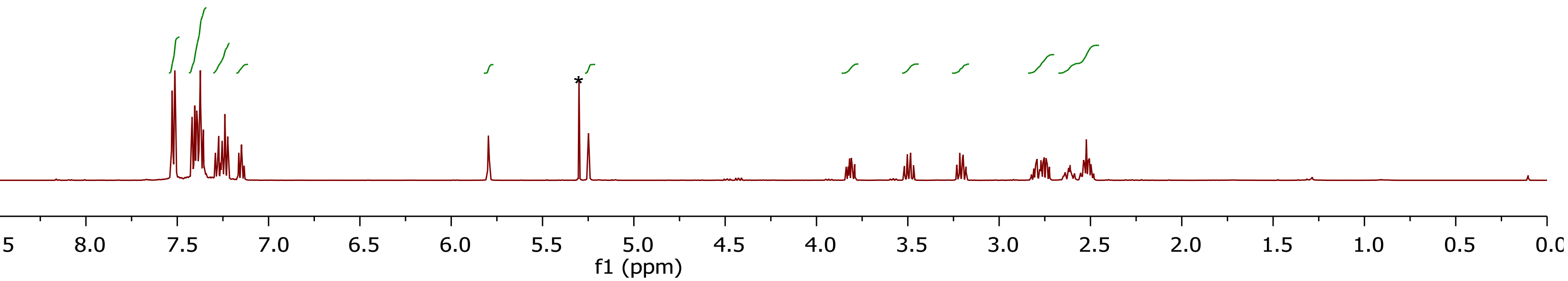

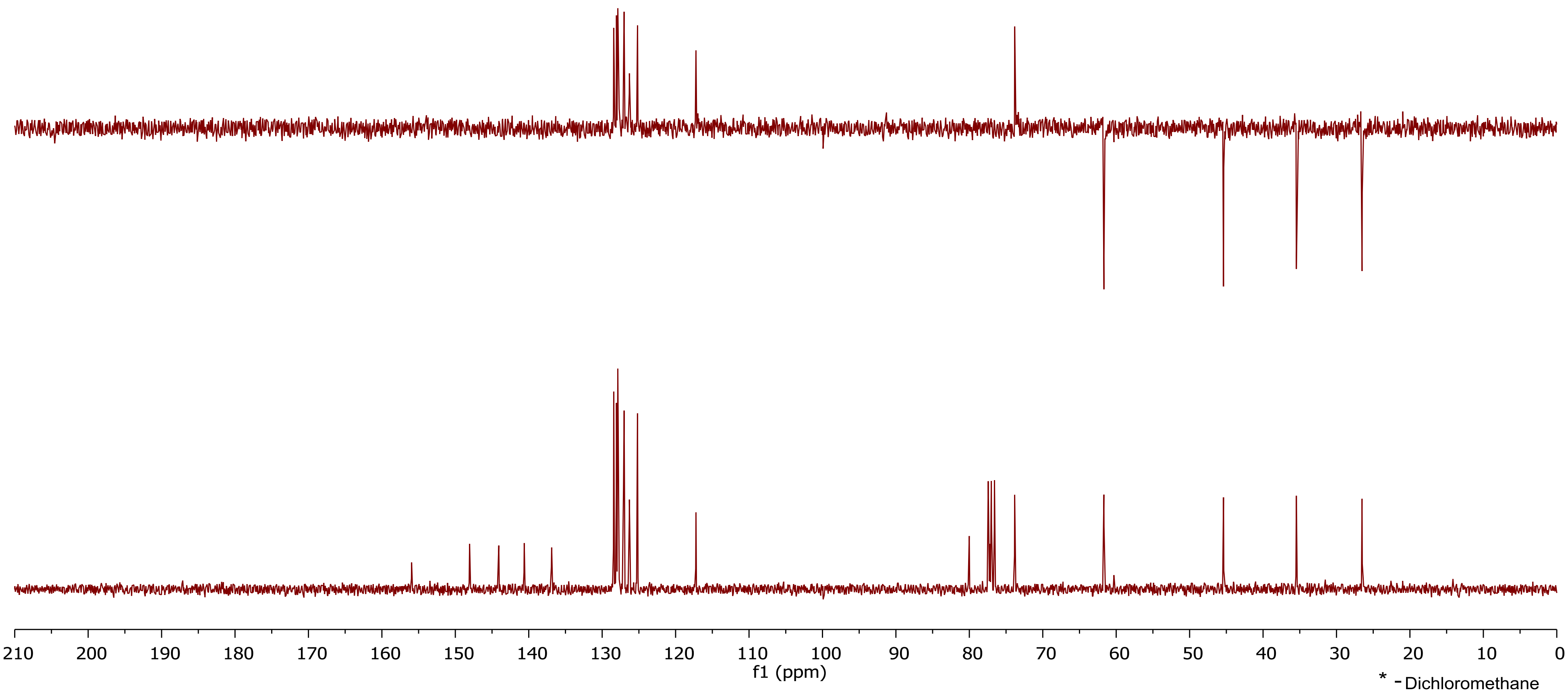

* - Dichloromethane

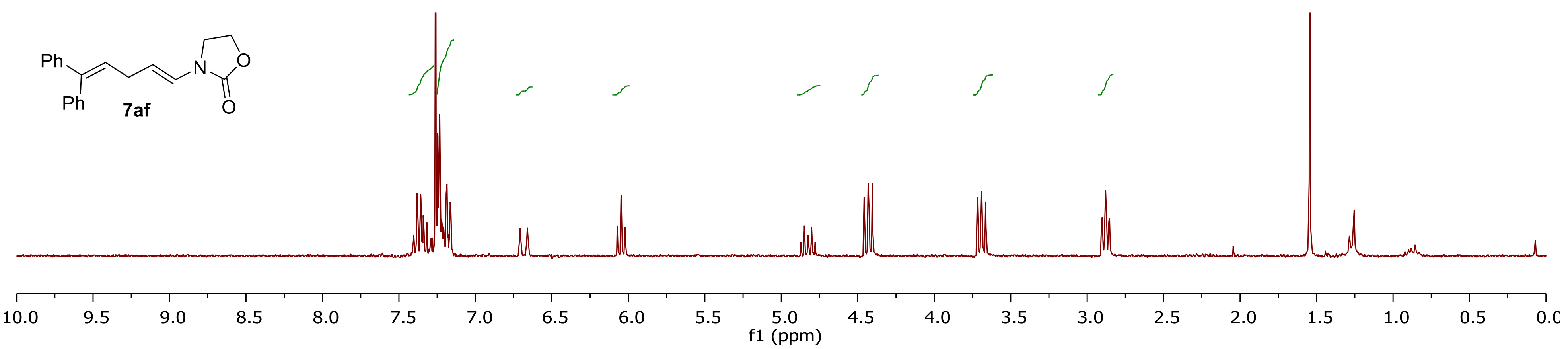
Ph
Ph
7af
N
O
O
10.0 9.5 9.0 8.5 8.0 7.5 7.0 6.5 6.0 5.5 5.0 4.5 4.0 3.5 3.0 2.5 2.0 1.5 1.0 0.5 0.0
f1 (ppm)

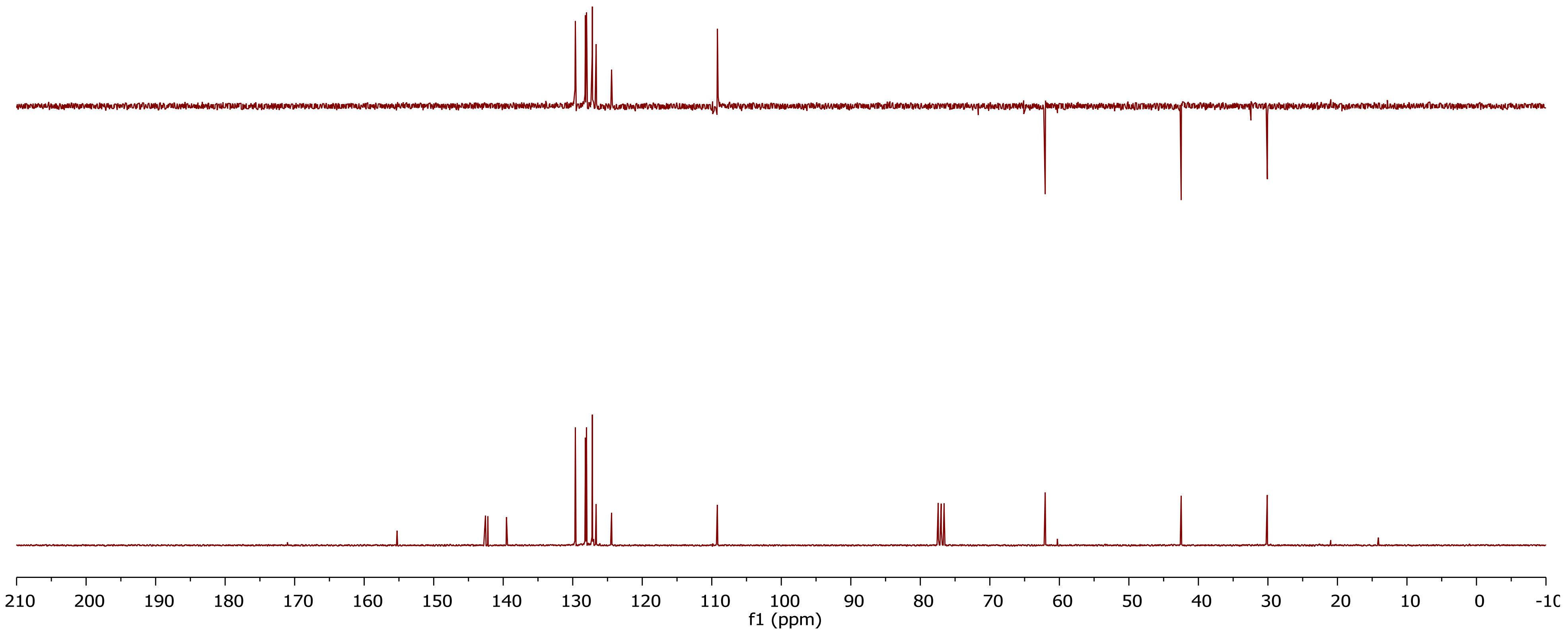
210 200 190 180 170 160 150 140 130 120 110 100 90 80 70 60 50 40 30 20 10 0 -10
f1 (ppm)

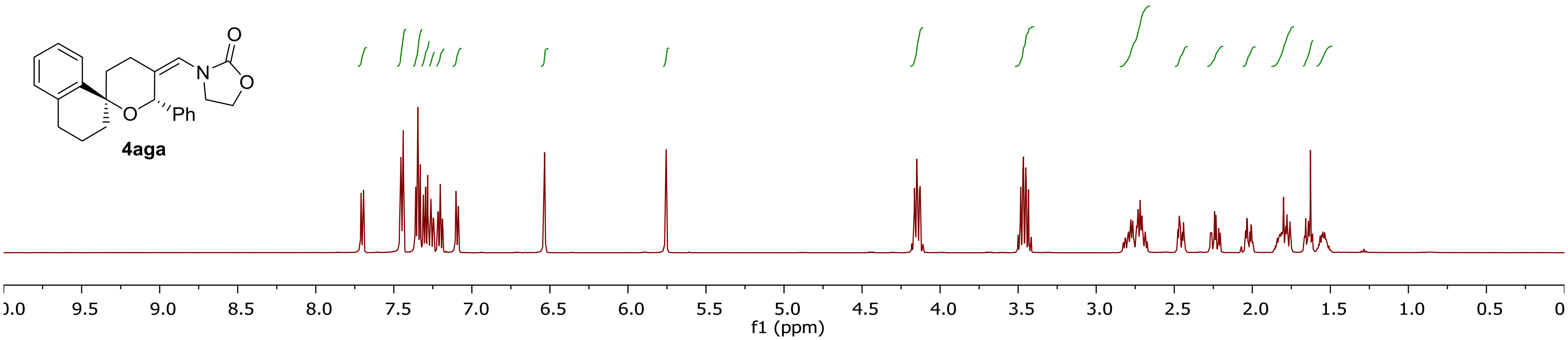
O
N
O
O
Ph
4aga
f1 (ppm)

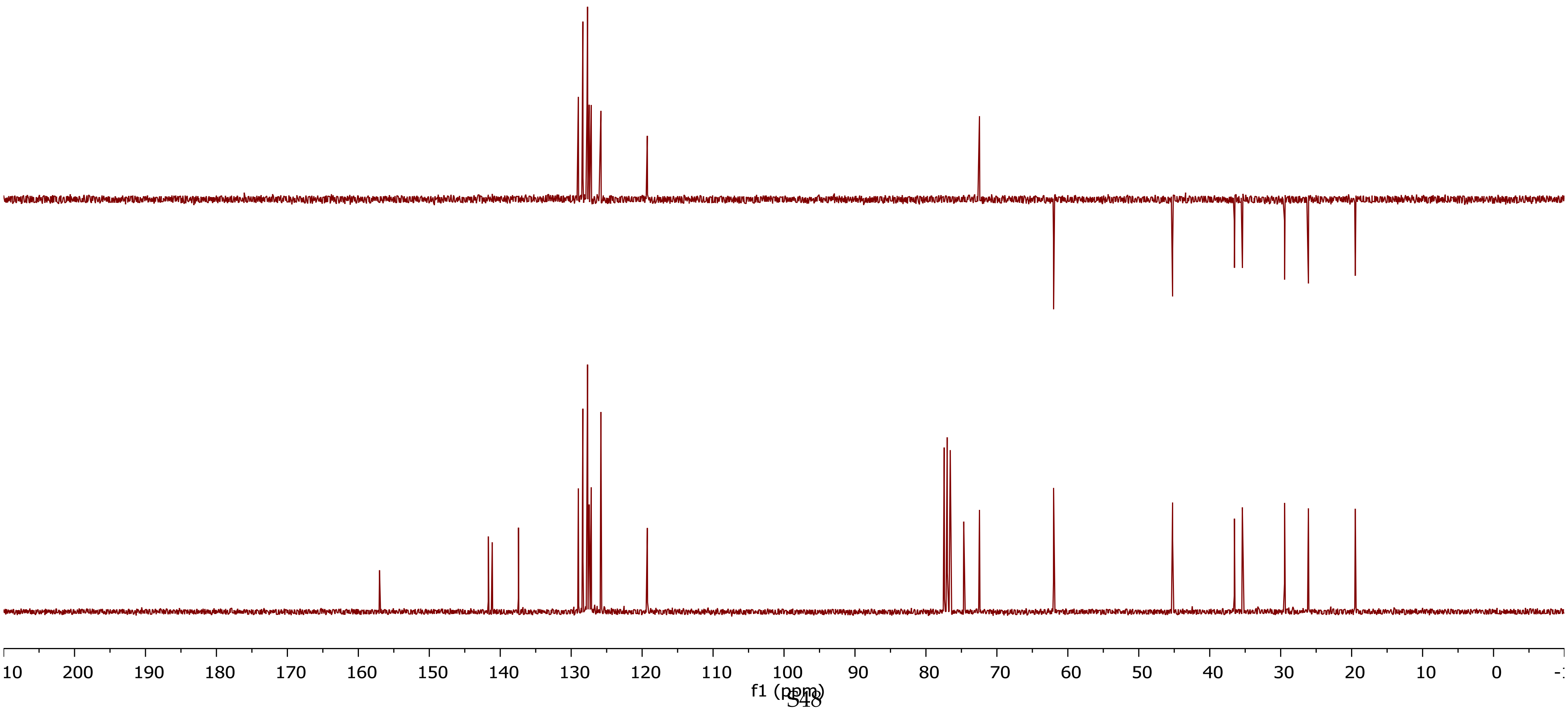
f1 (ppm)

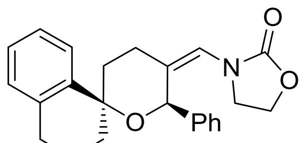

4aga'

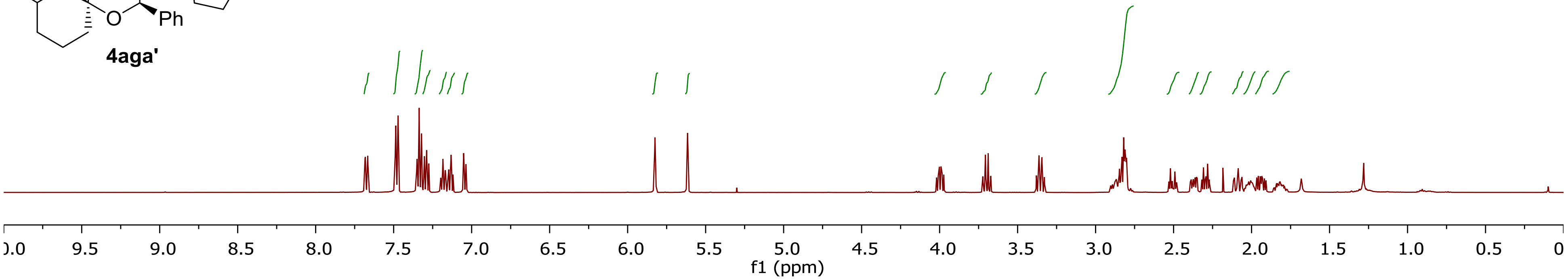

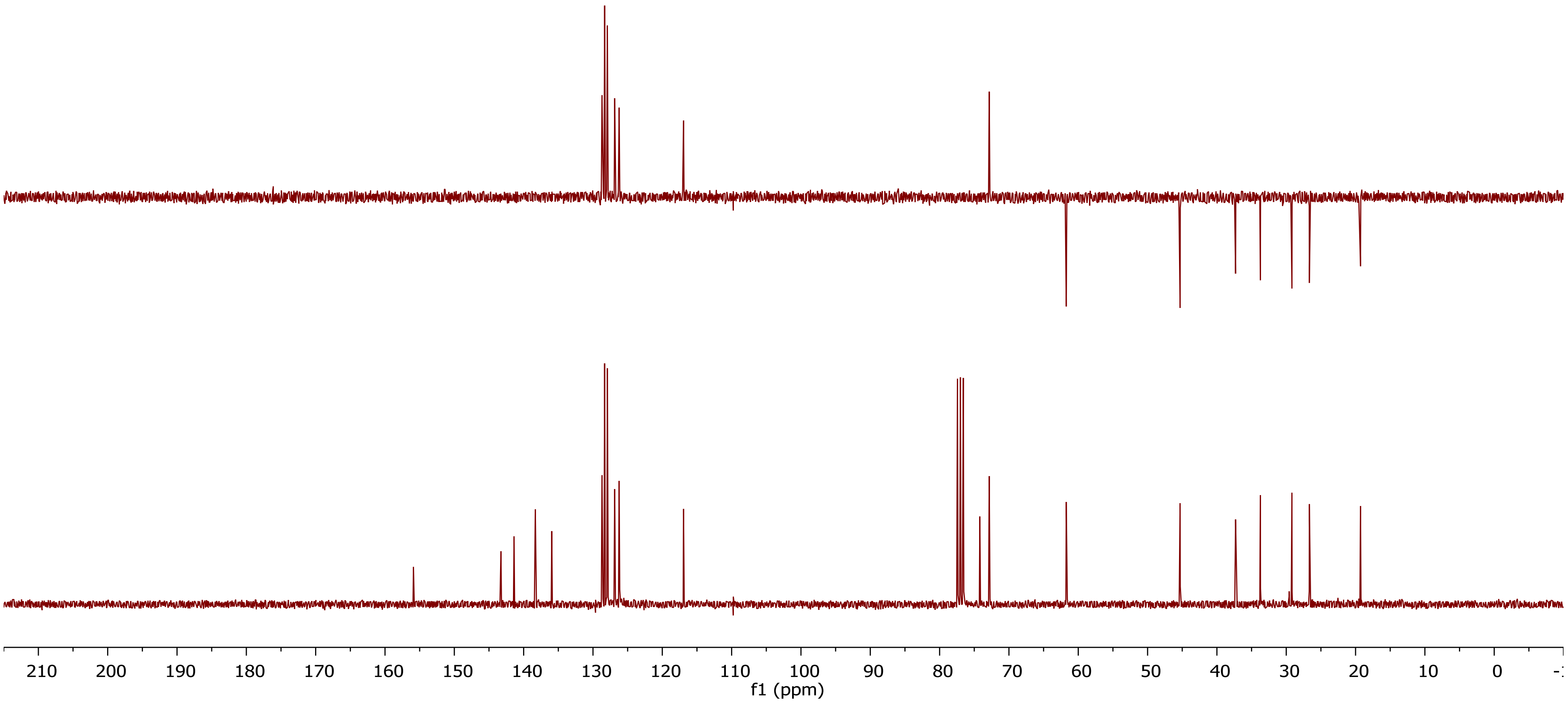

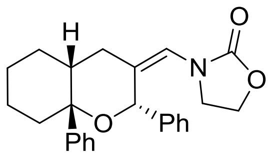

4aha

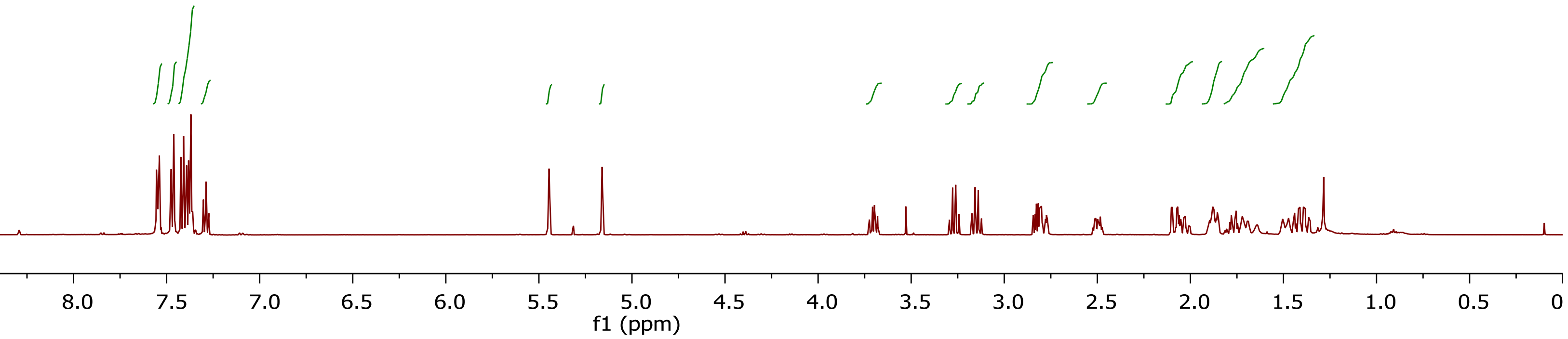

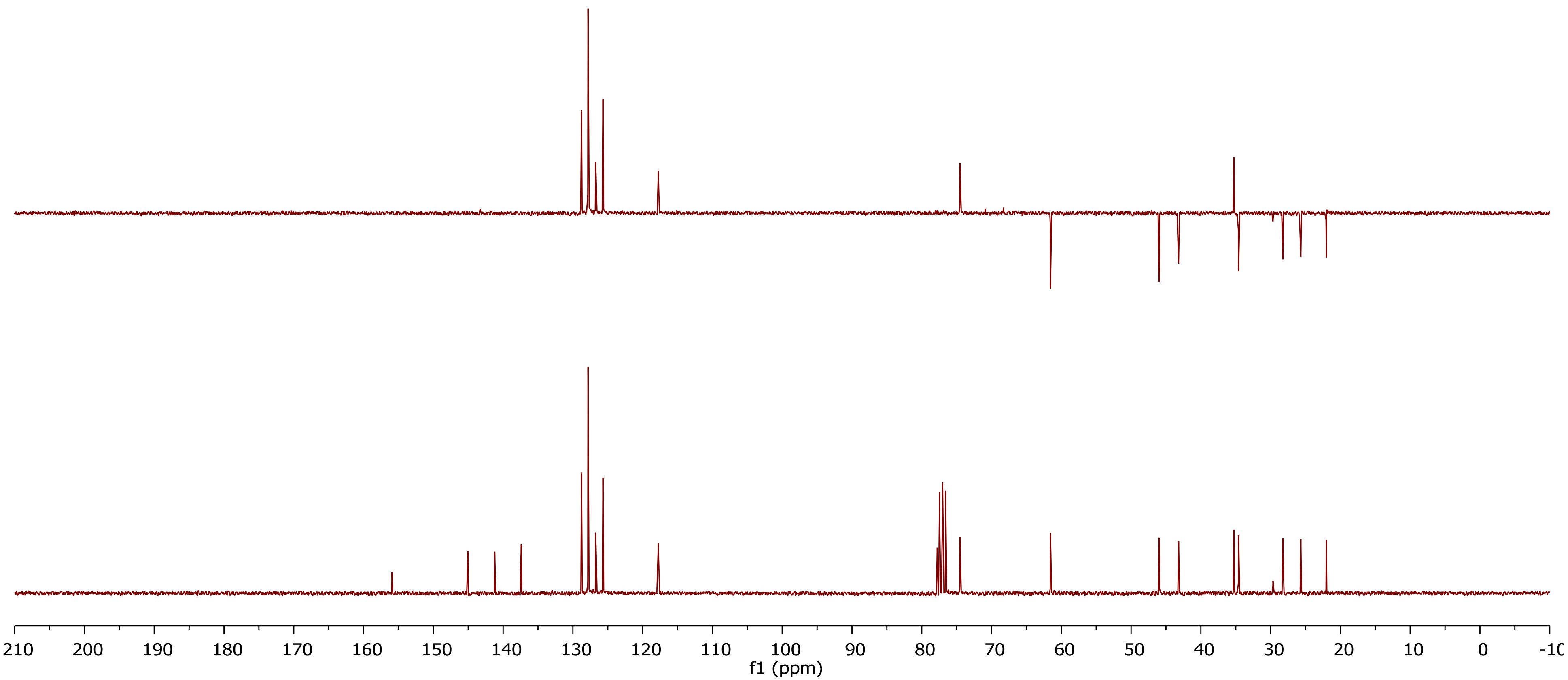

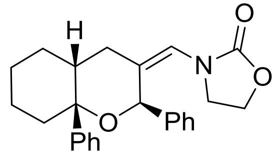

4aha'

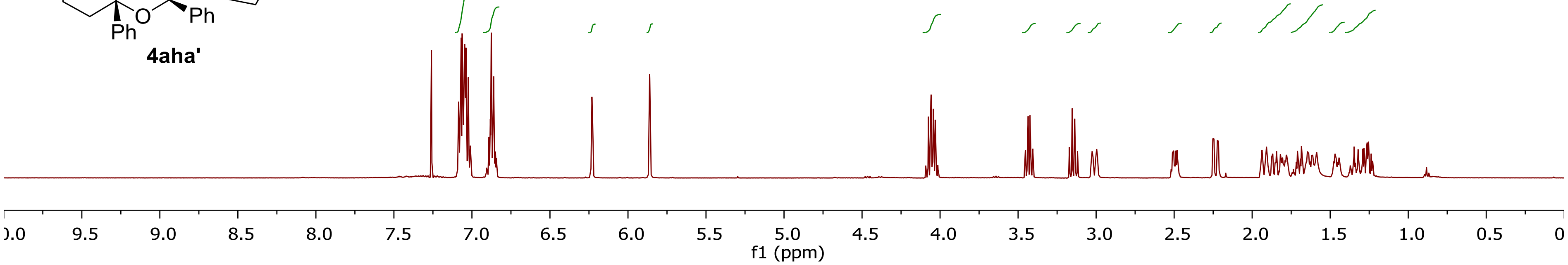

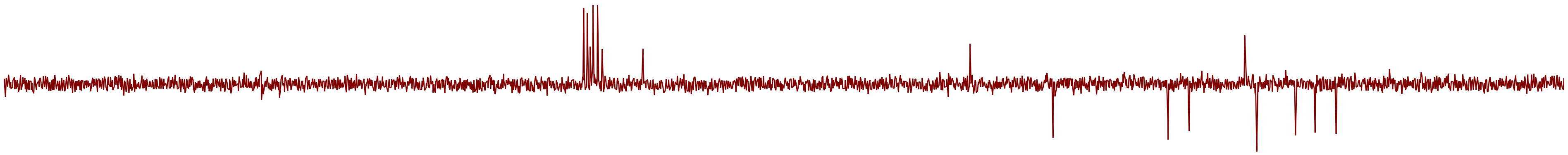

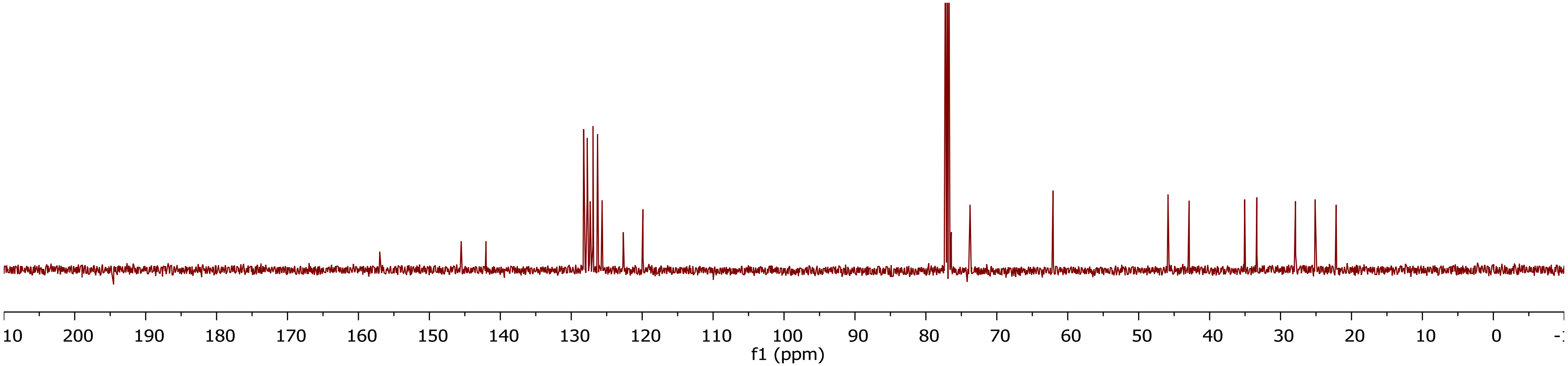

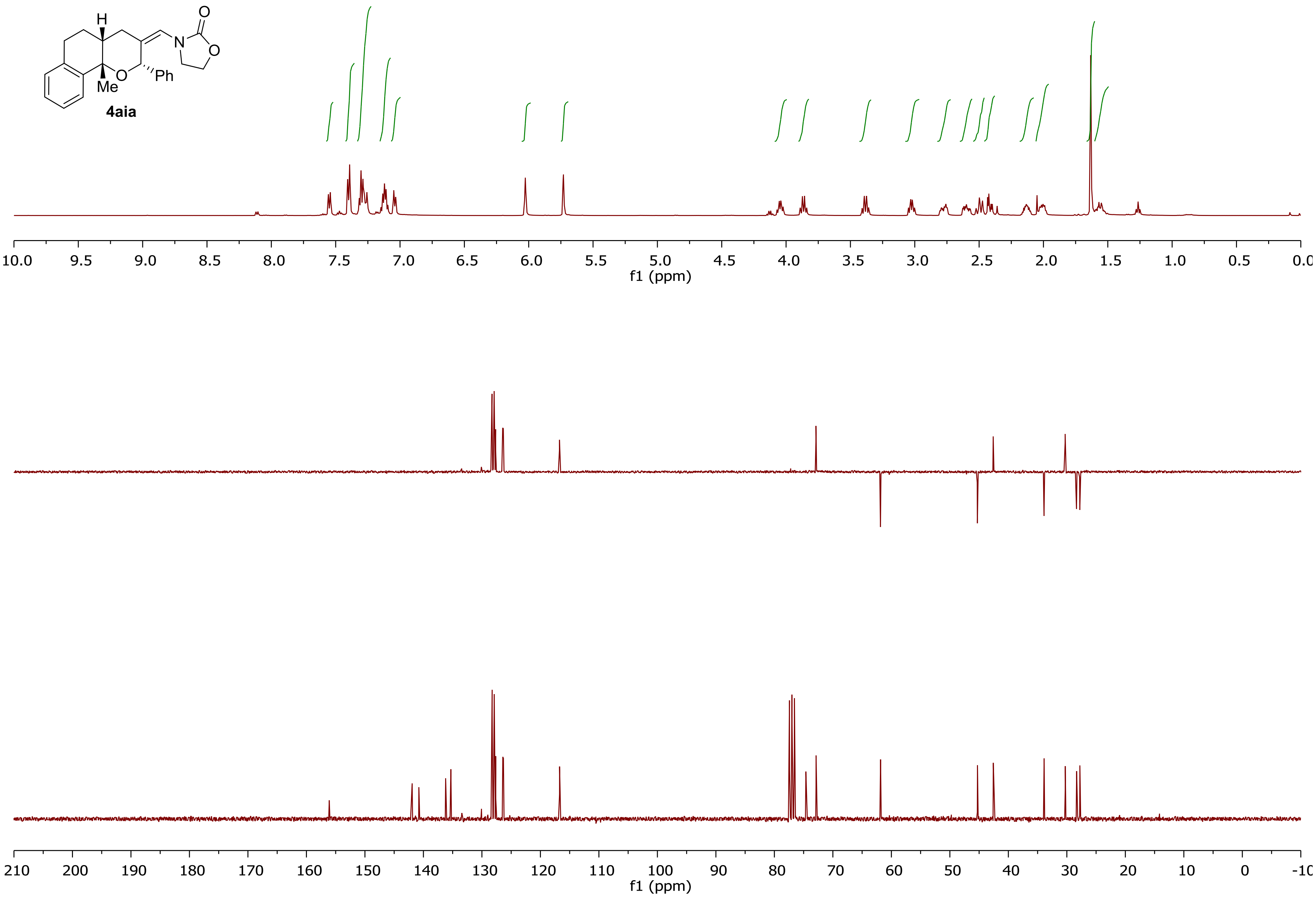
4aia
Me
Ph
H
f1 (ppm)
f1 (ppm)

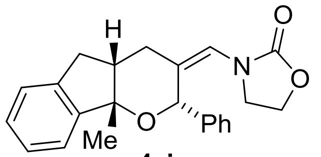

4aja

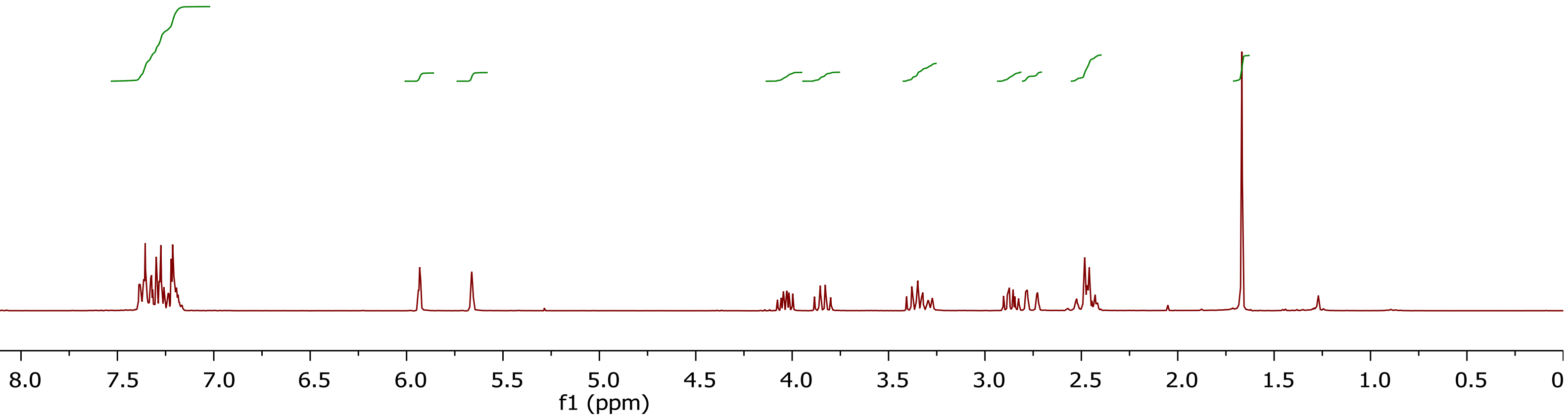

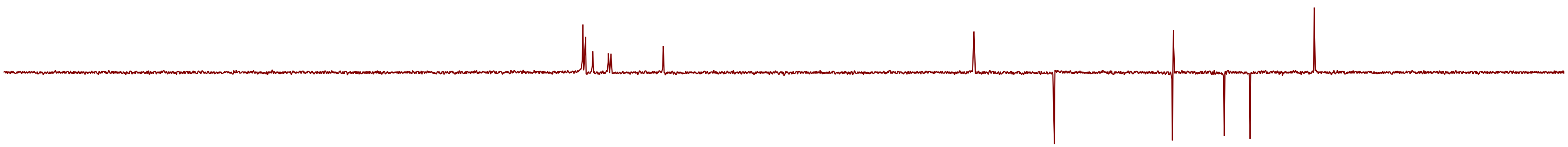

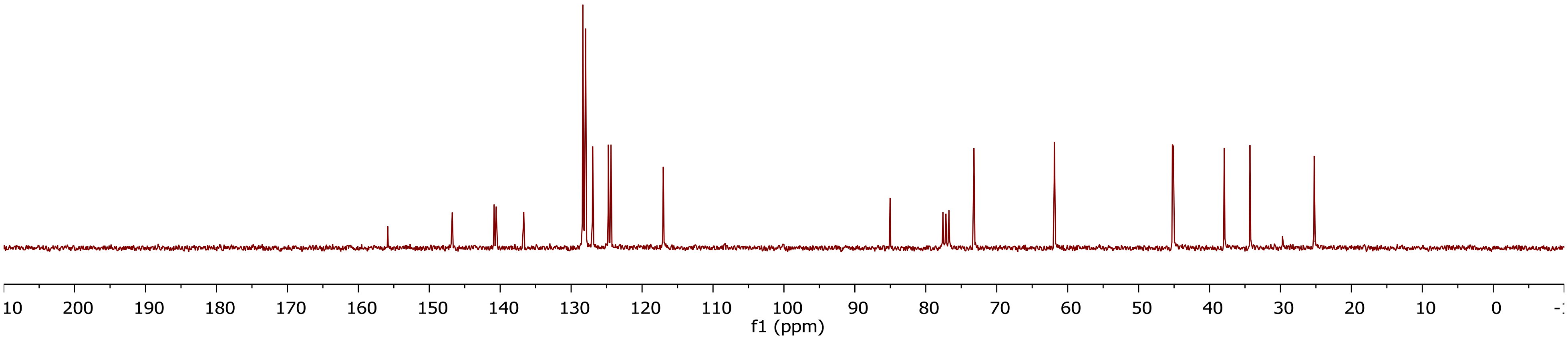

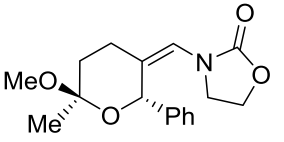
MeO
Me
O
Ph
N
O
O

4aka

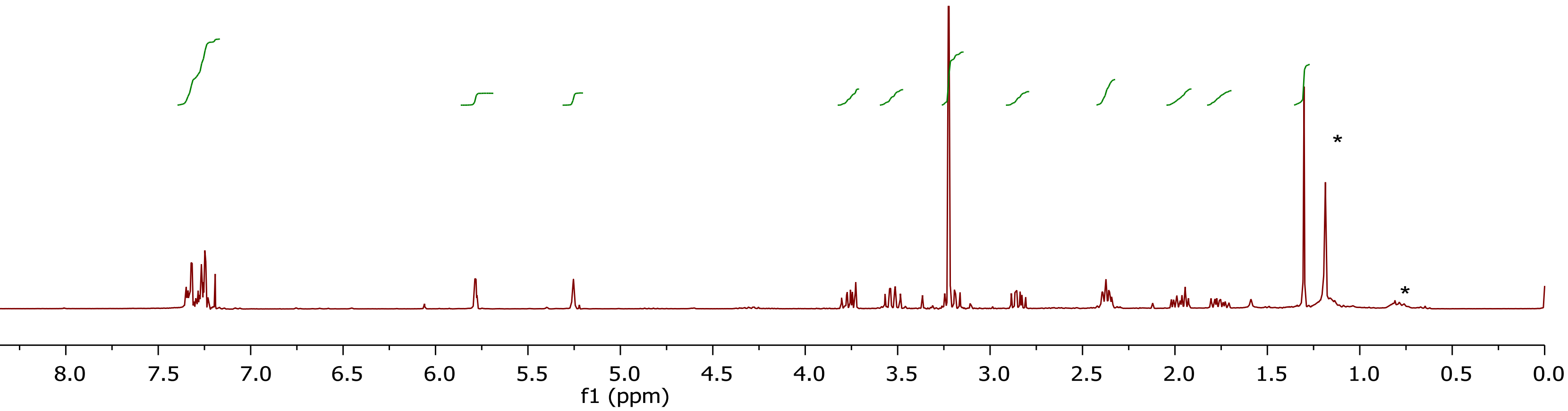
*
*
9.5
9.0
8.5
8.0
7.5
7.0
6.5
6.0
5.5
5.0
4.5
4.0
3.5
3.0
2.5
2.0
1.5
1.0
0.5
0.0
f1 (ppm)

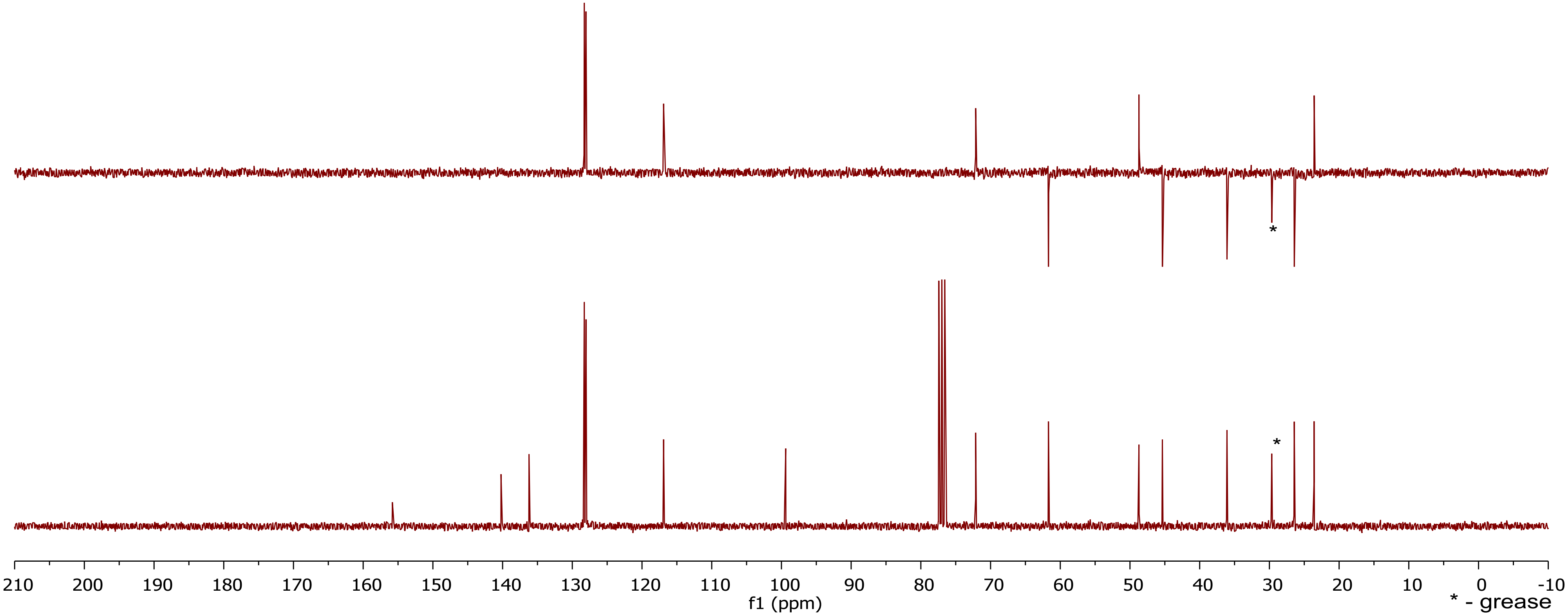
*
*
210
200
190
180
170
160
150
140
130
120
110
100
90
80
70
60
50
40
30
20
10
0
-10
f1 (ppm)
* - grease

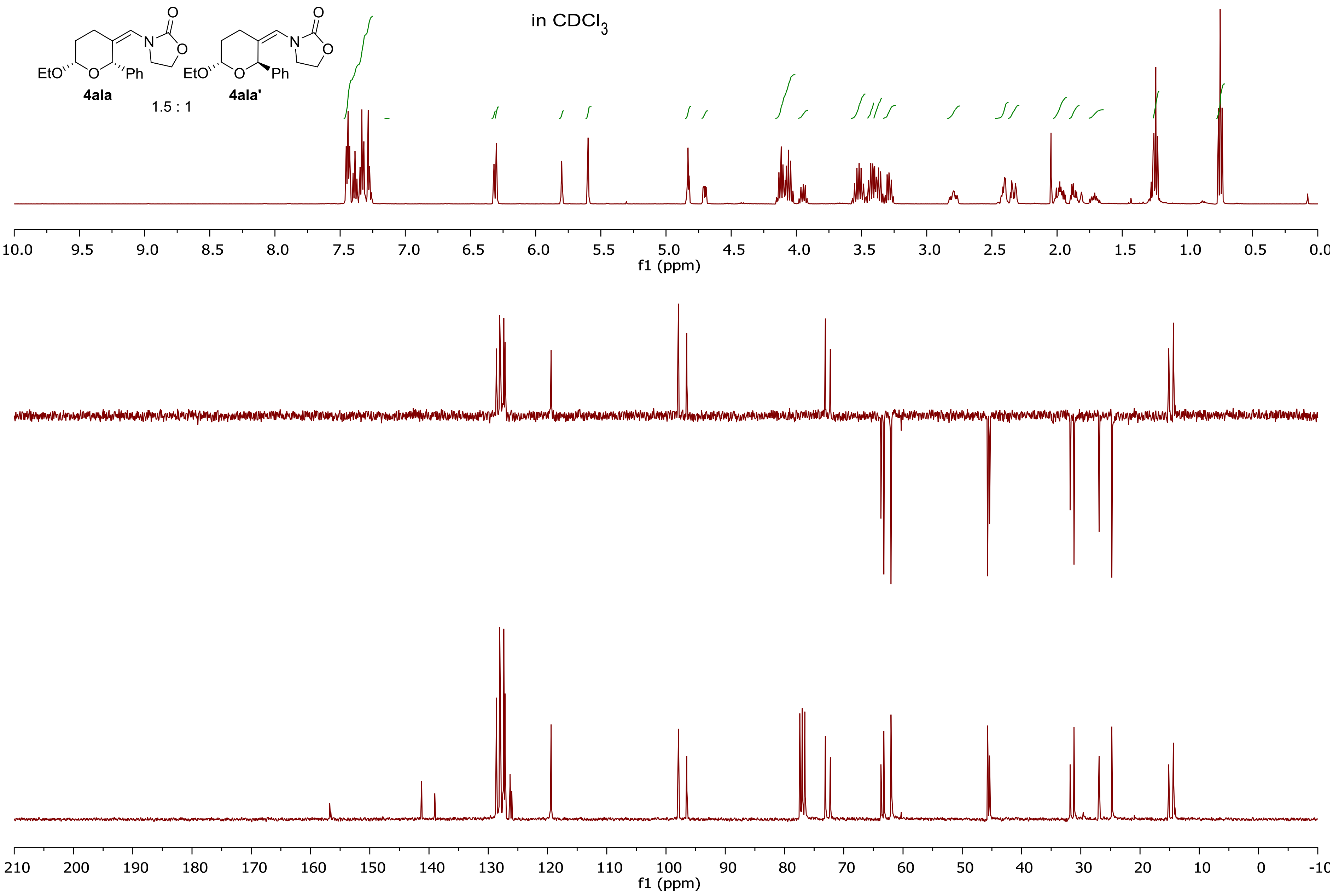
EtO
O
Ph
N
O
O
4ala
1.5 : 1
4ala'
in CDCl3
f1 (ppm)
f1 (ppm)

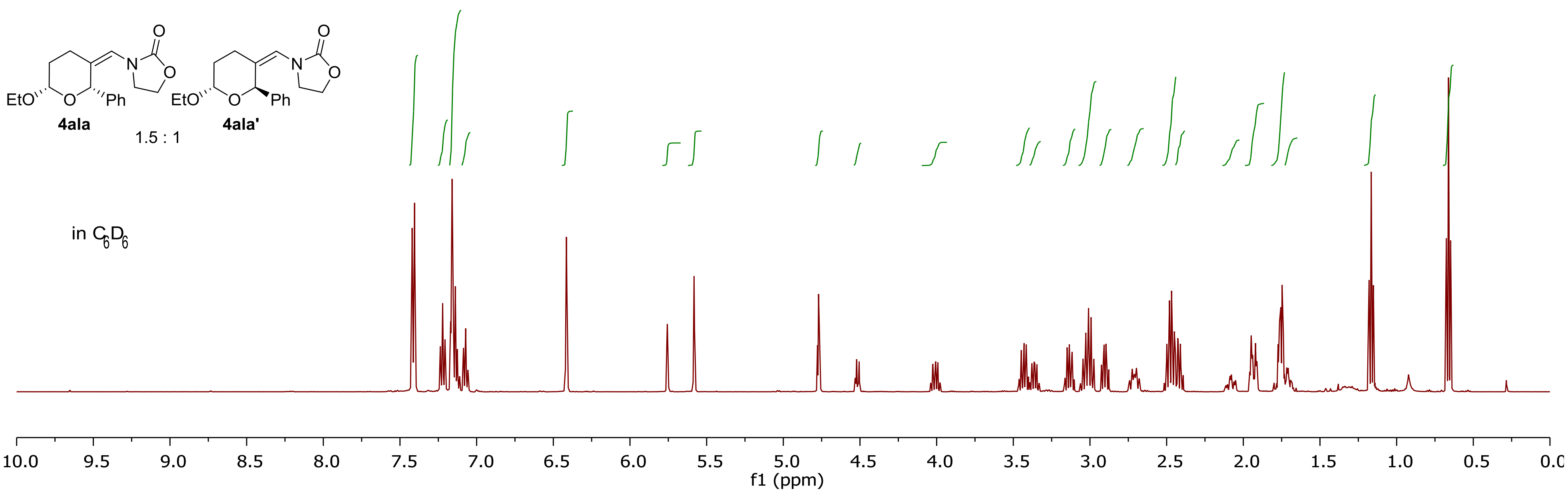

EtO
O
Ph
N
O
O
4ala
1.5 : 1
4ala'
in C_6D_6
f1 (ppm)

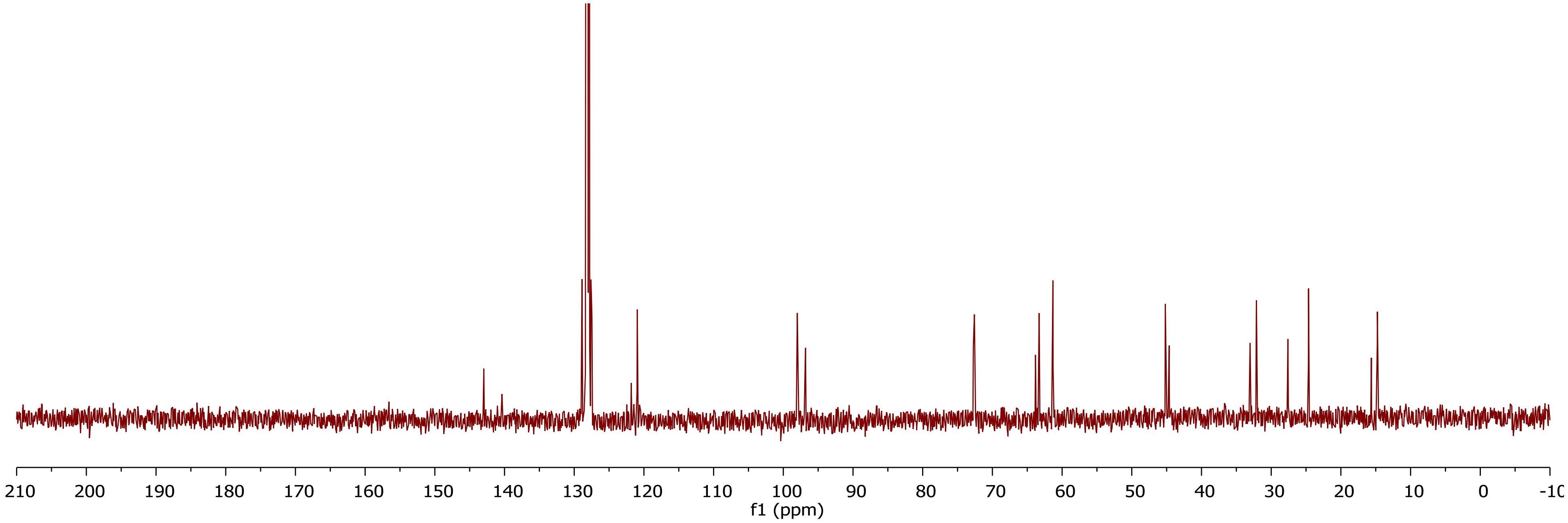

f1 (ppm)

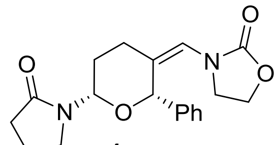

4ama

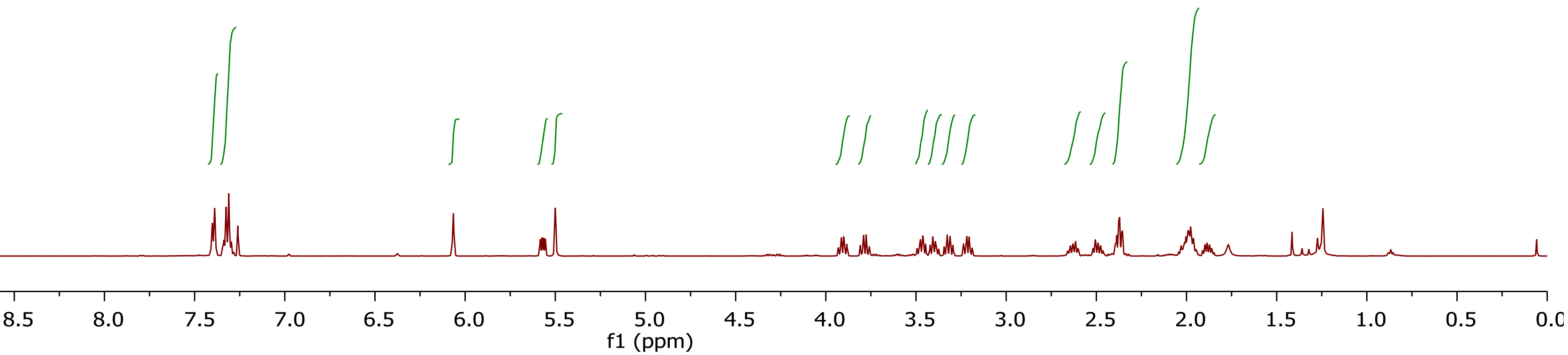

10.0
9.5
9.0
8.5
8.0
7.5
7.0
6.5
6.0
5.5
5.0
4.5
4.0
3.5
3.0
2.5
2.0
1.5
1.0
0.5
0.0
f1 (ppm)

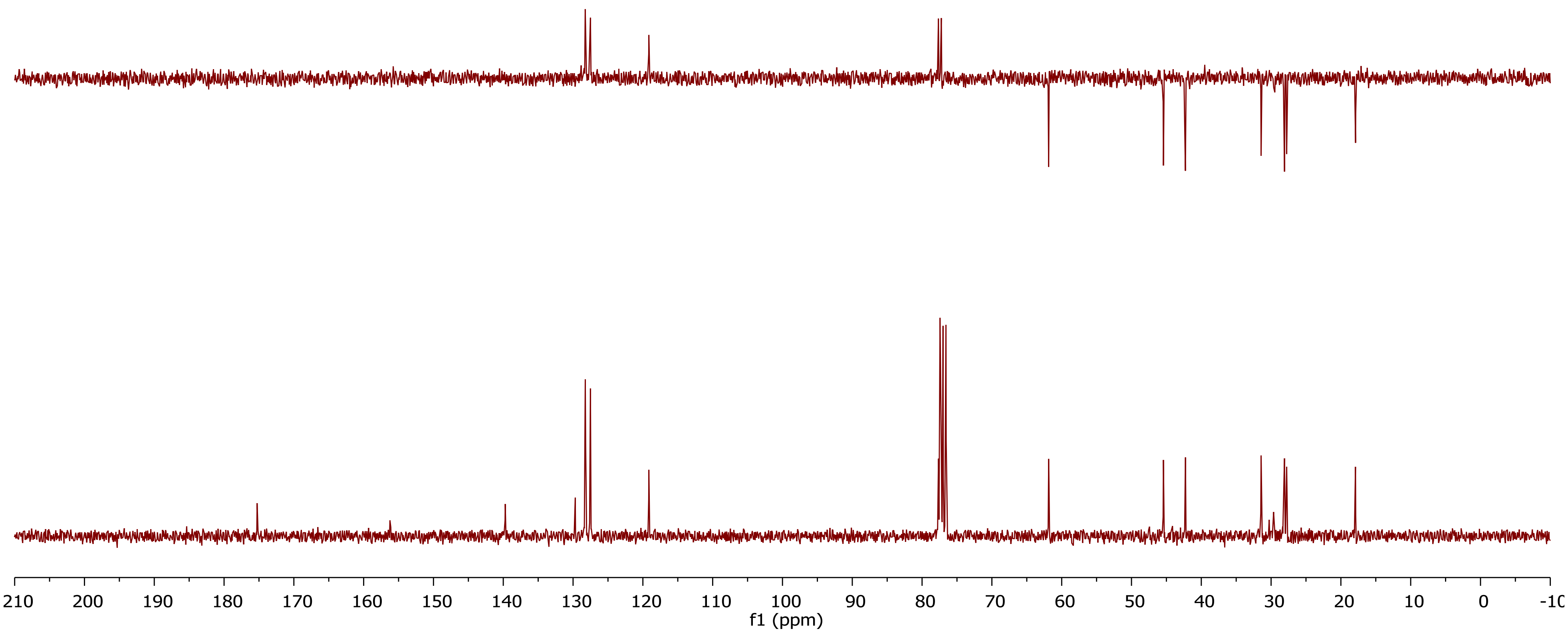

210
200
190
180
170
160
150
140
130
120
110
100
90
80
70
60
50
40
30
20
10
0
-10
f1 (ppm)

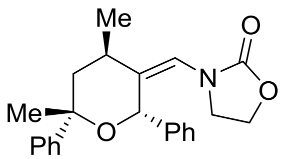
Me
O
N
O
Me
Ph
O
Ph

4bba

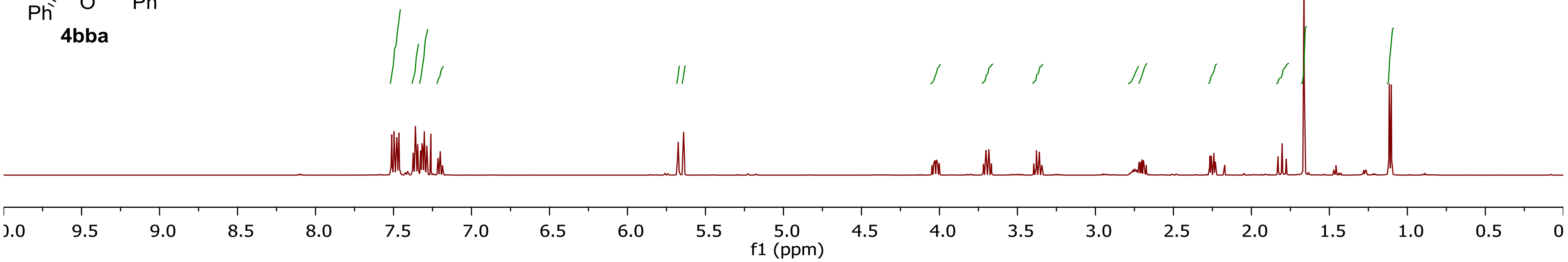
0.0
9.5
9.0
8.5
8.0
7.5
7.0
6.5
6.0
5.5
5.0
4.5
4.0
3.5
3.0
2.5
2.0
1.5
1.0
0.5
0
f1 (ppm)

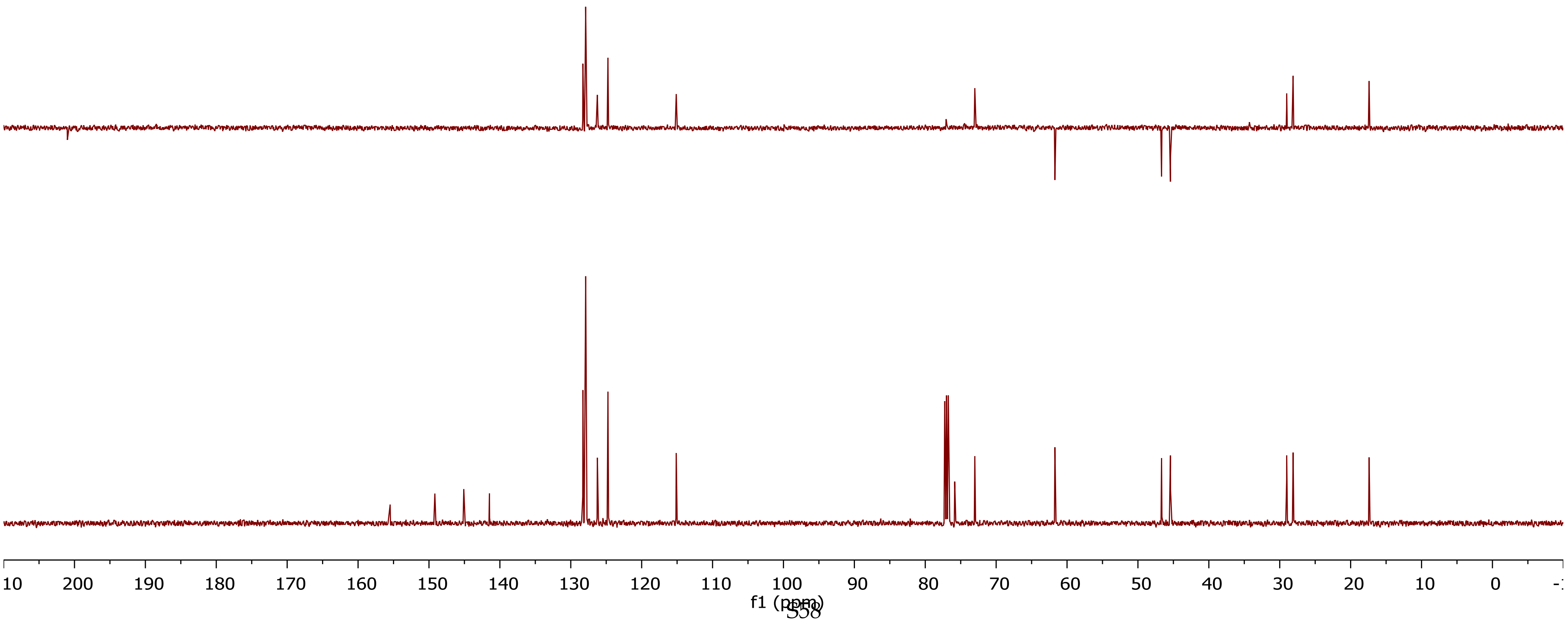
10
200
190
180
170
160
150
140
130
120
110
100
90
80
70
60
50
40
30
20
10
0
f1 (ppm)

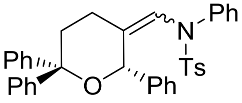

4cfa

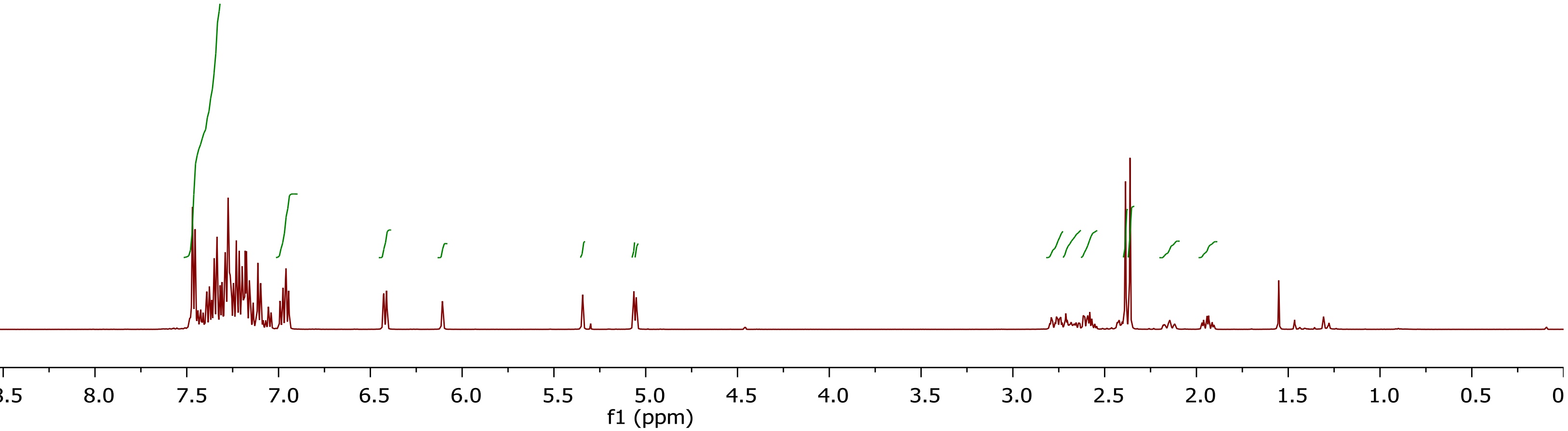

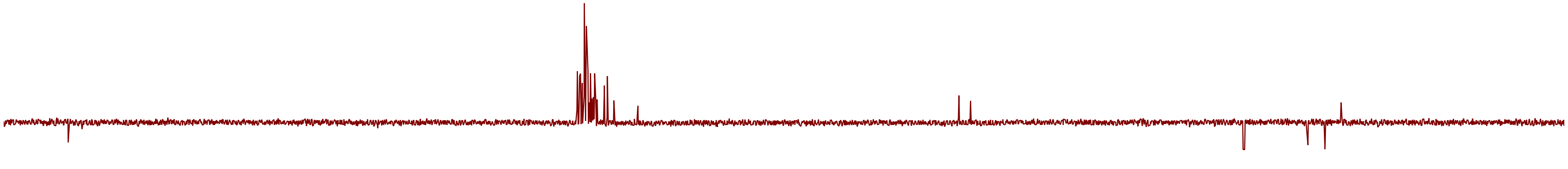

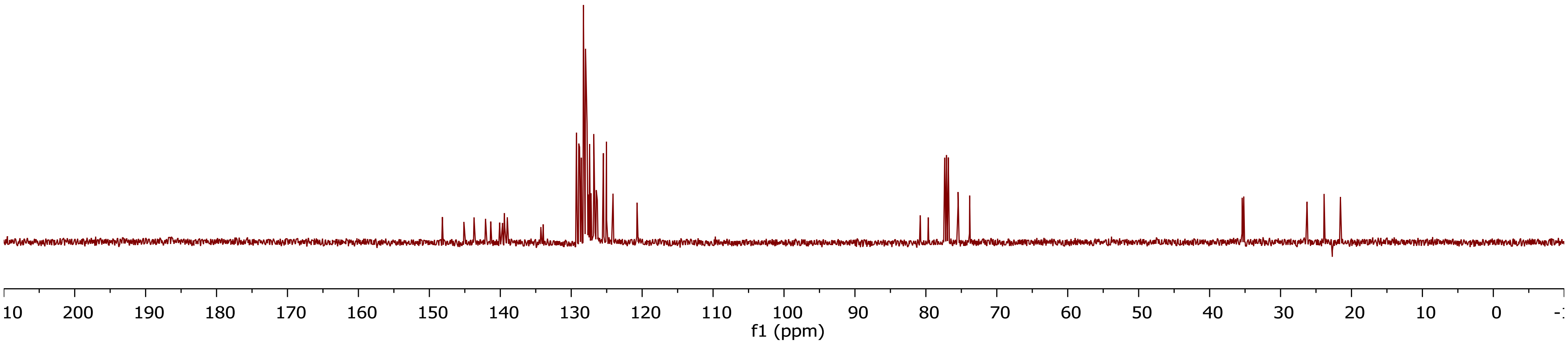

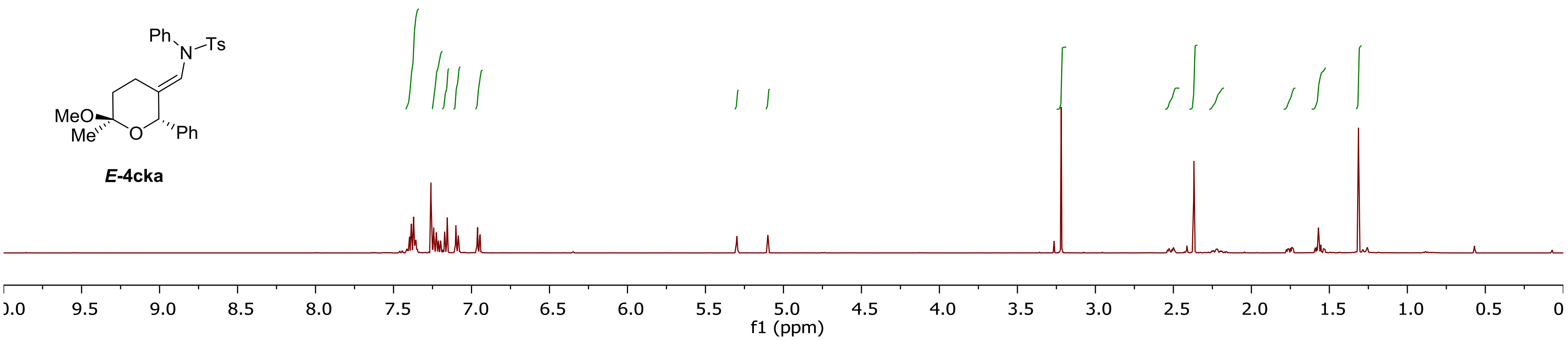

***E*-4cka**

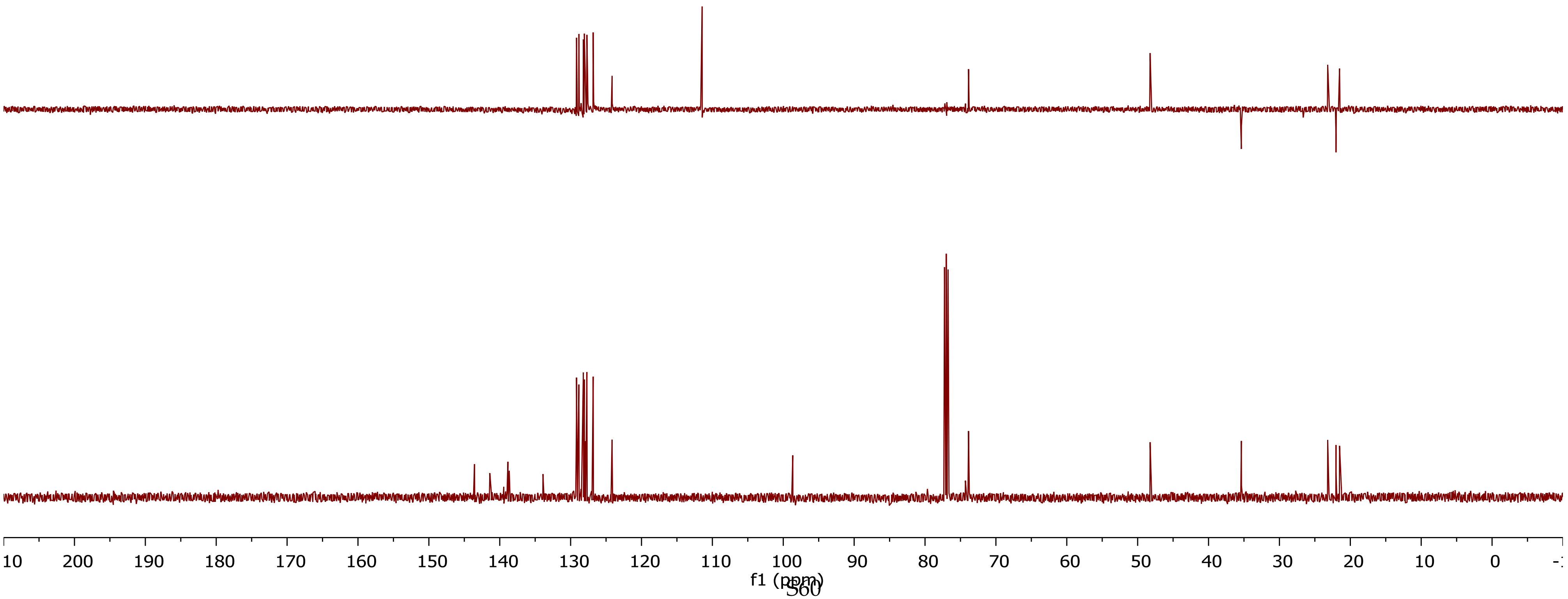

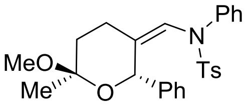
Ph
N
Ts
MeO
Me
O
Ph

Z-**4cka**

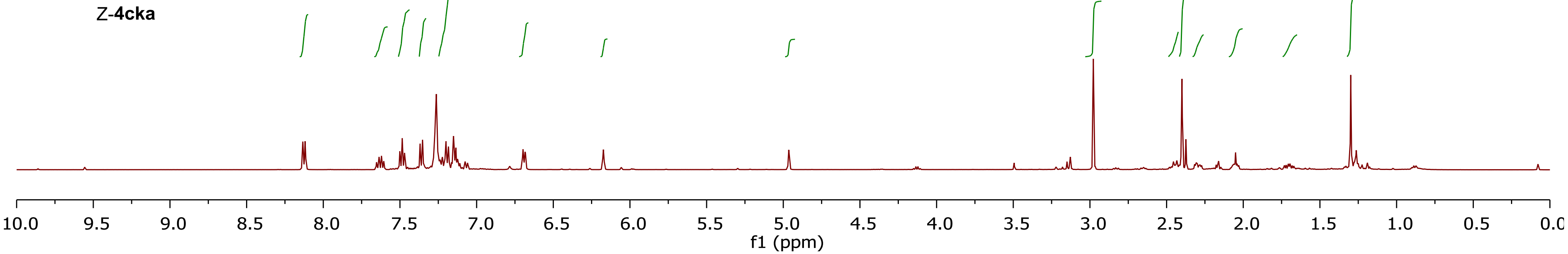
10.0 9.5 9.0 8.5 8.0 7.5 7.0 6.5 6.0 5.5 5.0 4.5 4.0 3.5 3.0 2.5 2.0 1.5 1.0 0.5 0.0
f1 (ppm)

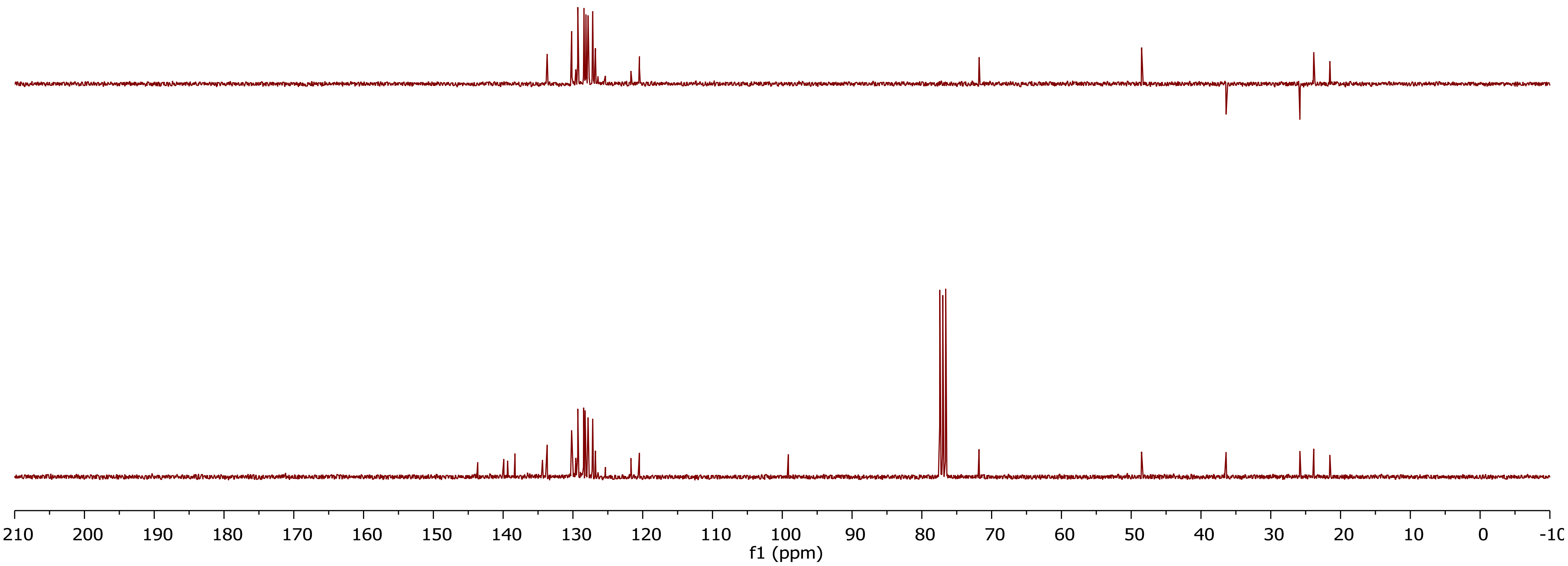
210 200 190 180 170 160 150 140 130 120 110 100 90 80 70 60 50 40 30 20 10 0 -10
f1 (ppm)

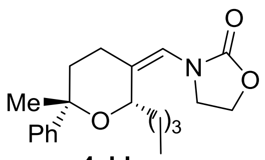

Me
Ph
O
N
O
O
3

4abb

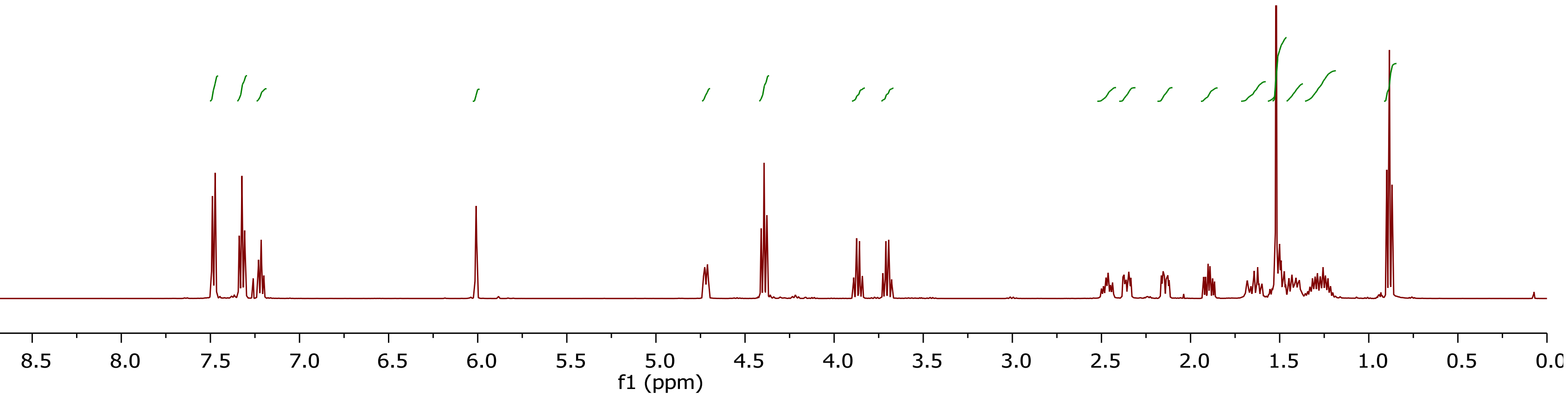

10.0 9.5 9.0 8.5 8.0 7.5 7.0 6.5 6.0 5.5 5.0 4.5 4.0 3.5 3.0 2.5 2.0 1.5 1.0 0.5 0.0
f1 (ppm)

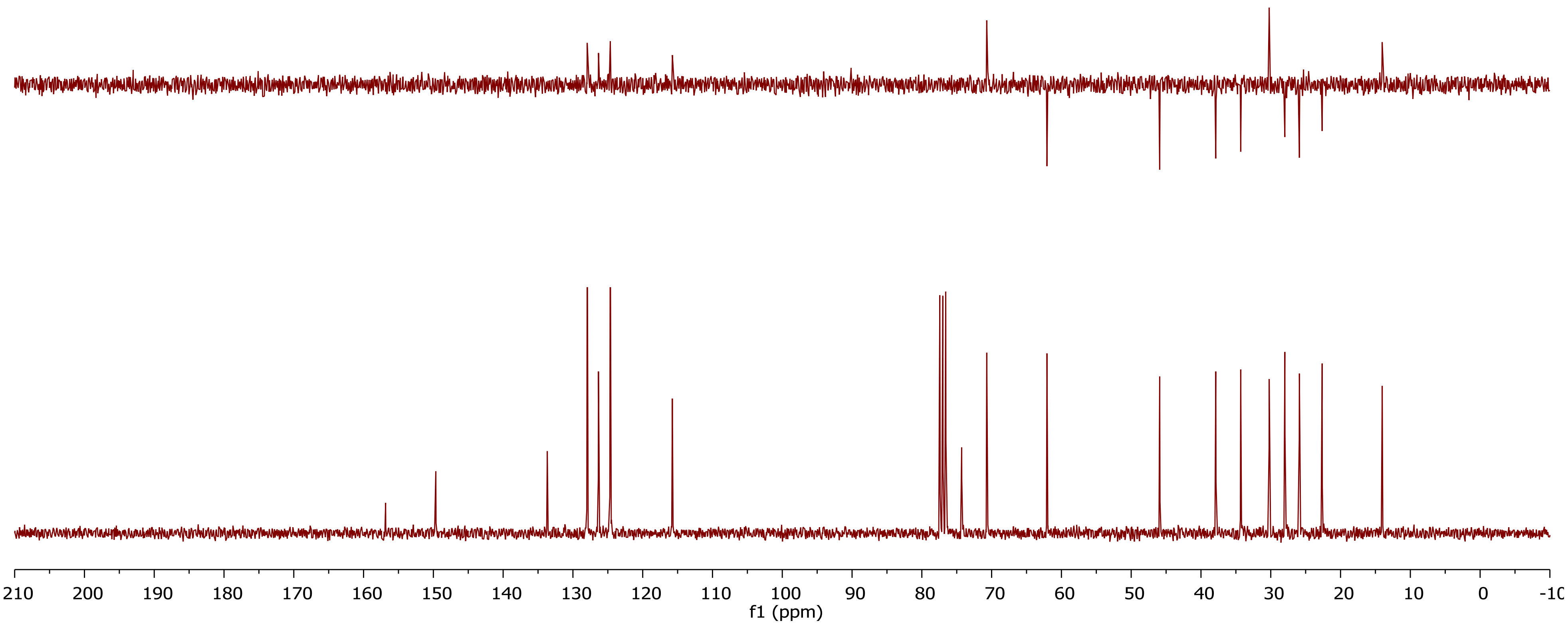

210 200 190 180 170 160 150 140 130 120 110 100 90 80 70 60 50 40 30 20 10 0 -10
f1 (ppm)

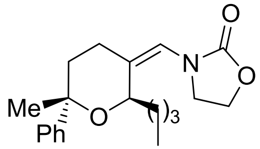

4abb'

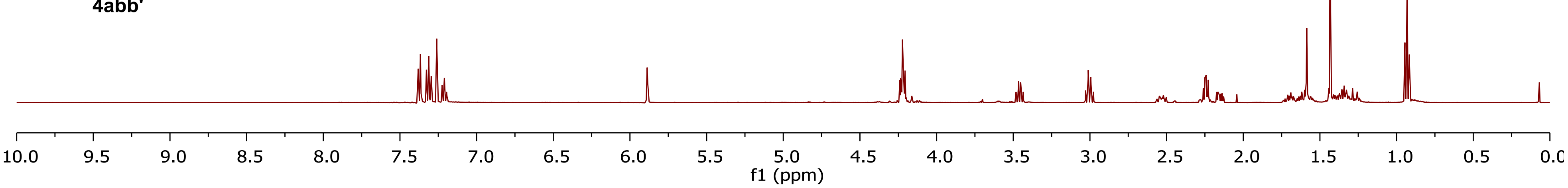

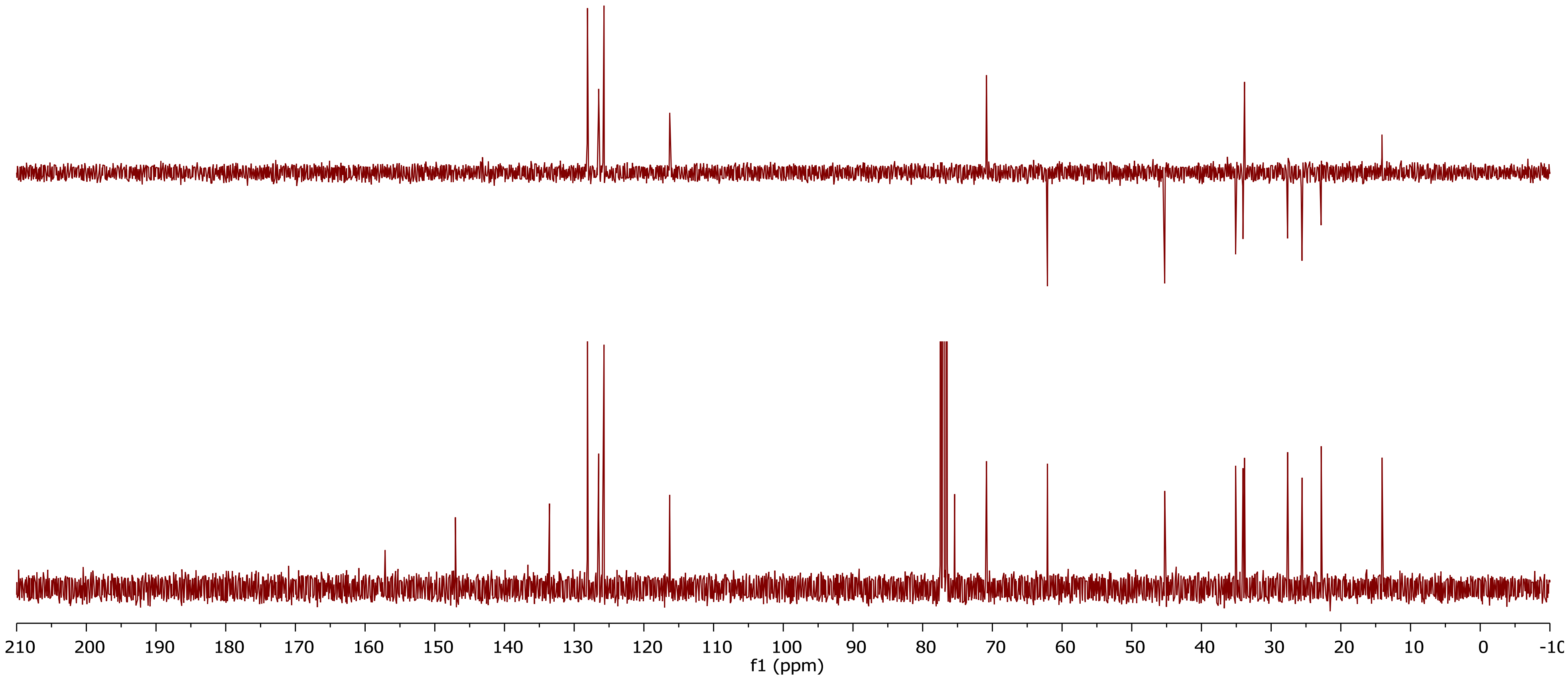

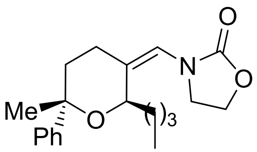
Me
Ph
O
N
O
O
3

4abb'

In C_6D_6

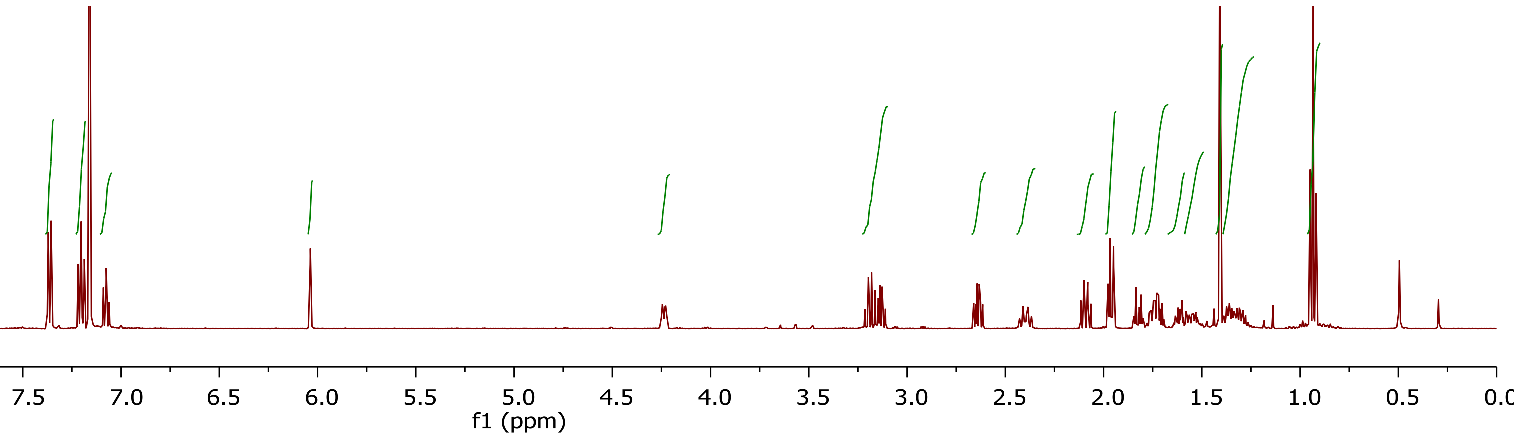
10.0 9.5 9.0 8.5 8.0 7.5 7.0 6.5 6.0 5.5 5.0 4.5 4.0 3.5 3.0 2.5 2.0 1.5 1.0 0.5 0.0
f1 (ppm)

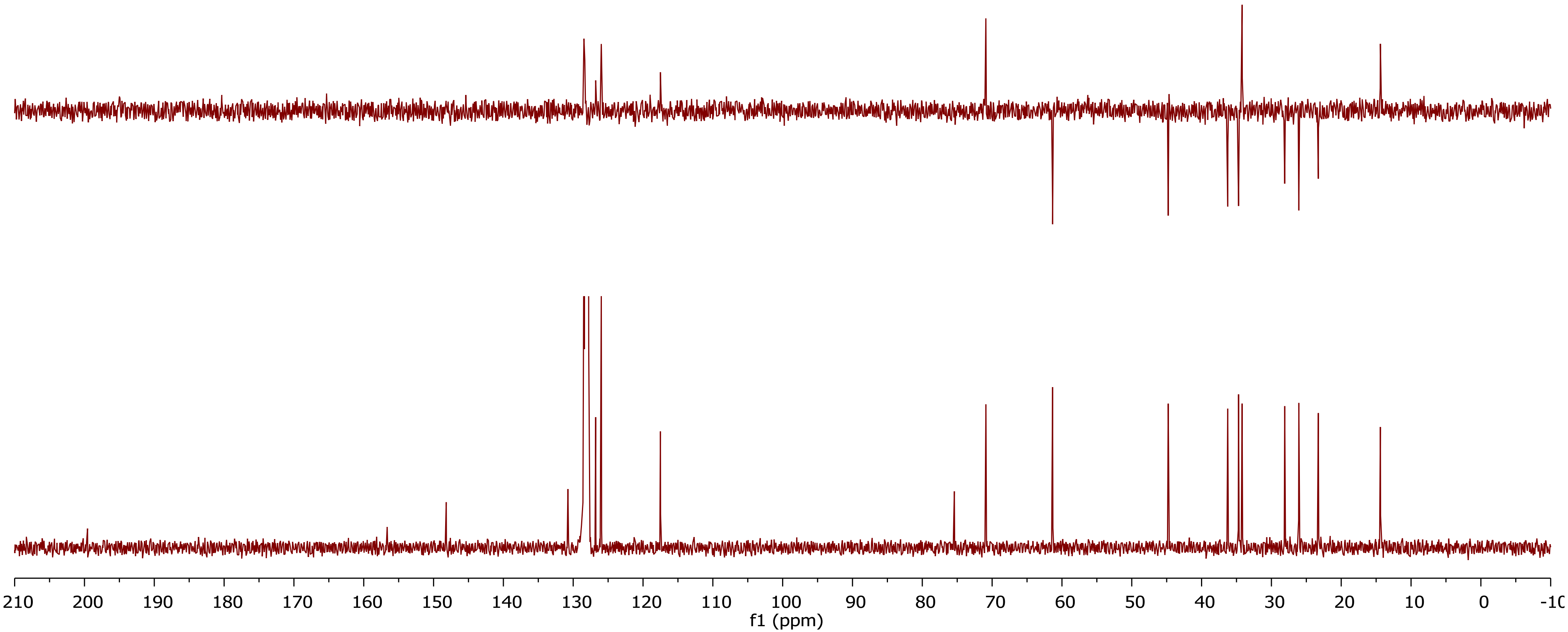
210 200 190 180 170 160 150 140 130 120 110 100 90 80 70 60 50 40 30 20 10 0 -10
f1 (ppm)

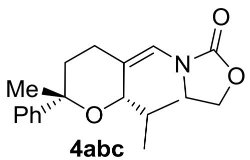

O
N
O
Me
Ph
O

4abc

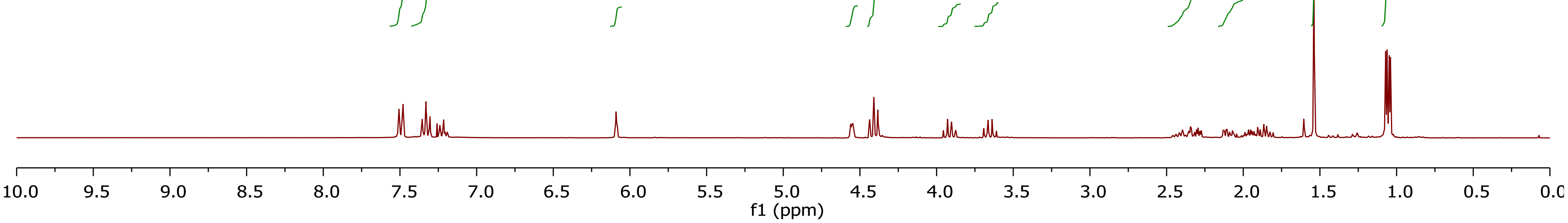

10.0 9.5 9.0 8.5 8.0 7.5 7.0 6.5 6.0 5.5 5.0 4.5 4.0 3.5 3.0 2.5 2.0 1.5 1.0 0.5 0.0
f1 (ppm)

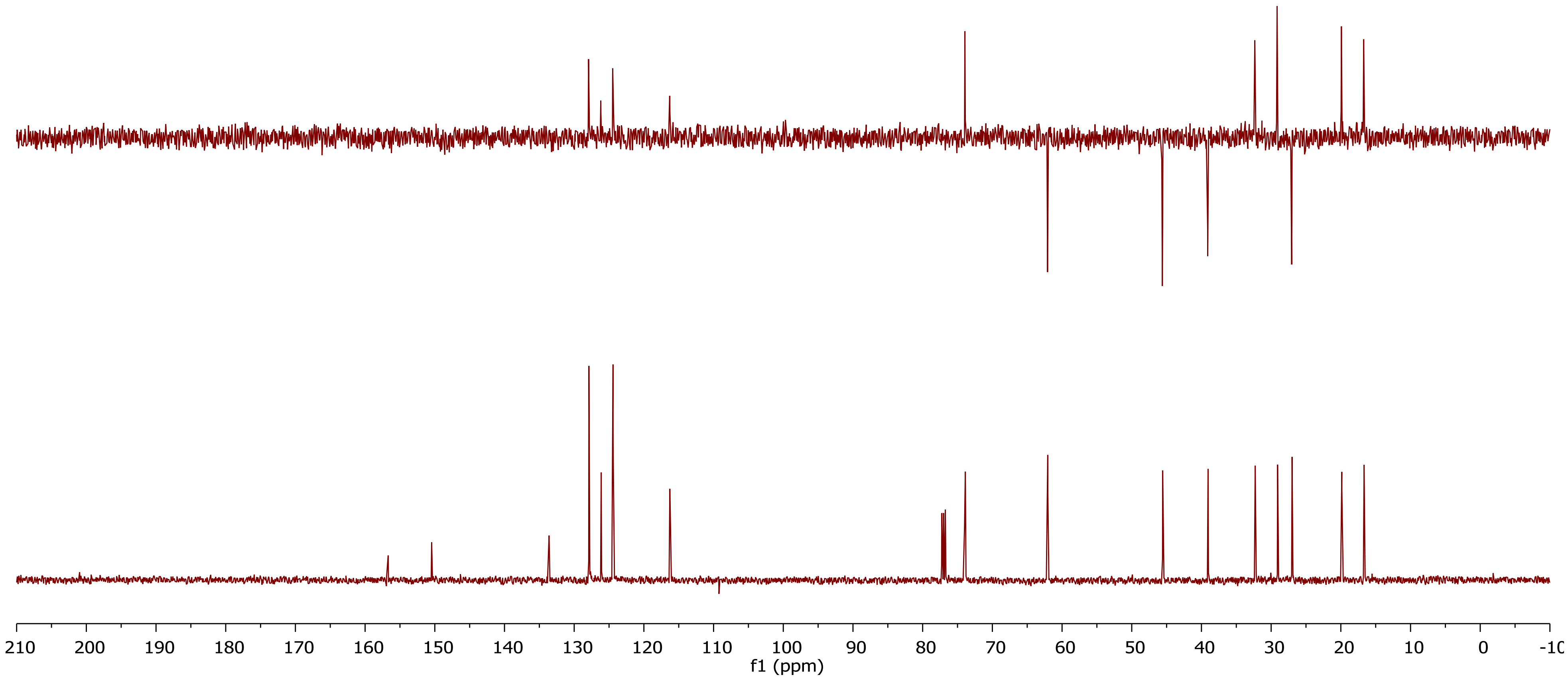

210 200 190 180 170 160 150 140 130 120 110 100 90 80 70 60 50 40 30 20 10 0 -10
f1 (ppm)

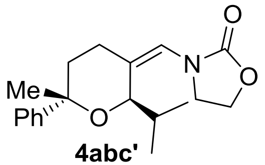
Me
Ph
O
N
O
O

4abc'

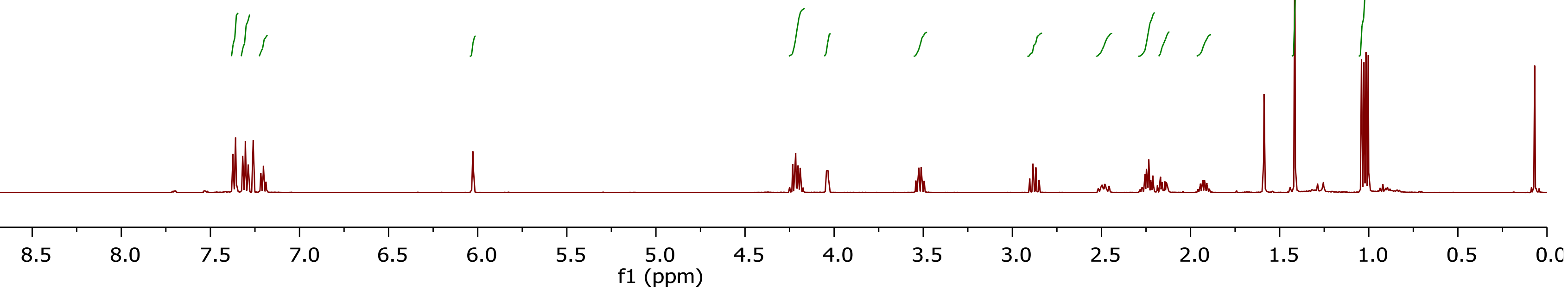
10.0 9.5 9.0 8.5 8.0 7.5 7.0 6.5 6.0 5.5 5.0 4.5 4.0 3.5 3.0 2.5 2.0 1.5 1.0 0.5 0.0
f1 (ppm)

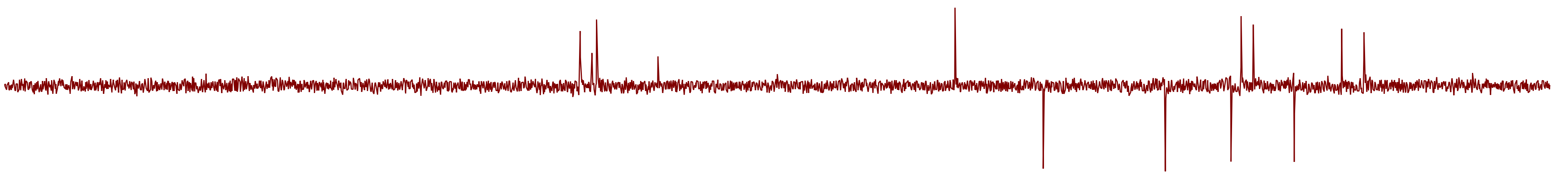

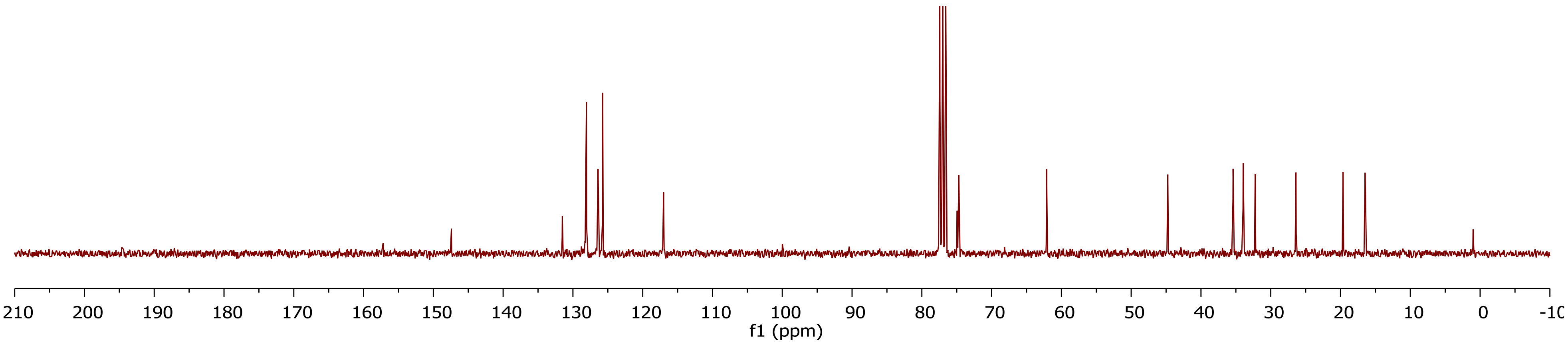
210 200 190 180 170 160 150 140 130 120 110 100 90 80 70 60 50 40 30 20 10 0 -10
f1 (ppm)

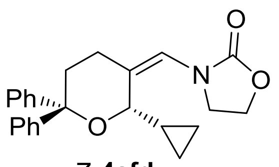

Z-**4afd**

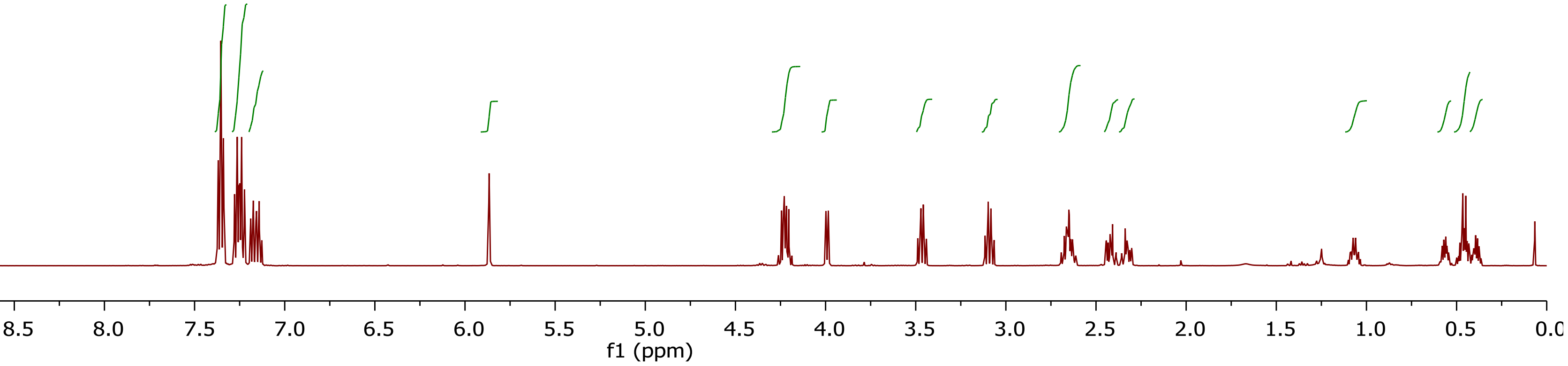

10.0
9.5
9.0
8.5
8.0
7.5
7.0
6.5
6.0
5.5
5.0
4.5
4.0
3.5
3.0
2.5
2.0
1.5
1.0
0.5
0.0
f1 (ppm)

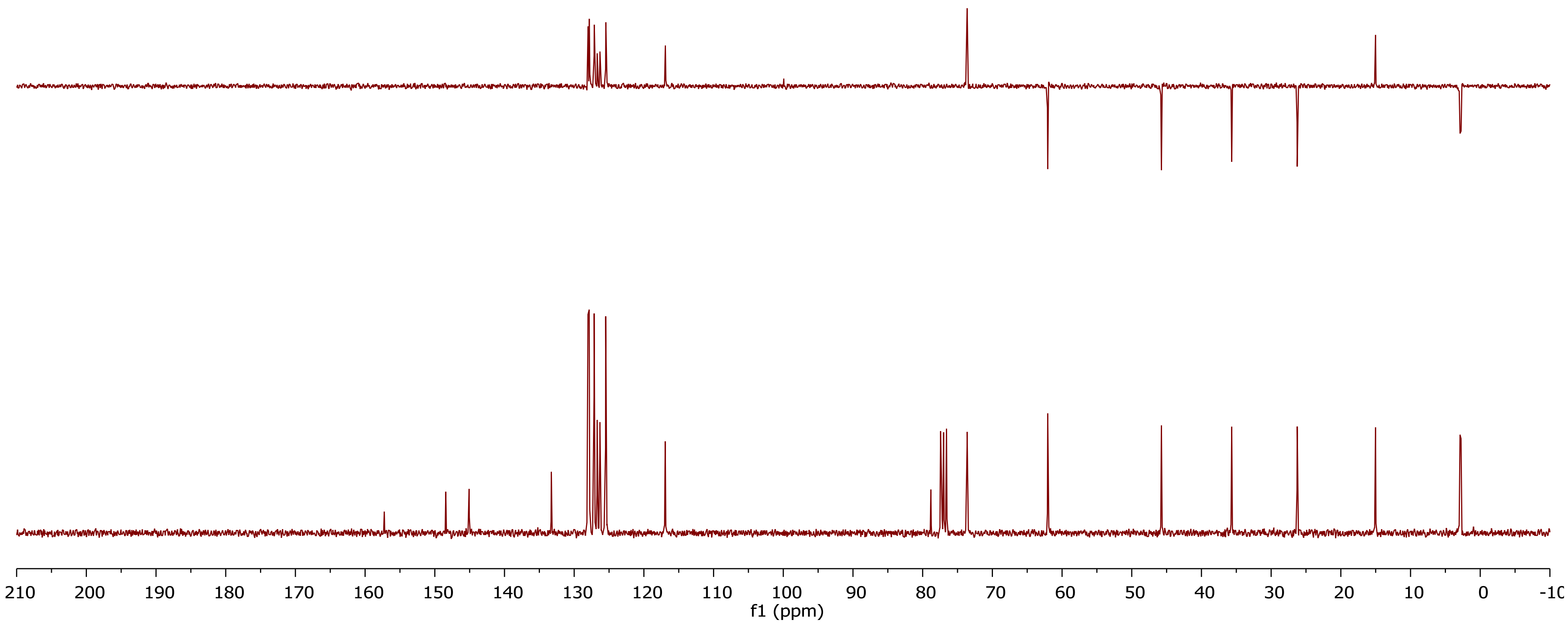

210
200
190
180
170
160
150
140
130
120
110
100
90
80
70
60
50
40
30
20
10
0
-10
f1 (ppm)

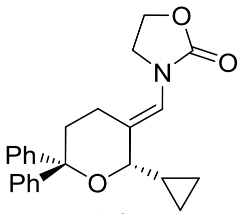

O
N
O
Ph
Ph
O

E-**4afd**

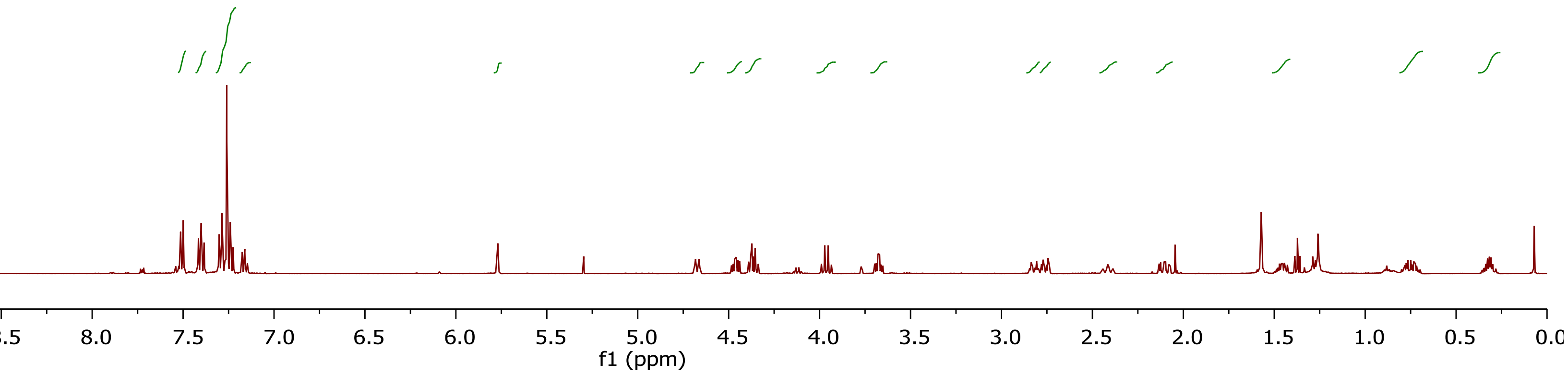

10.0 9.5 9.0 8.5 8.0 7.5 7.0 6.5 6.0 5.5 5.0 4.5 4.0 3.5 3.0 2.5 2.0 1.5 1.0 0.5 0.0
f1 (ppm)

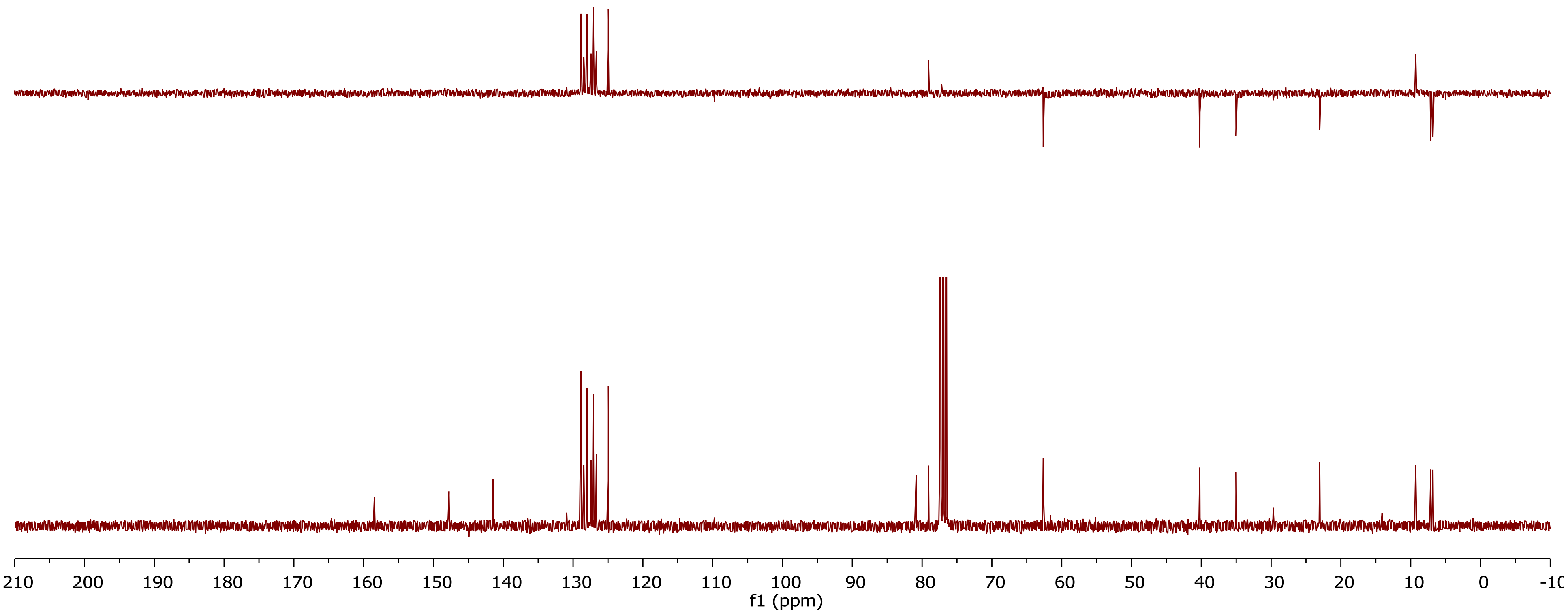

210 200 190 180 170 160 150 140 130 120 110 100 90 80 70 60 50 40 30 20 10 0 -10
f1 (ppm)

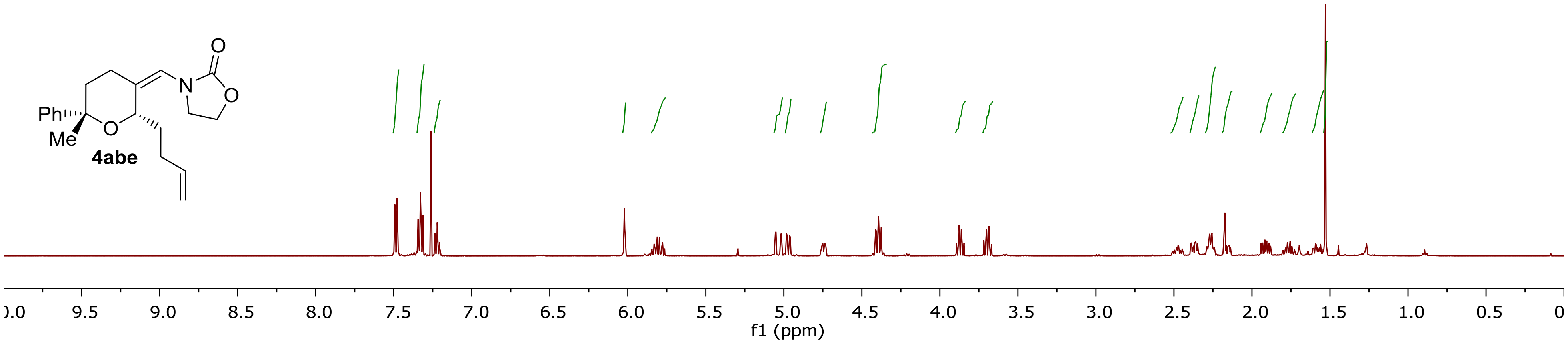
Ph
Me
O
N
O
O
4abe
f1 (ppm)

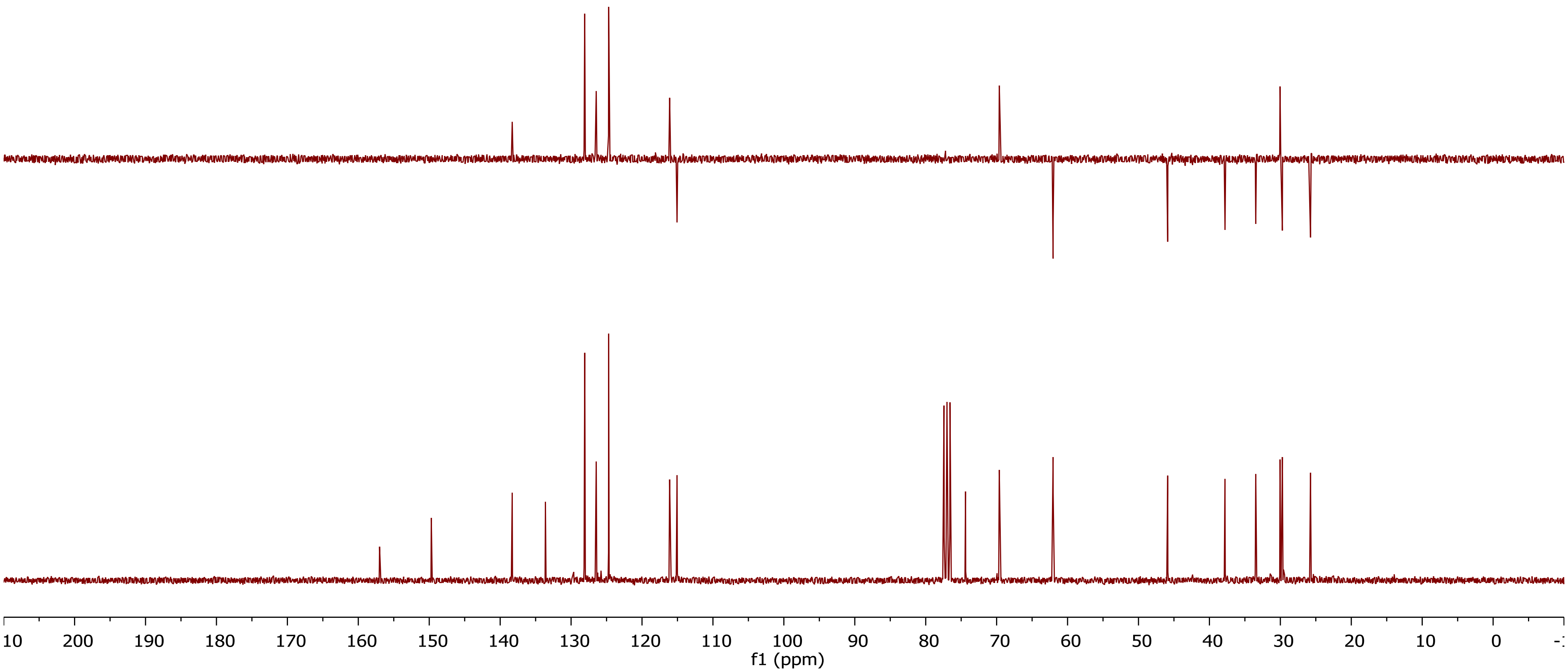
f1 (ppm)

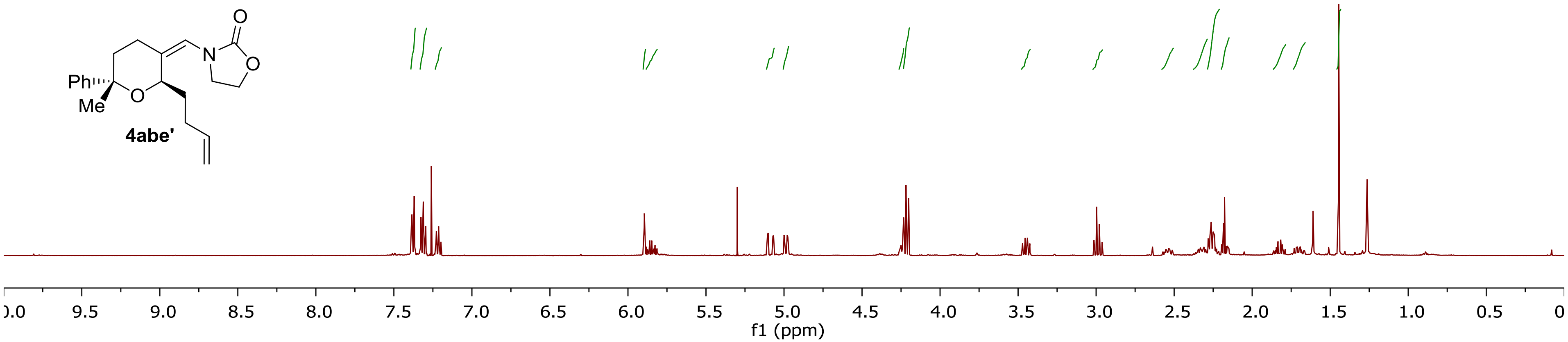

4abe'

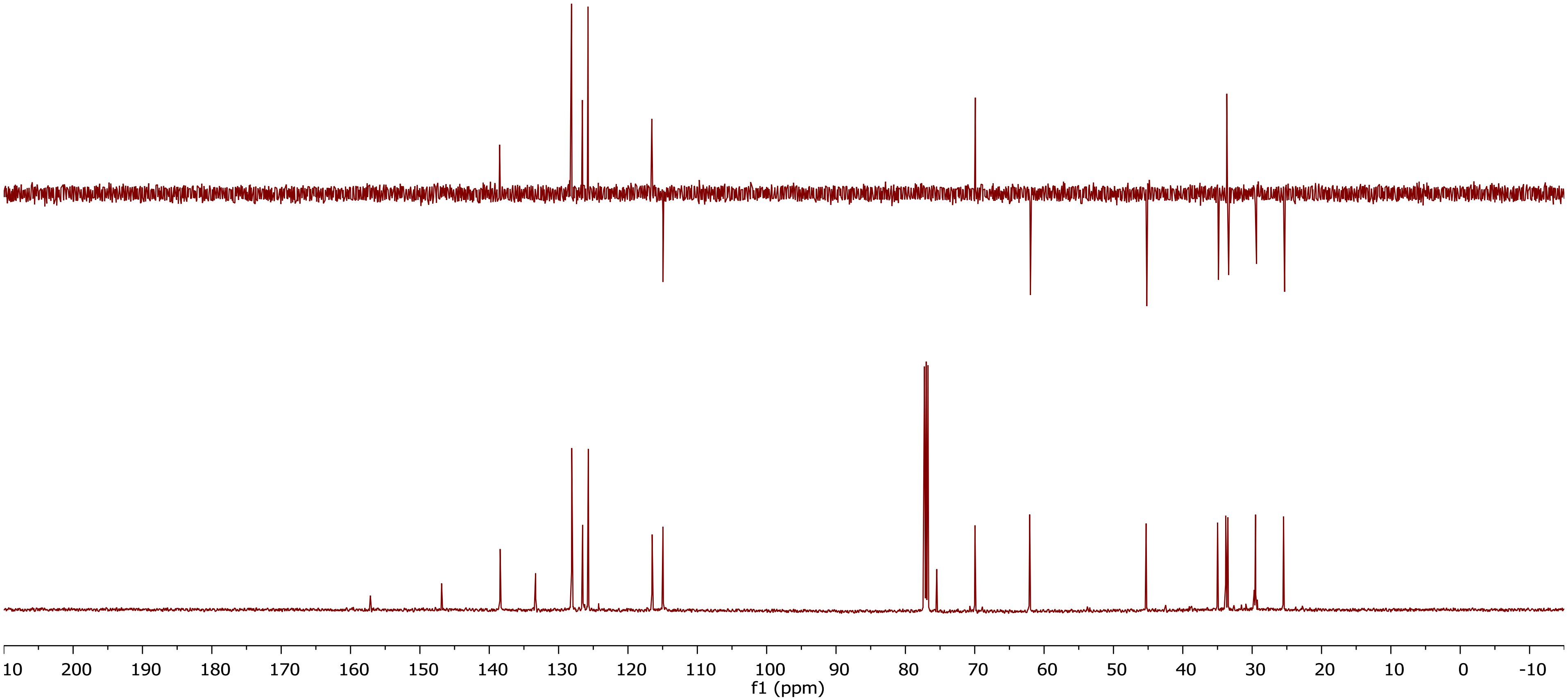

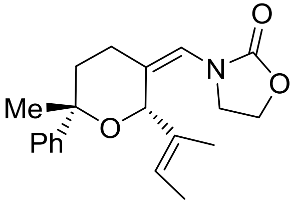

4abf

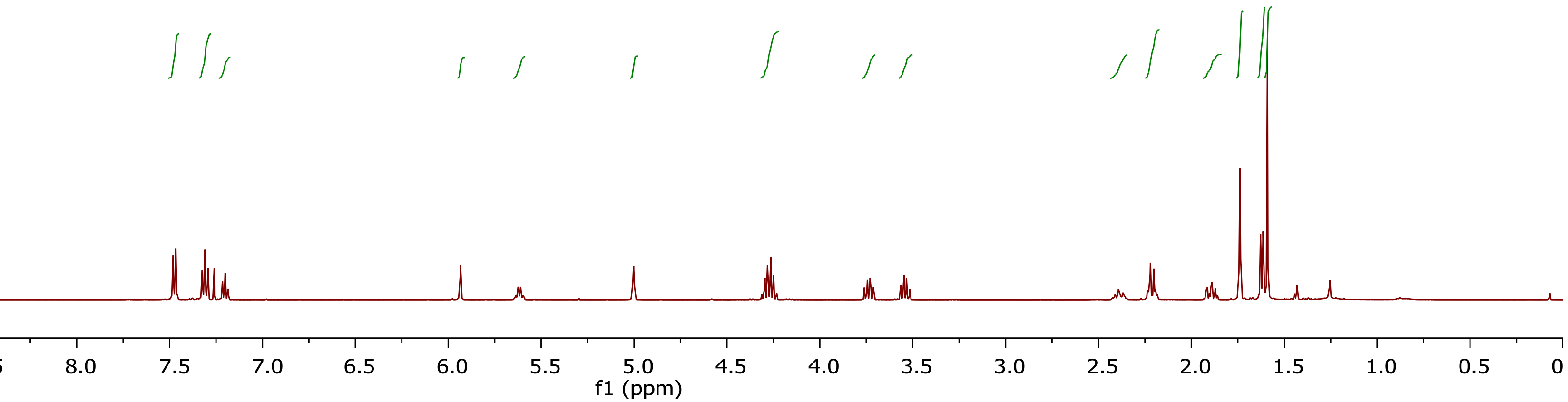

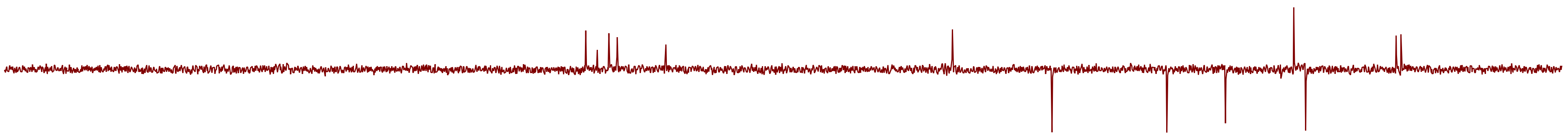

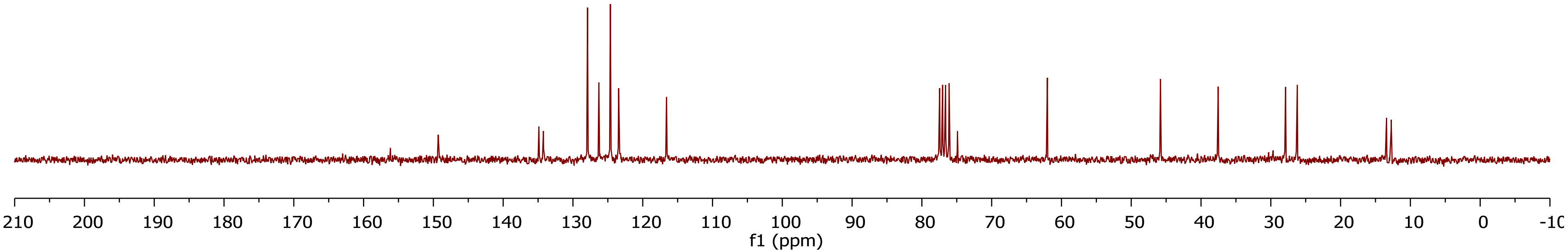

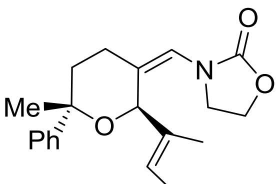

4abf'

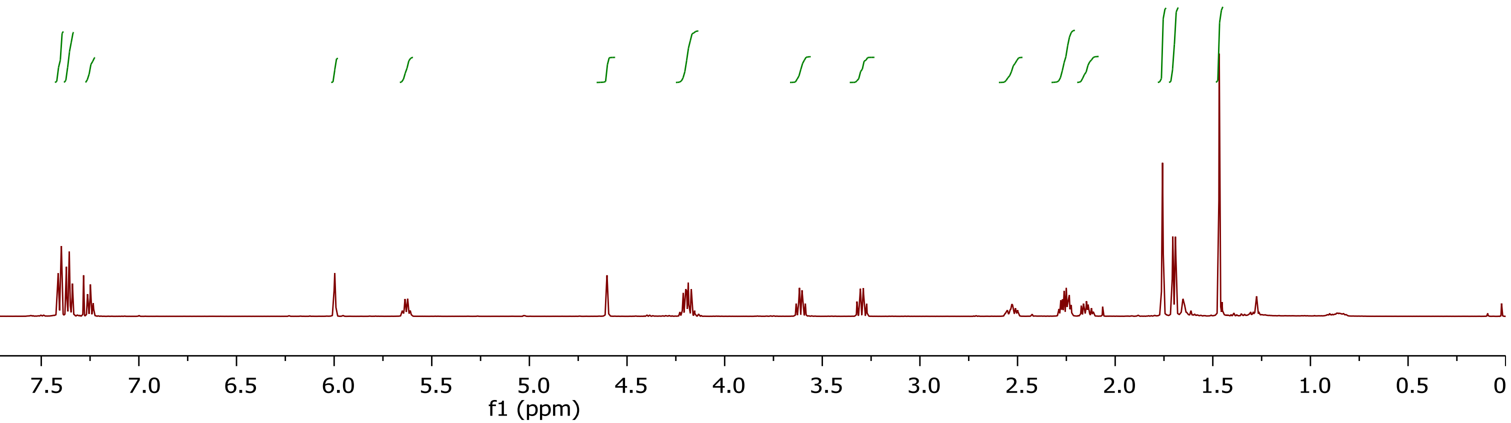

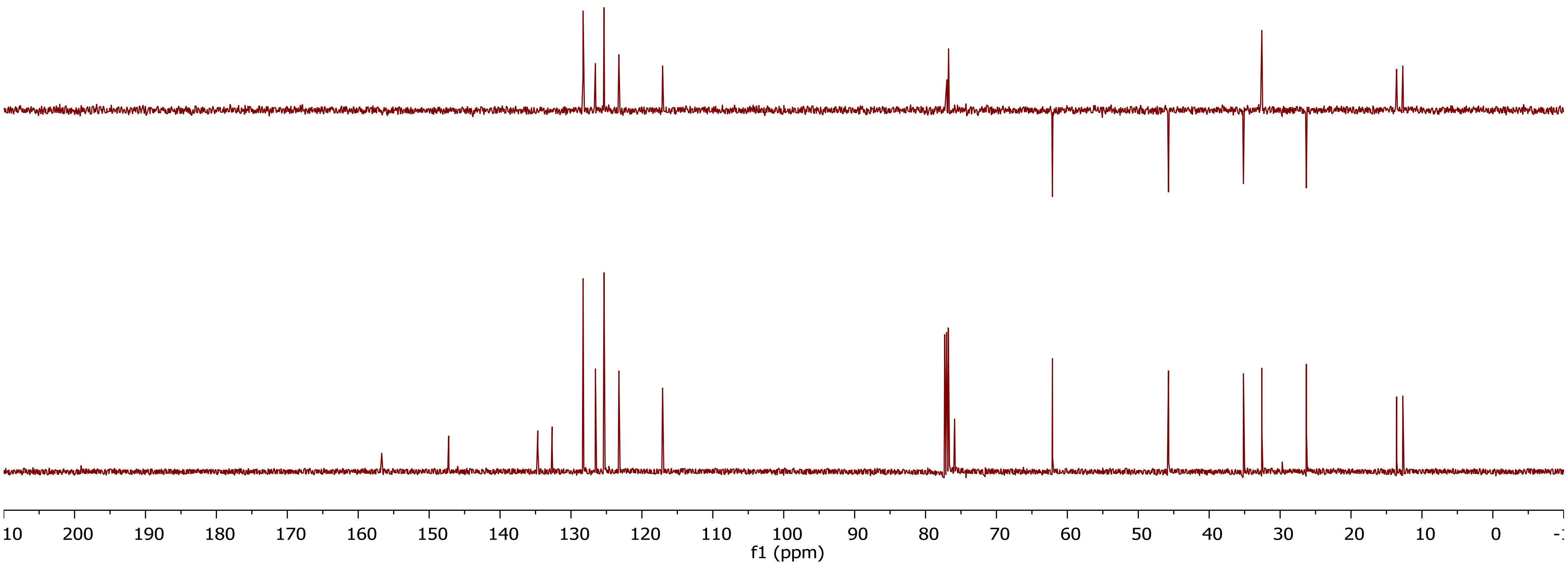

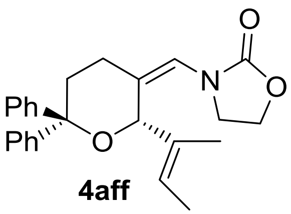

4aff

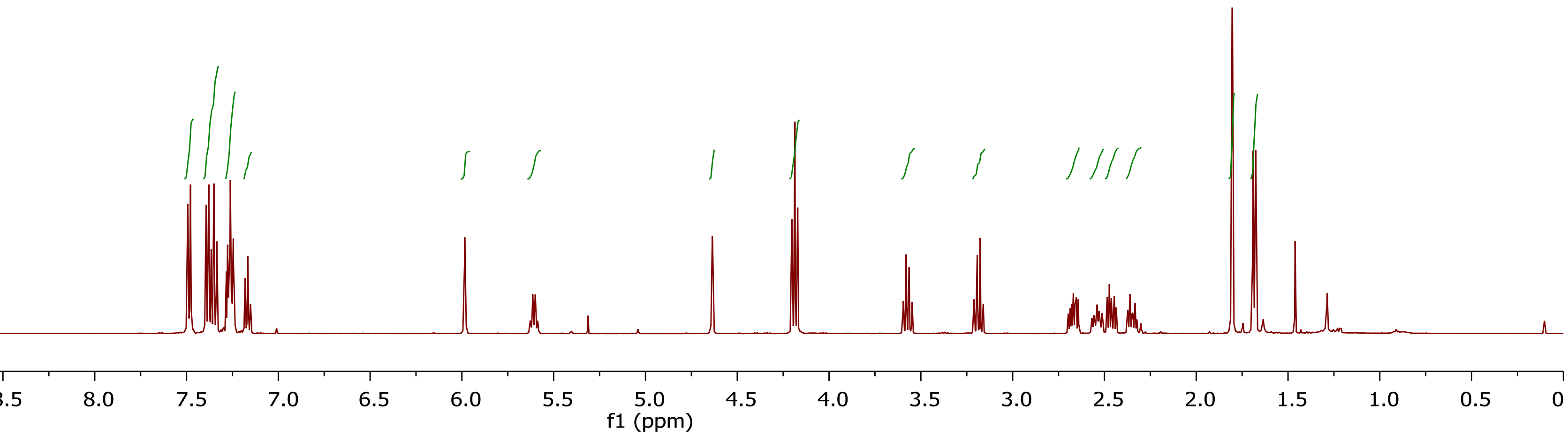

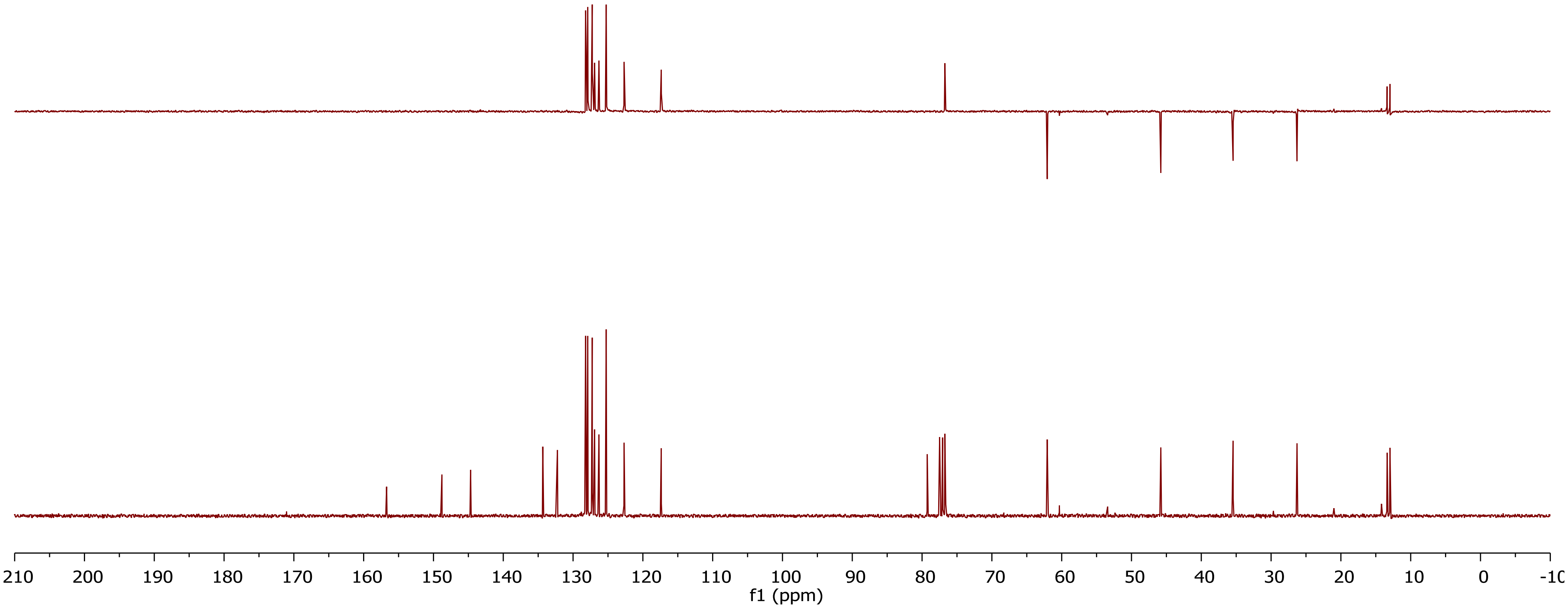

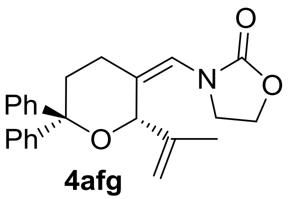

4afg

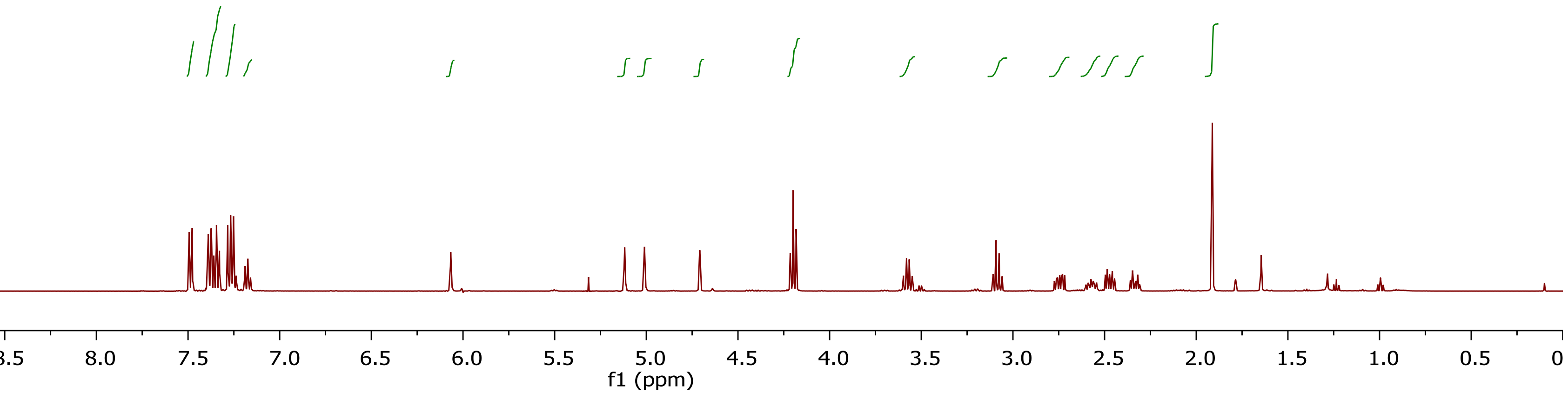

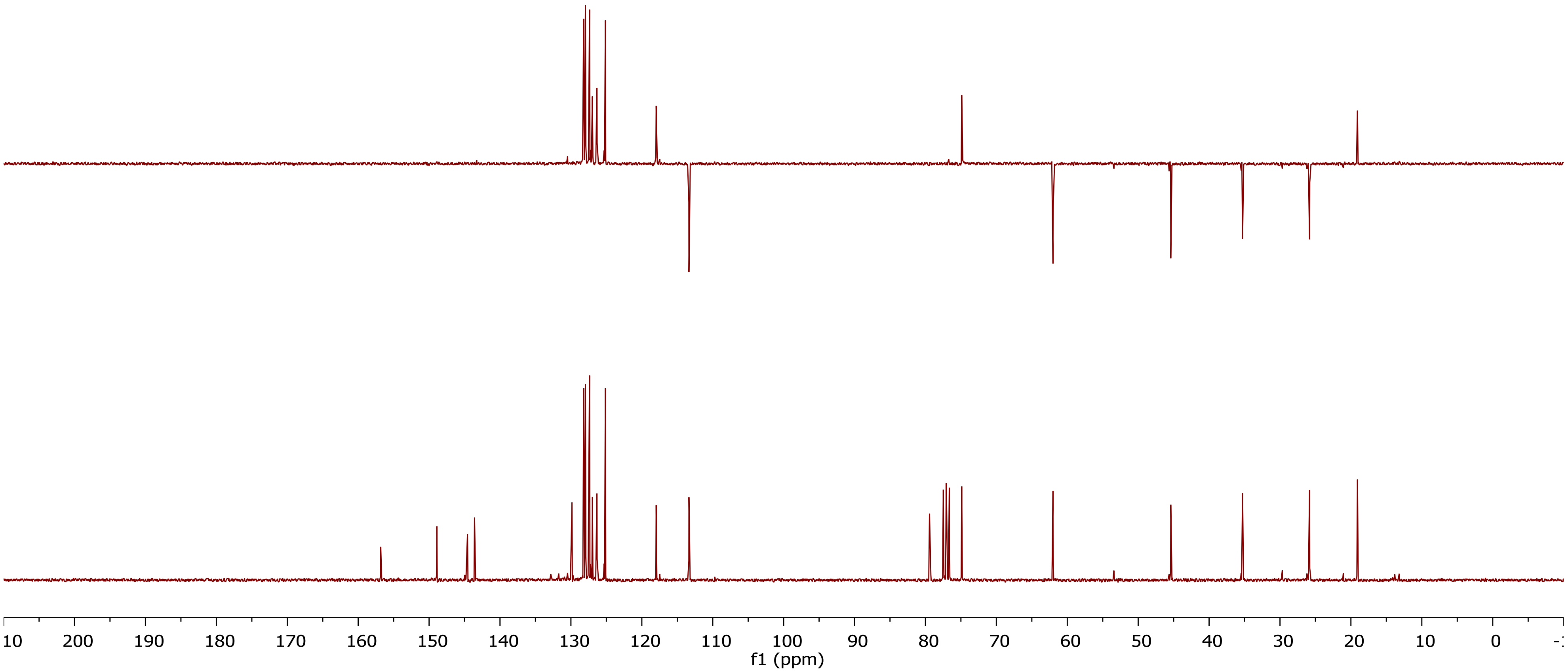

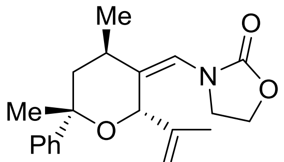

4bbf

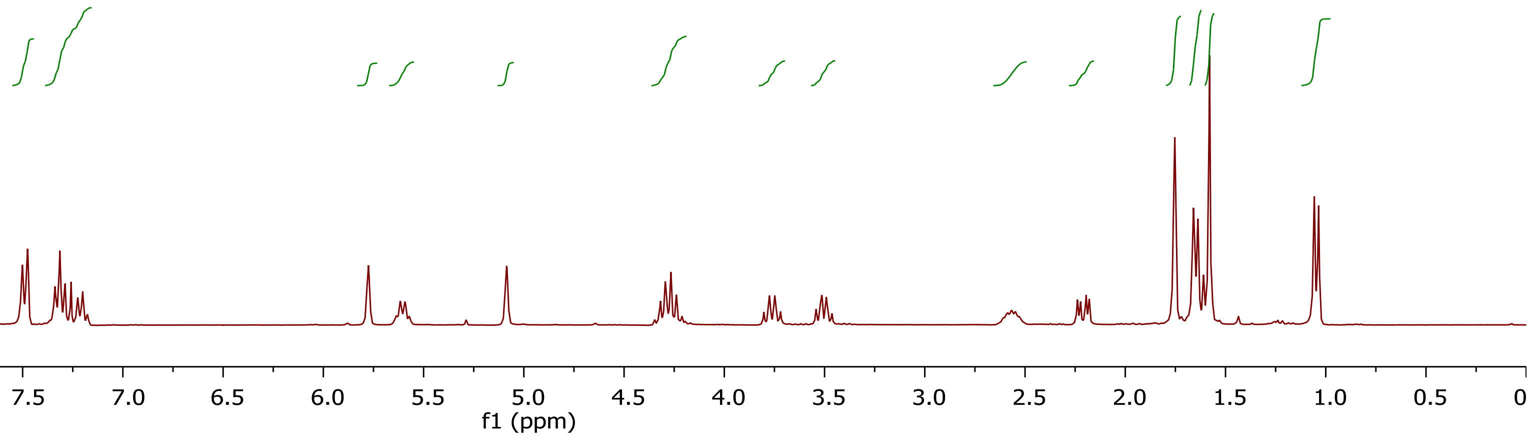

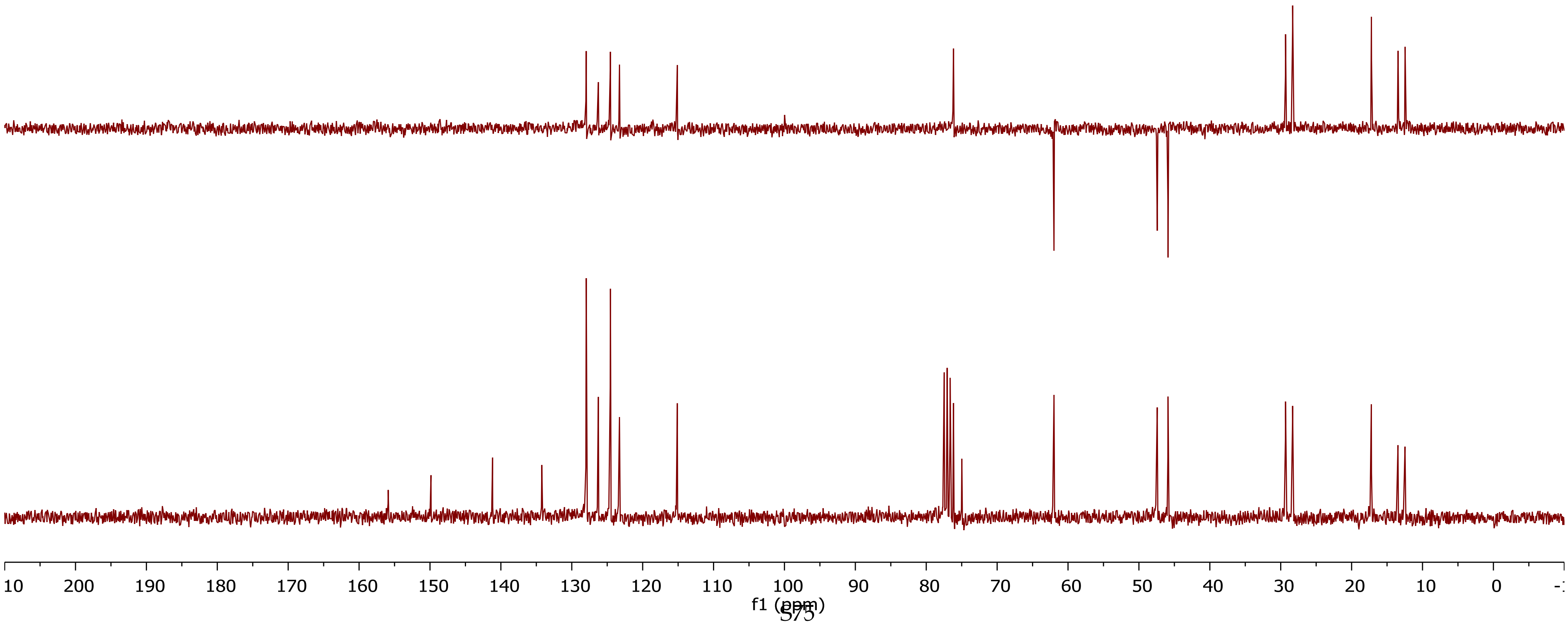

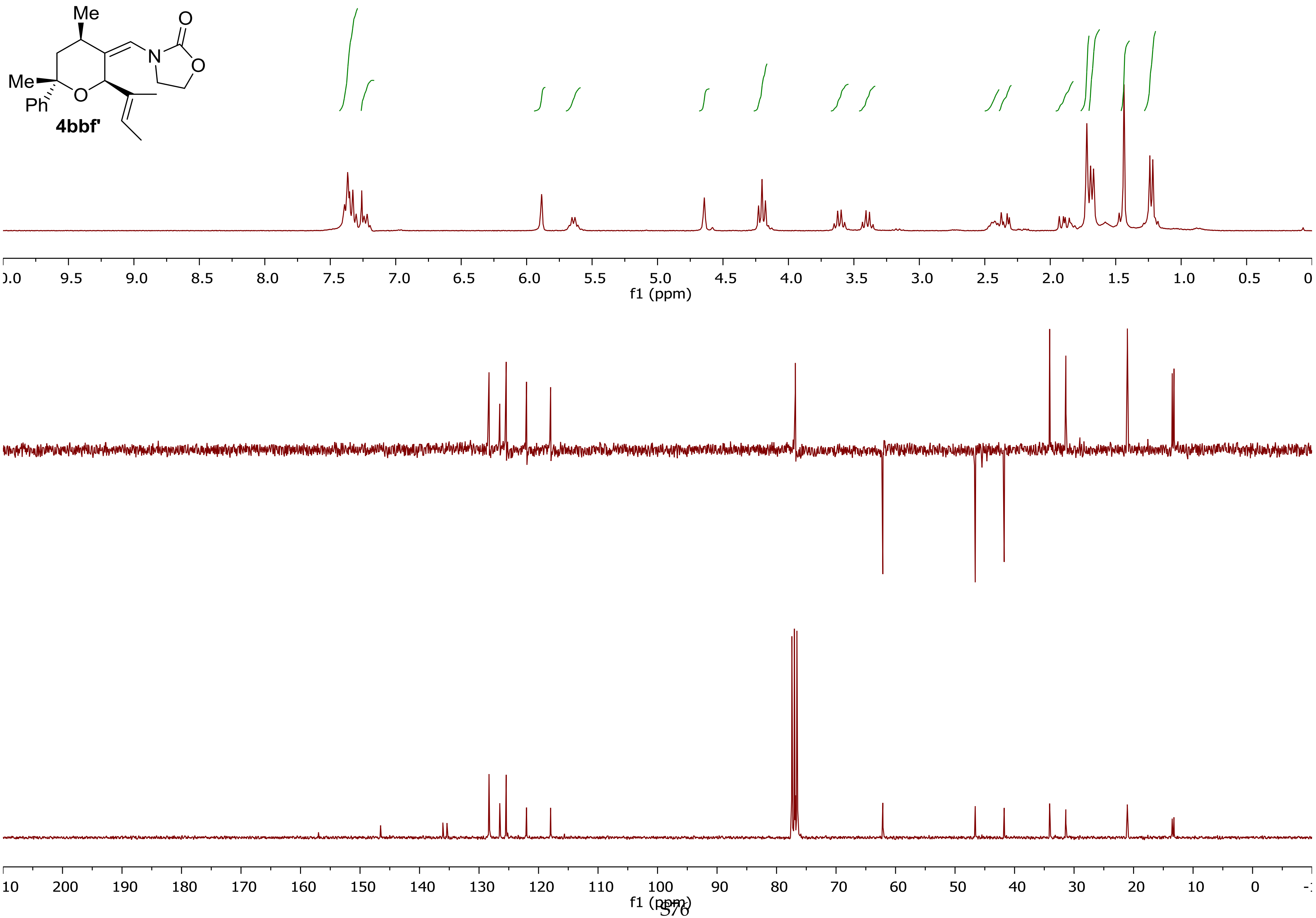

4bbf'

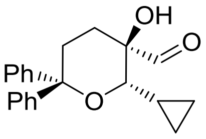

11

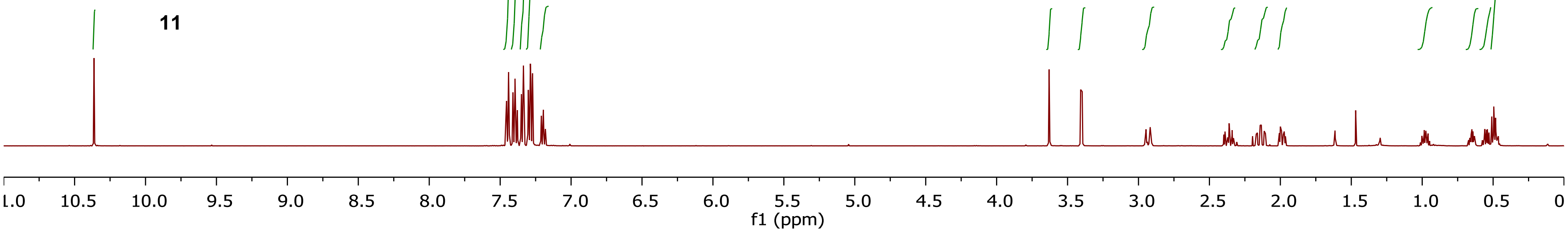

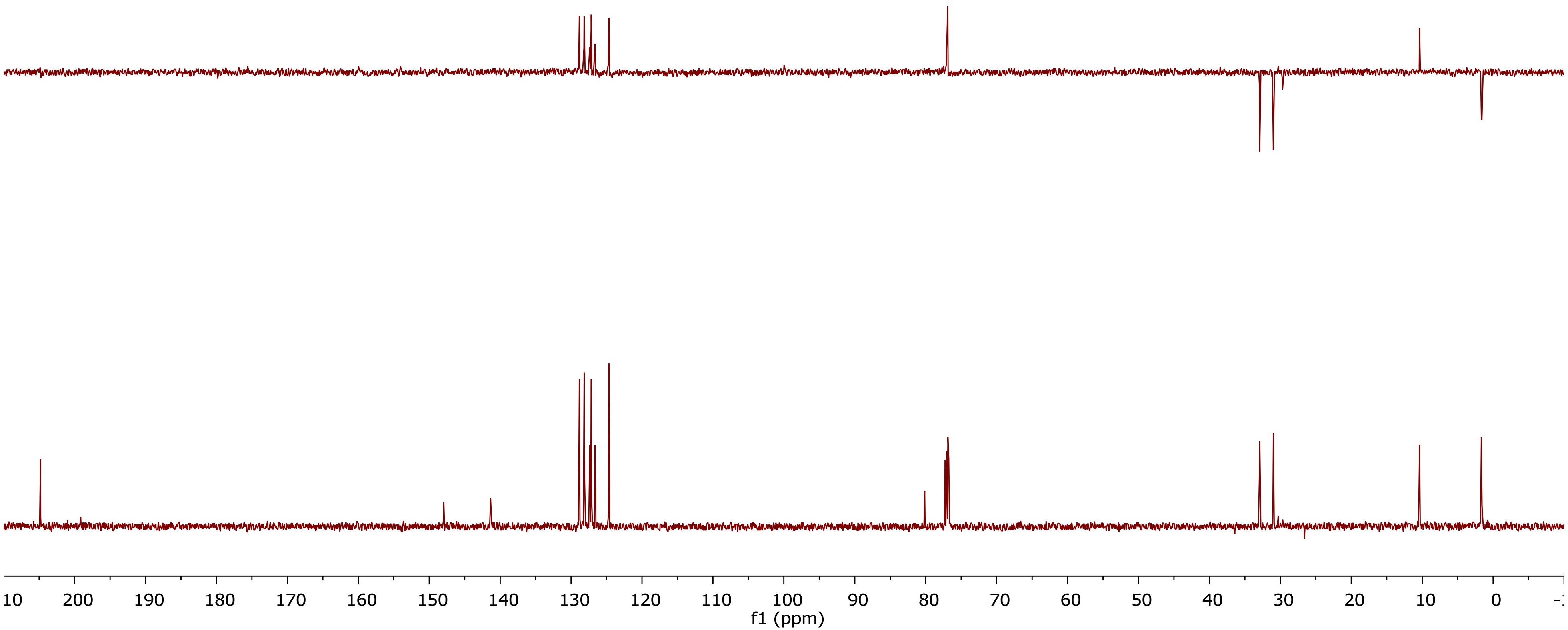

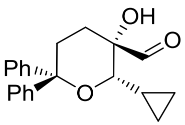

epi-**11**

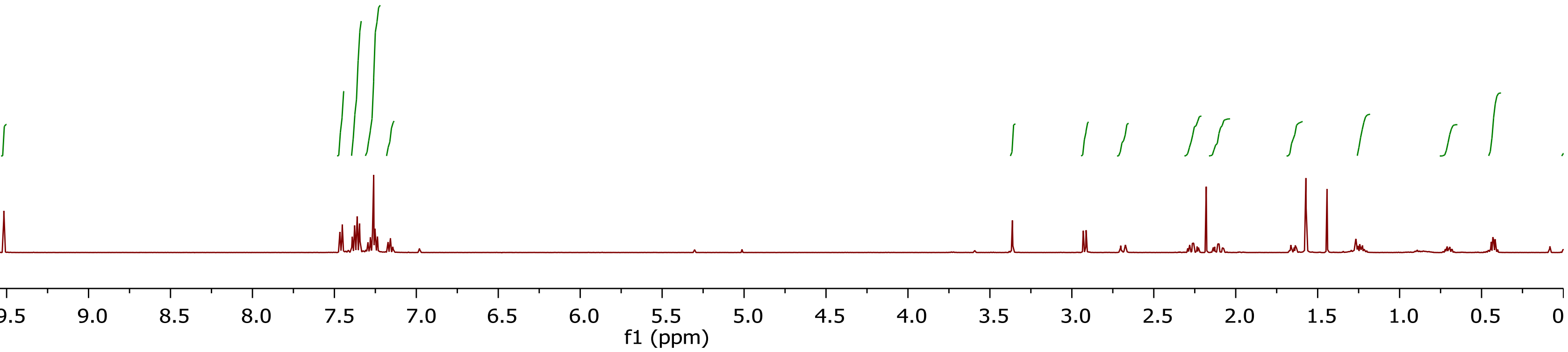

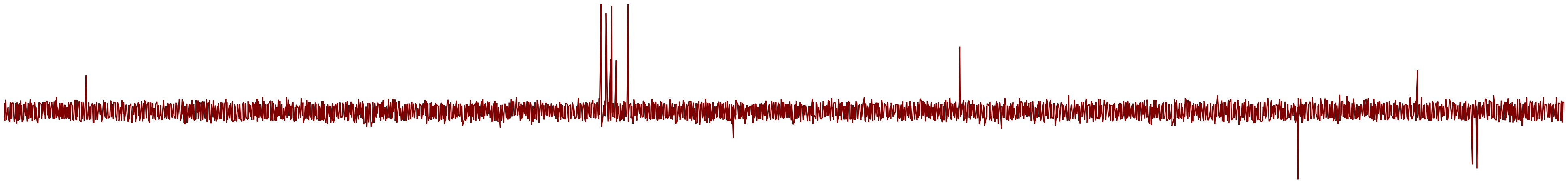

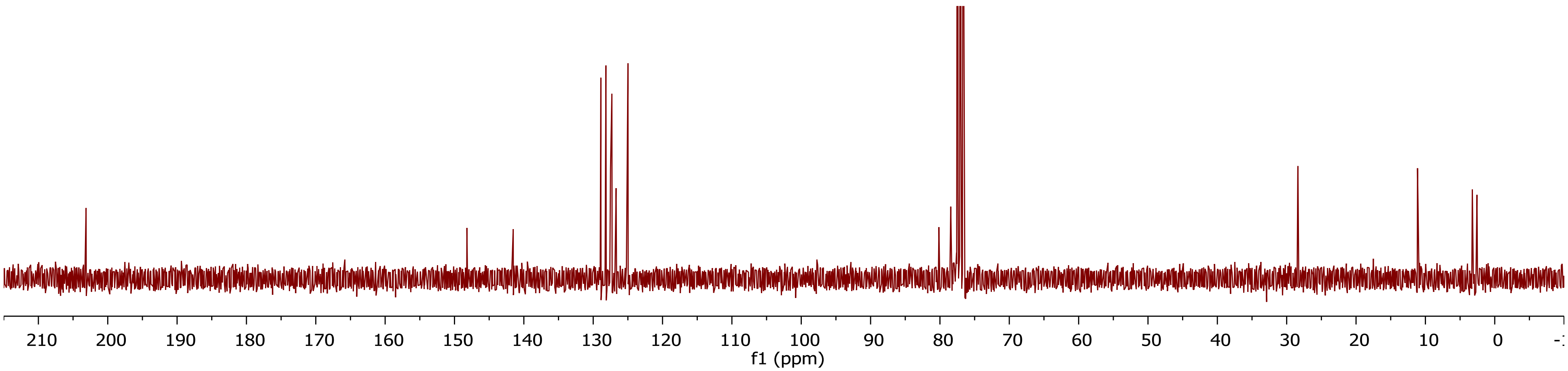

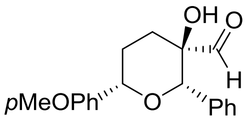

12

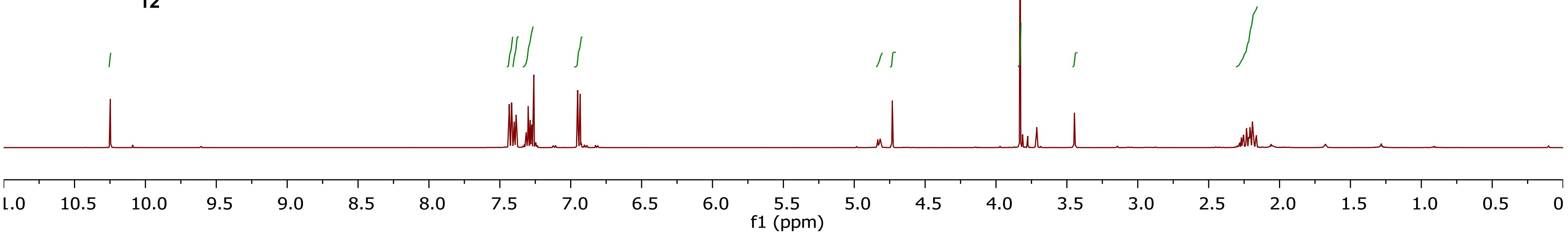

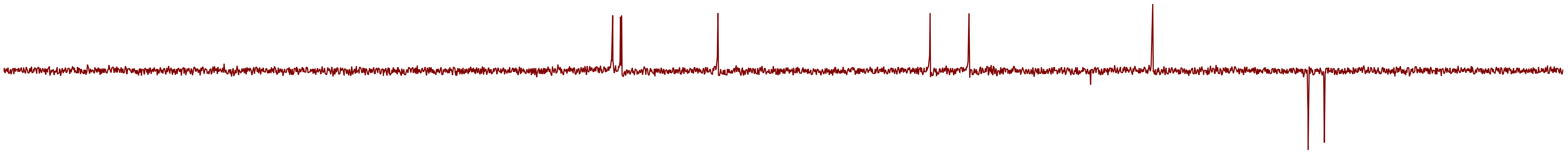

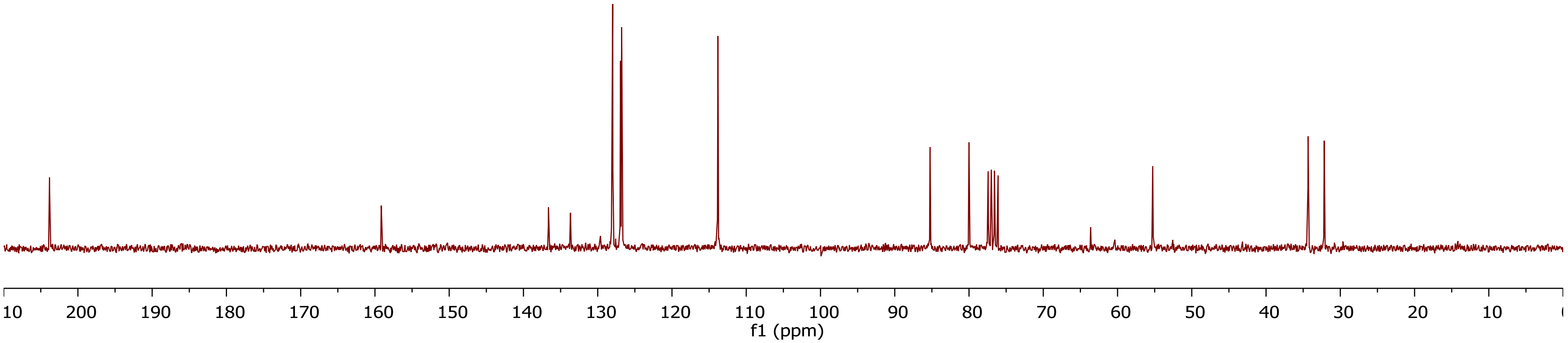

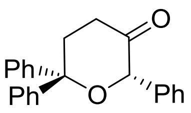
O
Ph
Ph
O
Ph

13

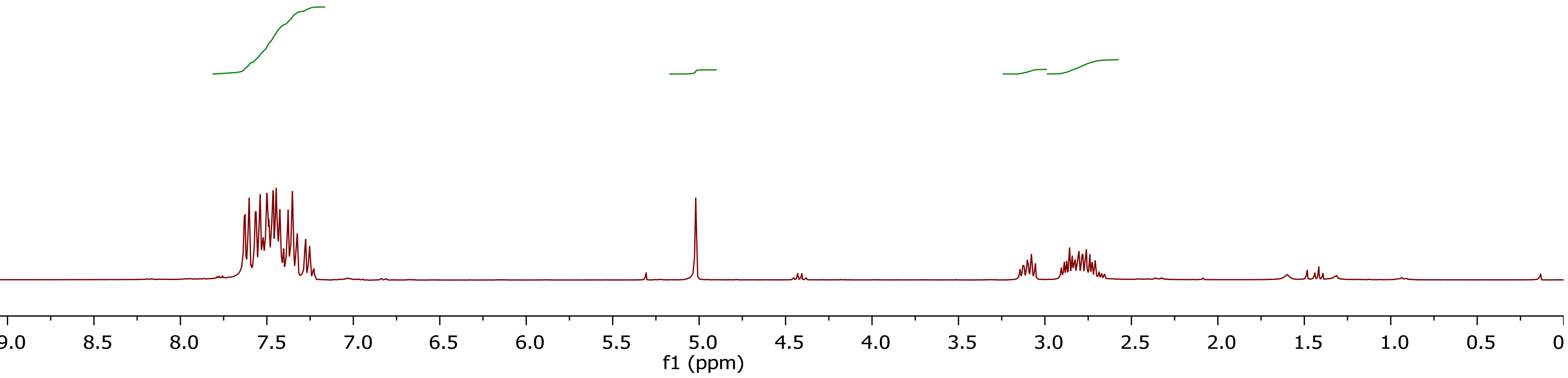
9.5 9.0 8.5 8.0 7.5 7.0 6.5 6.0 5.5 5.0 4.5 4.0 3.5 3.0 2.5 2.0 1.5 1.0 0.5 0
f1 (ppm)

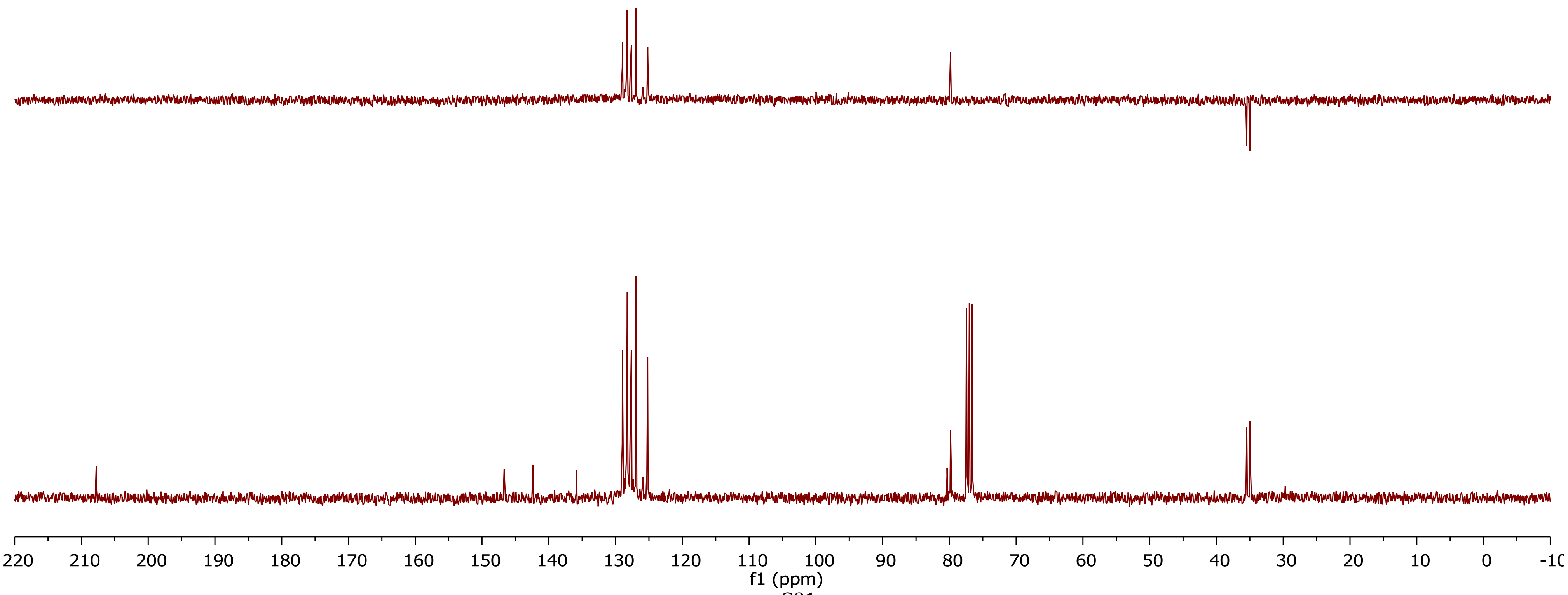
220 210 200 190 180 170 160 150 140 130 120 110 100 90 80 70 60 50 40 30 20 10 0 -10
f1 (ppm)

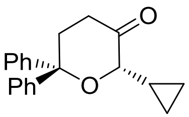

14

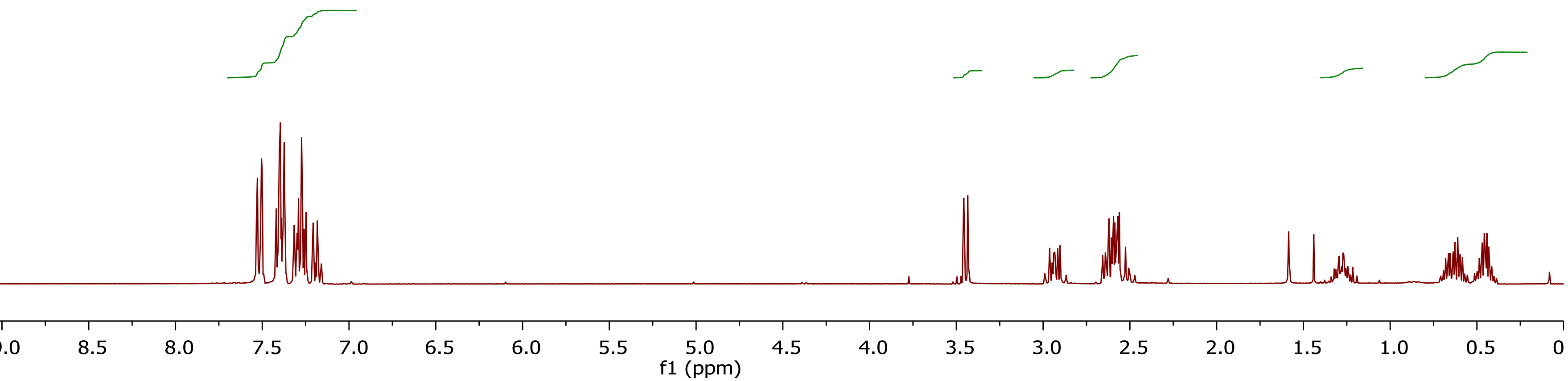

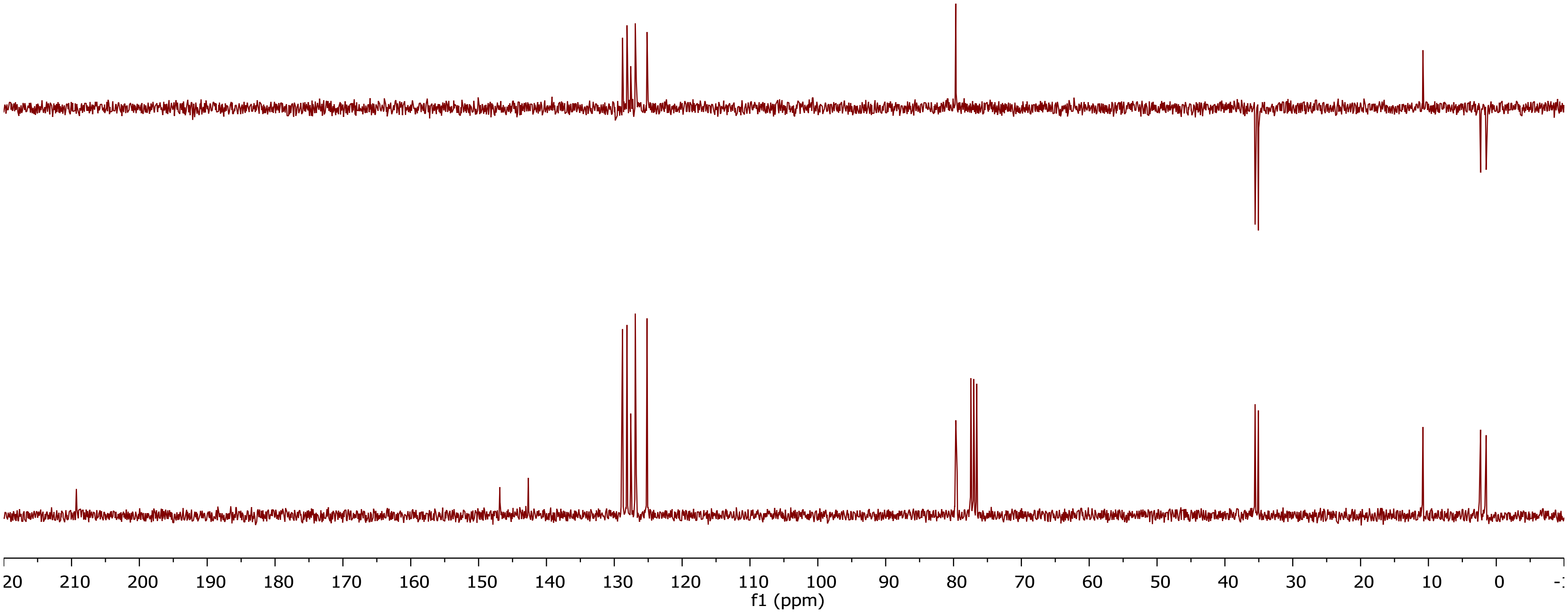

Supplement: Supplementary file 1 [file SC-006-C5SC00295H-s001.pdf]
